# Flora and fauna: how nonhuman species interact with natural and man-made EMF at ecosystem levels and public policy recommendations

**DOI:** 10.3389/fpubh.2025.1693873

**Published:** 2025-11-19

**Authors:** B. Blake Levitt, Henry C. Lai, Albert M. Manville, Theodora Scarato

**Affiliations:** 1 National Association of Science Writers, Berkeley, CA, United States; 2 Department of Bioengineering, University of Washington, Seattle, WA, United States; 3 Advanced Academic Program’s Environmental Sciences and Policy Division, School of Arts and Sciences, Johns Hopkins University, Washington, DC, United States; 4 Environmental Health Sciences, Bozeman, MT, United States

**Keywords:** electromagnetic fields, radiofrequency radiation, wildlife, nonhuman species, low-intensity effects, airspace-as-habitat, aeroecology, National Environmental Policy Act

## Abstract

In the last 60 years, there has been a steady increase in ambient exposures from nonionizing electromagnetic fields (EMF) between 0 and 300 GHz, primarily in the radiofrequency (RF) ranges between 30 kHz and 3 GHz. Each technology has introduced a layer of exposures with different transmission characteristics into the environment, creating what is today a broad scope of complex chronic, low-intensity, ambient exposures known to be biologically active in human and nonhuman species alike. The next generation of broadband technology employs a wide span of simultaneous frequency exposures for pervasive civilian use with signaling characteristics heretofore never deployed. Fifth and sixth generation (5G, 6G) networks utilize significantly higher areas of the electromagnetic spectrum >3.5 GHz unlike previous wireless technologies. The scale at which this EMF deployment unfolded has now reached documented proportions that simply do not exist in nature, creating 24/7 exposures to a novel energetic form of air pollution. While there are extensive local variations in exposure intensities, e.g., rural versus urban environments with proximity to transmission sources being the controlling variable, the advent of significantly increased satellites in low earth orbits, disseminating radiofrequency EMF (RF-EMF) toward Earth in broad radiation patterns, has now all but erased such demographic distinctions. Nowhere on Earth today is completely RF-EMF free. Nonhuman species are highly sensitive to the Earth’s geomagnetic fields which are used for orientation, migration, mating, food finding, territorial defense, and all of life’s activities. Compared to human abilities, myriad species have evolved an exceptionally sensitive physical array of electro/magneto-receptors with which to perceive environmental EMF often at, or very near, natural geomagnetic fields. Today’s exposures are capable, even at very low intensities, of disrupting critical fauna/flora functions. Any existing exposure standards are strictly for humans. Discussed are nonhuman unique physiologies and potential resonant matches at ambient levels today. Policy recommendations for wildlife protection includes discussion of “airspace as habitat,” adherence to existing laws, and mitigation that could include frequency re-allocation, redesign of hardware and network engineering, and societies moving away from certain competitive economic models, as well as EMF-free zones during migration and breeding seasons where possible.

## Part 1. Introduction: historical background

The extraordinary perceptual abilities of some nonhuman species—e.g., avian, insect, and cetacean abilities to migrate thousands of miles; mammalian and reptile species’ ability to predict coming storms and earthquakes days/weeks in advance; and nocturnal species’ keen vision and resourceful predation abilities among many others—have long fascinated humans and researchers alike. Early humans attributed these abilities to mystical powers. We now know much more about highly sensitive electromagnetic perception mechanisms (1) controlling these abilities in both fauna and flora and to many it is no less wondrous. It also, however, makes many species highly vulnerable to the predilections of the singular dominant species that mostly lacks such abilities—humans (The human eye is the only recognized organ evolved to perceive the electromagnetic spectrum, e.g., visible light).

Harold Saxon Burr, MD (1889–1973), who taught anatomy and neuro-anatomy at The Yale School of Medicine for 43 years, was the first scientist to conduct precise long-term field studies of natural electromagnetic field (EMF) effects on flora and fauna. Burr focused on establishing the first unified electrodynamic field theory of life, using hydra, frog, and salamander models because of their morphogenic properties (2, 3). Much was learned due to Burr’s painstaking pioneering work about amphibian electrophysiology and cellular microcurrent in wound healing (4, 5), including the electrophysiological properties of cell differentiation, and eventually dedifferentiation pertinent to all contemporary stem cell research. Today Burr’s work has implications regarding endogenous microcurrent and limb regeneration in humans, as well as dedifferentiation/stem cell/morphogenesis for cancer treatment and other healing modalities (6).

Burr also took exacting interest in the electric potential of trees, measuring how they changed in relationship to atmospheric alterations. He measured the electric potential in two tree species (maple [*Acer* spp.] and elm [*Ulmus* spp.]) located on one property, and another maple tree for comparison 40 miles (64.4 km) away. Numerous measurements via embedded electrodes recorded hourly readings from 1953 to 1961. Simultaneous records of temperature, humidity, barometric pressure, sunlight, moon cycles, sunspot activity, weather conditions, atmospheric-potential gradients, earth-potential gradients, and cosmic rays were correlated with tree electric potentials. It was found that the *electrical environment* correlated most closely with tree potentials while meteorological factors like light, temperature and atmospheric pressure did not in an immediate relationship (7). Burr also installed equipment that measured the potential between electrodes in the earth (about 10 miles/16.1 km apart) and the potential gradient of the air, finding that the air and earth potentials fluctuated exactly with the phase of the tree potentials.

Burr found that trees completely changed polarity when thunderstorms were approaching; the natural electrical environment correlated closely with tree potentials in a kind of entrainment to diurnal, lunar and annual cycles; and passing thunderstorms elicited anomalous behavior in the trees in direct parallel to measurements with the earth electrodes.

There are no other long-term field studies as detailed as Burr’s of atmospheric EMF effects on plant species. His early research set the framework regarding how plants sense natural EMF and take immediate information from it. It is only recently that a research team in Italy (8) took up the mantle with an interest in spruce tree (*Picea ables*) reactions to solar events to assess their individual and collective bioelectrical responses to a solar eclipse. They found trees anticipated the eclipse and synchronized their bioelectrical behavior hours in advance, with older trees showing greater anticipatory behavior in early time-asymmetry and entropy increases. They concluded that a collective relationship between trees exists with perception of upcoming celestial events; that it is shaped by individual tree age, and that there’s a collective history pointing to the significance of synchrony in plants with natural phenomena.

As with flora, so with fauna.

### Ecosystem perspective and low-intensity exposures

Similar synchronicities as noted above with the natural environment exist throughout diverse species. All of life evolved within the Earth’s natural electromagnetic fields. This paper addresses potential adverse anthropogenic effects at ecosystem levels due to human technology. We examine nonionizing bands of the electromagnetic spectrum (EMF 0–300 GHz) which include the frequencies that fall between visible light below the ultraviolet range and the Earth’s natural static fields with a particular focus on the radiofrequency (RF) bands between 30 kHz and 300 GHz—the primary wireless technologies contributing to rising ambient background levels today [see Supplementary material 1 in Reference (9)]. Since biological effects of static and ELF-EMF and RF are inevitably related, discussion of studies on these fields are juxtapositioned throughout this text.

In its natural state, very little radiofrequency radiation (RFR) reaches the Earth’s surface. Aside from the Earth’s natural extremely low frequency (ELF) direct current (DC) static magnetic fields and the Schumann Resonances, lightning and sunlight would primarily comprise our ecological exposures to the electromagnetic spectrum.

Of particular concern are the low-intensity anthropogenic exposures comparable to environmental exposures today (see Supplementary material 1). Animal species move in and out of these near-and far-fields constantly. At 100–200 ft. (30.5–61 m) distance from cell phone antenna arrays, an animal moving through the area can be exposed to a power density (energy moving through space) of 0.001 mW/cm^2^ (i.e., 1.0 μW/cm^2^). Depending on the frequency and object exposed, e.g., bird, insect, etc., the Specific Absorption Rate (SAR) at such a distance can be 0.001 W/kg (i.e., 1.0 mW/kg). Avian, bat, and insect species can often reach very close lateral proximities to transmitting infrastructure like small cells and cell tower antennas and experience much higher exposures. Depending on radiation patterns, absorption rates for small animals at ground level can be significantly higher too due to their smaller size. In Supplementary material 1, there are 33 starred citations at or significantly lower than SAR of 0.001 W/kg that include myriad effects capable of translating to effects in the wild.

### Unique wildlife sensitivities

All living organisms evolved in a matrix of the Earth’s static geomagnetic fields. Biological disturbance occurs at very low intensities to unfamiliar fields, sometimes even far below geomagnetic fields. Natural cellular biocurrent—minute electric current involved with all ‘excitable’ cells, e.g., nerve and muscle cell activities, can also be disturbed by exposures to various EMFs.

It has long been known that the Earth’s geomagnetic field is needed to coordinate embryonic development in many species, and provides directional information for many migratory species, including: amphibians, birds, fish, turtles, insects, and even some bacteria (See Supplementary material 2 for a sampling of research citations per species). Natal homing behavior in some turtle, salmon, eel, cetacean, avian and insect species, e.g., Monarch butterflies (*Danaus plexippus*), also rely on natural geomagnetic fields to guide them over thousands of miles annually (10–13). Species that migrate according to the geomagnetic field are capable not only of detecting the field but also the orientation of the field.

Highly sensitive biological mechanisms are widely found in many nonhuman species in specialized electro/magneto-receptor cells that differ greatly between species according to environment. These enable living organisms to detect the presence, and immediate changes in, environmental fields at very low intensities. Unfamiliar low-intensity anthropogenic fields can disturb perceptions (14).

In aqueous environments with high conductivity and low impedance, electric fields as low as 5 nV/cm (similar to that produced by the muscles of a shrimp) can be detected in many species. Electroreceptors are the primary mechanisms and minute electric currents/fields are used to find mates and food and thought to be a form of communication in many species (15). In elasmobranch fish (sharks, rays, and skates), electric fields are perceived via highly conductive gel-filled canals near the head called Ampullae of Lorenzini, and magnetic fields through receptors in their mouths and eyes (16–18). Anthropogenic RF may even play a role in increased cetacean strandings in specific areas near coastal military radars (19).

In ground-based and aerial environments, electromagnetic perception is via a combination of a natural ferrous mineral called magnetite that has been found in every species studied (20), and through a complex free-radical pair reaction and conversion of electrons in a group of protein compounds known as cryptochromes (21, 22).

Cryptochromes are found in the retinas of nocturnal migratory songbirds showing complex communication with the brain for orientation when relying on magnetoreception (23, 24). Cryptochromes are also found to be a critical magnetoreception component in fruit flies [*Drosophila melanogaster* (25, 26)] and are also present in the retinas of other animals (27). Research also indicates cryptochromes in plants, which may be responsible for the effect of EMF on plant growth (28, 29). Cryptochromes are also known to be involved with circadian rhythms (30).

Oscillating magnetic fields have been reported to disrupt the migratory compass orientation in migratory birds (31–33), which are known to use a radical pair mechanism located in the eye that provides compass-like directional information, while magnetite in the upper beak senses magnetic intensity, thus providing positional information (34, 35).

The above are some examples of the information available; for more see Supplementary material 2.

### What the research shows

Anthropogenic EMFs in all frequencies affect biological systems in vastly different ways than natural fields (36). Decades of *in vitro* and *in vivo* studies have found effects at very low intensities comparable to ecosystem levels today. Researchers have been experimenting on animal models for over 100 years and know how EMF couples with all animal taxa. Many biological effects have been documented at low intensities comparable to what wildlife experience within 200–500 ft. (61–152 m) of a cell tower. Reported effects include: genetic, growth, and reproductive alterations; increases in permeability of the blood brain barrier; stress protein increases; behavioral changes; molecular, cellular, and metabolic alterations; and increases in cancer risk (see Supplementary material 1).

Major literature reviews exist in all frequencies on low-level EMF effects to non-human species (37–46). Most environmentalists and regulators are unaware of this body of work.

### Ecosystem effects

Between 2021 and 2022, three of the authors (BBL, HCL, AMM) published four papers (9, 47–49) that matched for the first time the measured rising ambient RF levels in various environments to the low-intensity effects literature comparable to far-field exposures that wildlife encounters today. They found enough recent research on increasing ambient levels, and an overwhelming amount of evidence in all five animal kingdoms and taxa studied, to warrant concern. Extensive tables of biological effects contained in the papers’ Supplements found: 27 studies on measured rising background levels; 123 studies on combined fauna/flora effects; and 59 studies on genetic effects (12 on ELF; 47 on RF). For flora: 16 studies were found on static magnetic fields; five studies on pulsed magnetic fields; eight studies on ELF magnetic fields; and nine studies on RF. The flora studies alone pointed to the fact that plant growth and physiology—with root systems anchored in the ground while tree tops thrive in the air—are affected by exposure to EMF in complex synergistic ways and are susceptible to multi-frequency exposures throughout their life spans.

Three of the above mentioned papers (9, 47, 48) when combined, cited over 1,000 studies and many more have been added to the database since the 2021 publications. Ambient RF-EMF low-intensity levels are a growing ubiquitous unrecognized environmental cyto-and- genotoxin: in a 2010 paper on cell tower exposures, two of the authors (BBL, HCL) cited 57 low-intensity papers (50); in the 2021 papers 123 were cited (47); today (October 2025) there are at least 248 updated studies (see Supplementary material 1). Increases in ambient levels between the 1980’s and today directly parallel unprecedented species losses, among other factors like climate change and habitat destruction (46).

A clear pattern emerged in the flora studies (over 200 were found): plants, trees, and seeds respond positively, e.g., increased germination, cell growth, and vitality to natural static and ELF-EMF, but react adversely to RFR with failure to germinate or thrive, and with increased dieback. One explanation is that static and ELF-EMF’s positive effects and RFR’s negative effects is based on morphological differences in plant cells (versus animal cells) in that plant cell walls are active organelles that regulate cell division and growth, as well as control cellular communications. Plant cell walls contain significant amounts of water. It is thus possible that cell-wall absorption of RF causes a microthermal effect that adversely affects plant cell functions, including apoptosis, while ELF-EMF is not likely to cause such heating effects. [See Supplementary material 4 in reference (47)].

Small cells mounted on utility poles now commonly used for 5G bring RF very close to flora. Progressive defoliation has been documented in Europe (51) in slow tree dieback after cell towers/antennas were installed nearby. The same can be expected of small cells. This is particularly relevant for urban forestry efforts, parks, and tree cover in a warming world. Cell infrastructure is the largest contributor to increasing ambient exposures over the last three decades (52).

Extrapolations from laboratory research to the wild are difficult to make. In the wild, there is more genetic variation and mobility, as well as variables that confound precise data assessment. But there is clearly a growing body of evidence that finds damage to numerous wildlife species near communications infrastructure, especially where radiation measurements have been made. Far more field research needs to be done as the ambient levels continue to rise with each new technology. This is a serious new environmental concern.

### 5G is different

Novel EMF exposures do not allow living organisms to adjust since signaling characteristics change rapidly as new technologies are developed. Species cannot adapt or evolve with them.

Compared to natural geomagnetic fields and Schumann Resonances, anthropogenic EMFs, since their inception, have infused the environment with unusual signaling characteristics, odd waveforms, and modulations at intensities not found in nature. But 5G utilizes for the first time even more novel signaling characteristics—i.e., phased arrays with high peak exposures, Massive MIMO (multiple-in, multiple out sequencing), and focused beam steering that targets devices but passes through everything in its path—at higher frequencies >6 GHz that are capable of affecting insect populations in particular. Nothing like 5G has ever been used in broad civilian applications. And it is being deployed without environmental review of any kind.

5G in particular may impact insect populations as its carrier frequency, (millimeter waves [mmw]), couples maximally with some insects (53). Insects the size of fruit flies reach peak absorption in the upper microwave bands. Both thermal and nonthermal effects will likely occur.

Insects are inefficient thermoregulators and are especially vulnerable to temperature changes. One review of 73 reports found extinction rates had greatly accelerated. Insects in particular showed dramatic declines that could lead to a 40% extinction rate over the next several decades (54).

All radiofrequency transmissions contain ELF components in the form of pulsations and other signal characteristics used in modulation (55, 56), including broadband 5G. Honey bees are a seminal thoroughly modeled insect species for ELF and RF, including analysis of ecological effects. In a recent well-designed study, Molina-Montenegro et al. (57), found adverse effects in honey bees (*Apis mellifera*) exposed to ELF-EMF near high-tension powerline pylons in California, U.S. They assessed effects on pollination efficiency using both field and laboratory experiments via measured levels of gene and protein expression in metabolic pathways involved in stress and behavioral responses; and secondarily on honey bee behavior and seed production by measuring pollination failure on plant communities richness/abundance. They found that ELF-EMF caused a strong stress response in bees as demonstrated by enhanced heat-shock proteins (Hsp70) and antioxidant gene expression as well as increased expression of behavior-related genes. Ecological impacts to California poppy plants (*Eschscholzia californica*) growing near powerlines translated to lower floral visits by honey bees which then reduced seed production, which, in turn, lowered diversity and plant abundance. Adverse ELF effects between bees and plants were a proven closed loop. Negative effects were related to distance from the source and were observed only when transmission towers were online. They concluded that ELF-EMF has impacts on honey bees leading to ecological diminishment. There are many studies on honey bees going back decades (47, and see Supplementary material 2).

Environmental EMFs may also play a role in colony collapse disorder in bees (58), among many other factors.

5G’s potential lethality to insects has recently been studied by researchers using computer modeling of honey bees and other insects based on frequency alone to determine power absorption rates with an eye toward understanding higher frequency effects >6 GHz. Thielens et al. (59) used numerical simulations of honey bee models to determine absorbed RF power as a function of frequency between 0.6 and 120 GHz which includes 5G and 6G. Computer simulations were then matched for the first time with *in-situ* measurements of environmental RF-EMF exposure near beehives in Belgium to estimate realistic exposure and absorbed power values in honey bees. They found that a relatively small shift of 10% of environmental incident power density from frequencies below 3 GHz used in 2–4G networks to the higher frequencies for 5G leads to a relative increase in absorbed power by a factor higher than 3. Frequency was the determining component. Thus, the shift to higher frequencies alone is detrimental to insects because their absorption rate is higher and thermoregulation abilities are lower.

Toribio et al. (60) investigated honey bee absorption rates in near-field exposures at 5G frequency ranges between 6 and 240 GHz, and also looked to see if the radiation performance of antennas remains stable when an insect is nearby. Using numerical simulations and micro-CT scanning, they found that in near-field exposures, the absorbed power can increase by a factor of 53, e.g., higher by a factor of 7 than the reported far-field increases noted in the above study. The simulations also found antenna radiation efficiency decreased by up to −40% when a bee appears in the near-field (the bee is absorbing RF). RF transmission gain patterns were also found to depend on the separation distance between the bee and the antenna, with a stronger higher frequency dependence.

Jeladze et al. (61) modeled honey bee exposures to RF between 2.5 and 100 GHz using numerical simulations based on the finite-difference time-domain (FDTD) technique with the goal of creating predictive SAR estimates in honey bee tissues. Whole-body and brain tissue-averaged honey bee SAR values were determined for all considered frequencies and normalized on 1 mW/cm^2^ incident plane-wave power density; SAR values were also averaged over a volume of 1 milligram of tissue. They found, not surprisingly, that EMF absorption in insects depends on E-field polarization, frequency, and the insect’s body peculiarities.

In a follow-up study, Jeladze et al. (62) broadened their inquiry with several other adult insect models: ladybug (*Coccinellidae septempunctata*), worker honey bee, wasp (*Vespidae* spp.), and mantis (*Mantis* spp.) at frequencies between 2.5 and 100 GHz. The goal was the same: estimate SAR values in tissues to inform predictive potential via energy absorption values per insect species. Whole-body and tissue-averaged, and using 1 mg SAR values were determined in insects for all considered frequencies for 10 different incident plane waves. SAR values were normalized to 1 mW/cm^2^ incident power density. Maximal EMF absorption in brain tissues was determined at 6, 12, and 25 GHz for the most considered insects while ladybug brain tissue reached maximal absorption at 60 GHz. This was the first study to estimate SARs for multiple insects over a wide range of RF frequencies using 3-tissue heterogenous insect 3D models created specifically for this research. They validated tissues’ dielectric properties and found EMF insect energy absorption highly dependent on frequency, polarization, and insect morphology.

The above body of studies alone should trigger a halt to any broad deployment of RF technologies in higher frequency ranges >3 GHz without significant environmental review and may indicate the need to reengineer 5G and newer networks altogether into lower frequency ranges.

One glaring problem is that newest exposure regulations for humans by The International Commission on Non-ionizing Radiation Protection (63) that are widely adopted throughout Europe and elsewhere now allow for higher RF *exposures* in the 5G ranges and are expected to top heating thresholds even for humans. U.S. regulations created by The Institute of Electrical and Electronics Engineers (64) and adopted by the U.S. Federal Communications Commission (65), already allow higher exposures in 5G frequencies.

Higher allowances for humans may have widespread catastrophic environmental impacts from insect mortality alone, capable of punching holes in the entire food web. The human food supply is potentially endangered from just this one new technology and the above describes just a few insect species. For additional species, see Supplementary material 2.

## Part 2. Addressing radiation impacts to wildlife: policy ramifications

### Defining the problem

In the United States, the Federal Communications Commission (FCC), which has jurisdiction over RF exposure standards, has systematically allowed wireless infrastructure to be excluded from environmental review, delegating oversight to industry players while disregarding public and scientific input. People are sometimes confused by the term “standards” (which indicate enforceability), versus “guidelines” (which imply choice and voluntary adherence). Guidelines are what the professional panels of scientists at IEEE and ICNIRP recommend in their advisory roles to regulatory entities, but once guidelines are adopted by regulatory agencies, they become enforceable standards. The problem is getting agencies to enact that enforcement. The same is true of existing laws that could protect wildlife from EMFs if they were applied to environmental EMFs.

The current regulatory framework is insufficient to protect humans (66) and even less equipped to address nonhuman species. There are no acute or chronic EMF exposure guidelines or standards for wildlife in the U.S. by the FCC or IEEE, nor any licensing and/or regulatory rules and procedures. The same is true internationally regarding the recommended limits by ICNIRP adopted throughout Europe, Australia and other countries. There are other U.S. agencies besides the FCC with enforcement powers to address this, e.g., the U.S. Environmental Protection Agency (EPA) and the U.S. Fish and Wildlife Service (USFWS), but the EPA has been completely defunded for nonionizing radiation research, even as they maintain oversight for all environmental radiation effects, and the USFWS has no in-house expertise to investigate it.

Any suppositions that standards written for humans are sufficient to protect wildlife too completely miss the point of nonhuman species’ unique physiologies and extreme sensitivities far beyond that of humans.

Despite a growing call for action to address nonionizing radiation impacts, efforts to strengthen protections have been stalled by a politically and financially influential industry, coupled with a lack of political will and regulatory capture of U.S. Federal agencies (67)—all worsened by recent cuts to federal funding, staffing, and research capacity. A critical barrier to environmental protection lies in the fact that federal agencies have not formally recognized or evaluated the ecological effects of nonionizing radiation.

### Airspace as habitat

One of the first steps toward effective integration of EMF ecological effects into the larger regulatory picture is to understand that airspace is “habitat” for many species, like water and ground is for species we already protect against various pollutants and interferants. Many wildlife species, especially birds, bats, and insects, depend on airspace for migration, mating, foraging, nesting/roosting, and territorial defense. It is time to recognize nonionizing EMF as a biologically active form of air pollution (acknowledging it is also a form of ground and water pollution too), and develop rules at the pertinent regulatory agencies to designate ‘airspace as habitat’ so EMF can be regulated like other pollutants. Defining airspace as habitat would provide a legal foundation to assess cumulative EMF impacts as well as mitigate exposures. With that definition established, the framework within which anthropogenic EMF acts as a biological agent in the biosphere would then be clarified.

It must be pointed out that EMFs do not always act according to standard toxicology models. EMFs are on a continuum of physiological “process” effects, not unlike endocrine disruptors, rather than chemical toxin effects. EMFs are capable of both directly and indirectly impacting numerous biological processes, cell types, and organs at both high and low intensities. The variables depend on frequency, tissue type, species, size, orientation toward the radiating source, dose rate, exposure intensity, duration, and modulation, among others. The relationship is often nonlinear with the greatest effects occurring at lower intensities unlike classic linear dose–response toxin models. Such nonlinear dose–response relationships are common for many environmental toxins. [For a more in-depth discussion of airspace-as-habitat, see reference (48)].

### Existing laws that could protect wildlife

There are numerous effective laws in the U.S. and in other countries that could protect many species of wildlife if we apply them to EMF like other pollutants. The discussion below pertains to U.S. laws but similar protective laws exist throughout Europe, in Canada, and elsewhere and U.S. laws can be used as models for other countries to adopt. In addition, some U.S. laws are regional with cooperative agreements between Canada and Mexico too, especially for migratory species (Box 1).

BOX 1 **Existing U.S. laws/advisories that can apply to EMF environmental policy.**

• The National Environmental Policy Act (NEPA)

• NEPA Interagency Agreement with the U.S. Fish and Wildlife Service

• The Migratory Bird Treaty Act

• The Bald and Golden Eagle Protection Act

• The Fish and Wildlife Conservation Act

• The Endangered Species Act

• …other agency regulatory agreements

### National Environmental Policy Act

NEPA is a critical federal law designed to ensure that all major actions undertaken by federal agencies, including the approval of communications towers and wireless infrastructure, are evaluated for their environmental, ecological, and cumulative impacts. Enacted in 1970 under the Nixon Administration, it is an iconic environmental law constantly under attack by industries of all kinds (There are again current attempts to get it thrown out altogether). Under NEPA, federal agencies must assess any action with foreseeable environmental effects. However, the Federal Communications Commission has failed to comply with NEPA in numerous ways.

Erica Rosenberg, an environmental/public lands policy attorney with over 30 years’ experience in U.S. Federal agencies, Congress, and academia, worked at FCC’s Wireless Telecommunications Bureau (2014–2021) as a NEPA specialist. In 2022, Rosenberg wrote a definitive report on FCC environmental procedures and NEPA (68) in which she details how FCC systematically fails to comply by improperly categorizing major federal actions, like wireless infrastructure deployment, as exempt from review, thereby delegating environmental assessments to industry with no agency oversight; suppressing public involvement; ignoring cumulative and aesthetic impacts; and treating community concerns as legal complaints rather than legitimate input.

Rosenberg’s paper is a roadmap for how to incorporate EMF into NEPA reviews in which she states:

- “Effects” need to be regulated as a part of National Environmental Policy Act (NEPA; 42 U.S.C. 4,321 et seq.) by a thorough, meaningful, and scientifically valid site review of all communications towers, structures, satellites, and related platforms whose transmitting and receiving frequencies are authorized and permitted by the FCC.
- “Effects” under NEPA are defined (40 CFR 1,508.1) as, “changes to the human environment from a proposed action or alternatives that are reasonably foreseeable,” including direct and indirect impacts, and cumulative effects, and can encompass environmental, social, and economic changes that may occur as a result of the action, whether happening at the same time and place or later in time and further removed in distance from the impact (69). The terms “effects” and “impacts” are used interchangeably in NEPA analysis.
- “Direct effects” are impacts that occur at the time and place of a proposed action (e.g., the release of EMF from a communication tower killing or injuring migratory birds at specific times and durations at a specified tower).
- “Indirect effects” are impacts occurring later in time (e.g., impacts from EMF affecting the breeding and nesting behavior of migratory birds over time).
- “Cumulative effects” include the combined impacts of a proposed action when added to other past, present, and reasonably foreseeable future actions affecting the same area or wildlife species. These, for example, could collectively include the impacts to resident, breeding, feeding, transient, and migrating birds from cell tower radiation in the immediate area over time; ambient EMF in the immediate area and from nearby communication towers; habitat clearing for a tower; construction and maintenance site disturbance; herbicide use to maintain vegetation around towers; and other radiation impacts including from colocation of other antennas on a specified tower adding to the levels of EMF released.

### Major impediments to NEPA

A major complicating factor for NEPA enforcement is the U.S. Environmental Protection Agency (EPA), which should be responsible for determining what constitutes an “effect,” but has failed to define EMFs as having any such effects. EPA research into EMF bioeffects was halted in the mid-1990s and has since suffered from further staffing cuts. There is no dedicated EMF research effort in any agency for humans or wildlife. Without formal recognition of EMF effects, the NEPA process cannot function as intended, leaving wildlife unprotected.

Equally important, FCC’s procedures now allow most cell towers and wireless infrastructure to be “Categorically Excluded” (Cat-Exed) under the FCC’s NEPA review process (70). Examples include FCC’s approval/fast tracking of the 5G network (71) which includes hundreds of thousands of new small cell transmitters mounted on utility poles, plus the agency’s approval of the use of higher frequency millimeter-waves (72) for 5G without any environmental or wildlife assessment review.

Also categorically excluded from NEPA reviews or environmental assessment are all satellite systems, despite the U.S. Government Accountability Office concluding a multitude of possible environmental impacts, including increased space debris, atmospheric pollution, and interference with astronomical observations (73). The FCC continues to approve new iterations of Elon Musk’s StarLink communications system, with no radiation impact assessments to humans, wildlife or their habitats from satellites transmitting in broad radiation patterns (like a flashlight beam) to and from ground-based and low-earth orbit infrastructure respectively, contributing significant 5G radiation to every corner on Earth for the first time at such a significant scale.

### NEPA inter-agency cooperation

In February 2014, the U.S. Fish and Wildlife Service’s Division of Migratory Bird Management played a key role in prompting the U.S. Department of the Interior to formally request that the National Telecommunications and Information Administration (NTIA), part of the Department of Commerce, conduct a cumulative radiation impact study as part of a NEPA Environmental Impact Statement (EIS) review of their emergency broadcast First Responder Network (FirstNet).

The request included a letter and technical attachment (74) co-authored by one of this paper’s authors (AMM) and was aimed to evaluate radiation exposure and infrastructure impacts to migratory birds such as tower collision mortalities. The letter notes studies in Europe have documented nest and site abandonment, plumage deterioration, locomotion problems, reduced survivorship, and death.

The groundbreaking proposal in interagency collaboration, which saw an Environmental Impact Statement (EIS) initiated under the Obama administration (2014), was ultimately terminated during the first Trump administration. But since it set a precedent for establishing studies between federal agencies, it can hopefully be incorporated again in the future.

### U.S Fish and Wildlife Service

#### Migratory Bird Treaty Act

U.S. Fish and Wildlife Service (USFWS) plays several important roles by implementing steps to “avoid or minimize bird take.” “Take” under the Migratory Bird Treaty Act (MBTA; 16 U.S.C. 703 et. seq.) and its implementing regulations is defined as the un-permitted death, injury or crippling loss of migratory birds. USFWS does not normally issue permits for “incidental or accidental take” of migratory birds but when a *protected* migratory bird (under endangered or species- of-special-concern status) is involved, MBTA currently provides virtually no provision for “accidental or incidental take,” e.g., causing injury or death, even when otherwise normal, legal business practices or personal activities are involved (70). The U.S. Congress noted the “take” of even one protected migratory bird to be a violation of the statute, with fines and criminal penalties that can be extensive (A federal permit is also required to possess a migratory bird and/or its parts). Where “take” can be avoided or minimized by the implementation of published/scientifically valid conservation measures (e.g., the USFWS’s 2021 voluntary communication tower guidelines) (75), bird “take” and other negative impacts to the avian community can be reduced or even avoided. This applies to EMF exposure too. Injury, crippling loss, and death from nonionizing radiation can be considered “take” under the MBTA.

#### Bald and Golden Eagle Protection Act

Bald Eagles (*Haliaeetus leucocephalus*) and Golden Eagles (*Aquila chrysaetos*) are protected by the Bald and Golden Eagle Protection Act (BGEPA; 50 C.F.R. 22.3, 22.26 and 22.27). “Take” under BGEPA is more expansive than under MBTA (70) and includes the pursuit, shooting, poisoning, capturing, killing, trapping, collecting, molesting, and disturbing both species (50 C.F.R. 22.3). Permits are required for “disturbance take” (e.g., construction near an active Bald Eagle nest) and “take resulting in mortality” where an activity may result in eagle deaths (50 C.F.R. 22.26; e.g., collisions with Bald Eagle fledglings near an airport), as well as for “take of nests,” for example where nests need to be removed when too close to an active airport approach/departure runway (50 C.F.R. 22.27).

As cell towers and wireless antennas are increasingly sited near eagle habitats and nesting areas, there are real concerns regarding the “take” of eagles from wireless infrastructure exposure. However, the U.S. Fish and Wildlife Service, which enforces BGEPA, has yet to address this. There is no clear guidance or permitting protocols to monitor, assess or mitigate EMF-related impacts. But with proper refunding at EPA, new staffing at USFWS, and inter-agency cooperation, there could be.

#### Fish and Wildlife Conservation Act

The Fish and Wildlife Conservation Act authorizes/guides the U.S. Fish and Wildlife Service to monitor, assess, and promote the conservation of non-game (i.e., nonhunted) birds, especially declining species, to prevent the need for federal listing and to maintain bird populations at stable or increasing numbers. This is a daunting challenge due to the direct and indirect effects of the many structural issues impacting migratory birds—e.g., collisions with towers, wind turbines, power lines, bridges, and building glass; electrocutions at power lines; impacts from climate change, habitat loss/degradation, pesticides, predation by domestic cats—and now from rising ambient levels of EMFs too (48). USFWS also tracks the growing numbers of Birds of Conservation Concern (BCCs), e.g., species in decline but not yet ready for Federal listing as threatened or endangered (50). Currently, there are some 273 species and subspecies on the national BCC, Service Regional BCC, and Bird Conservation Region BCC lists, providing an early warning of likely peril unless the population trends are reversed. These BCC lists require periodic reviews and updates under provisions of the Fish and Wildlife Conservation Act (16 U.S.C. 2,901–2,912).

Nonionizing EMFs should be systematically considered in the USFWS’s conservation efforts since its impacts on bird populations add to the cumulative threats already identified, plus it aligns with the agency’s responsibility under the Fish and Wildlife Conservation Act.

#### Endangered Species Act

Federally listed bird species, whose populations (based on systematic reviews) are declining (some precipitously), are those designated and protected under the Endangered Species Act (ESA; 7 U.S.C. 136, 16 U.S.C. 1,531 et seq.). Current species include some 78 endangered and 15 threatened bird species on the List of Threatened and Endangered Species. An “endangered species” faces a significant risk of extinction in the near, foreseeable future throughout all or a significant portion of its range. A “threatened species” is at risk of becoming endangered in the near future. Collectively, BCC and ESA-listed birds represent at least 366 bird species (36% of the total U.S. bird population) in decline (some seriously), with numbers of both listed and BCC species growing (76). Additionally, the USFWS is also tasked to maintain stable or increasing breeding populations of Bald and Golden Eagles under regulations of BGEPA through compliance with NEPA.

Additionally, habitats are often despoiled by tower construction/operation, resulting in degradation, as well as bird and bat attraction/collision with towers, guy-wires, and lights. This is in addition to habitat fragmentation, noise disruptions, and other impacts (70). Studies have shown that cell tower radiation can couple with avian brain/eye tissue and act as an attractant to migratory birds, while also disrupting and disorienting them (14).

#### Next steps: changes needed at key federal agencies

All must recognize and address wildlife impacts under whatever statutory authority is within their purview. The two major agencies discussed below would have the most immediate impact.

BOX 2 **Federal agencies with a stake in recognizing airspace-as habitat and EMF effects to wildlife include but are not limited to:**

• The U.S. Environmental Protection Agency (EPA)

• U.S. Fish and Wildlife Service (USFWS)

• The Bureau of Land Management (BLM)

• U.S. Geological Survey (USGS)

• U.S. Forest Service (USFS)

• The Army Corps of Engineers (ACORE)

• The Federal Communications Commission

### U.S. Environmental Protection Agency

#### Background

The U.S. Environmental Protection Agency [EPA, with a dedicated team of researchers (77)], was once the lead federal agency responsible for researching the health effects of nonionizing electromagnetic fields backed by legal authority concerning radiation protection under the Public Health Services Act, Atomic Energy Act, and National Environmental Policy Act (NEPA) (78). However, EMF bioeffects research was defunded in the mid-1990s and the agency no longer conducts EMF research even though all radiation fields, including the nonionizing bands, remain under its environmental purview.

As noted in a 1978 GAO report, the EPA’s responsibility for eliminating/reducing harmful EMF effects had initiated the agency’s research effort because there was insufficient data upon which to base a safety standard (79). In 1994, a GAO report (80) updated Congress that the EPA was still actively evaluating the biological effects of radiofrequency radiation and was in the process of developing exposure guidelines, which the agency later indicated were nearing completion and expected to be released in 1995 (81). However, amidst the heavy industry lobbying around the 1996 Telecommunications Act of 1996 (82) that would permanently change the wireless landscape forever, there was a provision giving exposure standards-setting and enforcement to the FCC for the first time (Since 1968, FCC’s guidelines had been voluntary and states could create their own too). Near that time, Congress also defunded EPA’s research efforts, effectively halting its work on developing science-based safety limits. FCC, which is a licensing and engineering entity that relies on outside health/environmental expertise to help formulate policy, lost its major independent government research/advisory partner on RF exposures just as wireless technologies were about to take off.

Today, the EPA has formally acknowledged that it “does not have a *funded* mandate” to evaluate wireless bioeffects. When asked in 2020 (83), and again in 2023 (84), about potential RF impacts on birds, bees, and trees, the agency stated it was “not aware of any EPA reviews that have been conducted on this topic.”

Federal RF bioeffects research has all but ceased. There is no federal entity with expertise to evaluate the biological effects with implications to wildlife and the environment. The sole remaining federally funded initiative was in the 2000 National Toxicology Program’s (NTP) $30 million animal study, which found “clear evidence” that RF exposure causes cancer and DNA damage in animals. Ironically, the U.S. Food and Drug Administration FDA, which originally nominated the NTP study in 1999 due to concern over long-term health effects in humans, later dismissed NTP’s findings, arguing that animal studies were not applicable to human health (85) (Reverse logic would then imply the findings are more directly applicable to animals, such as small mammals nesting in trees near cell antennas and airborne species).

Despite its statutory authority, EPA is failing to protect the environment from EMF’s increasingly pervasive ambient pollution. The agency should be refunded for this particular area.

### Federal Communications Commission

The FCC is the only U.S. federal agency with an active role in ambient EMF control from infrastructure and some devices like cell phones which are also controlled by the U.S. Food and Drug Administration (Antennas and devices transmitting at or under 1,000 watts of effective radiated power are categorically exempt from FCC licensing requirements but documenting compliance is up to manufacturers).

FCC procedures to check for standards *compliance* regarding ambient RF levels are also designed exclusively around humans and fail to consider wildlife exposure *conditions*, e.g., compliance measurements near cell towers involve testing RF at ground level or other areas accessible to people, neglecting exposure in zones where birds, bats, insects, and tree canopies inhabit, including upper tree canopies and airspace at the height of the tower in a direct lateral exposure. As a result, towers can be deemed compliant even when they emit RF levels that exceed federal limits by thousands of times in near-field zones around transmitting antennas where wildlife exists. Further, in the U.S. there is no national measurement, oversight, or enforcement program/strategy in place to monitor levels near humans, much less in wildlife and ecologically sensitive areas. This void represents a significant omission in the U.S. regulatory framework.

As of this writing, there is one pending federal court mandate against the FCC to respond to research on wildlife impacts that could alter this. In 2021, the U.S. Court of Appeals, DC Circuit, ruled in *Environmental Health Trust et al. v. FCC* (86) that the FCC’s refusal to update its 1996 limits was “arbitrary and capricious,” and in violation of the Administrative Procedures Act because the FCC did not show adequate review of the submitted evidence related to numerous issues such as long-term health effects and children’s vulnerability. Further, the Court stated the FCC had “completely failed” to address the “substantive evidence of potential environmental harms” on the record, which included dozens of studies showing serious impacts to birds, bees, trees, and plants, some of which was previously provided by the USFWS’s Migratory Bird Division to the FCC (74). As of this writing, the FCC has not responded to the Court’s mandate, despite the submission to the FCC of numerous research studies published since the ruling.

No government worldwide has established science-based RF standards reflecting a threshold below which no adverse effects occur in animals and plants. This is a critical regulatory gap, given the low-intensity effects literature that now exist and is pertinent to ambient exposures (see Supplementary material 1). Exposures are expected to exceed even thermal thresholds throughout Europe and elsewhere that adopted ICNIRP standards (see Section ‘5G is Different’ above). We are headed in the wrong direction.

### Recommendations to protect wildlife

It is essential that science-based non-ionizing electromagnetic field exposure limits be developed specifically to protect wildlife, including continuous, chronic, low-intensity exposures. Meaningful RF limits will require large-scale, long-term research initiatives that include: laboratory experiments, field-based ecological studies, cross-species assessments for varying sensitivities among taxa, engineering studies that characterize real-world exposure scenarios, and species-specific dosimetry.

On a more granular level, it’s critical to broaden the database that has begun matching RF resonant absorption in insects >3 GHz to include other species across the nonionizing frequency bands, with at least representative species per taxa computer modeled for resonance relationships. Many species experience significant EMF resonance effects depending on body size, water content, and morphology (62). Modeling resonance relationships via computational simulations with laboratory validation can identify at-risk species to various frequencies, including species-specific absorption peaks and exposure thresholds to better predict impacts across taxa. Because of the complexity of biological responses (often nonlinear and species-specific), this will require computational, engineering, biology, and federal/state expertise.

### Different exposure guidelines for wildlife

There may be circumstances in which specific ELF/RF-EMF exposures need to be mitigated, e.g., during mating or migration periods when endangered or species-of-special-concern are known to inhabit particular habitats for shorter durations than year-round species. For instance, shallow springtime vernal pools that dry up during the rest of the year are the only breeding grounds for some endangered amphibians and salamanders in certain regions and it is known that both species are sensitive to anthropogenic ELF ground currents as well as RF (87, 88). Many avian and insect species also have a short-term seasonal presence in specific locations.

Anthropogenic ELF/RF-EMF is extraordinarily different in kind, field strength, duration, frequency, and signaling characteristics than anything that exists in nature. EMF can, and does, interrupt normal migration, mating, and food finding functions in nonhuman species, including in all environments (ground, air, aquatic) and in all taxa from microbiota to mammals (9, 38–43, 47, 49, 89, 90).

Any attempt to create exposure guidelines for flora and fauna needs to include the lowest intensity effects in both EMF static and anthropogenic exposures because that is where nonhuman species have evolved their unique physiological electromagnetic receptors. To exclude that literature is to miss essential human and nonhuman physiological difference. This is not an area where we can ignore fundamental differences in order to justify existing exposure standards for humans or conjure that such standards are sufficient for nonhuman protection too. That simply will never be the case.

Unfortunately, there is currently a new working group under ICNIRP’s umbrella (91) whose mission is to do just that and there are a handful of studies pointing to the direction they will go and the methodology to be used. Several of the people in the working group are ICNRP members, including current and former ICNIRP chairs (91), and were instrumental in forming the criteria for a recent series of systematic reviews for the World Health Organization regarding human exposures which have come under heavy criticism for conflicts of interest, flawed methodology and failure to include critical information in their meta-analyses via their intentional exclusion criteria (92–96).

One example of a flawed research approach pertinent to wildlife that parallels the same flaws in the WHO systematic reviews is the proposal by Karipidis et al. (97) for a mapping protocol in which the authors described their criteria for review of existing evidence of anthropogenic RF-EMF effects on flora and fauna. Notably excluded was the most salient research at the lowest intensities at or near natural background levels where many nonhuman species have finely tuned electromagnetic sensory receptors. The protocol’s elimination criterion defines study controls as: “Sham exposure, no exposure beyond the background exposure level (which can be assumed to be negligibly low), or exposure at a lower level” (97).” Therefore the lowest most biologically active exposures to wildlife were likely mixed in with controls and vanish into the analysis as a non-exposure, if they were included at all. The final publication from that proposal was Brzozek et al. (98) and it was inevitably skewed toward a biased outcome (The final paper interestingly left out the statements on low-level exclusions). Karipidis is the current director of ICNIRP, the first author of the proposal, and a co-author of the Brzozek et al. systematic review (98) with the stated purpose to see if current exposure standards for humans can also be considered protective of wildlife and plants. Their conclusion is that the research reviewed “…raises doubts on whether animals and plants are truly affected at levels below human exposure limits.” Their exclusion criteria are also similar to the WHO systematic reviews methodology: 24,000 papers screened for inclusion but only 334 were considered eligible (277 fauna, 97 flora). Thus, like the flawed WHO systematic reviews that Karipidis played a leading role in conducting, ICNIRP’s standards could be justified as applying to nonhuman species too. But that is fundamentally an insupportable conclusion when the most pertinent research is factored in.

The recent effort with conflicts of interest is by Prokscha et al. (99) which was heavily funded by industry associated/pro-technology development entities in Europe (See their paper’s Acknowledgements and Funding). This study contains impressive modeling of simulated honey bee whole body and individual organ/body parts under THz frequency exposures which are significantly higher frequencies than similar studies conducted by some of the same authors (59–62). The study involved detailed material characterization of honey bee anatomy using resin-manufactured model bees to enhance computational accuracy in the THz range. RF-EMF exposure simulations estimated at 300 GHz revealed that at very short distances (1 mm) from the antenna, the authors said that bees: “…can absorb up to 26% of the input power into an antenna. In the near-field, the majority of the power is absorbed in the bee’s exoskeleton. However, in the far-field, the inner tissues absorb a slightly higher relative fraction of the absorbed power. Observing compliance with the ICNIRP reference levels in the THz application, the maximally allowed far-field exposure of 10 W/m^2^ corresponds to an average absorbed power of 0.29 mW into a honey bee worker.” These absorption values are well within the range of many low-intensity effects studies in Supplementary material 1. Honey bees reach resonance in frequency ranges below the THz bands but this study shows that a quarter of the transmitted THz power is absorbed by the bee.

What is most problematic about this study however, is the fact that the purpose was to create THz technology to *monitor* bees and other small insects in the wild and in apiculture. It was intended to facilitate the development of new technologies using additional introduced frequencies in environmental settings to which insects are highly sensitive and reactive in order to monitor how they are doing in their natural environments. The paper envisions selling such equipment to bee keepers. There is a serious disconnect here, and potential damage to the very animals they are trying to observe.

It is possible that wildlife species could be afforded some level of protection if the recommendations as discussed in the “Science-Based Policy” section of “Recommendations to Protect Wildlife” below regarding allowable exposures are adopted. The values are based on the median of the low-level effects literature in Supplementary material 1 with a 10-fold safety protection factor built in as a reference level.

This is a highly inexact method of wildlife protection but is a beginning approach. The preferred model would be to systematically delineate specific resonance factors in representative sizes per species families that expands upon the approach of Thielens (53); Thielens et al. (59); Herssens et al. (100); Toribio and Thielens (101); Velghe et al., (102); Jeladze et al. (61, 62) beyond insects. It is possible to create an exposure model for all taxa with some clarity, but it will take time/research funding by appropriate independent investigators separate from industry influence as is unfortunately the current situation with the ICNIRP special working group that closely mirrors the mistakes of the former WHO papers mentioned. Their approach is methodologically flawed from the start.

### Agency reform

#### Ethical model for environmental radiation protection already exists

There is a risk management approach for environmental radiation protection for ionizing radiation (IR), i.e., high intensity exposures, lasting significantly longer in the environment, capable of damaging DNA directly as well as causing a number of long-term devastating multi-generational effects to humans and nonhumans alike. Such ionizing effects are well established. No such risk management approach exists for nonionizing radiation (NIR). The physical mechanisms of action between ionizing and nonionizing radiation are different in some ways and similar in others. For instance, they break molecular bonding differently. IR has enough energy (>12 eV) to break certain atomic bonds directly whereas NIR breaks bonds via indirect mechanisms such as the creation of reactive oxygen species (ROS) leading to oxidative stress. In many ways, cellular reactions to NIR are more subtle than with IR via complex cellular stress responses at various exposure intensities, frequencies, and modulations that can affect multiple cell types with different long-term reactions (103). What NIR and IR have in common is that long-term biological damage is caused by excessive production of ROS and the inability of biology to repair damage other that via cell death which is a living organism’s natural self-defense in order to preserve the entire organism. But when cells are sufficiently damaged beyond repair yet do not die, cells can continue to reproduce in a damaged state, capable of passing on mutant conditions.

IR and NIR effects are different enough that there are good reasons to regulate them separately, but some of the definitional divide is also based on professional “cultural territoriality” too.

The International Commission on Radiation Protection [ICRP; (104)] is a respected independent nongovernmental organization that provides government entities with recommendations/guidance associated with ionizing radiation from artificial sources used in medicine, industry, nuclear enterprises, and naturally occurring sources. It was founded in 1928 by the International Congress of Radiology to advance science and radiological protection for public benefit.

In recent years, ICRP has also created a framework for wildlife protection via risk management that could be useful in regulating nonionizing radiation as well. Their approach is based on ethical imperatives as first principles to prevent/reduce the frequency of deleterious environmental radiation effects to a level of negligible impacts on biological diversity maintenance, as well as species conservation and the health/status of natural habitats, communities, and ecosystems. ICRP also recognizes the need for national authorities to demonstrate such protection within legislative frameworks.

ICRP’s focus is commensurate with overall level of risk and compatible with other environmental protections. They further acknowledge that there are necessary numerical guidelines regarding exposure/dose, dose/effect, and effect/consequence relationships. Their goal is to create for the environment “a sound scientific system” similar to that for human protection via a set of reference animals and plants. ICNIRP Publication 108 (104) defines a small set of reference species and relevant databases, discusses pathways of exposure, and collates/discusses best-available dosimetry data at different stages of life cycles. The publication further developed data sets for ionizing radiation measurements that may be within, or external to, each organism.

Their intention is that such information be used by national entities to develop more applied/specific numerical approaches to the assessment and management of risks to non-human species as national needs and situations arise. It is not intended to set regulatory standards per se, but rather as a practical tool for high-level guidance to help regulators/technology operators demonstrate compliance with existing legislation and to provide the basis for the creation of environmental standards by other entities.

The general outlines of ICRP’s recommendations can be adapted to nonionizing wildlife protection too but much definitional ground work needs to be laid first, e.g., the recognition of airspace-as habit/aeroecology and database creation of representative species effects.

Changes at key federal agencies are important. Below are recommendations (see Supplementary material 3 for a consolidated table).

#### Environmental Protection Agency

- Define airspace as habitat: The Environmental Protection Agency (EPA), and all relevant agencies, should recognize nonionizing EMF as a biologically active form of pollution to air, ground and water. Airpsace should be designated as “habitat” so EMF can be regulated like other pollutants. Many wildlife species, especially birds, bats, and insects, depend on airspace for migration, mating, foraging, and territorial defense. Defining airspace as habitat provides a legal foundation to assess cumulative EMF impacts as well as mitigate exposures.
- EPA should launch a dedicated research program to evaluate the biological and ecological impacts of radiofrequency (RF) radiation, treating it as an environmental pollutant akin to lead and pesticides.
- This program should include hazard identification, long-term exposure studies, cumulative risk assessments, and synergies with other environmental stressors with a focus on both human health as well as wildlife.
- Determine what ICRP flora/fauna protection for ionizing radiation can/does apply to nonionizing protection; institute regulations according to ethical and biological constraints.
- The EPA should ensure that rigorous independent science, separate from industry influence, guides the development of science-based, federally developed safety standards that protect the public and the environment.
- EPA should maintain at least one bioelectromagnetics scientist to guide any programs.

#### U.S. Fish and Wildlife Service

- Re-initiate the inter-agency USFWS/NTIA EIS collaboration that provides the framework/preliminary study design for a radiation research bird study, which has already been developed.
- Acknowledge/address EMF effects to migratory birds, federally listed fish and wildlife, and other protected plants and animals. Exposure standards for wildlife need to include guidelines for “avoiding or minimizing take” of birds/listed species to include exposure standards for wildlife from chronic, low-level exposures to EMF.
- In December 2021, the USFWS updated a migratory bird “take” rule which overturned a Trump administration rule (M-37050) that had allowed incidental take. Amend the 2021 “take” rule to include known/suspected impacts from EMF radiation on migratory birds.

#### Federal Communications Commission

- The FCC should respond to the Court mandate in *EHT et al. v. FCC* by requesting relevant expert agencies evaluate current scientific evidence on flora and fauna.
- FCC should request research in relevant agencies to address data gaps to address updating regulations to include wildlife protections for chronic, low-level exposures.
- The FCC should include thorough assessment of wireless technology effects in tower permitting processes and incorporate changes into their rulemaking regarding “effects of communication towers on migratory birds.”
- The FCC should work with USFWS to help private landowners develop ESA Section 10 Habitat Conservation Plans, especially in “critical habitat” where recovery goals for listed species have been designated and where impacts from cell towers and other radiation sources are at issue.
- The FCC should establish a measuring/monitoring program for transparency and environmental oversight to include a centralized, publicly accessible registry of all existing/proposed cell towers, 4G, 5G, and base station wireless facilities.
- The FCC should implement a nationwide EMF monitoring program that prioritizes ecologically sensitive areas where wildlife are vulnerable, e.g., national/state parks, forests, wildlife refuges, wetlands, coastal ecosystems, migratory corridors, and designated wilderness areas, along with human populations in other areas.
- The monitoring program should collaborate with environmental agencies to include environmental surveillance to track potential effects on wildlife, plants and trees. Monitoring species behavior, reproduction, and population trends in high-exposure areas would help identify risks and inform responsible infrastructure deployment, just as public health systems track human outcomes.
- FCC needs to bring its environmental review process in line with NEPA and restore public accountability. This includes developing NEPA-compliant procedures to assess cumulative environmental impacts on wildlife, insects, and flora.
- The agency should require documentation of categorically excluded (CE) facilities, such as small cells, and make environmental assessments, including RF compliance reports, publicly accessible.
- Regular, independent spot checks, particularly of RF levels pre- and post-construction, should be mandated for base station wireless facilities (cell towers, 4G and 5G).
- FCC should no longer allow industry advocates to self-determine the extent or need for environmental review. Further, public notice and comment periods must be meaningful, transparent and genuinely considered in the decision-making.
- FCC should implement NEPA reviews for all satellite systems, present and proposed.
- FCC should request congressional funding for at least one wildlife biologist on staff.

#### General

- **Pre-market testing:** All relevant agencies should premarket test technologies within their purview before deployment to include a cost–benefit/cumulative effects analysis regarding their impacts.
- **Pre-deployment environmental consultation and assessment:** Federal agencies should conduct full environmental reviews prior to licensing and national buildout of major new technologies like 5G, 6G and beyond.
- **Post-market environmental surveillance reporting systems:** Biologists, local governments and the public should have accessible transparent mechanisms to report ecological impacts. Such a system would support timely investigations; help identify patterns and inform mitigation measures.
- **Submit mitigation recommendations:** The relevant federal/state/local agencies related to natural resources should be consulted on proposed networks and deployment of wireless facilities and submit recommendations on protecting sensitive species.
- **Interagency coordination:** Agencies should develop internal expertise in bioelectromagnetics, establish formal interagency coordination, convene regular meetings to include the FCC, EPA, USFWS, NTIA, and others. Collaboratively assess/manage EMF/RFR effects through joint research, analysis and advisement to policymakers.

#### Science-based policy

Due to the many variables involved with biological effects—frequency, signal strength, duration, pulsing, modulation, resonance effects, and fluctuating wildlife presence, it is indeed a challenge to designate “safe” levels of radiation exposure in the environment, but a number of key steps can be taken to make facilities and habitats safer for wildlife. Compromises are understood as necessary but they must be science-based.

No one exposure limit can encompass protection for all flora and fauna given the variables, vast physical differences, and environments. What may protect one species may prove detrimental to another. It will be years before we have a general framework of frequency-dependent whole-body and-organ specific resonance factors per species. Thielens et al. (53); Toribio et al. (60); Jeladze et al. (61, 62) and Velghe et al. (102) have charted a computer modeled way forward for representative insect species that should be expanded to all taxa. There will always be unique absorption capacities per species and variations within those species. The best we can do at present is acknowledge the limitations of any broad approach and proceed accordingly over time to fill in the gaps.

One generalized method is to use the median of the low-intensity effects literature in Supplementary material 1 (see Box 2) to which most species are exposed today with an additional safety factor of 10 built in, knowing that this may not be enough to fully protect many avian, bat, and insect species capable of reaching very close proximities to infrastructure transmitters like small cells/towers. Until research is completed on resonance and other factors that clearly characterize risk to myriad species, we can only approximate exposure reduction based on data we do have for the most vulnerable species. It then becomes a question of what safety factor is most effective to potentially cover a broader range.

- **Exposures limits:** Set evidence-based RFR exposure limits and regulations for wildlife. As a starting point based on the median of the low-intensity studies in Supplementary material 1 with a 10-fold protection level, for wildlife this equals: SAR: 0.003 W/kg (3 mW/kg); Incident Power Density: ~0.005 mW/cm^2^ (5.0 μW/cm^2^). This lowers the ICNIRP and FCC exposure standards by a factor of more than 100.
- **Resonance modeling:** Evaluate species sensitivity and model whole-body and organ-specific resonance relationships per taxa; create a comprehensive database that is species- and frequency-specific, based on computer modeling matched with laboratory and/or field validation.
- **Update compliance procedures:** Tower/small cell compliance testing procedures must be updated to include RF exposure assessments at antenna height and in nearby wildlife habitats, not just at human-occupied ground levels.
- **Robust environmental evaluations:** Agencies need to conduct detailed environmental evaluations (e.g., EIS NEPA reviews that include mitigation) where new communication networks are proposed for wildlife/wilderness areas. Public consultation should be mandatory.
- **Environmental impact statements (EIS):** reviews should include proposed levels of electromagnetic radiation emissions as well as assessments of strategic design modifications to mitigate RF exposure including: directing antenna emissions away from critical wildlife areas like foraging, nesting, and denning sites; ELF mitigation could include designating appropriate locations for high-tension corridors and modifying existing wire configurations to reduce/cancel magnetic field intensity in sensitive habitats.
- **Satellite systems subject to NEPA:** All satellite systems, especially in low earth orbits like StarLink, must be subject to full NEPA reviews for effects to humans, wildlife, and atmospheric perturbations capable of adversely affecting habitat. Satellites have been categorically excluded from review from the start under the assumption that radiation reaching Earth was too low to matter. That is not accurate with the lower orbits used at today’s satellite deployment scale occurring from many countries. Some migratory bird species fly at heights that increase their exposures significantly, especially from 5G networks. We are unfortunately going in the wrong direction with FCC rulings and political influence that exempt satellites from all NEPA review (105, 106).
- **Targeted protections for wildlife and habitats:** Wildlife does not have the luxury of EMF avoidance that humans do. Shielding and physical avoidance barriers around towers and high tension corridors are options to deter terrestrial ungulates, carnivores and small mammals but are not effective at blocking birds, bats, and flying insects. Only RF reduction can do that. Since the intensity of natural RFR is low (i.e., low noise level), and wireless communications use specific frequencies and modulations, reducing the emission intensity should have little effect on signal-to-noise detection by a sensitive receiver species.
- **Setbacks in federal parkland and state-protected areas:** Enhanced, species-specific targeted protections for wildlife and habitats should include: Minimum of 1,500 feet (457 m) setbacks between antennas and sensitive wildlife habitats to minimize ecological disruption (107, 108). This is particularly critical for areas such as National Audubon Society Important Bird Areas, ESA-designated critical habitats, designated wilderness areas, and federally listed bat hibernacula, requiring regulatory action by the FCC.
- **Establish low- to no-EMF zones:** Low- and no-EMF areas can protect important wildlife habitats and ecologically sensitive areas, especially during migration and mating seasons. These include designated wilderness areas, critical habitats, national parks, preserves and monuments, national historic sites, national forests and wildlife refuges, and world heritage sites among others.
- **Remove/restrict existing infrastructure:** In particularly sensitive areas containing endangered species, removing or restricting new wireless development may be needed. 5G and 6G’s use of higher-frequency sub-millimeter/millimeter waves that pose heightened risks to sensitive taxa, especially insects, should be prohibited in ecologically sensitive areas. Insects are food supplies for everything else. This will require coordinated involvement from the FCC, USFWS, NPS, USFS, BLM, ACORE, and others will need to be key players in these efforts including state-level park and wildlife management agencies.
- **Barriers, radiation patterns, and others:** Inexpensive mitigation measures include: maximizing the distance between wildlife and EMF sources with barriers, re-design of transmission radial plots away from sensitive habitats, and powerline placement on pylons to reduce magnetic fields near corridors can all help mitigate effects to wildlife. Regarding avian protection, information for the electric utility and communications industries is available through the Avian Power Line Interaction Committee (APLIC) (109, 110).
- **Wildlife radio tracking:** Radio tracking gear attached to wildlife by researchers, wildlife professionals, animal trackers, and others use pulse modulated RF in communication with satellites and can cause adverse effects to animal health as well as interfering with magnetoreception, behavior, hunting and reproduction, and can cause tissue damage over time (111, 112). Wildlife should not be unnecessarily enlisted as inadvertent research subjects or for human curiosity/entertainment. Non-invasive tracking methods should be prioritized over telemetry when possible, safer technologies need to be designed, and telemetry tracking gear more carefully assessed by state/Federal agencies that use/permit such gear (e.g., NPS, USFWS, USFS, BLM, ACORE, and NOAA-Fisheries). Best management practices should be used, including configuring devices to minimize EMF emissions especially to the brain, keeping radio telemeters in sleep mode where feasible, reducing the amount of tracking gear and the number of target animals used, as well as lowering the transmission times/rates. Tagged animals should be monitored for health and behavioral effects (111). Pertinent agencies that use such gear, as well as the Federal Trade Commission, could provide updated consumer tips to scientists regarding telemeter use (113). State veterinary departments should be made aware of the potential impacts from radiation before issuing permits needed by wildlife researchers to conduct their tracking studies on State and private lands.
- **As low as reasonably achievable (ALARA):** The ALARA Principle should follow science-based thresholds (114), minimizing exposure levels wherever possible. In practical terms, this means that whenever a new tower or wireless facility is proposed, regulators, network engineers, and decision-makers must ask: “Is the radiation truly as low as it can be?” This question must guide every aspect of infrastructure planning, including placement, design, transmission power densities, frequencies used, time-of-use relative to wildlife presence, migration/reproduction timeframes and more. ALARA alone, however, is not enough since no formal definition of what ALARA means for wildlife currently exists. Without scientifically established exposure limits, industry players may claim they are already meeting ALARA, when they are in fact greenlighting harmful exposures. To truly protect ecosystems, we must develop robust, science-based limits grounded in a comprehensive understanding of biological impacts across species and habitats.
- **Technologies designed with wildlife in mind:** New technologies and communication networks need to be designed with wildlife protection as a priority. Supported by the scientific evidence, there is growing scientific momentum toward incorporating environmental considerations into the design and deployment of communication technologies (115).

## Discussion

Virtually all terrestrial plants and animals, and some marine organisms are inadvertently affected by exposures from anthropogenic ELF/RF-EMF, including at extremely low thresholds, barely above natural background levels. The evidence for impacts is incontrovertible (9, 47, 48). Yet despite the ever-mounting scientific evidence that EMF poses adverse biological risks to myriad flora and fauna, Federal agencies continue to ignore or minimize these impacts, leaving a critical gap in environmental protection.

When cell towers and wireless infrastructure are proposed, various permitting procedures review the U.S. Federal natural resource agencies that license and/or maintain a range of communication devices on the properties they manage, e.g., communication towers, emergency broadcast systems, and numerous other sources of point-to-point and Internet communications, but fail to address impacts to wildlife and their habitats.

As the current 6th major extinction epoch proceeds (116, 117) inaction is no longer a defensible response. The unchecked proliferation of electromagnetic fields is compounding the already severe threats facing wildlife. If we effectively reduce ambient EMF exposures, it might give imperiled species continuing chances to recuperate and reverse population declines.

Regulatory agencies must adopt science-based limits that take ambient low-intensity exposures into consideration, implement robust environmental assessments, and treat nonionizing EMF as a biologically active pollutant. The survival of countless species depends on how we respond today.

Any serious inquiry into EMF wildlife effects must begin from biological realities, not pre-existing dosimetry perspectives (49) as reflected in current standards for humans. Any attempts to claim that the standards for humans are adequate for nonhuman species (118) must be challenged as physiological impossibility. Rising ambient levels today may be leading to ecological crisis (9). New 5G and proposed next-generation networks are likely to target seminal species like insects upon which the entire food chain relies for survival.

A government-wide response is urgently needed, one that recognizes EMF as an air pollutant and treats airspace as habitat through the new discipline of air ecology (or aeroecology). Protecting this dimension of habitat is as critical as safeguarding land and water.

We must establish wildlife-specific radiation exposure guidelines, particularly for chronic, low-intensity exposures that current standards ignore. A robust, independent research program, free from industry influence, is essential and it needs to be developed, funded and implemented as soon as possible. Rigorously mitigating exposure to wildlife must become a standard practice. Existing field protocols, such as those used by the U.S. Forest Service can be adapted to monitor/ assess radiation effects in real-world settings. Many of the tools and knowledge already exist. We have field research protocols for cell towers, e.g., by the USFS that can be easily modified to include studies of radiation in the field (75).

Different exposure guidelines need to be created to protect wildlife and flora based on the low-intensity effects literature in Supplementary material 1 which is mostly comprised of animal studies. Truly protecting wildlife, however, is a daunting task that may include frequency re-allocation, redesign of hardware and network engineering, and society moving away from certain competitive economic models, as well as ELF/RF-EMF-free zones during migration and breeding seasons (48, 49).

Some of the suggested agency reforms will, of course, require significant new funding at specific agencies like EPA and USFWS for added staff and research facilities. But many suggestions can be accomplished through better testing/enforcement within existing budgets, simple cooperation between agencies, and better technology and infrastructure design funded by vested industries. What is needed is the political will/institutional commitment to act. This will require not just agency reform, but a coordinated effort involving policymakers, scientists, conservationists, and the public. A science-based, risk-mitigation approach will ensure that wildlife and ecosystems are protected in the digital age.

Given the complexity of ecosystems and the subtle cumulative effects of chronic low-intensity exposures, delay will only deepen the ecological harm.

## Author’s note

This paper is based on the combined presentations of four invited speakers at the Environmental Health Trust Symposium at the Yale School of Medicine, June 5, 2024, Session Title: “EMF Impacts to Flora and Fauna, Policy Development and Regulatory Gaps.”
