# Correction: Flora and fauna: how nonhuman species interact with natural and man-made EMF at ecosystem levels and public policy recommendations

**DOI:** 10.3389/fpubh.2026.1796521

**Published:** 2026-02-12

**Authors:** B. Blake Levitt, Henry C. Lai, Albert M. Manville, Theodora Scarato

**Affiliations:** 1 National Association of Science Writers, Berkeley, CA, United States; 2 Department of Bioengineering, University of Washington, Seattle, WA, United States; 3 Advanced Academic Program's Environmental Sciences and Policy Division, School of Arts and Sciences, Johns Hopkins University, Washington, DC, United States; 4 Environmental Health Sciences, Bozeman, MT, United States

**Keywords:** electromagnetic fields, radiofrequency radiation, wildlife, nonhuman species, low-intensity effects, airspace-as-habitat, aeroecology, National Environmental Policy Act

An incorrect word was used in the section **Part 1. Introduction: historical background***, Ecosystem perspective and low-intensity exposures*, paragraph 2.

The paragraph read:

“In its natural state, very little radiofrequency radiation (RFR) reaches the Earth's surface. Aside from the Earth's natural extremely low frequency (ELF) direct current (DC) static magnetic fields and the Schumann Resonances, lightening and sunlight would primarily comprise our ecological exposures to the electromagnetic spectrum.”

The corrected paragraph reads:

“In its natural state, very little radiofrequency radiation (RFR) reaches the Earth's surface. Aside from the Earth's natural extremely low frequency (ELF) direct current (DC) static magnetic fields and the Schumann Resonances, lightning and sunlight would primarily comprise our ecological exposures to the electromagnetic spectrum.”

An incorrect unit was used in the section **Part 2. Addressing radiation impacts to wildlife: policy ramifications**, *Science-based polic*y, paragraph 4.

The phrase read “SAR: 0.003 W/kg (3 μW/kg);”.

The corrected phrase reads “SAR: 0.003 W/kg (3 mW/kg);”

The original version of this article has been updated.

