# Supplementary material for: Flora and fauna: how nonhuman species interact with natural and man-made EMF at ecosystem levels and public policy recommendations
Source: Front Public Health. 2025 Nov 19;13:1693873. doi: 10.3389/fpubh.2025.1693873 (PMC12675998; doi:10.3389/fpubh.2025.1693873)
Supplement: Supplementary file 1 [file Supplementary_file_1.docx]

**November 3, 2025**

**Supplement Table 1**

**Biological Effects in Animals and Plants Exposed to Low-Intensity RFR**

**(SAR< 0.4 W/kg; Starred studies (*) are <0.001 W/kg)**

|  |  | **SAR (W/kg)** | **Effects reported** |
| --- | --- | --- | --- |
| Adey et al. (1982) | Cerebral cortex of cats immobilized under local anesthesia; exposed to 450 MHz RFR modulated at 16 Hz for 1 h | 0.29 | Increased calcium efflux |
| Aitken et al. (2005) | Mice exposed to 900 MHz RFR, 12 h/day; 7 days | 0.09 | Genotoxic effect in sperm |
| * Akdag et al. (2016) | Rats exposed to 2400 MHz RFR from a Wi-Fi signal generator for a year | 0.000141 (minimum)- 0.007127 (maximum) | DNA damage in testes |
| Alchalabi et al. (2016) | Offspring from pregnant ats exposed to 1800 MHz GSM signal; 1 or 2 h/day for 1, 2, or 3 weeks | 0.048 | Detrimental effects on pre-and postnatal development |
| Alkis et al. (2019a) | Rats exposed to 900; 1800; and 2100 MHz RFR; 2 h/day; 6 months | Brain SAR:  900 MHz -0.0845; 1800 MHz-0.04563; 210 MHz-0.03957 | DNA single strand break and oxidative damages in frontal lobe |
| Alkis et al. (2019b) | Rats exposed to 900; 1800; and 2100 MHz RFR; 2 h/day;6 months | Brain SAR:  900 MHz -0.000333; 1800 MHz-0.002647; 210 MHz-0.002815 | DNA strand beaks and oxidative DNA damage in testicular tissue |
| Amiri et al. (2023) | Liver of rats exposed to 2450 MHz RFR from a Wi-Fi router; 4 h/day for 4 weeks | 0.03 | Decreased superoxide dismutase and catalase CA, increased lipid peroxidation, attenuated by swimming |
| Arendash et al. (2010) | Normal and transgenic Mice exposed to 918 MHz RFR, 2 h/day for 2-8 months | 0.25 | Improved cognitive behavior, decreased amyloid-β deposition in brain |
| Atasoy et al. (2013) | Rats exposed to 2437 MHz (Wi-Fi) RFR; 24 h/day for 20 weeks | maximum 0.091 | Oxidative DNA damage in blood and testes |
| Arslan et al. (2024) | Rats exposed to 217-Hz modulated 1800 MHz RFR; 2 h/day for 8 weeks | 0.06 | p38MAPK gene expression up-regulated in brain |
| Aynali et al. (2013) | Laryngotracheal mucosa of rats exposed to 2450 MHz RFR; 1 h/day for 28 days | 0.143 | Increased lipid peroxidation; decreased glutathione peroxidase; attenuated by melatonin |
| Bakacak et al. (2015) | Female rats exposed to 900 MHz RFR 15 min/day for 15 days at the abdominal region | 0.018 - 4 | Decrease in the number of ovarian follicles |
| * Barteri et al. (2016) | Lactoperoxidase and horseradish peroxidase exposed to 900 MHz RFR modulated at 217 Hz, 30 min, | 0.00004 | RFR caused conformational changes of the active sites of the enzymes and influenced the formation and stability of the intermediate free radicals. |
| Bedir et al. (2018) | Rat exposed to 2100 MHz RFR, 6 or 19 h/day, 30 days | 0.024 | Oxidative process-mediated renal injury |
| Bektas et al. (2022) | Brain of normal and diabetic rats exposed to 3,500 MHz RFR, GSM-modulated; 2 h/day for 30 days | 0.323 | Increased total oxidant status and H_2_O_2_, decreased total antioxidant status. |
| Bektas et al. (2023) | Muscle of normal and diabetic rats exposed to 900 MHz, 1800 MHz, 2100 MHz RFR; 2 h/day for 1 month | 0.026, 0,164, 0.173 | Increased lipid peroxidation and ischemia-modified albumin; decreased catalase, and glutathione |
| Bektas et al. (2024) | Cardiac blood and fat tissue from normal and diabetic rats exposed to 3500 MHz RFR 2 h/day, 5 days/week for 1 month | 0.037 | Total oxidant status, total antioxidant status, and H_2_O_2_ level affected by RFR and diabetic condition (mostly increases); tissue-dependent effects observed. |
| Belyaev et al. (2005) | Human white blood cells exposed to 915 MHz GSM signal, 24 or 48 h | 0.037 | Genetic changes |
| Belyaev et al. (2009) | Human white blood cells exposed to 915 MHz, 1947 MHz; GSM, UMTS signals; 24 or 72 h | 0.037 | Affected DNA repair mechanism |
| Bilgici et al. (2018) | Rats exposed to 2450 MHz RFR 1 h/day for 30 days | 0.0233 | Increased inflammation and testicular damage |
| * Blackman et al. (1980) | Forebrain of chicken exposed to 50 MHz, AM at 16 Hz, 20 min | 0.0014 | Affected calcium efflux |
| * Blackman et al. (1989) | Forebrain of chicken exposed to 50 MHz, AM at 16 Hz, 20 min | 0.00036 | Affected calcium efflux |
| Bodera et al. (2015) | Blood, liver, kidney, and brain of healthy rats and rats with persistent inflammatory state exposed to 1800 MHz RFR, 15 min/day for 5 days | 0.028 | Increased lipid peroxidation in brain, blood, and kidney after RFR + tramadol. |
| Bodin et al. (2023) | Pregnant rats and offspring exposed to 3500 MHz continuous wave RFR for 22 h/day from gestational day 8 to post-natal day 21 | 0.07 | Disturbance in open field behavior and delayed incisor eruption |
| Bodin et al. (2025) | Mouse neuroepithelial stem cells exposed to GSM-900 MHz RFR for 3 or 7 days | 0.08 | Increased double strand DNA breaks and apoptosis |
| * Bourdineaud et al. (2017) | Earthworm (Eisenia fetida) exposed to 900 MHz RFR, 2 h | 0.00013-0.009 | DNA modification. increased catalase gene expression |
| Bozok et al. (2023) | Pregnant rats exposed to 900, 1800, or 2100 MHz RFR for 6, 12, or 24 h/day for 20 days | 0.087-0.17 | Myocardial tissue damage observed, increased lipid peroxidation, decreased antioxidation (dependent on frequency and exposure duration) |
| * Burlaka et al. (2013) | Embryos of Japanese quails exposed to GSM 900 MHz RFR; 38 h, 5 or 10 days | 0.000003 | Increased O_2_^.-^, nitric oxide, 8-hydroxydeoxyguanosine; decreased superoxide dismutase and catalase |
| Calis et al. (2021) | Pregnant rats exposed to 1800 MHz RFR, 1 h/day during whole pregnancy | 0.23 | Female offspring have lower mean number of primordial and secondary follicles, and a higher atresia score |
| * Cantu et al. (2023) | Human keratinocytes exposed to 900 MHz RFR for 1 h | 0.00155 | Six common DNA targets that were both differentially methylated and differentially expressed |
| Cao et al (2015) | Plasma from rats exposed to 1800 MHz RFR, 2 h/day for 32 days | 0.05653 | Circadian rhythms of melatonin, glutathione peroxidase and superoxide dismutase shifted. |
| Cappucci et al. (2022) | Head, ovary, and testis of Drosophila melanogaster exposed to 2437 MHz RFR (Wi-Fi) from embryo to adult stage | 0.0608 | Increased gene expression of hsp70, induced gene instability, and increased DNA damage, Increased reactive oxygen species |
| Capri et al. (2004) | Human lymphocytes exposed to 900 MHz, GSM signal, 1 h/day, 3 days | 0.07 | Cell proliferation and membrane chemistry |
| Čermak et al. (2020) | V79 cells exposed to 915-MHz GSM-modulated RFR for 1, 2, or 3 h. | 0.23, 0.8, or 1.6 | Microtubular structure impairment at 0.23 W/kg for 3 h |
| Cetin et al. (2014) | Pregnant rats and offspring exposed to 900 or 1800 MHz RFR, 1 h/day during pregnancy and postnatal period (to 6^th^ week) | 0.15 | Brain and liver glutathione peroxidase activities, as well as liver vitamin A and β-carotene concentrations decreased in the offspring, whereas brain iron, vitamin A, and β-carotene concentrations increased in the exposed groups. |
| Ceyhan et al. (2012) | Skin of rats exposed to pulsed 2450 MHz RFR; 1 h/day for 4 weeks | 0.064 | Decreased superoxide dismutase and glutathione peroxidase, increased catalase and lipid peroxidation. |
| Chandel et al. (2019a) | Onion roots (Allium cepa L.) exposed to 2350 MHz RFR for 1, 2, or 4 h | 0.313 | Increased mitotic index and chromosomal aberration; significant increase in DNA single strand breaks at 2 and 4 h. |
| Chandel et al. (2019b) | Onion roots (Allium cepa L.) exposed to 2100 MHz RFR for 1 or 4 h | 0.282 | Increased mitotic index, chromosomal aberration, and DNA single strand breaks after 4 h of exposure. |
| Chaturvedi et al. (2011) | Brain cells of rats exposed to 2450 MHz RFR, 2 h/day for 30 days | 0.03561 | Increased DNA strand breaks |
| Chauhan et al. (2017) | Liver, brain, spleen, kidney, and testis of rats exposed to 2450 MHz RFR, 2 h/day for 35 days | 0.14 | Increased lipid peroxidation in liver, brain, and spleen. |
| Chen et al. (2014) | Male mice exposed to 1800 MHz RFR 2 h/day for 32 days | 0.2221 | Reduced testicular sperm head count and serum testosterone, increased serum estradiol. circadian rhythms of testicular sperm head count and estradiol disappeared after exposure. |
| Comelekoglu et al. (2018) | Sciatic nerve of rats exposed to 1800 MHz RFR, 1 h/day, 4 weeks | 0.00421 | Changes in electrical activity, increased catalase, and degeneration of myelinated fibers |
| Coskun et al. (2024) | Parotid gland of rats exposed to 1800 MHz RFR, 6 or 12 h/day for 30 days | 0.117 | Increased total oxidant level, decreased total antioxidant status, increased apoptosis and histopathology |
| D’Andrea et al. (1986) | Rats exposed to 2450 MHz RFR 7 h/day.7 days/week for 90 days | 0.14 (0.11-0.18) | Affected shuttle box performance and schedule-controlled behavior. |
| Dasdag et al. (2015) | Rats exposed to 2450 MHz RFR 24 h/day for 1 year | 0.00242 | Increased Sperm head defect; decreased weight of the epididymis and seminal vesicles, seminiferous tubules diameter and tunica albuginea thickness |
| Delavarifar et al. (2020) | Male mice exposed to 2450 MHz RFR; 2 h/day for 4 days | 0.03- 0.092 | Increased sperm concentration |
| * [Demirbağ](https://pubmed.ncbi.nlm.nih.gov/?sort=date&term=Demirba%C4%9F+B&cauthor_id=37717340) et al. (2023) | Rats exposed to 1800 MHz RFR 1 h/day for 30 days | Maximum in body 0.0029; at testis: 0.001 | Ultrastructural changes seminiferous tubule and Leydig cells in the testis of RFR group; Increased lipid peroxidation, decreased catalase and superoxide dismutase |
| De Pomerai et al. (2003) | Protein exposed to 1-GHz RFR,  24 or 48 h | 0.015 | Protein damages |
| * Deshmukh et al. (2013a) | Rats exposed to 900, 1800, and 2450 MHz RFR; 2 h/day for 30 days | 0.0006-0.0007 | DNA strand breaks in brain |
| Deshmukh et al. (2013b) | Rats exposed to 900 MHz; 2 h/day for 30 days | 8.47 x 10^-5^ | Increased lipid peroxidation and protein oxidation in blood |
| * Deshmukh et al. (2015) | Rats exposed to 900, 1800, and 2450 MHz RFR; 2 h/day for180 days | 0.0006-0.0007 | Declined cognitive functions, increased brain HSP70 and DNA strand break |
| * Deshmukh et al. (2016) | Rats exposed 900, 1800, and 2450 MHz; 2 h/day for 90 days | 0.0006-0.0007 | Declined cognitive functions, increased brain HSP70 and DNA strand break in rats |
| D’Inzeo et al. (1988) | Cultured chick microtubes exposed to 10.75 GHz; 30-120 sec | 0.008 | Operation of acetylcholine-related ion-channels in cells |
| Djordjevic et al. (2015) | Liver of rats exposed to 900 MHz RFR from a mobile phone; 4 h/day for 20. 40, or 60 days | 0.089 | Depending on exposure duration, increased lipid peroxidation, protein oxidation, catalase, and 8-hydroxydeoxyguanosine |
| Durdik et al. (2019) | Human umbilical cord blood cells including CD34+ hematopoietic stem/progenitor cells exposed to GSM 900 RFR; 1 h | 0.004 -0.040 | Increased reactive oxygen species |
| Dutta et al. (1984) | Human neuroblastoma cells exposed to 915 MHz RFR, sinusoidal AM at 16 Hz, 30 min | 0.05 | Increase in calcium efflux |
| Dutta et al. (1989) | Human neuroblastoma cells, 147 MHz, AM at 16 Hz, 30 min | 0.005 | Increase in calcium efflux |
| Dutta et al. (1994) | Escherichia coli cultures containing a plasmid with a mammalian gene for enolase were exposed for 30 min to 147 MHz RFR AM at16 or 60 Hz | 0.05 | Enolase activity in exposed cultures RFR at AM at 16 Hz showed enhanced activity Enhanced, and AM at 60 Hz showed reduced activity (Modulation frequencies. 16 and 60 Hz, caused similar effects.) |
| * Eberhardt et al. (2008) | Rats exposed to 900 MHz GSM RFR for 2 h | 0.00012-0.12 | Changed blood-brain barrier permeability and nerve cell damage in the brain |
| Echchgadda et al. (2022) | Rat primary hippocampal neurons exposed to  3000 MHz RFR for 1 h | Average 0.3 | Decreased amplitude of action potential (AP) and resting membrane potential (MP), and increased neuronal excitability and synaptic transmission |
| Eker et al. (2018) | Rats exposed to 1800 MHz RFR, 2 h/day for 8 weeks | 0.06 | Increased caspase-3 and p38MAPK expressions in eye. |
| Elekes et al. (1996) | Mice exposed to 2450 MHz (continuous-wave or amplitude modulated at 50 Hz square wave) RFR, 3h/day for 6 days | 0.14 | Increased antibody producing cells in spleen of male mice. (No effect on female mice.) |
| Er et al. (2022) | Testis of rats exposed to 900 MHz RFR, 2 h/day, 5 day/week for 1 or 10 weeks | whole body 1.157, testis 0.107 | Increase in the RFR group in the acute period, whereas no difference in the chronic groups for the apoptotic indices (capase-3 and Bcl-xl); p-p38 and p-JNK protein expressions increased significantly in RFR groups in both periods. |
| Er et al, (2025) | Brain of rats exposed to 2100 MHz RFR, 2 h/day, 5 day/week for 1 or 10 weeks | 0.27 | Increased lipid peroxidation and protein carboxylation, decreased SOD and CAT only in 1-week exposure group. |
| Ertilav et al. (2018) | Hippocampal and dorsal root ganglion neurons of rats exposed to 900 or 1800 MHz RFR modulated at 217 Hz; 1 h/day (5 days/week) for 1 year | 0.15 | Increased reactive oxygen species (calcium involved) |
| Esmekaya et al. (2011) | Human peripheral blood lymphocytes exposed to 1800 MHz GSM- (217 HZ) modulated RFR for 6, 8, 24, or 48 h | 0.21 | Chromatin changes and increases in sister chromatin exchange. |
| Falcioni et al. (2018) | Rats exposed to 1800 MHz RFR from prenatal life to death at 19 h/day | 0.1, 0.03, or 0.001 | Increase in tumors in brain and heart |
| Forgacs et al. (2006) | Mice exposed to 1800 MHz RFR, GSM- 217 Hz pulses, 576 μs pulse width; 2 h/day, 10 days | 0.018 | Increase in serum testosterone |
| Furtado-Filho et al. (2014) | Pregnant rats exposed to 950 MHz RFR for 0.5 h/day for 51 days (21 days of gestation and 6-30 days old) | SAR of pregnant rat 0.01-0.03 W/kg; neonate 0.88 W/kg, 6-day old 0.51 W/kg, 15-day old 0.18 W/kg, 30-day old 0.06 W/kg | Decreased DNA strand breaks in liver of 15-day old and increased breaks in 30-day old offspring. |
| * Garaj-Vrhovac et al. (2011) | Operators of two types of marine radars (3, 9.4, and 5.5 GHz); average time on job 2-16 years | 0.0005-0.004 (time averaged) | Increased genetic damages in blood lymphocytes |
| Gautam et al. (2024) | Rats exposed to 2350 MHz RFR for 2 h/day for 56 days | 0.0625 | Male reproductive system, hematological system, liver, and kidney affected; reduction in testosterone, total antioxidant capacity, and increased lipid peroxidation in testis. |
| Gautam et al. (2025) | Rats exposed to 35.5 MHz RFR; 2 h/day for 60 days | 0.07-0.0564 | Sperm damage; increased DNA strand breaks; increased lipid peroxidation, decreased superoxide dismutase and total antioxidant status |
| * [Ghazizadeh and](http://www.ncbi.nlm.nih.gov/pubmed?term=Ghazizadeh%20V%5BAuthor%5D&cauthor=true&cauthor_uid=24792079) [Nazıroğlu (2014)](http://www.ncbi.nlm.nih.gov/pubmed?term=Naz%C4%B1ro%C4%9Flu%20M%5BAuthor%5D&cauthor=true&cauthor_uid=24792079) | Hippocampal cells of pentylentetrazol-induced epileptic rats exposed to 2450 MHz (Wi-Fi) RFR; 1 h | 0.000052 | enhanced reactive oxygen species in epileptic rat hippocampus |
| Gökçek-Saraç et al. (2017) | Rats exposed to 900 MHz or 2100 MHz RFR, 2 h/day for 1 week or 10 weeks | 900 MHz (whole body SAR 0.0053 W/kg); 2100 MHz (0.128 W/kg) | Enzymes of N-methyl-D-aspartate receptor-related signaling pathways in hippocampus higher in 10- week than in 1-week group |
| * Gremiaux et al. (2016) | Rose exposed to 900 MHz RFR, 3x 39 min every 48 h at 2 stages of development | 0.00072 | Delayed and reduced growth |
| Grigor'ev et al. (2010) | Blood serum of rats exposed to 2450 MHz RFR; 7 h/day for 30 days | 0.16 | Increased nitric oxide reaction products, lipid peroxidation, and hydroxylated fatty acids |
| Gulati et al. (2020) | Human lymphocytes exposed to UMTS signals at different frequency channels used by 3 G mobile phone (1923, 1947.47, and 1977 MHz) for 1 or 3 h | 0.04 | DNA damage found only in cells exposed to 1977-MHz field |
| [Güler](https://pubmed.ncbi.nlm.nih.gov/?sort=date&term=G%C3%BCler+G&cauthor_id=26520616) et al. (2016) | Rabbit exposed to 1800 MHz 217-Hz modulated RFR in utero and extra-utero | 0.018 | Increased 8-OHdG and lipid peroxidation in brain tissue |
| Gupta et al. (2018) | Rats exposed to 2450 MHz RFR; 1 h/day for 28 days | 0.0616 | Cognitive deficit, loss of mitochondrial functions, activation of apoptotic factors in hippocampus; affected cholinergic system |
| Gürler et al. (2014) | Brain and blood from rats exposed to 2450 MHz RFR; 1 h/day for 30 days | 0.02 | Increased lipid peroxidation in male and female; increased 8-hydroxydeoxyguanosine in female animals |
| Halgamuge et al. (2015) | Growth parameters of soybean seedlings; 2 h | GSM 217-Hz modulated (4.8 x 10^-7^, 4.9 x 10^-5^, and 0.0026 W/kg) or CW (0.00039 and 0.02 W/kg) 900-MHz | Modulated and CW fields produced different patterns of growth effects. There was an amplitude effect and extremely low-level modulated field (4.8 x 10^-7^ W/kg) affected all parameters |
| Hancı et al. (2013) | Rats exposed to 900 MHz RFR for 1 h/day during 13-21 days of pregnancy; testis of offspring examined on postnatal day 21 | 0.0265 | RFR caused irregularities in seminiferous tubule basal membrane and epithelium, immature germ cells in the lumen, and a decreased diameter in seminiferous tubules and thickness of epithelium; increased Apoptotic index, lipid peroxidation and DNA oxidation |
| Hancı et al. (2015) | Rats exposed to 900 MHz RFR for 1 h/day during 13-21 days of pregnancy; spleen and thymus of offspring examined on postnatal day 21 | 0.025 | Pathological changes in cell morphology in the thymic and splenic tissues;  lipid peroxidation increased and glutathione decreased in thymus whereas both were increased in the spleen. |
| Hanci et al. (2018) | Rats exposed to 900 MHz RFR, 1 h/day to postnatal day 60. | 0.0067 | Changes in morphology and increase in oxidative stress marker in testis |
| Hanci et al. (2025) | Offspring of pregnant rats exposed (1 h/day on day1-21 of pregnancy) to 900 MHz RFR | 0.0173, 0.0063, 0.0059 depending on size of animal | Change in morphology and oxidative damage in sciatic nerve. |
| Hatice et al. (2014) | Blood and brain of rats exposed to 2450 MHz RFR; 1 h/day for 30 days | 0.02 | Increased 8-hydroxydeoxyguanosine in plasma and brain and protein oxidation only in blood plasma |
| He et al. (2016) | Mouse bone marrow stromal cells exposed to 900 MHz RFR 3 h/day for 5 days | 2.5 x 10^-4^ | Increased expression of PARP-1 mRNA |
| He et al. (2017) | Mouse bone marrow stromal cells exposed to 900 MHz RFR 3 h/day for 5 days | 2.5 x 10^-4^ | Increased mRNA of poly(ADP-ribose) polymerase-1, cells treated with RFR and gamma radiation showed less genetic damage and faster kinetics of repair. |
| Hidisoglu et al. (2018) | Temporal cortex of rats exposed to GSM 2100 MHz RFR, 2 h/day for 7 days | Brain 0.27 | Increased lipid peroxidation |
| Hekmat et al. (2013) | Calf thymus exposed to 940 MHz RFR, 45 min | 0.04 | Conformational changes in DNA |
| Ivaschuk et al. (1997) | Nerve growth factor-treated PC12 rat pheochromocytoma cells 836.55 MHz TDMA signal,  20 min | 0.026 | Transcript levels for c-jun altered |
| Jech et al. (2001) | Narcoleptic patients; 900 MHz, GSM- 217 Hz pulses, 577 μs pulse width; 45 min | 0.06 | Improved cognitive functions |
| Jha et al. (2025) | Overy of rats exposed to 700 MHz RFR; 6 h/day for 10 days or 4 h/day for 60 days | 0.375 | Increased Oxidative lipid damage and decreased SOD; morphological changes |
| Jonwal et al. (2018) | Mice exposed to 2450 MHz RFR; 2 h/day for 30 days | 0.09 | In testis: increased catalase, lipid peroxidation, reactive oxygen species, decreased glutathione peroxidase, testosterone, and superoxide dismutase; histopathological changes in seminiferous tubules; increased micronucleus in blood. |
| Jooyan et al. (2023) | Chinese hamster ovary cells exposed to 900 MHz RFR (217-Hz modulation) for 24 h | 0.2-0.4 | Increased single strand DNA breaks, no effect on micronuclear formation, No effect on COX-2 gene expression. |
| * [Karadayi](https://pubmed.ncbi.nlm.nih.gov/?sort=date&term=Karadayi+A&cauthor_id=38466013) et al. (2024) | Rats exposed to 2450 MHz RFR 1 h/day during pregnancy and offspring to 45 days old | 0.00051 | Increased in soluble Receptor Activator of Nuclear factor-kB Ligand (sRANKL) and Osteoprotegerin (OPG) in serum and tibia; increased apoptosis. (sRANKL- involved in bone regeneration and remodeling; OPG-protein in regulation of bone density.) |
| Karamazi et al. (2025) | Pregnant rats exposed to 6,000 MHz RFR, 4 h/day in first 18 days of gestation | 0.054 | Fetal bone development affected. |
| Katirci et al. (2024) | Rats exposed to 2100 MHz RFR; 2 h/day for 1 or 10 weeks | Whole body 0.16; testis 0.0347 | Affected Tyro 3, Axl, and Mer (TAM) receptors and their ligands in testes, essential for spermatogenesis and testosterone production. |
| Keleş et al. (2019) | Rats exposed to 900 MHz RFR; 1 h/day for 25 days | 0.012 | Higher number of pyramidal and granule neurons in hippocampus |
| Keleş et al. (2024) | Rats exposed to 900 MHz RFR; 1 h/day for 25 days | 0.01 (from Aslan et al. Biotechnic &Histochemistry 92:324-330, 2017) | Increased lipid peroxidation, glutathione, catalase, and superoxide dismutase in kidney |
| Kerimoğlu et al. (2016a) | Lumbar spinal cord of rats exposed to 900 MHz RFR; 1 h/day on postnatal days 22-60 | 0.0093 | Increased lipid peroxidation; decreased superoxide dismutase, glutathione |
| Kerimoğlu et al. (2016b) | Brain of rats exposed to 900 MHz RFR; 1 h/day on postnatal days 21-60 | 0.0093 | Increased lipid peroxidation, glutathione; decreased catalase |
| Kerimoğlu et al. (2016c) | Heart of rats exposed to 900 MHz RFR; 1 h/day on postnatal days 21-59 | 0.0093 | Increased lipid peroxidation, superoxide dismutase; decreased glutathione, catalase |
| Kerimoğlu et al. (2018) | Sciatic nerve of rats exposed to 900 MHz RFR; 1 h/day on postnatal days 21-59 | 0.0093 | Increased lipid peroxidation, superoxide dismutase, catalase |
| * Kesari and Behari (2009) | Rats exposed to 50 GHz RFR; 2 h/day for 45 days | 0.0008 | Double strand DNA breaks observed in brain cells |
| * Kesari and Behari (2010a) | Rats exposed to 50 GHz RFR; 2 h/day for 45 days | 0.0008 | Changes in oxidative processes and apoptosis in reproductive system |
| Kesari and Behari (2010b) | Sperm of rat exposed to 2450 MHz RFR; 2 h/day for 35 days | 0.11 | Decreased glutathione peroxidase, superoxide dismutase; increased catalase |
| Kesari et al. (2010) | Rats exposed to 2450 MHz RFR at 50-Hz modulation, 2 h/day for 35 days | 0.11 | DNA double strand breaks in brain cells |
| Kesari et al. (2014) | Rats exposed to a 3D cell phone. 2 h/day for 60 days | 0.26 | Increased DNA double strand breaks, micronuclei, Caspase 3 and apoptosis in brain cells; activation of hsp27/p38MAPK stress pathway. |
| Kues et al. (1992) | Monkeys (M. mulatta and M. fascicularis) treated with timolol or pilocarpine exposed to pulsed 2450 MHz RFR, 4 h/day for 3 days | 0.26 | Corneal endothelial damage |
| Kulaber et al. (2017) | Thymus of rats exposed to 900 MHz RFR; 1 h/day between postnatal days 22-59 | 0.067 | Increased lipid peroxidation |
| Kumar et al. (2010a) | Rats exposed to 10 GHz RFR, 2 h/day for 45 days | 0.014 | Cellular changes and increase in reactive oxygen species in testes |
| Kumar et al. (2010b) | Rats exposed to 10 GHz RFR, 2 h/day for 45 days; or 50 GHz, 2 h/day for 45 days | 0.014 (10 GHz);  0.0008 (50 GHz) | Genetic damage in blood cells |
| Kumar et al. (2011a) | Rats exposed to 2450 MHz RFR; 2 h/day for 60 days | 0.014 | Increases in caspase and creatine kinase in sperm and decreases in testosterone and melatonin in blood |
| Kumer et al. (2011b) | Spermatozoa of rats exposed to 10 GHz RFR, 2 h/day for 45 days | 0.014 | Increased reactive oxygen species |
| Kumar et al. (2012) | Rats exposed to 10-GHz RFR; 2 h/day for 45 days | 0.014 | Increased creatine phosphate and decreased melatonin and lipid peroxidation in sperms |
| Kumar et al. (2013) | Rats exposed to 10- GHz RFR; 2 h/ day for 45 days | 0.014 | Increased micronucleus in blood cells and DNA strand breaks in spermatozoa. |
| Kumar et al. (2016) | Zea mays seedling exposed to 1800-MHz RFR, for 0.5, 1, 2, or 4 h | 0.169 | longer exposure of 4 h caused significant growth and biochemical alterations |
| Kumar et al. (2020) | Onion exposed to 900 MHz or 1800 MHz RFR for 0.5-4 h | 900 MHz- 0.092; 1800 MHz- 0.169 | Effects on root length and thickness, micronucleus formation. DNA strand breaks observed. 1800 MHz more potent that 900 MHz field |
| Kumar et al. (2021) | Hippocampus of rats exposed to 900, 1800, or 2450 MHz RFR for 2 h/day for 1-month, 3-month and 6-month | Rats exposed to 900 MHz, 1800 MHz, and 2450 MHz RFR at a SAR of 5.84 × 10^-4^ W/kg, 5.94 × 10^-4^ W/kg and 6.4 × 10^-4^ W/kg, respectively. | Significant epigenetic modulations were observed in the hippocampus, larger changes with increasing frequency and exposure duration. |
| Kunjilwar and Behari (1993) | Rats exposed to 147 MHz and subharmonic 73.5 and 36.75 MHz amplitude modulated at 16 and 76 Hz; 2 h/day for 30-35 days | 0.1-0.14 | Decreased acetylcholine esterase in brain |
| Kuybulu et al. (2016) | Rats exposed to 2450 MHz RFR either prenatally (in utero) or postnatally (18 days to 12^th^ week old); 1h/day | 0.143 | Caused chronic kidney damages- histologically and biochemically.  Increased lipid peroxidation; decreased superoxide dismutase |
| Kuzay et al. (2017) | Testis of normal and diabetic rats exposed to 2100 MHz RFR; 20 min/day, 5 day/week for one month. | 0.23 | Increased lipid peroxidation and nitric oxide; decreased glutathione |
| Kwee et al. (2001) | Transformed human epithelial amnion cells exposed to 960 MHz GSM signal, 20 min | 0.0021 | Increased Hsp-70 stress protein |
| Lameth et al. (2025) | Cerebral cortex of Rats head exposed to 3.5 GHz RFR, 1 h/day (5 days/week) for 6 weeks. | 0.19 | Up-regulation of genes related to glutamatergic synapses |
| Lerchl et al. (2008) | Hamsters exposed to 383 MHz (TETRA), 900 and 1800 MHz (GSM)  24 h/day, 60 days | 0.08 | Metabolic changes in hamster |
| Lian et al. (2018) | Yeast strains (NT64C and SB34)  exposed to 2000 MHz RFR for 96 h | 0.12 | Increased generation and propagation of yeast prions, level of reactive oxygen species, and activities of superoxide dismutase and catalase |
| Lin et al. (2016) | Budding yeast exposed to 2000 MHz RFR for 96 h | 0.12 | Upregulate the expression of genes involved in glucose transportation and the tricarboxylic acid (TCA) cycle, but not the glycolysis pathway. |
| [López-Furelos](https://pubmed.ncbi.nlm.nih.gov/?sort=date&term=L%C3%B3pez-Furelos+A&cauthor_id=27589837) et al. (2016) | Rats exposed to 900 or 2450 MHz RFR or combination of the two frequencies for 1-2 h | 0.0367-0.0723 (average whole body) | Changes in HSP 90 and 70 levels in cerebrum and cerebellum |
| López-Furelos et al. (2018) | RAW 264.7 cells (mouse macrophage-like leukemia virus transformed cells) exposed to 900 and 2450 MHz single or combined RFR; 4, 24, 48, or 72 h | 0.0842-0.406 | Increased nitric oxide depending on exposure time. (Decreased phagocytic activity) |
| López-Martín et al. (2009) | Picotoxin-induced seizure-prone rats exposed to pulse-modulated GSM and unmodulated signals; 2 h | 0.03-0.26 | c-Fos expression in brain |
| Maalouf et al. (2023) | Adipose tissues of rats exposed to 900 MHz CW RFR; 1 h twice a day for 3 or 7 days | 0.1 or 0.4 | Changed (increase and decrease) expression of genes involved in lipid, metabolism, energy homeostasis, and thermal regulation, |
| Manta et al. (2014) | Drosophila melanogaster exposed to radiation from a wireless DECT (Digital Enhanced Cordless Telephone) base radiation (1.88-1.90 GHz; pulsed (at 100 Hz rate and 0.08 ms duration) for 0.5 - 96 h | 0.009 | Increased reactive oxygen species in male and female at 6 h; ovary reactive oxygen species level increased after 0.5 h and reached maximum after I h exposure |
| Manta et al. (2017) | Ovary of Drosophila melanogaster exposed to radiation from a GSM 1800 MHz mobile phone in talk mode for 30 min | 0.15 | Increased reactive oxygen species production immediately, non-targeted transcriptional reprogramming of gene expression at 2 h, and apoptosis at 4 h post-exposure. |
| Makova et al. (2005) | Human white blood cells exposed to 915 and 905 MHz GSM signal, 1 h | 0.037 | Altered chromatin conformation |
| Markova et al. (2010) | Human diploid VH-10 fibroblasts and human adipose-tissue derived mesenchymal stem cells exposed to GSM (905 MHz or 915 MHz) or UMTS (1947.4 MHz, middle channel) RFR for 1, 2, or 3 h | 0.037-0.039 | Inhibited tumor suppressor TP53 binding protein 1 (53BP1) foci that are typically formed at the sites of DNA double strand break location. |
| Marinelli et al. (2004) | Human leukemia cell exposed to 900 MHz CW RFR  2 - 48 h | 0.0035 | Cell’s self-defense responses triggered by DNA damage. |
| Meena et al. (2014) | Rats exposed to 2450 MHz RFR; 2 h/day for 45 days | 0.14 | Induced oxidative stress in testis; effects reversed by melatonin |
| * Megha et al. (2012) | Rats exposed to 900 MHz or 1800 MHz, 2 h/day for 30 days | 900 MHz -0.00059; 1800 MHz -0.00058 | Impaired cognitive functions; Increased lipid peroxidation, and protein oxidation; decreased glutathione in brain |
| * Megha et al. (2015a) | Rats exposed to 900 and 1800 MHz RFR for 30 days (2 h/day, 5 days/week) | 0.00059 and 0.00058 | Reduced levels of neurotransmitters dopamine, norepinephrine, epinephrine, and serotonin, and downregulation of mRNA of tyrosine hydroxylase and tryptophan hydroxylase (synthesizing enzymes for the transmitters) in the hippocampus. |
| * Megha et al. (2015b) | Rats exposed to 900, 1800, and 2450 MHz RFR for 60 days (2 h/day, 5 days/week) | 0.00059, 0.00058, and 0.00066 | Increased DNA damage in the hippocampus |
| Migdal et al. (2023) | Bees exposed to 900 MHz RFR for 0.25, 1, or 3 h | 0.05, 0.3, or 1.4 | Increased expression of Hsp70 and Hsp 90 genes (at 0.05 W/kg for 3 h) |
| Misa Agustiño et al. (2012) | Rats exposed to 2450 MHz RFR for 30 min | 0.046 | Decreased HSP-90 and HSP-70 in thyroid gland. |
| Mohammed et al. (2013) | Rats exposed to 900 MHz continuous-wave, 900 MHz modulated at 8 and 16 Hz; 1 h/day for 1 month | Spatial peak SAR 0.245 | Increased latency of REM sleep |
| Nakamura et al. (1998) | Pregnant rats exposed to 2450 MHz RFR for 90 min | 0.36-0.44 | Affected beta-endorphin in blood and anterior pituitary gland, blood progesterone, and splenic natural killer cell activity |
| Navakatikian and Tomashevskaya (1994) | Rats exposed to 2450 MHz CW and 3000 MHz pulse-modulated 2-μs pulses at 400 Hz,  Single (0.5-12 h) or repeated (15-60 days, 7-12 h/day) | 0.0027 | Behavioral and endocrine changes, and decreases in blood concentrations of testosterone and insulin.  CW-no effect |
| [Naziroğlu and](http://www.ncbi.nlm.nih.gov/pubmed?term=Naziro%C4%9Flu%20M%5BAuthor%5D&cauthor=true&cauthor_uid=19637079) [Gümral (2009)](http://www.ncbi.nlm.nih.gov/pubmed?term=G%C3%BCmral%20N%5BAuthor%5D&cauthor=true&cauthor_uid=19637079) | Brain of rats exposed to 2450 MHz (217 Hz pulses) RFR; 1 h/day for 28 days | 0.1434 | Decreased vitamin C, E, and A |
| Nazıroğlu et al. (2012a) | Human leukemia 60 cells exposed to 2450-MHz RFR for 1, 2, 12, or 24 h | 0.11 | Increased in cytosolic free Ca²⁺ concentrations; increased lipid peroxidation |
| Nazıroğlu et al. (2012b) | Brain and dorsal root ganglion neuron of rats exposed to 2450 MHz 217 Hz modulation RFR; 1 h/day for 30 days | 0.1 | Increased lipid peroxidation in dorsal root ganglion neurons, attenuated by melatonin (RFR induced calcium influx in brain and ganglion neurons attenuated by melatonin.) |
| Nittby et al. (2008a) | Rats exposed to 915 MHz GSM signal, 6 h | 0.013 (whole body average SAR);  0.03 (head) | Altered gene expression in cortex and hippocampus |
| * Nittby et al. (2008b) | Rats exposed to GSM-900 MHz RFR, 2 h/week for 55 weeks | 0.0006 | Cognitive impairment |
| Odaci and Özyılmaz (2015) | Testis of rats exposed 900 MHz RFR; 1h/day for 30 days. | 0.025 | Produced vacuoles in seminiferous tubules basal membrane and edema in the inter-tubular space and apoptosis; decreased lipid peroxidation, catalase, superoxide dismutase, and glutathione. |
| Odaci et al. (2015) | Pregnant rats exposed to 900 MHz RFR, 1 h/day, on days 13-21 of pregnancy. | 0.024 | Kidney tissue of 21-day old male offspring showed histological degeneration, increased lipid peroxidation. And decreased catalase and superoxide dismutase. |
| Odaci et al. (2016) | Pregnant Sprague -Dawley rats exposed to 900 MHz RFR 1 h/day during days 13 - 21 of pregnancy. | 0.024 | Testis and epididymis of offspring showed higher DNA oxidation |
| Okatan et al. (2018) | Ovary of rats exposed to 900 MHz RFR; 1 h/day from 35-59 days old | 0.0098 | Histopathological changes observed |
| Okatan et al. (2019) | Liver of rats exposed to 900 MHz RFR; 1 h/day on 35-59 postnatal days | 0.0096 | Decreased 8-hydroxydeoxyguanosine and superoxide dismutasis |
| Oksay et al. (2014) | Testis of rats exposed to 2450 MHz RFR; 1 h/day for 30 days | 0.1 | Increased lipid peroxidation; attenuated by melatonin |
| Özdemir et al. (2021) | Optic nerve of rats exposed to LTE-Advanced Pro (4.5 G) mobile phone (00-2600 MHz); 2 h/day for 6 weeks | 0.0035 at eye | Increased lipid peroxidation; decreased catalase and superoxide dismutase |
| Ozguner et al. (2005a) | Kidney of rats exposed to 900 MHz RFR; 30 min/day for 3 months | 0.016 | Increased lipid peroxidation and nitric oxide; decreased superoxide dismutase, catalase, and glutathione peroxidase |
| Ozguner et al. (2005b) | myocardial tissue of rats exposed to 900 MHz (217 Hz pulse modulation) RFR; 30 min/day for 10 days | 0.016 | Increased lipid peroxidation and nitric oxide; decreased superoxide dismutase, catalase, and glutathione peroxidase |
| Ozgur et al. (2010) | Guinea pigs exposed to 1800-MHz GSM signal, 10 or 20 min/day for 7 days | 0.38 | Changes in oxidative status in the liver |
| Ozlem Nisbet et al. (2012) | Rats exposed to 1800 or 900 MHz RFR; 2 h/day for 90 days | 0.003 | Increased testosterone in blood; morphological changes in testis dependent on frequency |
| Ozorak et al. (2013) | Kidney and testis of rats exposed to 2450, 900, and 1800 MHz (217 Hz modulation) RFR; 1 h/day prenatal to 6 weeks of age | 0.18 | Increased lipid peroxidation in kidney and testis; decreased total antioxidant status, glutathione, glutathione peroxidase at 4^th^ week postnatal; Decreased glutathione, total antioxidant status in kidney at 5^th^ week; Increased lipid peroxidation, decreased total antioxidant status and glutathione in kidney and testis at 6^th^ week. |
| Özyılmaz et al. (2024) | Thyroid of 6-month old offspring of pregnant rats exposed to 2450 MHz RFR, 24 h/day during pregnancy period | 0.012 | Morphological changes in mononuclear cell infiltration and vascular increase in congestion. |
| Özyılmaz et al. (2025) | Thyroid of 12-month old offspring of pregnant rats exposed to 2450 MHz RFR, 24 h/day during pregnancy period | 0.012 | Increases in fibrosis, atypical thyrocytes , and degenerated follicles, and colloid reduction. Increased apoptosis and double strand DNA breaks. |
| Pandey and Giri (2018) | Mice exposed to 900 MHz GSM RFR; 3 h twice/day for 35 days | 0.0516 | Testis showed morphological changes, increased DNA strand breaks and lower sperm count, and increased free radical formation, Effects attenuated by melatonin. |
| Pandey et al. (2017) | Mice exposed to 900-MHz RFR for 4 or 8 h per day for 35 days | 0.0054-0.0516 | DNA strand breaks in germ cells |
| Pavicic et al. (2006) | Chinese hamster V79 cells exposed to 864 and 935 MHz CW RFR, 1-3 h | 864 MHz at 0.08 W/kg; 935 MHz at 0.12 W/kg | Cell growth affected |
| * Pelletier et al. (2013) | Rat, 900 MHz, 23.4 h/day for 5 weeks | 0.0001-0.0003 | Induced energy saving process in body |
| * [Pérez-Castejón et al.](http://www.ncbi.nlm.nih.gov/pubmed?term=%22P%C3%A9rez-Castej%C3%B3n%20C%22%5BAuthor%5D&itool=EntrezSystem2.PEntrez.Pubmed.Pubmed_ResultsPanel.Pubmed_RVAbstract) (2009) | human astrocytoma cancer cells; 9600 MHz, 90% AM, 24 h | 0.0004 | Increase proliferation rate and connexin 43 expression |
| Perov et al. (2019) | Rats exposed to 171 MHz CW RFR,  6 h/day for15 days | 0.006 | Stimulation of adrenal gland activity |
| * Persson et al. (1997) | Rats exposed to 915 MHz RFR -CW and pulse-modulated (217-Hz, 0.57 ms; 50-Hz, 6.6 ms) 2-960 min. | 0.0004 | Increase in permeability of the blood-brain barrier.  CW more potent |
| Phillips et al. (1998) | Human leukemia cells exposed to 813.5625 MHz (iDEN); 836.55 MHz (TDMA) signals, 2 or 21 h | 0.0024 | DNA damage observed |
| Piccinetti et al. (2018) | Zebrafish exposed to 100 MHz RFR,  24-72 h post-fertilization | 0.08 | Retarded embryonic development |
| Porcher et al. (2023) | Leaves of Arabidopsis thaliana exposed to 2450 MHz RFR for 30 min | 0.21 | Increased H_2_O_2_ |
| Postaci et al. (2018) | Rats exposed to 2600 MHz RFR, 1 h/day for 30 days | 0.011 | Cellular damages and oxidative damages in liver |
| * Pyrpasopoulou et al. (2004) | Rats exposed to 9.4 GHz GSM  (50 Hz pulses, 20 μs pulse length) signal, 1-7 days postcoitum | 0.0005 | Exposure during early gestation affected kidney development |
| Qin et al. (2014) | Rats exposed 1800 MHz RFR 2 h/day for 32 days | 0.0405 | Disruption of circadian rhythms, decreased testosterone levels, lower daily sperm production and sperm motility, down-regulated testis marker enzyme activity, and altered mRNA expression of cytochrome P450 and steroidogenic acute regulatory protein. Effect more pronounce at a certain time of the day. |
| Qin et al. (2018) | Mice exposed to 1800-MHz RFR, 2 h/day for 32 days | 0.0553 | Inhibition of testosterone synthesis |
| Qin et al. (2019) | Mouse Leydig cells exposed to 1800 MHz RFR; 1, 2, or 4 h | 0.116 | Down-regulation of testosterone synthase genes and clock genes. |
| Rafati et al. (2015) | Frog gastroenemius muscle exposed to cell phone jammers; 1 m away, 3 x 10 min periods | For different jammers:  0.01-0.05 | Latency of contraction affected |
| * Sagioglou et al. (2016) | Drosophila melanogaster exposed to 100, 395, 682, 900 MHz RFR for 6 or 60 min/day for 6 days post-hatching | 0.00001- 0.102 | All exposure protocols resulted in an increase of apoptosis in egg chambers |
| Salford et al. (2003) | Rats exposed to 915 MHz GSM, 2 h | 0.02 | Nerve cell damage in brain |
| Sangun et al. (2015) | Rats exposed to 2450 MHz RFR during prenatal and postnatal periods; 1 h/day | 0.143 | Increased total oxidant status and oxidative stress index in brain and testis. |
| Sannino et al. (2014) | Human lymphocytes exposed to 1950 MHz RFR for 20 h and then challenged with 1.0 or 1.5 Gy X-irradiation | 0.3 | A decrease in the number of micronuclei in lymphocytes exposed to RFR + x-ray as compared with those subjected to x-ray alone |
| Sarimov et al. (2004) | Human lymphocytes exposed to 895-915 MHz GSM signal,  30 min | 0.0054 | Chromatin affected similar to stress response. |
| Schwarz et al. (2008) | Human fibroblasts exposed to 1950 MHz UMTS signal,  24 h | 0.05 | Changes in genes. |
| * Schwartz et al. (1990) | Frog hearts exposed to 240-MHZ RFR CW or sinusoidally modulated at 0.5 or 16 Hz for 30 min. | 0.00015-0.003 | Movement of calcium affected only with 16-Hz modulation at 0.003 and 0.00015 W/kg. No effect with CW and 0.5 Hz modulation. |
| Seewooruttun et al. (2025) | Adipose tissue from rats exposed to 3.5 GHz (0.07 W/kg) ot 900 MHz (0.24 W/kg) RFR; I h/day for 1 or 2 weeks. | 0.07 | Decreased in adipogenic biomarker gene expression- PRDM16 and C/EBPβ. Both genes are involved in the proliferation and differentiation of mature brown adipocytes from precursors. |
| Sefidbakht et al. (2014) | HEK293T cell with firefly luciferease gene exposed to a 940 MHz RFR for 15-90 min | 0.09 | Endogenous luciferase activity was reduced after 30 and 45 min of continuous exposure, while after 60 min, the exposed cell lysate showed higher luciferase activity |
| Sepehrimanesh et al. (2014a) | Rats exposed to 900 MHz RFR for 1, 2, or 4 h/day for 30 days | 0.19-1.22 (range of whole body SAR) | Disturbance in reproductive hormones |
| Sepehrimanesh et al. (2014b) | Rats exposed to 900 MHz RFR for 1, 2, or 4 h/day for 30 days | 0.19-1.22 | Changes in proteome, particularly in protein species in the testis |
| Sepehrimanesh et al. (2017) | Rats exposed to 900 MHz RFR for 1, 2, or 4 h/day for 30 days | 0.19-1.22 | Increases in testicular proteins in adults that are related to carcinogenic risk and reproductive damage |
| Shahin et al. (2013) | Mice exposed to 2450 MHz RFR, 2 h/day for 45 days | 0.023 | Increased DNA strand breaks in brain; decreased nitric oxide in blood; Increased reactive oxygen species, decreased superoxide dismutase, catalase. Glutathione peroxidase in liver, kidney, and Ovary |
| Shahin et al. (2014) | Mice exposed to 2450 MHz RFR; 2 h/day for 30 days | 0.018 | Decreased sperm count and viability, plasma testosterone; increased testicular nitric oxide synthase |
| Shahin and Singh (2018) | Mice exposed to 2450 MHz RFR; 2 h/day for 15, 30, or 60 days | 0.0146 | Induced redox imbalance and apoptosis in testis, |
| Sharma et al. (2017) | Brain of mice exposed to 10 GHz RFR 2 h/day for 15 days | 0.179 | Changes in oxidative enzymes, deficit in spatial memory, and histological changes in CA1 region of the hippocampus, cerebral cortex, and ansiform lobule of cerebellum. Effects persisted for at least 4 weeks. |
| Sharma and Shukla (2020) | Rats exposed to 900 MHz RFR for 1, 2, or 4 h/day for 90 days | Brain- 0.231 | Increased DNA single strand breaks  and increased oxidative stress in brain. |
| Sharma et al (2024) | Trigonella foenum-graecum L. seeds exposed to 2100 or 2300 MHz RFR for 0.5, 1,2 , 4, 8 h/day for 7 days | 0.098 | Increased chromosomal aberration, increased lipid peroxidation and antioxidant enzyme activities. |
| Singh et al. (2022) | Rats exposed to 1964.7 MHz RFR, 3 h/day, 5 days/week for 20 weeks | 0.015 | Protein deregulation in hippocampus |
| * Sirav and Seyhan (2011) | Rats exposed to CW 900 MHz or 1800 MHz for 20 min | CW 900 MHz (0.00426 W/kg) or 1800 MHz (0.00146 W/kg) | Increased blood-brain barrier permeability in male rats, no significant effect on female rats |
| Sirav and Seyhan (2016) | Rats exposed to pulsed-modulated (217 Hz, 517 μs width) 900 MHz or 1800 MHz RFR for 20 min | 0.02 | In male rats, both frequencies increased blood-brain barrier permeability, 1800 MHz is more effective than 900 MHz; in female rats, only 900 MHz field caused an effect |
| Sokolovic et al. (2008) | Rats exposed to 900 MHz GSM RFR from a mobile phone; 4 h/day for 20, 40, or 60 days | 0.043-0.135 | Increased lipid peroxidation, protein oxidation; Decreased CAT, xanthine oxidase in brain |
| Sokolovic et al. (2013) | Rats exposed to 900 MHz GSM RFR from a mobile phone; 4 h/day for 20, 40, or 60 days | 0.043-0.135 | Increased lipid peroxidation, protein oxidation; Decreased CAT, xanthine oxidase; increased acid- and alkaline DNAse in thymus |
| Sokolovic et al. (2015) | Rats exposed to 900 MHz GSM RFR from a mobile phone; 4 h/day for 20, 40, or 60 days | 0.043-0.135 | Increased lipid peroxidation, protein oxidation, xanthine oxidase; decreased CAT in testis |
| Somosz et al. (1991) | Rat embryo 3T3 cells exposed to 2450-MHz 16-Hz square modulated RFR | 0.024 | Increased the ruffling activity of the cells, and caused ultrastructural alteration in the cytoplasm. CW was less effective |
| Spandole-Dinu et al. (2023) | Brain of mice exposed to 2450 MHz RFR with signal of a Wi-Fi router fed to an antenna for 16 weeks | 0.01786 | Brain global DNA methylation was lower in exposed mice. |
| Stagg et al. (1997) | Glioma cells exposed to 836.55 MHz TDMA signal, duty cycle 33%, 24 h | 0.0059 | Glioma cells showed significant increases in thymidine incorporation, which may be an indication of an increase in cell division |
| Stankiewicz et al. (2006) | Human white blood cells exposed to 900 MHz GSM signal, 217 Hz pulses-.577 ms width, 15 min | 0.024 | Immune activities of human white blood cells affected. |
| Stasinopoulou et al. (2016) | Rats exposed to wireless 1880-1900MHz Digital Enhanced Communication Telephony (DECT) base radiation; 12 h/day during pregnancy; offspring exposed for another 22 days | 0.016-0.020 | Pyramidal cell loss and glia fibrillary acidic protein (GFAP) over-expression were detected in the CA4 region of the hippocampus of the 22-day old pups. |
| Suhhova et al. (2013) | Human exposed to 450 MHz RFR modulated at 40 Hz (1 min ON/1 min OFF), 10 min each for 10 cycles | 0.003 | Affected alpha, beta 1 and beta 2 bands of resting EEG in 20% of subjects; effects were intensity-dependent |
| Sun Y. et al. (2017) | Human HL-60 cells exposed to 900 Hz RFR, 4 h/day for 5 days | peak and average SAR 4.1 x 10^-4^ and 2.5 x 10^-4^ W/kg | Increased oxidative DNA damage and reactive oxygen species; decreased mitochondrial gene expression |
| Szymanski et al. (2020) | Human blood mononucleus cells exposed to pulse-modulated 900 MHz RFR, two 15-min exposure | 0.024 | Cells demonstrated high immunological activity of monocytes and T-cell response to concanavalin A |
| Tahir et al. (2024) | Pregnant rats exposed to 2450 MHz RFR during pregnancy and offspring for 45 days after birth (1 h/day) | Cochlear SAR showing effects: 0.0023- 0.032 | Inner ear damage and apoptosis in cochlea of offspring. |
| Tan et al. (2022) | Rats exposed to 2450 MHz RFR; 12 h/day; starting one month before fertilization to the offspring were two months old. Exposure continues for 4 generations. | Adult: whole body 0.186, thymus 0.068; offspring: whole body 0.287, thymus 0.116 | The number of offspring and mass of all rats decreased in the third-generation group. Vascularization was observed in the thymus of the fourth-generation offspring. |
| Tang et al. (2015) | Rats exposed to 900 MHz RFR, 3 h/day for 14-28 days | 0.016 | Spatial long-term memory deficit |
| Tas et al. (2014) | Rats exposed to 900 MHz RFR; 3 h/day for 1 year | 0.0623 | RFR altered some morphological parameters in the testis. |
| * Tattersall et al. (2001) | Rat hippocampus, 700 MHz, 5-15 min | 0.0016 | Changes in hippocampal functions |
| * Tkalec et al. (2013) | Earthworm exposed to continuous-wave and AM-modulated 900-MHz RFR for 2 - 4 h | 0.00013, 0.00035, 0.0011, and 0.00933 | Increased DNA strand breaks |
| Tomruk et al. (2022) | Rabbits exposed to 1800 MHz GSM signal; 15 min/day for 7 days during pregnancy (15^th^ to 22^nd^ day) | 0.002 | Cellular reactive oxygen species- dependent disturbances in metabolic activity in liver of offspring. |
| Topal et al. (2015) | Pregnant rats exposed to 900 MHz RFR; 1 h/day during days 13-21 of pregnancy | 0.027 | Induced oxidative stress and pathological alterations in the liver in the offspring. |
| Tripathi et al. (2023) | Rats exposed to 1760 MHz RFR; 2 h/day for 8 weeks | 0.18 | Disrupted insulin receptor signals, mitochondrial activity, and the antioxidant defense system in the hypothalamus and liver. |
| [Trosić](https://pubmed.ncbi.nlm.nih.gov/?sort=date&term=Trosi%C4%87+I&cauthor_id=19329382) and  [Pavicić](https://pubmed.ncbi.nlm.nih.gov/?sort=date&term=Pavici%C4%87+I&cauthor_id=19329382) (2009) | Chinese hamster V79 cells exposed to 935 MHz RFR for 1-3 h | 0.12 | Declined proliferation and microtubule structure disruption observed |
| Tsoy et al. (2019) | Human and rat primary astrocytes exposed to 918 MHz RFR pulsed (0.58 ms) AM at 217 Hz; 24 h | 0.2 | Decreased β-amyloid-induced oxidative stress |
| Tsybulin et al. (2013) | Japanese Quail embryos exposed to GSM 900 MHz signal, 48 sec on/12 sec off; 38 or 158 h | 0.003 | Decreased DNA strand break at 38 h and increased in 158 h exposure in cells. |
| [Türedi](https://pubmed.ncbi.nlm.nih.gov/?sort=date&term=T%C3%BCredi+S&cauthor_id=25166431) et al. (2015) | Pregnant rats exposed to 900 MHz RFR; 1 h/day on days 13-21 of pregnancy | 0.025 | Oxidative stress and histopathological changes in male rat offspring heart tissue detected. |
| [Türedi](https://pubmed.ncbi.nlm.nih.gov/?sort=date&term=T%C3%BCredi+S&cauthor_id=25166431) et al. (2016) | Pregnant rats exposed to 900 MHz RFR; 1 h/day on days 13-21 of pregnancy | 0.01 | Decrease in ovarian follicle reservoirs in female off spring at the beginning of the prepubertal period. |
| Türedi et al. (2017) | Kidney and bladder of rats exposed to 900 MHz RFR; 1 h/day from 22-59 days of age | 0.0067 | Increased lipid peroxidation; decreased catalase and glutathione. |
| Upadhyaya et al. (2022) | Tomato plants exposed to 1800 MHz RFR up 12-120 h | 0.0316 (leaves);  0.15 (fruits) | Decreased total antioxidant activities and other chemicals. |
| Vafaei et al. (2020) | Placenta of pregnant mice exposed to 2.4 GHz RFR from a D-link Wi-Fi router; 2 or 4 h/day at 30 or 60 cm from router from 5 days after mating to 1 day before expected delivery | 0.09  at 30 cm | Increased gene expression of SOD, and GDKN1A, and GADD45a (DNA repair enzymes) |
| * Velizarov et al. (1999) | Human epithelial amnion cells exposed to 960 MHz GSM signal,  217 Hz square-pulse, duty cycle 12%, 30 min | 0.000021 | Decreased proliferation |
| Veyret et al. (1991) | Mice exposed to 9.4 GHz 1 μs pulses at 1000 pps, also with or without sinusoidal AM between 14 and 41 MHz; 10 h/day for 5 days. Response only with AM modulation, direction of response depended on AM frequency | 0.015 | Changes in functions of the mouse immune system. |
| Wang S et al. (2025) | Mice exposed to 2450 MHz RFR, 3 h/day for 8 weeks; testes studied. | 0.3 | Decreased SOD, CAT, GSH; increased LPO and 8OHdG; morphological changes in spermatozoa |
| * Wang XW et al. (2010) | Mice exposed to 200 electromagnetic pulses | average SAR 0.00004 | Blood-testis barrier injury detected. |
| Wang Y et al. (2022) | Mating Drosophilia melanogaster exposed to 3500 MHz RFR for three days. | 0.0026, 0.026, and 0.26 | Enhanced pup development observed at 0.0026 W/kg |
| Wang Y et al. (2025) | Male Drosophilia melanogaster exposed to 3500 MHz RFR for whole lifetime | 0.0026, 0.026, and 0.26 | Decreased ROS and CAT at 0.026 W/kg |
| Wen et al. (2021) | Mouse bone marrow stromal cells exposed to 900 MHz RFR; 4 h/day for 5 days | Peak and average SAR 4.1 × 10^−4^ and 2.5 × 10^−4^ W/kg, respectively | Increased reactive oxygen species at 30 min and 4 h post-exposure (normal at 24 h) |
| * Wolke et al. (1996) | Isolated guinea pig heart muscle cells exposed to 900, 1300, 1800 MHz, square-wave modulated at 217 Hz; Also 900 MHz with CW, 16 Hz, 50 Hz and 30 KHz modulations, 500 sec | 0.001 | Changed calcium concentration in heart muscle cells |
| Xie et al. (2021) | Mouse bone marrow stem cells; CW 900 MHz RFR; 4 h/day for 5 days | Peak 4.1 x 10^-4^; average 2.5 x 10^-4^ | Increased reactive oxygen species free radicals; induced mitochondrial unfolded protein. |
| Xu et al. (2020) | Mice exposed to 1800 MHz RFR; 2 h/day for 7 days | 0.2986 | Decreased sperm motility and increased sperm deformity rate; decreased the antioxidant enzymes activities of G6 PDH, ACP and CAT, and increased lipid peroxidation in testicular tissue |
| Yavas et al (2024) | Rats exposed to 2100 MHz RFR, 5 h/day for 14 days | 0.292 | Decreased Bax, increased bcl-2 gene expression, increased double strand DNA breaks in brain tissue |
| Yilmaz et al. (2017) | Pregnant rats exposed to 900 MHz RFR; 24 h/day for 20 days | 0.087 | Histological damage and changes in oxidative chemicals in liver of offspring at 60 days old. |
| Yilmaz et al. (2025) | Rats exposed to 589 GHz RFR 2 h/day or 30 days | 0.0213 | Histological damage and changes in oxidative chemicals in spermatozoa and testis. |
| Yüksel et al. (2016) | Pregnant rats and offspring exposed to 900, 1800, or 2450 MHz RFR; 60 min/day during pregnancy and growth (experiment over 4 generation) | 0.1 | Decreased plasma prolactin, progesterone, and estrogen levels, and increased uterine oxidative stress in pregnant rats and their offspring. |
| Yurekli et al. (2006) | Rats exposed to 945 MHz GSM, 217 Hz pulse-modulation  7 h/day for 8 days | 0.0113 | Changes in free radical chemistry |
| Zeni et al. (2012) | Human blood lymphocytes exposed to 1950 MHz UMTS RFR for 20 h at 1.25, 0.6, 0.3, or 0.15 W/kg, and treated with mitomycin C | 0.3 | RFR at 0.3 W/kg reduced micronucleus formation induced by mitomycin C. |
| Zong et al. (2015) | Mice exposed to 900 MHz RFR, 4 h/day for 7 days | 0.05 | Attenuated bleomycin-induced DNA breaks and repair |
| [Zosangzuali](https://pubmed.ncbi.nlm.nih.gov/?sort=pubdate&term=Zosangzuali+M&cauthor_id=33687298) et al. (2021) | Brain, heart, kidney and liver of mice exposed to 1800 MHz RFR (12 m from a mobile phone base station); 6, 12, or 24 h/day for 45 days | 0.013 | Decreased glutathione, , glutathione S-transferase, and superoxide dismutase, increased lipid peroxidation in brain at 12 and 24 h/day exposure; no effect on heart, kidney and liver |

**Acute exposure Repeated/chronic exposure**

**--------------------------------------------------------**

**In vivo 32 165 197 (79%)**

**-------------------------------------------------------------------------------------------------------**

**In vitro 42 9 51 (21%)**

**-------------------------------------------------------**

**74 (30%) 174(70%) 248**

**SAR: Mean = 0.071 W/kg; median = 0.026 W/kg; n = 248 (range: 0.000003-0.3951)**

**When studies that reported effects at SAR < 0.08 W/kg (limit of local exposure in most guidelines) are considered: Mean = 0.019 W/kg; median = 0.013 W/kg; n = 167 (range: 0.000003-0.08).**

**References**

[Adey](https://pubmed.ncbi.nlm.nih.gov/?sort=date&term=Adey+WR&cauthor_id=6812594) WR,  [Bawin](https://pubmed.ncbi.nlm.nih.gov/?sort=date&term=Bawin+SM&cauthor_id=6812594) SM,  [Lawrence](https://pubmed.ncbi.nlm.nih.gov/?sort=date&term=Lawrence+AF&cauthor_id=6812594) AF. Effects of weak amplitude-modulated microwave fields on calcium efflux from awake cat cerebral cortex. Bioelectromagnetics 3(3):295-307, 1982.

Aitken, R.J., Bennett, L.E., Sawyer, D., Wiklendt, A.M., King, B.V. Impact of radio frequency electromagnetic radiation on DNA integrity in the male germline. Inter J Androl 28:171-179, 2005.

[Akdag, M.Z](https://www.ncbi.nlm.nih.gov/pubmed/?term=Akdag%20MZ%5BAuthor%5D&cauthor=true&cauthor_uid=26775760)., [Dasdag, S](https://www.ncbi.nlm.nih.gov/pubmed/?term=Dasdag%20S%5BAuthor%5D&cauthor=true&cauthor_uid=26775760)., [Canturk, F](https://www.ncbi.nlm.nih.gov/pubmed/?term=Canturk%20F%5BAuthor%5D&cauthor=true&cauthor_uid=26775760)., [Karabulut, D](https://www.ncbi.nlm.nih.gov/pubmed/?term=Karabulut%20D%5BAuthor%5D&cauthor=true&cauthor_uid=26775760)., [Caner, Y](https://www.ncbi.nlm.nih.gov/pubmed/?term=Caner%20Y%5BAuthor%5D&cauthor=true&cauthor_uid=26775760)., [Adalier, N](https://www.ncbi.nlm.nih.gov/pubmed/?term=Adalier%20N%5BAuthor%5D&cauthor=true&cauthor_uid=26775760). Does prolonged radiofrequency radiation emitted from Wi-Fi devices induce DNA damage in various tissues of rats? [J Chem Neuroanat.](https://www.ncbi.nlm.nih.gov/pubmed/26775760) 75(Pt B):116-122, 2016.

Alchalabi ASH, Aklilu E, Aziz AR, Malek F, Ronald SH, Khan MA. Different periods of intrauterine exposure to electromagnetic field: Influence on female rats' fertility, prenatal and postnatal development. Asian Pacific Journal of Reproduction 5: 14-23, 2016.

[Alkis, M.S](https://www.ncbi.nlm.nih.gov/pubmed/?term=Alkis%252520ME%25255BAuthor%25255D&cauthor=true&cauthor_uid=30669883)., [Bilgin, H.M](https://www.ncbi.nlm.nih.gov/pubmed/?term=Bilgin%252520HM%25255BAuthor%25255D&cauthor=true&cauthor_uid=30669883)., [Akpolat, V](https://www.ncbi.nlm.nih.gov/pubmed/?term=Akpolat%252520V%25255BAuthor%25255D&cauthor=true&cauthor_uid=30669883)., [Dasdag, S](https://www.ncbi.nlm.nih.gov/pubmed/?term=Dasdag%252520S%25255BAuthor%25255D&cauthor=true&cauthor_uid=30669883)., [Yegin, K](https://www.ncbi.nlm.nih.gov/pubmed/?term=Yegin%252520K%25255BAuthor%25255D&cauthor=true&cauthor_uid=30669883)., [Yavas, M.C](https://www.ncbi.nlm.nih.gov/pubmed/?term=Yavas%252520MC%25255BAuthor%25255D&cauthor=true&cauthor_uid=30669883)., [Akdag, M.Z](https://www.ncbi.nlm.nih.gov/pubmed/?term=Akdag%252520MZ%25255BAuthor%25255D&cauthor=true&cauthor_uid=30669883). Effect of 900-, 1800-, and 2100-MHz radiofrequency radiation on DNA and oxidative stress in brain. [Electromagn Biol Med.](https://www.ncbi.nlm.nih.gov/pubmed/30669883) 38:32-47, 2019a.

Alkis, M.S., Akdag, M.Z., Dasdag, S., Yegin, K., Akpolat, V. Single-strand DNA breaks and oxidative changes in rat testes exposed to radiofrequency radiation emitted from cellular phones. Biotech Biotech Equip. 33:1, 1733-1740, 2019b.

[Amiri](https://pubmed.ncbi.nlm.nih.gov/?sort=date&term=Amiri+H&cauthor_id=36731647) [H](https://pubmed.ncbi.nlm.nih.gov/36731647/#affiliation-1), [Shabkhiz](https://pubmed.ncbi.nlm.nih.gov/?sort=date&term=Shabkhiz+F&cauthor_id=36731647) [F](https://pubmed.ncbi.nlm.nih.gov/36731647/#affiliation-2),  [Pournemati](https://pubmed.ncbi.nlm.nih.gov/?sort=date&term=Pournemati+P&cauthor_id=36731647) [P](https://pubmed.ncbi.nlm.nih.gov/36731647/#affiliation-3),  [Quchan](https://pubmed.ncbi.nlm.nih.gov/?sort=date&term=Saffar+Kohneh+Quchan+AH&cauthor_id=36731647) [AHSK](https://pubmed.ncbi.nlm.nih.gov/36731647/#affiliation-1),  [Fard](https://pubmed.ncbi.nlm.nih.gov/?sort=date&term=Zeighami+Fard+R&cauthor_id=36731647) RZ. Swimming exercise reduces oxidative stress and liver damage indices of male rats exposed to electromagnetic radiation. Life Sci 317:121461, 2023.

[Arendash, G.W](http://www.ncbi.nlm.nih.gov/pubmed?term=Arendash%20GW%5BAuthor%5D&cauthor=true&cauthor_uid=20061638)., [Sanchez-Ramos, J](http://www.ncbi.nlm.nih.gov/pubmed?term=Sanchez-Ramos%20J%5BAuthor%5D&cauthor=true&cauthor_uid=20061638)., [Mori, T](http://www.ncbi.nlm.nih.gov/pubmed?term=Mori%20T%5BAuthor%5D&cauthor=true&cauthor_uid=20061638)., [Mamcarz, M](http://www.ncbi.nlm.nih.gov/pubmed?term=Mamcarz%20M%5BAuthor%5D&cauthor=true&cauthor_uid=20061638)., [Lin, X](http://www.ncbi.nlm.nih.gov/pubmed?term=Lin%20X%5BAuthor%5D&cauthor=true&cauthor_uid=20061638)., [Runfeldt, M](http://www.ncbi.nlm.nih.gov/pubmed?term=Runfeldt%20M%5BAuthor%5D&cauthor=true&cauthor_uid=20061638)., [Wang, L](http://www.ncbi.nlm.nih.gov/pubmed?term=Wang%20L%5BAuthor%5D&cauthor=true&cauthor_uid=20061638)., [Zhang, G](http://www.ncbi.nlm.nih.gov/pubmed?term=Zhang%20G%5BAuthor%5D&cauthor=true&cauthor_uid=20061638)., [Sava, V](http://www.ncbi.nlm.nih.gov/pubmed?term=Sava%20V%5BAuthor%5D&cauthor=true&cauthor_uid=20061638)., [Tan, J](http://www.ncbi.nlm.nih.gov/pubmed?term=Tan%20J%5BAuthor%5D&cauthor=true&cauthor_uid=20061638)., [Cao, C](http://www.ncbi.nlm.nih.gov/pubmed?term=Cao%20C%5BAuthor%5D&cauthor=true&cauthor_uid=20061638). Electromagnetic field treatment protects against and reverses cognitive impairment in Alzheimer's disease mice. [J Alzheimers Dis.](http://www.ncbi.nlm.nih.gov/pubmed/20061638) 19:191-210, 2010.

Arslan B, Aras N, Yaman S, Comelekoglu U. Investigation of genetic stress parameters in brain tissues of rats exposed to 1.8 GHz cell phone radiofrequency electromagnetic field. Med Science. 13(1):78-82, 2024.

Atasoy, H.I., Gunal, M.Y., Atasoy, P., Elgun, S., Bugdayci, G. Immunohistopathologic demonstration of deleterious effects on growing rat testes of radiofrequency waves emitted from conventional Wi-Fi devices. J Pediatr Urol. 9:223-229, 2013.

Aynali G, Nazıroğlu M, Celik O, Doğan M, Yarıktaş M, Yasan H. Modulation of wireless (2.45 GHz)-induced oxidative toxicity in laryngotracheal mucosa of rat by melatonin. Eur Arch Otorhinolaryngol. 270(5):1695-1700, 2013.

[Bakacak M](http://www.ncbi.nlm.nih.gov/pubmed/?term=Bakacak%20M%5BAuthor%5D&cauthor=true&cauthor_uid=26043407), [Bostancı MS](http://www.ncbi.nlm.nih.gov/pubmed/?term=Bostanc%C4%B1%20MS%5BAuthor%5D&cauthor=true&cauthor_uid=26043407), [Attar R](http://www.ncbi.nlm.nih.gov/pubmed/?term=Attar%20R%5BAuthor%5D&cauthor=true&cauthor_uid=26043407), [Yıldırım ÖK](http://www.ncbi.nlm.nih.gov/pubmed/?term=Y%C4%B1ld%C4%B1r%C4%B1m%20%C3%96K%5BAuthor%5D&cauthor=true&cauthor_uid=26043407), [Yıldırım G](http://www.ncbi.nlm.nih.gov/pubmed/?term=Y%C4%B1ld%C4%B1r%C4%B1m%20G%5BAuthor%5D&cauthor=true&cauthor_uid=26043407), [Bakacak Z](http://www.ncbi.nlm.nih.gov/pubmed/?term=Bakacak%20Z%5BAuthor%5D&cauthor=true&cauthor_uid=26043407), [Sayar H](http://www.ncbi.nlm.nih.gov/pubmed/?term=Sayar%20H%5BAuthor%5D&cauthor=true&cauthor_uid=26043407), [Han A](http://www.ncbi.nlm.nih.gov/pubmed/?term=Han%20A%5BAuthor%5D&cauthor=true&cauthor_uid=26043407). The effects of electromagnetic fields on the number of ovarian primordial follicles: An experimental study. [Kaohsiung J Med Sci.](http://www.ncbi.nlm.nih.gov/pubmed/26043407?dopt=Abstract) 31(6):287-292, 2015.

[Barteri M](http://www.ncbi.nlm.nih.gov/pubmed/?term=Barteri%20M%5BAuthor%5D&cauthor=true&cauthor_uid=25577980), [De Carolis R](http://www.ncbi.nlm.nih.gov/pubmed/?term=De%20Carolis%20R%5BAuthor%5D&cauthor=true&cauthor_uid=25577980), [Marinelli F](http://www.ncbi.nlm.nih.gov/pubmed/?term=Marinelli%20F%5BAuthor%5D&cauthor=true&cauthor_uid=25577980), [Tomassetti G](http://www.ncbi.nlm.nih.gov/pubmed/?term=Tomassetti%20G%5BAuthor%5D&cauthor=true&cauthor_uid=25577980), [Montemiglio LC](http://www.ncbi.nlm.nih.gov/pubmed/?term=Montemiglio%20LC%5BAuthor%5D&cauthor=true&cauthor_uid=25577980). Effects of microwaves (900 MHz) on peroxidase systems: a comparison between lactoperoxidase and horseradish peroxidase. [Electromagn Biol Med.](http://www.ncbi.nlm.nih.gov/pubmed/25577980) 35(2):126-133, 2016.

[Bedir, R](https://www.ncbi.nlm.nih.gov/pubmed/?term=Bedir%252520R%25255BAuthor%25255D&cauthor=true&cauthor_uid=30600117)., [Tumkaya, L](https://www.ncbi.nlm.nih.gov/pubmed/?term=Tumkaya%252520L%25255BAuthor%25255D&cauthor=true&cauthor_uid=30600117)., [Mercantepe, T](https://www.ncbi.nlm.nih.gov/pubmed/?term=Mercantepe%252520T%25255BAuthor%25255D&cauthor=true&cauthor_uid=30600117)., [Yilmaz, A](https://www.ncbi.nlm.nih.gov/pubmed/?term=Yilmaz%252520A%25255BAuthor%25255D&cauthor=true&cauthor_uid=30600117). Pathological findings observed in the kidneys of postnatal male rats exposed to the 2100 MHz electromagnetic field. [Arch Med Res.](https://www.ncbi.nlm.nih.gov/pubmed/?term=bedir+and+2100+MHz) 49:432-440, 2018.

[Bektas](https://pubmed.ncbi.nlm.nih.gov/?sort=date&term=Bektas+H&cauthor_id=36220504) [H](https://pubmed.ncbi.nlm.nih.gov/36220504/#affiliation-1),  [Algul](https://pubmed.ncbi.nlm.nih.gov/?sort=date&term=Algul+S&cauthor_id=36220504) [S](https://pubmed.ncbi.nlm.nih.gov/36220504/#affiliation-2),  [Altindag](https://pubmed.ncbi.nlm.nih.gov/?sort=date&term=Altindag+F&cauthor_id=36220504) [F](https://pubmed.ncbi.nlm.nih.gov/36220504/#affiliation-3),  [Yegin](https://pubmed.ncbi.nlm.nih.gov/?sort=date&term=Yegin+K&cauthor_id=36220504) [K](https://pubmed.ncbi.nlm.nih.gov/36220504/#affiliation-4),  [Akdag](https://pubmed.ncbi.nlm.nih.gov/?sort=date&term=Akdag+Z&cauthor_id=36220504) [Z](https://pubmed.ncbi.nlm.nih.gov/36220504/#affiliation-5),  [Dasdag](https://pubmed.ncbi.nlm.nih.gov/?sort=date&term=Dasdag+S&cauthor_id=36220504) [S.](https://pubmed.ncbi.nlm.nih.gov/36220504/#affiliation-6) Effects of 3.5 GHz (5G) Radiofrequency Radiation on Ghrelin, Nesfatin-1, and Irisin Levels in Diabetic and Healthy Brains. J Chem Neuroanat 126:102168, 2022.

[Bektas](https://pubmed.ncbi.nlm.nih.gov/?sort=date&term=Bektas+H&cauthor_id=36794487) [H](https://pubmed.ncbi.nlm.nih.gov/36794487/#affiliation-1), [Nalbant](https://pubmed.ncbi.nlm.nih.gov/?sort=date&term=Nalbant+A&cauthor_id=36794487) [A](https://pubmed.ncbi.nlm.nih.gov/36794487/#affiliation-2),  [Akdag](https://pubmed.ncbi.nlm.nih.gov/?sort=date&term=Akdag+MB&cauthor_id=36794487) [MB](https://pubmed.ncbi.nlm.nih.gov/36794487/#affiliation-3),  [Demir](https://pubmed.ncbi.nlm.nih.gov/?sort=date&term=Demir+C&cauthor_id=36794487) [C](https://pubmed.ncbi.nlm.nih.gov/36794487/#affiliation-4),  [Kavak](https://pubmed.ncbi.nlm.nih.gov/?sort=date&term=Kavak+S&cauthor_id=36794487) [S](https://pubmed.ncbi.nlm.nih.gov/36794487/#affiliation-5),  [Dasdag](https://pubmed.ncbi.nlm.nih.gov/?sort=date&term=Dasdag+S&cauthor_id=36794487) [S.](https://pubmed.ncbi.nlm.nih.gov/36794487/#affiliation-6) Adverse effects of 900, 1800 and 2100 MHz radiofrequency radiation emitted from mobile phones on bone and skeletal muscle. Electromagn Biol Med 42(1):12-20, 2023.

[Bektas](https://pubmed.ncbi.nlm.nih.gov/?sort=date&term=Bektas+H&cauthor_id=38369591) [H](https://pubmed.ncbi.nlm.nih.gov/38369591/#full-view-affiliation-1),  [Dasdag](https://pubmed.ncbi.nlm.nih.gov/?sort=date&term=Dasdag+S&cauthor_id=38369591) [S](https://pubmed.ncbi.nlm.nih.gov/38369591/#full-view-affiliation-2),  [Altindag](https://pubmed.ncbi.nlm.nih.gov/?sort=date&term=Altindag+F&cauthor_id=38369591) [F](https://pubmed.ncbi.nlm.nih.gov/38369591/#full-view-affiliation-3),  [Akdag](https://pubmed.ncbi.nlm.nih.gov/?sort=date&term=Akdag+MZ&cauthor_id=38369591) [MZ](https://pubmed.ncbi.nlm.nih.gov/38369591/#full-view-affiliation-4),  [Yegin](https://pubmed.ncbi.nlm.nih.gov/?sort=date&term=Yegin+K&cauthor_id=38369591) [K](https://pubmed.ncbi.nlm.nih.gov/38369591/#full-view-affiliation-5),  [Algul](https://pubmed.ncbi.nlm.nih.gov/?sort=date&term=Algul+S&cauthor_id=38369591) [S.](https://pubmed.ncbi.nlm.nih.gov/38369591/#full-view-affiliation-6) Effects of 3.5-GHz radiofrequency radiation on energy-regulatory hormone levels in the blood and adipose tissue. Bioelectromagnetics 45:209-217, 2024.

Belyaev, I.Y., Hillert, L., Protopopova, M., Tamm, C., Malmgren, L.O., Persson, B.R., Selivanova, G., Harms-Ringdahl, M. 915 MHz microwaves and 50 Hz magnetic field affect chromatin conformation and 53BP1 foci in human lymphocytes from hypersensitive and healthy persons. Bioelectromagnetics. 26:173-184, 2005.

[Belyaev, I.Y](http://www.ncbi.nlm.nih.gov/pubmed?term=%252522Belyaev%252520IY%252522%25255BAuthor%25255D&itool=EntrezSystem2.PEntrez.Pubmed.Pubmed_ResultsPanel.Pubmed_RVAbstract)., [Markovà, E](http://www.ncbi.nlm.nih.gov/pubmed?term=%252522Markov%2525C3%2525A0%252520E%252522%25255BAuthor%25255D&itool=EntrezSystem2.PEntrez.Pubmed.Pubmed_ResultsPanel.Pubmed_RVAbstract)., [Hillert, L](http://www.ncbi.nlm.nih.gov/pubmed?term=%252522Hillert%252520L%252522%25255BAuthor%25255D&itool=EntrezSystem2.PEntrez.Pubmed.Pubmed_ResultsPanel.Pubmed_RVAbstract)., [Malmgren, L.O](http://www.ncbi.nlm.nih.gov/pubmed?term=%252522Malmgren%252520LO%252522%25255BAuthor%25255D&itool=EntrezSystem2.PEntrez.Pubmed.Pubmed_ResultsPanel.Pubmed_RVAbstract)., [Persson, B.R](http://www.ncbi.nlm.nih.gov/pubmed?term=%252522Persson%252520BR%252522%25255BAuthor%25255D&itool=EntrezSystem2.PEntrez.Pubmed.Pubmed_ResultsPanel.Pubmed_RVAbstract). Microwaves from UMTS/GSM mobile phones induce long-lasting inhibition of 53BP1/gamma-H2AX DNA repair foci in human lymphocytes. Bioelectromagnetics. 30:129-141, 2009.

[Bilgici](https://pubmed.ncbi.nlm.nih.gov/?sort=date&term=Bilgici+B&cauthor_id=30028652) [B](https://pubmed.ncbi.nlm.nih.gov/30028652/#affiliation-1), [Gun](https://pubmed.ncbi.nlm.nih.gov/?sort=date&term=Gun+S&cauthor_id=30028652) [S](https://pubmed.ncbi.nlm.nih.gov/30028652/#affiliation-2),  [Avci](https://pubmed.ncbi.nlm.nih.gov/?sort=date&term=Avci+B&cauthor_id=30028652) [B](https://pubmed.ncbi.nlm.nih.gov/30028652/#affiliation-1),  [Akar](https://pubmed.ncbi.nlm.nih.gov/?sort=date&term=Akar+A&cauthor_id=30028652) [A](https://pubmed.ncbi.nlm.nih.gov/30028652/#affiliation-3),  [Engiz](https://pubmed.ncbi.nlm.nih.gov/?sort=date&term=K+Engiz+B&cauthor_id=30028652) [BK.](https://pubmed.ncbi.nlm.nih.gov/30028652/#affiliation-4) What is adverse effect of wireless local area network, using 2.45 GHz, on the reproductive system? Int J Radiat Biol 94(11):1054-1061, 2018.

[Blackman, C.F](http://www.ncbi.nlm.nih.gov/pubmed?term=%22Blackman%20CF%22%5BAuthor%5D)., [Benane, S.G](http://www.ncbi.nlm.nih.gov/pubmed?term=%22Benane%20SG%22%5BAuthor%5D)., [Joines, W.T](http://www.ncbi.nlm.nih.gov/pubmed?term=%22Joines%20WT%22%5BAuthor%5D)., [Hollis, M.A](http://www.ncbi.nlm.nih.gov/pubmed?term=%22Hollis%20MA%22%5BAuthor%5D)., [House, D.E](http://www.ncbi.nlm.nih.gov/pubmed?term=%22House%20DE%22%5BAuthor%5D). Calcium-ion efflux from brain tissue: power-density versus internal field-intensity dependencies at 50-MHz RF radiation. [Bioelectromagnetics.](javascript:AL_get(this,%20'jour',%20%0d%0a'Bioelectromagnetics.');) 1**:** 277-283, 1980.

[Blackman](https://pubmed.ncbi.nlm.nih.gov/?sort=date&term=Blackman+CF&cauthor_id=2540755), [C.F](https://pubmed.ncbi.nlm.nih.gov/2540755/#affiliation-1).,  [Kinney](https://pubmed.ncbi.nlm.nih.gov/?sort=date&term=Kinney+LS&cauthor_id=2540755), L.S.,  [House](https://pubmed.ncbi.nlm.nih.gov/?sort=date&term=House+DE&cauthor_id=2540755), D.E.,  [Joines](https://pubmed.ncbi.nlm.nih.gov/?sort=date&term=Joines+WT&cauthor_id=2540755), W.T. Multiple power-density windows and their possible origin. Bioelectromagnetics 10:115-128, 1989.

Bodera P, Stankiewicz W, Antkowiak B, Paluch M, Kieliszek J, Sobiech J, Niemcewicz M. Influence of electromagnetic field (1800 MHz) on lipid peroxidation in brain, blood, liver and kidney in rats. Int J Occup Med Environ Health. 28(4):751-759, 2015.

[Bodin](https://pubmed.ncbi.nlm.nih.gov/?sort=date&term=Bodin+R&cauthor_id=37851267) [R](https://pubmed.ncbi.nlm.nih.gov/37851267/#full-view-affiliation-1),  [Seewooruttun](https://pubmed.ncbi.nlm.nih.gov/?sort=date&term=Seewooruttun+C&cauthor_id=37851267) [C](https://pubmed.ncbi.nlm.nih.gov/37851267/#full-view-affiliation-2),  [Corona](https://pubmed.ncbi.nlm.nih.gov/?sort=date&term=Corona+A&cauthor_id=37851267) [A](https://pubmed.ncbi.nlm.nih.gov/37851267/#full-view-affiliation-2),  [Delanaud](https://pubmed.ncbi.nlm.nih.gov/?sort=date&term=Delanaud+S&cauthor_id=37851267) [S](https://pubmed.ncbi.nlm.nih.gov/37851267/#full-view-affiliation-2),  [Pelletier](https://pubmed.ncbi.nlm.nih.gov/?sort=date&term=Pelletier+A&cauthor_id=37851267) [A](https://pubmed.ncbi.nlm.nih.gov/37851267/#full-view-affiliation-2),  [Villégier](https://pubmed.ncbi.nlm.nih.gov/?sort=date&term=Vill%C3%A9gier+AS&cauthor_id=37851267) [A.](https://pubmed.ncbi.nlm.nih.gov/37851267/#full-view-affiliation-3) Sex-dependent impact of perinatal 5G electromagnetic field exposure in the adolescent rat behavior. Environ Sci Pollut Res Int 30(53):113704-113717, 2023.

Bodin R, Godin L, Mougin C, Lecomte A, Larrigaldie V, Feat-Vetel J, Méresse S, Montécot-Dubourg C, Marcelo P, Mortaud S, Villegier AS. Altered development in rodent brain cells after 900MHz radiofrequency exposure. Neurotoxicology. 2025 Sep 2:103312.

[Bourdineaud, J.P.](https://europepmc.org/search?query=AUTH:%252522Jean-Paul%252520Bourdineaud%252522), [Šrut, M](https://www.ncbi.nlm.nih.gov/pubmed/?term=%2525C5%2525A0rut%252520M%25255BAuthor%25255D&cauthor=true&cauthor_uid=28665795)., [Štambuk, A](https://www.ncbi.nlm.nih.gov/pubmed/?term=%2525C5%2525A0tambuk%252520A%25255BAuthor%25255D&cauthor=true&cauthor_uid=28665795)., [Tkalec, M](https://www.ncbi.nlm.nih.gov/pubmed/?term=Tkalec%252520M%25255BAuthor%25255D&cauthor=true&cauthor_uid=28665795)., [Brèthes, D](https://www.ncbi.nlm.nih.gov/pubmed/?term=Br%2525C3%2525A8thes%252520D%25255BAuthor%25255D&cauthor=true&cauthor_uid=28665795)., [Malarić, K](https://www.ncbi.nlm.nih.gov/pubmed/?term=Malari%2525C4%252587%252520K%25255BAuthor%25255D&cauthor=true&cauthor_uid=28665795)., [Klobučar, G.I.V](https://www.ncbi.nlm.nih.gov/pubmed/?term=Klobu%2525C4%25258Dar%252520GIV%25255BAuthor%25255D&cauthor=true&cauthor_uid=28665795). Electromagnetic fields at a mobile phone frequency (900 MHz) trigger the onset of general stress response along with DNA modifications in Eisenia fetida earthworms. [Arh Hig Rada Toksikol.](https://www.ncbi.nlm.nih.gov/pubmed/28665795) 68:142-152, 2017.

[Bozok](https://pubmed.ncbi.nlm.nih.gov/?sort=date&term=Bozok+S&cauthor_id=36383165) [S](https://pubmed.ncbi.nlm.nih.gov/36383165/#affiliation-1),  [Karaagac](https://pubmed.ncbi.nlm.nih.gov/?sort=date&term=Karaagac+E&cauthor_id=36383165) [E](https://pubmed.ncbi.nlm.nih.gov/36383165/#affiliation-2),  [Sener](https://pubmed.ncbi.nlm.nih.gov/?sort=date&term=Sener+D&cauthor_id=36383165) [D](https://pubmed.ncbi.nlm.nih.gov/36383165/#affiliation-3),  [Akakin](https://pubmed.ncbi.nlm.nih.gov/?sort=date&term=Akakin+D&cauthor_id=36383165) [D](https://pubmed.ncbi.nlm.nih.gov/36383165/#affiliation-4), [Tumkaya](https://pubmed.ncbi.nlm.nih.gov/?sort=date&term=Tumkaya+L&cauthor_id=36383165) [L.](https://pubmed.ncbi.nlm.nih.gov/36383165/#affiliation-5) The effects of long-term prenatal exposure to 900, 1800, and 2100 MHz electromagnetic field radiation on myocardial tissue of rats. Toxicol Ind Health 39(1):1-9, 2023.

Burlaka A, Tsybulin O, Sidorik E, Lukin S, Polishuk V, Tsehmistrenko S, Yakymenko I. Overproduction of free radical species in embryonal cells exposed to low intensity radiofrequency radiation. Exp Oncol. 35(3):219-225, 2013.

[Calis](https://pubmed.ncbi.nlm.nih.gov/?sort=date&term=Calis+P&cauthor_id=31820670) [P](https://pubmed.ncbi.nlm.nih.gov/31820670/#affiliation-1),  [Seymen](https://pubmed.ncbi.nlm.nih.gov/?sort=date&term=Seymen+M&cauthor_id=31820670) [M](https://pubmed.ncbi.nlm.nih.gov/31820670/#affiliation-2),  [Soykan](https://pubmed.ncbi.nlm.nih.gov/?sort=date&term=Soykan+Y&cauthor_id=31820670) [Y](https://pubmed.ncbi.nlm.nih.gov/31820670/#affiliation-3),  [Delen](https://pubmed.ncbi.nlm.nih.gov/?sort=date&term=Delen+K&cauthor_id=31820670) [K](https://pubmed.ncbi.nlm.nih.gov/31820670/#affiliation-4),  [SiravAral](https://pubmed.ncbi.nlm.nih.gov/?sort=date&term=Aral+BS&cauthor_id=31820670) [B](https://pubmed.ncbi.nlm.nih.gov/31820670/#affiliation-4),  [Kaplanoglu](https://pubmed.ncbi.nlm.nih.gov/?sort=date&term=Take+Kaplanoglu+G&cauthor_id=31820670) [GT](https://pubmed.ncbi.nlm.nih.gov/31820670/#affiliation-2), [Karcaaltincaba](https://pubmed.ncbi.nlm.nih.gov/?sort=date&term=Karcaaltincaba+D&cauthor_id=31820670) [D](https://pubmed.ncbi.nlm.nih.gov/31820670/#affiliation-1). Does exposure of smart phones during pregnancy affect the offspring's ovarian reserve? A Rat Model Study. Fetal Pediatr Pathol 40(2):142-152, 2021.

[Cantu](https://pubmed.ncbi.nlm.nih.gov/?sort=date&term=Cantu+JC&cauthor_id=36999566) [JC](https://pubmed.ncbi.nlm.nih.gov/36999566/#affiliation-1),  [Butterworth](https://pubmed.ncbi.nlm.nih.gov/?sort=date&term=Butterworth+JW&cauthor_id=36999566) [JW](https://pubmed.ncbi.nlm.nih.gov/36999566/#affiliation-1),  [Peralta](https://pubmed.ncbi.nlm.nih.gov/?sort=date&term=Peralta+XG&cauthor_id=36999566) [XG](https://pubmed.ncbi.nlm.nih.gov/36999566/#affiliation-2),  [Payne](https://pubmed.ncbi.nlm.nih.gov/?sort=date&term=Payne+JA&cauthor_id=36999566) [JA](https://pubmed.ncbi.nlm.nih.gov/36999566/#affiliation-3),  [Echchgadda](https://pubmed.ncbi.nlm.nih.gov/?sort=date&term=Echchgadda+I&cauthor_id=36999566) [I.](https://pubmed.ncbi.nlm.nih.gov/36999566/#affiliation-3) Analysis of global DNA methylation changes in human keratinocytes immediately following exposure to a 900 MHz radiofrequency field. Bioelectromagnetics 44(3-4):77-89, 2023.

[Cao H](http://www.ncbi.nlm.nih.gov/pubmed/?term=Cao%20H%5BAuthor%5D&cauthor=true&cauthor_uid=25685954), [Qin F](http://www.ncbi.nlm.nih.gov/pubmed/?term=Qin%20F%5BAuthor%5D&cauthor=true&cauthor_uid=25685954), [Liu X](http://www.ncbi.nlm.nih.gov/pubmed/?term=Liu%20X%5BAuthor%5D&cauthor=true&cauthor_uid=25685954), [Wang J](http://www.ncbi.nlm.nih.gov/pubmed/?term=Wang%20J%5BAuthor%5D&cauthor=true&cauthor_uid=25685954), [Cao Y](http://www.ncbi.nlm.nih.gov/pubmed/?term=Cao%20Y%5BAuthor%5D&cauthor=true&cauthor_uid=25685954), [Tong J](http://www.ncbi.nlm.nih.gov/pubmed/?term=Tong%20J%5BAuthor%5D&cauthor=true&cauthor_uid=25685954), [Zhao H](http://www.ncbi.nlm.nih.gov/pubmed/?term=Zhao%20H%5BAuthor%5D&cauthor=true&cauthor_uid=25685954). Circadian Rhythmicity of Antioxidant Markers in Rats Exposed to 1.8 GHz Radiofrequency Fields. [Int J Environ Res Public Health.](http://www.ncbi.nlm.nih.gov/pubmed/25685954?dopt=Abstract) 12(2):2071-2087, 2015.

[Cappucci](https://pubmed.ncbi.nlm.nih.gov/?sort=date&term=Cappucci+U&cauthor_id=36552798), U., [Assunta Maria Casale](https://pubmed.ncbi.nlm.nih.gov/?sort=date&term=Casale+AM&cauthor_id=36552798), [Mirena Proietti](https://pubmed.ncbi.nlm.nih.gov/?sort=date&term=Proietti+M&cauthor_id=36552798), [Fiorenzo Marinelli](https://pubmed.ncbi.nlm.nih.gov/?sort=date&term=Marinelli+F&cauthor_id=36552798), [Livio Giuliani](https://pubmed.ncbi.nlm.nih.gov/?sort=date&term=Giuliani+L&cauthor_id=36552798), [Lucia Piacentini](https://pubmed.ncbi.nlm.nih.gov/?sort=date&term=Piacentini+L&cauthor_id=36552798). WiFi Related Radiofrequency Electromagnetic Fields Promote Transposable Element Dysregulation and Genomic Instability in *Drosophila melanogaster.* Cells 11(24):4036, 2022.

Capri, M., Scarcella, E., Fumelli, C., Bianchi,. S., Mesirca, P., Agostini, C., Antolini, A., Schiavoni, A., Castellani, G., Bersani, F., Franceschi, C. In vitro exposure of human lymphocytes to 900 MHz CW and GSM modulated radiofrequency: studies of proliferation, apoptosis and mitochondrial membrane potential. Radiat Res. 162:211-218, 2004.

Čermak AMM, Ilić K, Pavičić I. Microtubular structure impairment after GSM-modulated RF radiation exposure. Arh Hig Rada Toksikol. 71:205-210, 2020.

Cetin H, Nazıroğlu M, Celik O, Yüksel M, Pastacı N, Ozkaya MO. Liver antioxidant stores protect the brain from electromagnetic radiation (900 and 1800 MHz)-induced oxidative stress in rats during pregnancy and the development of offspring. J Matern Fetal Neonatal Med. 27(18):1915-1921, 2014.

[Ceyhan AM](http://www.ncbi.nlm.nih.gov/pubmed?term=Ceyhan%20AM%5BAuthor%5D&cauthor=true&cauthor_uid=22237725), [Akkaya VB](http://www.ncbi.nlm.nih.gov/pubmed?term=Akkaya%20VB%5BAuthor%5D&cauthor=true&cauthor_uid=22237725), [Güleçol ŞC](http://www.ncbi.nlm.nih.gov/pubmed?term=G%C3%BCle%C3%A7ol%20%C5%9EC%5BAuthor%5D&cauthor=true&cauthor_uid=22237725), [Ceyhan BM](http://www.ncbi.nlm.nih.gov/pubmed?term=Ceyhan%20BM%5BAuthor%5D&cauthor=true&cauthor_uid=22237725), [Özgüner F](http://www.ncbi.nlm.nih.gov/pubmed?term=%C3%96zg%C3%BCner%20F%5BAuthor%5D&cauthor=true&cauthor_uid=22237725), [Chen W](http://www.ncbi.nlm.nih.gov/pubmed?term=Chen%20W%5BAuthor%5D&cauthor=true&cauthor_uid=22237725). Protective effects of β-glucan against oxidative injury induced by 2.45-GHz electromagnetic radiation in the skin tissue of rats. [Arch Dermatol Res.](http://www.ncbi.nlm.nih.gov/pubmed/22237725) 304(7):521-527, 2012.

[Chandel S](https://www.ncbi.nlm.nih.gov/pubmed/?term=Chandel%20S%5BAuthor%5D&cauthor=true&cauthor_uid=31297205), [Kaur S](https://www.ncbi.nlm.nih.gov/pubmed/?term=Kaur%20S%5BAuthor%5D&cauthor=true&cauthor_uid=31297205), [Issa M](https://www.ncbi.nlm.nih.gov/pubmed/?term=Issa%20M%5BAuthor%5D&cauthor=true&cauthor_uid=31297205), [Singh HP](https://www.ncbi.nlm.nih.gov/pubmed/?term=Singh%20HP%5BAuthor%5D&cauthor=true&cauthor_uid=31297205), [Batish DR](https://www.ncbi.nlm.nih.gov/pubmed/?term=Batish%20DR%5BAuthor%5D&cauthor=true&cauthor_uid=31297205), [Kohli RK](https://www.ncbi.nlm.nih.gov/pubmed/?term=Kohli%20RK%5BAuthor%5D&cauthor=true&cauthor_uid=31297205). Exposure to mobile phone radiations at 2350 MHz incites cyto- and genotoxic effects in root meristems of *Allium cepa*. [J Environ Health Sci Eng.](https://www.ncbi.nlm.nih.gov/pubmed/31297205) 17(1):97-104, 2019a.

[Chandel S](https://www.ncbi.nlm.nih.gov/pubmed/?term=Chandel%20S%5BAuthor%5D&cauthor=true&cauthor_uid=31115694), [Kaur S](https://www.ncbi.nlm.nih.gov/pubmed/?term=Kaur%20S%5BAuthor%5D&cauthor=true&cauthor_uid=31115694), [Issa M](https://www.ncbi.nlm.nih.gov/pubmed/?term=Issa%20M%5BAuthor%5D&cauthor=true&cauthor_uid=31115694), [Singh HP](https://www.ncbi.nlm.nih.gov/pubmed/?term=Singh%20HP%5BAuthor%5D&cauthor=true&cauthor_uid=31115694), [Batish DR](https://www.ncbi.nlm.nih.gov/pubmed/?term=Batish%20DR%5BAuthor%5D&cauthor=true&cauthor_uid=31115694), [Kohli RK](https://www.ncbi.nlm.nih.gov/pubmed/?term=Kohli%20RK%5BAuthor%5D&cauthor=true&cauthor_uid=31115694). Appraisal of immediate and late effects of mobile phone radiations at 2100 MHz on mitotic activity and DNA integrity in root meristems of Allium cepa. [Protoplasma.](https://www.ncbi.nlm.nih.gov/pubmed/31115694) 256(5):1399-1407, 2019b.

Chaturvedi, C.M., Singh, V.P., Singh, P., Basu, P., Singaravel, M., Shukla, R.K., Dhawan ,A., Pati, A.K., Gangwar, R.K., Singh, S.P. 2.45 GHZ (CW) microwave irradiation alters circadian organization, spatial memory, DNA structure in the brain cells and blood cell counts of male mice, Mus musculus. Prog Electromagn Res B. 29:23-42, 2011.

[Chauhan P](https://www.ncbi.nlm.nih.gov/pubmed/?term=Chauhan%20P%5BAuthor%5D&cauthor=true&cauthor_uid=27362544), [Verma HN](https://www.ncbi.nlm.nih.gov/pubmed/?term=Verma%20HN%5BAuthor%5D&cauthor=true&cauthor_uid=27362544), [Sisodia R](https://www.ncbi.nlm.nih.gov/pubmed/?term=Sisodia%20R%5BAuthor%5D&cauthor=true&cauthor_uid=27362544), [Kesari KK](https://www.ncbi.nlm.nih.gov/pubmed/?term=Kesari%20KK%5BAuthor%5D&cauthor=true&cauthor_uid=27362544). Microwave radiation (2.45 GHz)-induced oxidative stress: Whole-body exposure effect on histopathology of Wistar rats. [Electromagn Biol Med.](https://www.ncbi.nlm.nih.gov/pubmed/27362544) 36(1):20-30, 2017.

[Chen L](http://www.ncbi.nlm.nih.gov/pubmed?term=Chen%20L%5BAuthor%5D&cauthor=true&cauthor_uid=24564122), [Qin F](http://www.ncbi.nlm.nih.gov/pubmed?term=Qin%20F%5BAuthor%5D&cauthor=true&cauthor_uid=24564122), [Chen Y](http://www.ncbi.nlm.nih.gov/pubmed?term=Chen%20Y%5BAuthor%5D&cauthor=true&cauthor_uid=24564122), [Sun J](http://www.ncbi.nlm.nih.gov/pubmed?term=Sun%20J%5BAuthor%5D&cauthor=true&cauthor_uid=24564122), [Tong J](http://www.ncbi.nlm.nih.gov/pubmed?term=Tong%20J%5BAuthor%5D&cauthor=true&cauthor_uid=24564122).[Chronotoxicity of 1800 MHz microwave radiation on sex hormones and spermatogenesis in male mice]. [Wei Sheng Yan Jiu.](http://www.ncbi.nlm.nih.gov/pubmed/24564122) 43(1):110-115, 2014.[Article in Chinese]

[Comelekoglu](https://pubmed.ncbi.nlm.nih.gov/?sort=pubdate&term=Comelekoglu+U&cauthor_id=30328127), [U](https://pubmed.ncbi.nlm.nih.gov/30328127/#affiliation-1)., [Aktas](https://pubmed.ncbi.nlm.nih.gov/?sort=pubdate&term=Aktas+S&cauthor_id=30328127), [S](https://pubmed.ncbi.nlm.nih.gov/30328127/#affiliation-2)., [Demirbag](https://pubmed.ncbi.nlm.nih.gov/?sort=pubdate&term=Demirbag+B&cauthor_id=30328127), [B](https://pubmed.ncbi.nlm.nih.gov/30328127/#affiliation-2).,  [Karagul](https://pubmed.ncbi.nlm.nih.gov/?sort=pubdate&term=Karagul+MI&cauthor_id=30328127), [M.I](https://pubmed.ncbi.nlm.nih.gov/30328127/#affiliation-2)., [Yalin](https://pubmed.ncbi.nlm.nih.gov/?sort=pubdate&term=Yalin+S&cauthor_id=30328127), [S](https://pubmed.ncbi.nlm.nih.gov/30328127/#affiliation-3)., [Yildirim](https://pubmed.ncbi.nlm.nih.gov/?sort=pubdate&term=Yildirim+M&cauthor_id=30328127), [M](https://pubmed.ncbi.nlm.nih.gov/30328127/#affiliation-3).,  [Akar](https://pubmed.ncbi.nlm.nih.gov/?sort=pubdate&term=Akar+A&cauthor_id=30328127), [A](https://pubmed.ncbi.nlm.nih.gov/30328127/#affiliation-4).,  [Engiz](https://pubmed.ncbi.nlm.nih.gov/?sort=pubdate&term=Korunur+Engiz+B&cauthor_id=30328127), [B.K](https://pubmed.ncbi.nlm.nih.gov/30328127/#affiliation-5)., [Sogut](https://pubmed.ncbi.nlm.nih.gov/?sort=pubdate&term=Sogut+F&cauthor_id=30328127), F., [Ozbay](https://pubmed.ncbi.nlm.nih.gov/?sort=pubdate&term=Ozbay+E&cauthor_id=30328127), E. Effect of low-level 1800 MHz radiofrequency radiation on the rat sciatic nerve and the protective role of paricalcitol. Bioelectromagnetics. 39:631-643, 2018.

[Coskun](https://pubmed.ncbi.nlm.nih.gov/?sort=date&term=Ozergin+Coskun+Z&cauthor_id=39331752) [ZO](https://pubmed.ncbi.nlm.nih.gov/39331752/#full-view-affiliation-1), [Tumkaya](https://pubmed.ncbi.nlm.nih.gov/?sort=date&term=Tumkaya+L&cauthor_id=39331752) [L](https://pubmed.ncbi.nlm.nih.gov/39331752/#full-view-affiliation-2),  [Yilmaz](https://pubmed.ncbi.nlm.nih.gov/?sort=date&term=Yilmaz+A&cauthor_id=39331752) [A](https://pubmed.ncbi.nlm.nih.gov/39331752/#full-view-affiliation-3),  [Dursun](https://pubmed.ncbi.nlm.nih.gov/?sort=date&term=Dursun+E&cauthor_id=39331752) [E](https://pubmed.ncbi.nlm.nih.gov/39331752/#full-view-affiliation-4),  [Mercantepe](https://pubmed.ncbi.nlm.nih.gov/?sort=date&term=Mercantepe+T&cauthor_id=39331752) [T](https://pubmed.ncbi.nlm.nih.gov/39331752/#full-view-affiliation-5),  [Kalkan](https://pubmed.ncbi.nlm.nih.gov/?sort=date&term=Kalkan+Y&cauthor_id=39331752) [Y](https://pubmed.ncbi.nlm.nih.gov/39331752/#full-view-affiliation-6),  [Ersoz](https://pubmed.ncbi.nlm.nih.gov/?sort=date&term=Ersoz+S&cauthor_id=39331752) [S.](https://pubmed.ncbi.nlm.nih.gov/39331752/#full-view-affiliation-7) Does Radiofrequency Radiation From Mobile Phones Affect the Formation of Parotid Gland Malignancy? An Experimental Study. Ear Nose Throat J 103(3_suppl):75S-82S, 2024.

[Dasdag S](http://www.ncbi.nlm.nih.gov/pubmed?term=Dasdag%20S%5BAuthor%5D&cauthor=true&cauthor_uid=24460421), [Taş M](http://www.ncbi.nlm.nih.gov/pubmed?term=Ta%C5%9F%20M%5BAuthor%5D&cauthor=true&cauthor_uid=24460421), [Akdag MZ](http://www.ncbi.nlm.nih.gov/pubmed?term=Akdag%20MZ%5BAuthor%5D&cauthor=true&cauthor_uid=24460421), [Yegin K](http://www.ncbi.nlm.nih.gov/pubmed?term=Yegin%20K%5BAuthor%5D&cauthor=true&cauthor_uid=24460421). Effect of long-term exposure of 2.4 GHz radiofrequency radiation emitted from Wi-Fi equipment on testes functions. [Electromagn Biol Med.](http://www.ncbi.nlm.nih.gov/pubmed/24460421) 34(1):37-42, 2015.

[Delavarifar S](https://pubmed.ncbi.nlm.nih.gov/?sort=date&term=S+D&cauthor_id=32337184), [Razi Z](https://pubmed.ncbi.nlm.nih.gov/?sort=date&term=Z+R&cauthor_id=32337184), [Tamadon A](https://pubmed.ncbi.nlm.nih.gov/?sort=date&term=A+T&cauthor_id=32337184),  [Rahmanifar F](https://pubmed.ncbi.nlm.nih.gov/?sort=date&term=F+R&cauthor_id=32337184), [Mehrabani D](https://pubmed.ncbi.nlm.nih.gov/?sort=date&term=D+M&cauthor_id=32337184), [Owjfard M](https://pubmed.ncbi.nlm.nih.gov/?sort=date&term=M+O&cauthor_id=32337184), [Koohi-Hoseinabadi O](https://pubmed.ncbi.nlm.nih.gov/?sort=date&term=O+KH&cauthor_id=32337184), [Zaker Abasali S](https://pubmed.ncbi.nlm.nih.gov/?sort=date&term=S+ZA&cauthor_id=32337184).  Low-power density radiations emitted from common Wi-Fi routers influence sperm concentration and sperm histomorphometric parameters: A new horizon on male infertility treatment. J Biomed Phys Eng 10(2):167-176, 2020.

[Demirbağ](https://pubmed.ncbi.nlm.nih.gov/?sort=date&term=Demirba%C4%9F+B&cauthor_id=37717340) [B](https://pubmed.ncbi.nlm.nih.gov/37717340/#full-view-affiliation-1), [Aktaş](https://pubmed.ncbi.nlm.nih.gov/?sort=date&term=Akta%C5%9F+S&cauthor_id=37717340) [S](https://pubmed.ncbi.nlm.nih.gov/37717340/#full-view-affiliation-2),  [Çömelekoğlu](https://pubmed.ncbi.nlm.nih.gov/?sort=date&term=%C3%87%C3%B6meleko%C4%9Flu+%C3%9C&cauthor_id=37717340) Ü,  [Kara](https://pubmed.ncbi.nlm.nih.gov/?sort=date&term=Kara+%C4%B0&cauthor_id=37717340) [I](https://pubmed.ncbi.nlm.nih.gov/37717340/#full-view-affiliation-2), [Yildirim](https://pubmed.ncbi.nlm.nih.gov/?sort=date&term=Yildirim+M&cauthor_id=37717340) [M](https://pubmed.ncbi.nlm.nih.gov/37717340/#full-view-affiliation-4),  [Derici Yildirim](https://pubmed.ncbi.nlm.nih.gov/?sort=date&term=Yildirim+DD&cauthor_id=37717340) [DD.](https://pubmed.ncbi.nlm.nih.gov/37717340/#full-view-affiliation-5)

Protective effect of paricalcitol in rat testicular damage induced by subchronic 1800 MHz radiofrequency radiation. Biochem Biophys Res Commun 680:42-50, 2023.

de Pomerai, D.I., Smith, B., Dawe, A., North, K., Smith, T., Archer, D.B., Duce, I.R., Jones, D., Candido, E.P. Microwave radiation can alter protein conformation without bulk heating. FEBS Lett. 543:93-97, 2003.

[Deshmukh, P.S](http://www.ncbi.nlm.nih.gov/pubmed?term=Deshmukh%252520PS%25255BAuthor%25255D&cauthor=true&cauthor_uid=23833433)., [Megha, K](http://www.ncbi.nlm.nih.gov/pubmed?term=Megha%252520K%25255BAuthor%25255D&cauthor=true&cauthor_uid=23833433)., [Banerjee, B.D](http://www.ncbi.nlm.nih.gov/pubmed?term=Banerjee%252520BD%25255BAuthor%25255D&cauthor=true&cauthor_uid=23833433)., [Ahmed, R.S](http://www.ncbi.nlm.nih.gov/pubmed?term=Ahmed%252520RS%25255BAuthor%25255D&cauthor=true&cauthor_uid=23833433)., [Chandn, S](http://www.ncbi.nlm.nih.gov/pubmed?term=Chandna%252520S%25255BAuthor%25255D&cauthor=true&cauthor_uid=23833433)., [Abegaonkar, M.P](http://www.ncbi.nlm.nih.gov/pubmed?term=Abegaonkar%252520MP%25255BAuthor%25255D&cauthor=true&cauthor_uid=23833433)., [Tripath, A.K](http://www.ncbi.nlm.nih.gov/pubmed?term=Tripathi%252520AK%25255BAuthor%25255D&cauthor=true&cauthor_uid=23833433). Detection of low level microwave radiation induced deoxyribonucleic acid damage vis-à-vis genotoxicity in brain of Fischer rats. [Toxicol Int.](http://www.ncbi.nlm.nih.gov/pubmed/23833433) 20:19-24, 2013a.

[Deshmukh PS](http://www.ncbi.nlm.nih.gov/pubmed?term=Deshmukh%20PS%5BAuthor%5D&cauthor=true&cauthor_uid=23720885), [Banerjee BD](http://www.ncbi.nlm.nih.gov/pubmed?term=Banerjee%20BD%5BAuthor%5D&cauthor=true&cauthor_uid=23720885), [Abegaonkar MP](http://www.ncbi.nlm.nih.gov/pubmed?term=Abegaonkar%20MP%5BAuthor%5D&cauthor=true&cauthor_uid=23720885), [Megha K](http://www.ncbi.nlm.nih.gov/pubmed?term=Megha%20K%5BAuthor%5D&cauthor=true&cauthor_uid=23720885), [Ahmed RS](http://www.ncbi.nlm.nih.gov/pubmed?term=Ahmed%20RS%5BAuthor%5D&cauthor=true&cauthor_uid=23720885), [Tripathi AK](http://www.ncbi.nlm.nih.gov/pubmed?term=Tripathi%20AK%5BAuthor%5D&cauthor=true&cauthor_uid=23720885), [Mediratta PK](http://www.ncbi.nlm.nih.gov/pubmed?term=Mediratta%20PK%5BAuthor%5D&cauthor=true&cauthor_uid=23720885). Effect of low level microwave radiation exposure on cognitive function and oxidative stress in rats. [Indian J Biochem Biophys.](http://www.ncbi.nlm.nih.gov/pubmed/23720885) 50(2):114-119, 2013b.

[Deshmukh, P.S](http://www.ncbi.nlm.nih.gov/pubmed/?term=Deshmukh%252520PS%25255BAuthor%25255D&cauthor=true&cauthor_uid=25749756)., [Nasare, N](http://www.ncbi.nlm.nih.gov/pubmed/?term=Nasare%252520N%25255BAuthor%25255D&cauthor=true&cauthor_uid=25749756)., [Megha, K](http://www.ncbi.nlm.nih.gov/pubmed/?term=Megha%252520K%25255BAuthor%25255D&cauthor=true&cauthor_uid=25749756)., [Banerjee, B.D](http://www.ncbi.nlm.nih.gov/pubmed/?term=Banerjee%252520BD%25255BAuthor%25255D&cauthor=true&cauthor_uid=25749756)., [Ahmed, R.S](http://www.ncbi.nlm.nih.gov/pubmed/?term=Ahmed%252520RS%25255BAuthor%25255D&cauthor=true&cauthor_uid=25749756)., [Singh, D](http://www.ncbi.nlm.nih.gov/pubmed/?term=Singh%252520D%25255BAuthor%25255D&cauthor=true&cauthor_uid=25749756)., [Abegaonkar, M.P](http://www.ncbi.nlm.nih.gov/pubmed/?term=Abegaonkar%252520MP%25255BAuthor%25255D&cauthor=true&cauthor_uid=25749756)., [Tripathi, A.K](http://www.ncbi.nlm.nih.gov/pubmed/?term=Tripathi%252520AK%25255BAuthor%25255D&cauthor=true&cauthor_uid=25749756)., [Mediratta, P.K](http://www.ncbi.nlm.nih.gov/pubmed/?term=Mediratta%252520PK%25255BAuthor%25255D&cauthor=true&cauthor_uid=25749756). Cognitive impairment and neurogenotoxic effects in rats exposed to low-Intensity microwave radiation. [Int J Toxicol.](http://www.ncbi.nlm.nih.gov/pubmed/25749756?dopt=Abstract) 34:284-290, 2015.

[Deshmukh, P.S](https://www.ncbi.nlm.nih.gov/pubmed/?term=Deshmukh%252520PS%25255BAuthor%25255D&cauthor=true&cauthor_uid=28081746)., [Megha, K](https://www.ncbi.nlm.nih.gov/pubmed/?term=Megha%252520K%25255BAuthor%25255D&cauthor=true&cauthor_uid=28081746)., [Nasare, N](https://www.ncbi.nlm.nih.gov/pubmed/?term=Nasare%252520N%25255BAuthor%25255D&cauthor=true&cauthor_uid=28081746)., [Banerjee, B.D](https://www.ncbi.nlm.nih.gov/pubmed/?term=Banerjee%252520BD%25255BAuthor%25255D&cauthor=true&cauthor_uid=28081746)., [Ahmed, R.S](https://www.ncbi.nlm.nih.gov/pubmed/?term=Ahmed%252520RS%25255BAuthor%25255D&cauthor=true&cauthor_uid=28081746)., [Abegaonkar, M.P](https://www.ncbi.nlm.nih.gov/pubmed/?term=Abegaonkar%252520MP%25255BAuthor%25255D&cauthor=true&cauthor_uid=28081746)., [Tripathi, A.K](https://www.ncbi.nlm.nih.gov/pubmed/?term=Tripathi%252520AK%25255BAuthor%25255D&cauthor=true&cauthor_uid=28081746)., [Mediratta, P.K](https://www.ncbi.nlm.nih.gov/pubmed/?term=Mediratta%252520PK%25255BAuthor%25255D&cauthor=true&cauthor_uid=28081746). Effect of low level subchronic microwave radiation on rat brain. [Biomed Environ Sci.](https://www.ncbi.nlm.nih.gov/pubmed/28081746) 29:858-867, 2016.

D’Inzeo, G., Bernardi, P., Eusebi, F., Grassi, F., Tamburello, C., Zani, B.M. Microwave effects on acetylcholine-induced channels in cultured chick myo­tubes. Bioelectromagnetics. **9:** 363-372, 1988.

[Djordjevic B](http://www.ncbi.nlm.nih.gov/pubmed/?term=Djordjevic%20B%5BAuthor%5D&cauthor=true&cauthor_uid=25665474), [Sokolovic D](http://www.ncbi.nlm.nih.gov/pubmed/?term=Sokolovic%20D%5BAuthor%5D&cauthor=true&cauthor_uid=25665474), [Kocic G](http://www.ncbi.nlm.nih.gov/pubmed/?term=Kocic%20G%5BAuthor%5D&cauthor=true&cauthor_uid=25665474), [Veljkovic A](http://www.ncbi.nlm.nih.gov/pubmed/?term=Veljkovic%20A%5BAuthor%5D&cauthor=true&cauthor_uid=25665474), [Despotovic M](http://www.ncbi.nlm.nih.gov/pubmed/?term=Despotovic%20M%5BAuthor%5D&cauthor=true&cauthor_uid=25665474), [Basic J](http://www.ncbi.nlm.nih.gov/pubmed/?term=Basic%20J%5BAuthor%5D&cauthor=true&cauthor_uid=25665474), [Jevtovic-Stoimenov T](http://www.ncbi.nlm.nih.gov/pubmed/?term=Jevtovic-Stoimenov%20T%5BAuthor%5D&cauthor=true&cauthor_uid=25665474), [Sokolovic DM](http://www.ncbi.nlm.nih.gov/pubmed/?term=Sokolovic%20DM%5BAuthor%5D&cauthor=true&cauthor_uid=25665474). The effect of melatonin on the liver of rats exposed to microwave radiation. [Bratisl Lek Listy.](http://www.ncbi.nlm.nih.gov/pubmed/25665474?dopt=Abstract) 116(2):96-100, 2015.

[Durdik M](https://www.ncbi.nlm.nih.gov/pubmed/?term=Durdik%20M%5BAuthor%5D&cauthor=true&cauthor_uid=31700008), [Kosik P](https://www.ncbi.nlm.nih.gov/pubmed/?term=Kosik%20P%5BAuthor%5D&cauthor=true&cauthor_uid=31700008), [Markova E](https://www.ncbi.nlm.nih.gov/pubmed/?term=Markova%20E%5BAuthor%5D&cauthor=true&cauthor_uid=31700008), [Somsedikova A](https://www.ncbi.nlm.nih.gov/pubmed/?term=Somsedikova%20A%5BAuthor%5D&cauthor=true&cauthor_uid=31700008), [Gajdosechova B](https://www.ncbi.nlm.nih.gov/pubmed/?term=Gajdosechova%20B%5BAuthor%5D&cauthor=true&cauthor_uid=31700008), [Nikitina E](https://www.ncbi.nlm.nih.gov/pubmed/?term=Nikitina%20E%5BAuthor%5D&cauthor=true&cauthor_uid=31700008), [Horvathova E](https://www.ncbi.nlm.nih.gov/pubmed/?term=Horvathova%20E%5BAuthor%5D&cauthor=true&cauthor_uid=31700008), [Kozics K](https://www.ncbi.nlm.nih.gov/pubmed/?term=Kozics%20K%5BAuthor%5D&cauthor=true&cauthor_uid=31700008), [Davis D](https://www.ncbi.nlm.nih.gov/pubmed/?term=Davis%20D%5BAuthor%5D&cauthor=true&cauthor_uid=31700008), [Belyaev I](https://www.ncbi.nlm.nih.gov/pubmed/?term=Belyaev%20I%5BAuthor%5D&cauthor=true&cauthor_uid=31700008). Microwaves from mobile phone induce reactive oxygen species but not DNA damage, preleukemic fusion genes and apoptosis in hematopoietic stem/progenitor cells. [Sci Rep.](https://www.ncbi.nlm.nih.gov/pubmed/31700008) 9(1):16182, 2019.

[Dutta, S.K](http://www.ncbi.nlm.nih.gov/pubmed?term=%252522Dutta%252520SK%252522%25255BAuthor%25255D)., [Subramoniam, A](http://www.ncbi.nlm.nih.gov/pubmed?term=%252522Subramoniam%252520A%252522%25255BAuthor%25255D)., [Ghosh, B](http://www.ncbi.nlm.nih.gov/pubmed?term=%252522Ghosh%252520B%252522%25255BAuthor%25255D)., [Parshad, R](http://www.ncbi.nlm.nih.gov/pubmed?term=%252522Parshad%252520R%252522%25255BAuthor%25255D). Microwave radiation-induced calcium ion efflux from human neuroblastoma cells in culture. Bioelectromagnetics. 5:71-78, 1984.

Dutta S.K., Ghosh, B., Blackman, C.F. Radiofrequency radiation-induced calcium ion efflux enhancement from human and other neuroblastoma cells in culture. Bioelectromagnetics. 10:197-202, 1989.

Dutta SK, Verma M, Blackman CF, Frequency-dependent alterations in enolase activity in Escherichia coli caused by exposure to electric and magnetic fields. Bioelectromagnetics. 15:377-383, 1994.

[Eberhardt , J.L](http://www.ncbi.nlm.nih.gov/pubmed?term=Eberhardt%20JL%5BAuthor%5D&cauthor=true&cauthor_uid=18821198)., [Persson, B.R](http://www.ncbi.nlm.nih.gov/pubmed?term=Persson%20BR%5BAuthor%5D&cauthor=true&cauthor_uid=18821198)., [Brun, A.E](http://www.ncbi.nlm.nih.gov/pubmed?term=Brun%20AE%5BAuthor%5D&cauthor=true&cauthor_uid=18821198)., [Salford, L.G](http://www.ncbi.nlm.nih.gov/pubmed?term=Salford%20LG%5BAuthor%5D&cauthor=true&cauthor_uid=18821198)., [Malmgren, L.O](http://www.ncbi.nlm.nih.gov/pubmed?term=Malmgren%20LO%5BAuthor%5D&cauthor=true&cauthor_uid=18821198). Blood-brain barrier permeability and nerve cell damage in rat brain 14 and 28 days after exposure to microwaves from GSM mobile phones. [Electromagn Biol Med.](http://www.ncbi.nlm.nih.gov/pubmed/18821198) 27:215-229, 2008.

[Echchgadda](https://pubmed.ncbi.nlm.nih.gov/?sort=date&term=Echchgadda+I&cauthor_id=35241689), [I](https://pubmed.ncbi.nlm.nih.gov/35241689/#affiliation-1).,  [Cantu](https://pubmed.ncbi.nlm.nih.gov/?sort=date&term=Cantu+JC&cauthor_id=35241689), [J.C](https://pubmed.ncbi.nlm.nih.gov/35241689/#affiliation-2).,  [Tolstykh](https://pubmed.ncbi.nlm.nih.gov/?sort=date&term=Tolstykh+GP&cauthor_id=35241689), [G.P](https://pubmed.ncbi.nlm.nih.gov/35241689/#affiliation-2).,  [Butterworth](https://pubmed.ncbi.nlm.nih.gov/?sort=date&term=Butterworth+JW&cauthor_id=35241689), [J.W](https://pubmed.ncbi.nlm.nih.gov/35241689/#affiliation-2).,  [Payne](https://pubmed.ncbi.nlm.nih.gov/?sort=date&term=Payne+JA&cauthor_id=35241689), [J.A](https://pubmed.ncbi.nlm.nih.gov/35241689/#affiliation-3).,  [Ibey](https://pubmed.ncbi.nlm.nih.gov/?sort=date&term=Ibey+BL&cauthor_id=35241689), [B.L.](https://pubmed.ncbi.nlm.nih.gov/35241689/#affiliation-3) Changes in the excitability of primary hippocampal neurons following exposure to 3.0 GHz radiofrequency electromagnetic fields. Sci Rep. 12:3506, 2022.

[Eker, E.D](https://www.ncbi.nlm.nih.gov/pubmed/?term=Eker%252520ED%25255BAuthor%25255D&cauthor=true&cauthor_uid=30226071),, [Arslan, B](https://www.ncbi.nlm.nih.gov/pubmed/?term=Arslan%252520B%25255BAuthor%25255D&cauthor=true&cauthor_uid=30226071)., [Yildirim, M](https://www.ncbi.nlm.nih.gov/pubmed/?term=Yildirim%252520M%25255BAuthor%25255D&cauthor=true&cauthor_uid=30226071)., [Akar, A](https://www.ncbi.nlm.nih.gov/pubmed/?term=Akar%252520A%25255BAuthor%25255D&cauthor=true&cauthor_uid=30226071)., [Aras, N](https://www.ncbi.nlm.nih.gov/pubmed/?term=Aras%252520N%25255BAuthor%25255D&cauthor=true&cauthor_uid=30226071). The effect of exposure to 1800 MHz radiofrequency radiation on epidermal growth factor, caspase-3, Hsp27 and p38MAPK gene expressions in the rat eye. [Bratisl Lek Listy.](https://www.ncbi.nlm.nih.gov/pubmed/30226071) 119:588-592, 2018.

Elekes, E, Thuroczy, G, Szabo, LD, Effect on the immune system of mice exposed chronically to 50 Hz amplitude-modulated 2.45 GHz microwaves. Bioelectromagnetics 17(3):246-248, 1996.

[Er](https://pubmed.ncbi.nlm.nih.gov/?sort=date&term=Er+H&cauthor_id=35015292) H,  [Tas](https://pubmed.ncbi.nlm.nih.gov/?sort=date&term=Tas+GG&cauthor_id=35015292) [GG](https://pubmed.ncbi.nlm.nih.gov/35015292/#affiliation-3),  [Soygur](https://pubmed.ncbi.nlm.nih.gov/?sort=date&term=Soygur+B&cauthor_id=35015292) B,  [Ozen](https://pubmed.ncbi.nlm.nih.gov/?sort=date&term=Ozen+S&cauthor_id=35015292) [S](https://pubmed.ncbi.nlm.nih.gov/35015292/#affiliation-5),  [Sati](https://pubmed.ncbi.nlm.nih.gov/?sort=date&term=Sati+L&cauthor_id=35015292) [L](https://pubmed.ncbi.nlm.nih.gov/35015292/#affiliation-6). Acute and chronic exposure to 900 MHz radio frequency radiation activates p38/JNK-mediated MAPK pathway in rat testis. Reprod Sci 29(5):1471-1485, 2022.

Er H, Basaranlar G., Derin N., Kantar D, Ozen S. The effects of short-term and long-term 2100 MHz radiofrequency radiation on adult rat auditory brainstem response. *Open Chemistry*, *23*(1), 20250173, 2025.

[Ertilav K](https://www.ncbi.nlm.nih.gov/pubmed/?term=Ertilav%20K%5BAuthor%5D&cauthor=true&cauthor_uid=29332300), [Uslusoy F](https://www.ncbi.nlm.nih.gov/pubmed/?term=Uslusoy%20F%5BAuthor%5D&cauthor=true&cauthor_uid=29332300), [Ataizi S](https://www.ncbi.nlm.nih.gov/pubmed/?term=Ataizi%20S%5BAuthor%5D&cauthor=true&cauthor_uid=29332300), [Nazıroğlu M](https://www.ncbi.nlm.nih.gov/pubmed/?term=Naz%C4%B1ro%C4%9Flu%20M%5BAuthor%5D&cauthor=true&cauthor_uid=29332300).Long term exposure to cell phone frequencies (900 and 1800 MHz) induces apoptosis, mitochondrial oxidative stress and TRPV1 channel activation in the hippocampus and dorsal root ganglion of rats. [Metab Brain Dis.](https://www.ncbi.nlm.nih.gov/pubmed/29332300) 33(3):753-763, 2018.

[Esmekaya MA](http://www.ncbi.nlm.nih.gov/pubmed?term=Esmekaya%20MA%5BAuthor%5D&cauthor=true&cauthor_uid=22014767), [Aytekin E](http://www.ncbi.nlm.nih.gov/pubmed?term=Aytekin%20E%5BAuthor%5D&cauthor=true&cauthor_uid=22014767), [Ozgur E](http://www.ncbi.nlm.nih.gov/pubmed?term=Ozgur%20E%5BAuthor%5D&cauthor=true&cauthor_uid=22014767), [Güler G](http://www.ncbi.nlm.nih.gov/pubmed?term=G%C3%BCler%20G%5BAuthor%5D&cauthor=true&cauthor_uid=22014767), [Ergun MA](http://www.ncbi.nlm.nih.gov/pubmed?term=Ergun%20MA%5BAuthor%5D&cauthor=true&cauthor_uid=22014767), [Omeroğlu S](http://www.ncbi.nlm.nih.gov/pubmed?term=Omero%C4%9Flu%20S%5BAuthor%5D&cauthor=true&cauthor_uid=22014767), [Seyhan N](http://www.ncbi.nlm.nih.gov/pubmed?term=Seyhan%20N%5BAuthor%5D&cauthor=true&cauthor_uid=22014767). Mutagenic and morphologic impacts of 1.8GHz radiofrequency radiation on human peripheral blood lymphocytes (hPBLs) and possible protective role of pre-treatment with Ginkgo biloba (EGb 761). [Sci Total Environ.](http://www.ncbi.nlm.nih.gov/pubmed/22014767) 410-411:59-64, 2011.

[Falcioni](https://pubmed.ncbi.nlm.nih.gov/?sort=date&term=Falcioni+L&cauthor_id=29530389), [L](https://pubmed.ncbi.nlm.nih.gov/29530389/#affiliation-1)., [Bua](https://pubmed.ncbi.nlm.nih.gov/?sort=date&term=Bua+L&cauthor_id=29530389),  L., [Tibaldi](https://pubmed.ncbi.nlm.nih.gov/?sort=date&term=Tibaldi+E&cauthor_id=29530389),  E.,  [Lauriola](https://pubmed.ncbi.nlm.nih.gov/?sort=date&term=Lauriola+M&cauthor_id=29530389),  [M](https://pubmed.ncbi.nlm.nih.gov/29530389/#affiliation-1).,  [De Angelis](https://pubmed.ncbi.nlm.nih.gov/?sort=date&term=De+Angelis+L&cauthor_id=29530389), [L](https://pubmed.ncbi.nlm.nih.gov/29530389/#affiliation-1).,  [Gnudi](https://pubmed.ncbi.nlm.nih.gov/?sort=date&term=Gnudi+F&cauthor_id=29530389), [F](https://pubmed.ncbi.nlm.nih.gov/29530389/#affiliation-1)., [Mandrioli](https://pubmed.ncbi.nlm.nih.gov/?sort=date&term=Mandrioli+D&cauthor_id=29530389), [D](https://pubmed.ncbi.nlm.nih.gov/29530389/#affiliation-1).,  [Manservigi](https://pubmed.ncbi.nlm.nih.gov/?sort=date&term=Manservigi+M&cauthor_id=29530389), [M](https://pubmed.ncbi.nlm.nih.gov/29530389/#affiliation-1)., [Manservisi](https://pubmed.ncbi.nlm.nih.gov/?sort=date&term=Manservisi+F&cauthor_id=29530389), [F](https://pubmed.ncbi.nlm.nih.gov/29530389/#affiliation-1)., [Manzoli](https://pubmed.ncbi.nlm.nih.gov/?sort=date&term=Manzoli+I&cauthor_id=29530389), [I](https://pubmed.ncbi.nlm.nih.gov/29530389/#affiliation-1)., [Menghetti](https://pubmed.ncbi.nlm.nih.gov/?sort=date&term=Menghetti+I&cauthor_id=29530389), [I](https://pubmed.ncbi.nlm.nih.gov/29530389/#affiliation-1)., [Montella](https://pubmed.ncbi.nlm.nih.gov/?sort=date&term=Montella+R&cauthor_id=29530389), [R](https://pubmed.ncbi.nlm.nih.gov/29530389/#affiliation-1)., [Panzacchi](https://pubmed.ncbi.nlm.nih.gov/?sort=date&term=Panzacchi+S&cauthor_id=29530389), [S](https://pubmed.ncbi.nlm.nih.gov/29530389/#affiliation-1)., [Sgargi](https://pubmed.ncbi.nlm.nih.gov/?sort=date&term=Sgargi+D&cauthor_id=29530389), [D](https://pubmed.ncbi.nlm.nih.gov/29530389/#affiliation-1)., [Strollo](https://pubmed.ncbi.nlm.nih.gov/?sort=date&term=Strollo+V&cauthor_id=29530389), [V](https://pubmed.ncbi.nlm.nih.gov/29530389/#affiliation-1)., [Vornoli](https://pubmed.ncbi.nlm.nih.gov/?sort=date&term=Vornoli+A&cauthor_id=29530389), [A](https://pubmed.ncbi.nlm.nih.gov/29530389/#affiliation-1)., [Belpoggi](https://pubmed.ncbi.nlm.nih.gov/?sort=date&term=Belpoggi+F&cauthor_id=29530389), [F.](https://pubmed.ncbi.nlm.nih.gov/29530389/#affiliation-2) Report of final results regarding brain and heart tumors in Sprague-Dawley rats exposed from prenatal life until natural death to mobile phone radiofrequency field representative of a 1.8 GHz GSM base station environmental emission. Environ Res. 165:496-503, 2018.

.

[Forgacs, Z](http://www.ncbi.nlm.nih.gov/entrez/query.fcgi?db=pubmed&cmd=Search&itool=pubmed_Abstract&term=%252522Forgacs+Z%252522%25255BAuthor%25255D)., [Somosy, Z](http://www.ncbi.nlm.nih.gov/entrez/query.fcgi?db=pubmed&cmd=Search&itool=pubmed_Abstract&term=%252522Somosy+Z%252522%25255BAuthor%25255D)., [Kubinyi, G](http://www.ncbi.nlm.nih.gov/entrez/query.fcgi?db=pubmed&cmd=Search&itool=pubmed_Abstract&term=%252522Kubinyi+G%252522%25255BAuthor%25255D)., [Bakos, J](http://www.ncbi.nlm.nih.gov/entrez/query.fcgi?db=pubmed&cmd=Search&itool=pubmed_Abstract&term=%252522Bakos+J%252522%25255BAuthor%25255D)., [Hudak, A](http://www.ncbi.nlm.nih.gov/entrez/query.fcgi?db=pubmed&cmd=Search&itool=pubmed_Abstract&term=%252522Hudak+A%252522%25255BAuthor%25255D)., [Surjan, A](http://www.ncbi.nlm.nih.gov/entrez/query.fcgi?db=pubmed&cmd=Search&itool=pubmed_Abstract&term=%252522Surjan+A%252522%25255BAuthor%25255D)., [Thuroczy, G](http://www.ncbi.nlm.nih.gov/entrez/query.fcgi?db=pubmed&cmd=Search&itool=pubmed_Abstract&term=%252522Thuroczy+G%252522%25255BAuthor%25255D). Effect of whole-body 1800 MHz GSM-like microwave exposure on testicular steroidogenesis and histology in mice. Reprod Toxicol. 22:111-117, 2006.

[Furtado-Filho, O.V](http://www.ncbi.nlm.nih.gov/pubmed?term=Furtado-Filho%20OV%5BAuthor%5D&cauthor=true&cauthor_uid=23789976)., [Borba, J.B](http://www.ncbi.nlm.nih.gov/pubmed?term=Borba%20JB%5BAuthor%5D&cauthor=true&cauthor_uid=23789976)., [Dallegrave, A](http://www.ncbi.nlm.nih.gov/pubmed?term=Dallegrave%20A%5BAuthor%5D&cauthor=true&cauthor_uid=23789976)., [Pizzolato, T.M](http://www.ncbi.nlm.nih.gov/pubmed?term=Pizzolato%20TM%5BAuthor%5D&cauthor=true&cauthor_uid=23789976)., [Henriques, J.A](http://www.ncbi.nlm.nih.gov/pubmed?term=Henriques%20JA%5BAuthor%5D&cauthor=true&cauthor_uid=23789976)., [Moreira, J.C](http://www.ncbi.nlm.nih.gov/pubmed?term=Moreira%20JC%5BAuthor%5D&cauthor=true&cauthor_uid=23789976)., [Saffi, J](http://www.ncbi.nlm.nih.gov/pubmed?term=Saffi%20J%5BAuthor%5D&cauthor=true&cauthor_uid=23789976). Effect of 950 MHz UHF electromagnetic radiation on biomarkers of oxidative damage, metabolism of UFA and antioxidants in the livers of young rats of different ages. [Int J Radiat Biol.](http://www.ncbi.nlm.nih.gov/pubmed/23789976) 90:159-168, 2014.

[Garaj-Vrhovac. V](http://www.ncbi.nlm.nih.gov/pubmed?term=Garaj-Vrhovac%20V%5BAuthor%5D&cauthor=true&cauthor_uid=20833106)., [Gajski, G](http://www.ncbi.nlm.nih.gov/pubmed?term=Gajski%20G%5BAuthor%5D&cauthor=true&cauthor_uid=20833106)., [Pažanin, S](http://www.ncbi.nlm.nih.gov/pubmed?term=Pa%C5%BEanin%20S%5BAuthor%5D&cauthor=true&cauthor_uid=20833106)., [Sarolić, A](http://www.ncbi.nlm.nih.gov/pubmed?term=Saroli%C4%87%20A%5BAuthor%5D&cauthor=true&cauthor_uid=20833106)., [Domijan, A.M](http://www.ncbi.nlm.nih.gov/pubmed?term=Domijan%20AM%5BAuthor%5D&cauthor=true&cauthor_uid=20833106)., [Flajs. D](http://www.ncbi.nlm.nih.gov/pubmed?term=Flajs%20D%5BAuthor%5D&cauthor=true&cauthor_uid=20833106)., [Peraica, M](http://www.ncbi.nlm.nih.gov/pubmed?term=Peraica%20M%5BAuthor%5D&cauthor=true&cauthor_uid=20833106). Assessment of cytogenetic damage and oxidative stress in personnel occupationally exposed to the pulsed microwave radiation of marine radar equipment. [Int J Hyg Environ Health.](http://www.ncbi.nlm.nih.gov/pubmed/20833106##) 4:59-65, 2011.

[Gautam](https://pubmed.ncbi.nlm.nih.gov/?sort=date&term=Gautam+R&cauthor_id=38102429) [R](https://pubmed.ncbi.nlm.nih.gov/38102429/#full-view-affiliation-1),  [Pardhiya](https://pubmed.ncbi.nlm.nih.gov/?sort=date&term=Pardhiya+S&cauthor_id=38102429) [S](https://pubmed.ncbi.nlm.nih.gov/38102429/#full-view-affiliation-1),  [Nirala](https://pubmed.ncbi.nlm.nih.gov/?sort=date&term=Nirala+JP&cauthor_id=38102429) [JP](https://pubmed.ncbi.nlm.nih.gov/38102429/#full-view-affiliation-1),  [Sarsaiya](https://pubmed.ncbi.nlm.nih.gov/?sort=date&term=Sarsaiya+P&cauthor_id=38102429) [P](https://pubmed.ncbi.nlm.nih.gov/38102429/#full-view-affiliation-1),  [Rajamani](https://pubmed.ncbi.nlm.nih.gov/?sort=date&term=Rajamani+P&cauthor_id=38102429) [P.](https://pubmed.ncbi.nlm.nih.gov/38102429/#full-view-affiliation-2) Effects of 4G mobile phone radiation exposure on reproductive, hepatic, renal, and hematological parameters of male Wistar rat. Environ Sci Pollut Res Int 31(3):4384-4399, 2024**.**

Gautam R, Jha N, Tomar AK, Nirala JP, Arora T, Rajamani P. Oxidative stress and testicular damage induced by chronic exposure to 35.5 GHz millimeter wave radiation in male Wistar rats. Andrology. 2025 Aug 12. doi: 10.1111/andr.70107.

[Ghazizadeh V](http://www.ncbi.nlm.nih.gov/pubmed?term=Ghazizadeh%20V%5BAuthor%5D&cauthor=true&cauthor_uid=24792079), [Nazıroğlu M](http://www.ncbi.nlm.nih.gov/pubmed?term=Naz%C4%B1ro%C4%9Flu%20M%5BAuthor%5D&cauthor=true&cauthor_uid=24792079). Electromagnetic radiation (Wi-Fi) and epilepsy induce calcium entry and apoptosis through activation of TRPV1 channel in hippocampus and dorsal root ganglion of rats. [Metab Brain Dis.](http://www.ncbi.nlm.nih.gov/pubmed/24792079) 29(3):787-799, 2014.

[Gökçek-Saraç, Ç](https://www.ncbi.nlm.nih.gov/pubmed/?term=G%C3%B6k%C3%A7ek-Sara%C3%A7%20%C3%87%5BAuthor%5D&cauthor=true&cauthor_uid=28565929), [Er, H](https://www.ncbi.nlm.nih.gov/pubmed/?term=Er%20H%5BAuthor%5D&cauthor=true&cauthor_uid=28565929)., [Kencebay Manas, C](https://www.ncbi.nlm.nih.gov/pubmed/?term=Kencebay%20Manas%20C%5BAuthor%5D&cauthor=true&cauthor_uid=28565929)., [Kantar Gok, D](https://www.ncbi.nlm.nih.gov/pubmed/?term=Kantar%20Gok%20D%5BAuthor%5D&cauthor=true&cauthor_uid=28565929)., [Özen, Ş](https://www.ncbi.nlm.nih.gov/pubmed/?term=%C3%96zen%20%C5%9E%5BAuthor%5D&cauthor=true&cauthor_uid=28565929)., [Derin, N](https://www.ncbi.nlm.nih.gov/pubmed/?term=Derin%20N%5BAuthor%5D&cauthor=true&cauthor_uid=28565929). Effects of acute and chronic exposure to both 900 MHz and 2100 MHz electromagnetic radiation on glutamate receptor signaling pathway. [Int J Radiat Biol.](https://www.ncbi.nlm.nih.gov/pubmed/28565929) 93:980-989, 2017.

Grémiaux, A., Girard, S., Guérin, V., Lothier, J., Baluška, F., Davies, E., Bonnet, P., Vian, A. Low-amplitude, high-frequency electromagnetic field exposure causes delayed and reduced growth in Rosa hybrida. J Plant Physiol. 190:44-53, 2016.

[Grigor'ev IuG](http://www.ncbi.nlm.nih.gov/pubmed?term=%22Grigor%27ev%20IuG%22%5BAuthor%5D), [Mikhaĭlov VF](http://www.ncbi.nlm.nih.gov/pubmed?term=%22Mikha%C4%ADlov%20VF%22%5BAuthor%5D), [Ivanov AA](http://www.ncbi.nlm.nih.gov/pubmed?term=%22Ivanov%20AA%22%5BAuthor%5D), [Mal'tsev VN](http://www.ncbi.nlm.nih.gov/pubmed?term=%22Mal%27tsev%20VN%22%5BAuthor%5D), [Ulanova AM](http://www.ncbi.nlm.nih.gov/pubmed?term=%22Ulanova%20AM%22%5BAuthor%5D), [Stavrakova NM](http://www.ncbi.nlm.nih.gov/pubmed?term=%22Stavrakova%20NM%22%5BAuthor%5D), [Nikolaeva IA](http://www.ncbi.nlm.nih.gov/pubmed?term=%22Nikolaeva%20IA%22%5BAuthor%5D), [Grigor'ev OA](http://www.ncbi.nlm.nih.gov/pubmed?term=%22Grigor%27ev%20OA%22%5BAuthor%5D). [Autoimmune processes after long-term low-level exposure to electromagnetic fields (the results of an experiment). Part 4. Manifestation of oxidative intracellular stress-reaction after long-term non-thermal EMF exposure of rats] [Radiats Biol Radioecol.](javascript:AL_get(this,%20'jour',%20'Radiats%20%0d%0aBiol%20Radioecol.');) 50(1):22-27, 2010. [Article in Russian]

Gulati, S., Kosik, P., Durdik, M., Skorvaga, M., Jakl, L., Markova, E., Belyaev, I. Effects of different mobile phone UMTS signals on DNA, apoptosis and oxidative stress in human lymphocytes.  Environ Pollut. 267:115632, 2020.

[Güler](https://pubmed.ncbi.nlm.nih.gov/?sort=date&term=G%C3%BCler+G&cauthor_id=26520616) [G](https://pubmed.ncbi.nlm.nih.gov/26520616/#full-view-affiliation-1),  [Ozgur](https://pubmed.ncbi.nlm.nih.gov/?sort=date&term=Ozgur+E&cauthor_id=26520616) [E](https://pubmed.ncbi.nlm.nih.gov/26520616/#full-view-affiliation-2),  [Keles](https://pubmed.ncbi.nlm.nih.gov/?sort=date&term=Keles+H&cauthor_id=26520616) [H](https://pubmed.ncbi.nlm.nih.gov/26520616/#full-view-affiliation-3), [Tomruk](https://pubmed.ncbi.nlm.nih.gov/?sort=date&term=Tomruk+A&cauthor_id=26520616) [A](https://pubmed.ncbi.nlm.nih.gov/26520616/#full-view-affiliation-1),  [Vural](https://pubmed.ncbi.nlm.nih.gov/?sort=date&term=Vural+SA&cauthor_id=26520616) [SA](https://pubmed.ncbi.nlm.nih.gov/26520616/#full-view-affiliation-4), [Seyhan](https://pubmed.ncbi.nlm.nih.gov/?sort=date&term=Seyhan+N&cauthor_id=26520616) [N.](https://pubmed.ncbi.nlm.nih.gov/26520616/#full-view-affiliation-1) Neurodegenerative changes and apoptosis induced by intrauterine and extrauterine exposure of radiofrequency radiation. J Chem Neuroanat 75(Pt B):128-133, 2016.

[Gupta, S.K](https://www.ncbi.nlm.nih.gov/pubmed/?term=Gupta%252520SK%25255BAuthor%25255D&cauthor=true&cauthor_uid=29872015)., [Mesharam, M.K](https://www.ncbi.nlm.nih.gov/pubmed/?term=Mesharam%252520MK%25255BAuthor%25255D&cauthor=true&cauthor_uid=29872015)., [Krishnamurthy, S](https://www.ncbi.nlm.nih.gov/pubmed/?term=Krishnamurthy%252520S%25255BAuthor%25255D&cauthor=true&cauthor_uid=29872015). Electromagnetic radiation 2450 MHz exposure causes cognition deficit with mitochondrial dysfunction and activation of intrinsic pathway of apoptosis in rats. [J Biosci.](https://www.ncbi.nlm.nih.gov/pubmed/?term=Gupta+SK+and+2450+MHz) 43:263-276, 2018.

[Gürler HS](http://www.ncbi.nlm.nih.gov/pubmed?term=G%C3%BCrler%20HS%5BAuthor%5D&cauthor=true&cauthor_uid=24844368), [Bilgici B](http://www.ncbi.nlm.nih.gov/pubmed?term=Bilgici%20B%5BAuthor%5D&cauthor=true&cauthor_uid=24844368), [Akar AK](http://www.ncbi.nlm.nih.gov/pubmed?term=Akar%20AK%5BAuthor%5D&cauthor=true&cauthor_uid=24844368), [Tomak L](http://www.ncbi.nlm.nih.gov/pubmed?term=Tomak%20L%5BAuthor%5D&cauthor=true&cauthor_uid=24844368), [Bedir A](http://www.ncbi.nlm.nih.gov/pubmed?term=Bedir%20A%5BAuthor%5D&cauthor=true&cauthor_uid=24844368). Increased DNA oxidation (8-OHdG) and protein oxidation (AOPP) by Low level electromagnetic field (2.45 GHz) in rat brain and protective effect of garlic. [Int J Radiat Biol.](http://www.ncbi.nlm.nih.gov/pubmed/24844368) 90:892-896, 2014.

[Halgamuge](https://pubmed.ncbi.nlm.nih.gov/?sort=pubdate&term=Halgamuge+MN&cauthor_id=25644316), [M.N](https://pubmed.ncbi.nlm.nih.gov/25644316/#affiliation-1).,  [Yak](https://pubmed.ncbi.nlm.nih.gov/?sort=pubdate&term=Yak+SK&cauthor_id=25644316), S.K.,  [Eberhardt](https://pubmed.ncbi.nlm.nih.gov/?sort=pubdate&term=Eberhardt+JL&cauthor_id=25644316), J.L. Reduced growth of soybean seedlings after exposure to weak microwave radiation from GSM 900 mobile phone and base station. Bioelectromagnetics. 36:87-95, 2015.

[Hancı H](http://www.ncbi.nlm.nih.gov/pubmed?term=Hanc%C4%B1%20H%5BAuthor%5D&cauthor=true&cauthor_uid=24095929), [Odacı E](http://www.ncbi.nlm.nih.gov/pubmed?term=Odac%C4%B1%20E%5BAuthor%5D&cauthor=true&cauthor_uid=24095929), [Kaya H](http://www.ncbi.nlm.nih.gov/pubmed?term=Kaya%20H%5BAuthor%5D&cauthor=true&cauthor_uid=24095929), [Aliyazıcıoğlu Y](http://www.ncbi.nlm.nih.gov/pubmed?term=Aliyaz%C4%B1c%C4%B1o%C4%9Flu%20Y%5BAuthor%5D&cauthor=true&cauthor_uid=24095929), [Turan I](http://www.ncbi.nlm.nih.gov/pubmed?term=Turan%20I%5BAuthor%5D&cauthor=true&cauthor_uid=24095929), [Demir S](http://www.ncbi.nlm.nih.gov/pubmed?term=Demir%20S%5BAuthor%5D&cauthor=true&cauthor_uid=24095929), [Colakoğlu S](http://www.ncbi.nlm.nih.gov/pubmed?term=Colako%C4%9Flu%20S%5BAuthor%5D&cauthor=true&cauthor_uid=24095929). The effect of prenatal exposure to 900-MHz electromagnetic field on the 21-old-day rat testicle. [Reprod Toxicol.](http://www.ncbi.nlm.nih.gov/pubmed/24095929) 42:203-209, 2013.

[Hancı H](http://www.ncbi.nlm.nih.gov/pubmed/?term=Hanc%C4%B1%20H%5BAuthor%5D&cauthor=true&cauthor_uid=25985826), [Türedi S](http://www.ncbi.nlm.nih.gov/pubmed/?term=T%C3%BCredi%20S%5BAuthor%5D&cauthor=true&cauthor_uid=25985826), [Topal Z](http://www.ncbi.nlm.nih.gov/pubmed/?term=Topal%20Z%5BAuthor%5D&cauthor=true&cauthor_uid=25985826), [Mercantepe T](http://www.ncbi.nlm.nih.gov/pubmed/?term=Mercantepe%20T%5BAuthor%5D&cauthor=true&cauthor_uid=25985826), [Bozkurt I](http://www.ncbi.nlm.nih.gov/pubmed/?term=Bozkurt%20I%5BAuthor%5D&cauthor=true&cauthor_uid=25985826), [Kaya H](http://www.ncbi.nlm.nih.gov/pubmed/?term=Kaya%20H%5BAuthor%5D&cauthor=true&cauthor_uid=25985826), [Ersöz Ş](http://www.ncbi.nlm.nih.gov/pubmed/?term=Ers%C3%B6z%20%C5%9E%5BAuthor%5D&cauthor=true&cauthor_uid=25985826), [Ünal B](http://www.ncbi.nlm.nih.gov/pubmed/?term=%C3%9Cnal%20B%5BAuthor%5D&cauthor=true&cauthor_uid=25985826), [Odacı E](http://www.ncbi.nlm.nih.gov/pubmed/?term=Odac%C4%B1%20E%5BAuthor%5D&cauthor=true&cauthor_uid=25985826). Can prenatal exposure to a 900 MHz electromagnetic field affect the morphology of the spleen and thymus, and alter biomarkers of oxidative damage in 21-day-old male rats? [Biotech Histochem.](http://www.ncbi.nlm.nih.gov/pubmed/25985826) 90(7):535-543, 2015.

[Hancı, H](https://www.ncbi.nlm.nih.gov/pubmed/?term=Hanc%2525C4%2525B1%252520H%25255BAuthor%25255D&cauthor=true&cauthor_uid=30009952)., [Kerimoğlu, G](https://www.ncbi.nlm.nih.gov/pubmed/?term=Kerimo%2525C4%25259Flu%252520G%25255BAuthor%25255D&cauthor=true&cauthor_uid=30009952)., [Mercantepe, T](https://www.ncbi.nlm.nih.gov/pubmed/?term=Mercantepe%252520T%25255BAuthor%25255D&cauthor=true&cauthor_uid=30009952)., [Odacı, E](https://www.ncbi.nlm.nih.gov/pubmed/?term=Odac%2525C4%2525B1%252520E%25255BAuthor%25255D&cauthor=true&cauthor_uid=30009952). Changes in testicular morphology and oxidative stress biomarkers in 60-day-old Sprague Dawley rats following exposure to continuous 900-MHz electromagnetic field for 1 h a day throughout adolescence. [Reprod Toxicol.](https://www.ncbi.nlm.nih.gov/pubmed/30009952) 81:71-78. 2018.

[Hancı](https://pubmed.ncbi.nlm.nih.gov/?sort=date&term=Hanc%C4%B1+H&cauthor_id=40099659) [H](https://pubmed.ncbi.nlm.nih.gov/40099659/#full-view-affiliation-1),  [Yenilmez](https://pubmed.ncbi.nlm.nih.gov/?sort=date&term=Yenilmez+E&cauthor_id=40099659) [E](https://pubmed.ncbi.nlm.nih.gov/40099659/#full-view-affiliation-2),  [Demir](https://pubmed.ncbi.nlm.nih.gov/?sort=date&term=Demir+S&cauthor_id=40099659) [S](https://pubmed.ncbi.nlm.nih.gov/40099659/#full-view-affiliation-3),  [Yıldırım](https://pubmed.ncbi.nlm.nih.gov/?sort=date&term=Y%C4%B1ld%C4%B1r%C4%B1m+M&cauthor_id=40099659) [M](https://pubmed.ncbi.nlm.nih.gov/40099659/#full-view-affiliation-4),  [Gedikli](https://pubmed.ncbi.nlm.nih.gov/?sort=date&term=Gedikli+%C3%96&cauthor_id=40099659) Ö,  [Kaya](https://pubmed.ncbi.nlm.nih.gov/?sort=date&term=Kaya+H&cauthor_id=40099659) [H.](https://pubmed.ncbi.nlm.nih.gov/40099659/#full-view-affiliation-6) The effect on rat peripheral nerve morphology and function of a 900-MHz electromagnetic field applied in the prenatal period. Electromagn Biol Med 2025 Mar 18:1-16. doi: 10.1080/15368378.2025.2479517. Online ahead of print.

Hatice Ş. Gürler, Birşen Bilgici, Ayşegül K. Akar, Leman Tomak & Abdülkerim Bedir. Increased DNA oxidation (8-OHdG) and protein oxidation (AOPP) by low level electromagnetic field (2.45 GHz) in rat brain and protective effect of garlic.  International Journal of Radiation Biology 90(10):892-896, 2014.

[He](https://pubmed.ncbi.nlm.nih.gov/?term=He+Q&cauthor_id=27190989), Q., [Sun](https://pubmed.ncbi.nlm.nih.gov/?term=Sun+Y&cauthor_id=27190989),Y.,  [Zong](https://pubmed.ncbi.nlm.nih.gov/?term=Zong+L&cauthor_id=27190989), L., [Tong](https://pubmed.ncbi.nlm.nih.gov/?term=Tong+J&cauthor_id=27190989),  J.,  [Cao](https://pubmed.ncbi.nlm.nih.gov/?term=Cao+Y&cauthor_id=27190989), Y. Induction of poly(ADP-ribose) polymerase in mouse bone marrow stromal cells exposed to 900 MHz radiofrequency fields: Preliminary observations. Biomed Res Int. 2016:4918691, 2016.

[He](https://pubmed.ncbi.nlm.nih.gov/?sort=date&term=He+Q&cauthor_id=28676262) [Q](https://pubmed.ncbi.nlm.nih.gov/28676262/#full-view-affiliation-1), [Zong](https://pubmed.ncbi.nlm.nih.gov/?sort=date&term=Zong+L&cauthor_id=28676262) [L](https://pubmed.ncbi.nlm.nih.gov/28676262/#full-view-affiliation-1),  [Sun](https://pubmed.ncbi.nlm.nih.gov/?sort=date&term=Sun+Y&cauthor_id=28676262) [Y](https://pubmed.ncbi.nlm.nih.gov/28676262/#full-view-affiliation-1), [Vijayalaxmi](https://pubmed.ncbi.nlm.nih.gov/?sort=date&term=Vijayalaxmi&cauthor_id=28676262) ,  [Prihoda](https://pubmed.ncbi.nlm.nih.gov/?sort=date&term=Prihoda+TJ&cauthor_id=28676262) [TJ](https://pubmed.ncbi.nlm.nih.gov/28676262/#full-view-affiliation-3), [Tong](https://pubmed.ncbi.nlm.nih.gov/?sort=date&term=Tong+J&cauthor_id=28676262) [J](https://pubmed.ncbi.nlm.nih.gov/28676262/#full-view-affiliation-1), [Cao](https://pubmed.ncbi.nlm.nih.gov/?sort=date&term=Cao+Y&cauthor_id=28676262) [Y.](https://pubmed.ncbi.nlm.nih.gov/28676262/#full-view-affiliation-4) Adaptive response in mouse bone marrow stromal cells exposed to 900 MHz radiofrequency fields: Impact of poly (ADP-ribose) polymerase (PARP). Mutat Res Genet Toxicol Environ Mutagen 820:19-25, 2017.

[Hekmat, A](http://www.ncbi.nlm.nih.gov/pubmed?term=Hekmat%252520A%25255BAuthor%25255D&cauthor=true&cauthor_uid=23164448)., [Saboury, A.A](http://www.ncbi.nlm.nih.gov/pubmed?term=Saboury%252520AA%25255BAuthor%25255D&cauthor=true&cauthor_uid=23164448)., [Moosavi-Movahedi, A.A](http://www.ncbi.nlm.nih.gov/pubmed?term=Moosavi-Movahedi%252520AA%25255BAuthor%25255D&cauthor=true&cauthor_uid=23164448). The toxic effects of mobile phone radiofrequency (940 MHz) on the structure of calf thymus DNA. [Ecotoxicol Environ Saf.](http://www.ncbi.nlm.nih.gov/pubmed/23164448) 88:35-41, 2013.

[Hidisoglu](https://pubmed.ncbi.nlm.nih.gov/?sort=date&term=Hidisoglu+E&cauthor_id=29939075) [E](https://pubmed.ncbi.nlm.nih.gov/29939075/#full-view-affiliation-1), [Kantar-Gok](https://pubmed.ncbi.nlm.nih.gov/?sort=date&term=Kantar-Gok+D&cauthor_id=29939075) [D](https://pubmed.ncbi.nlm.nih.gov/29939075/#full-view-affiliation-1),  [Ozen](https://pubmed.ncbi.nlm.nih.gov/?sort=date&term=Ozen+S&cauthor_id=29939075) [S](https://pubmed.ncbi.nlm.nih.gov/29939075/#full-view-affiliation-2),  [Yargicoglu](https://pubmed.ncbi.nlm.nih.gov/?sort=date&term=Yargicoglu+P&cauthor_id=29939075) [P.](https://pubmed.ncbi.nlm.nih.gov/29939075/#full-view-affiliation-1) Short-term 2.1 GHz radiofrequency radiation treatment induces significant changes on the auditory evoked potentials in adult rats. Int J Radiat Biol 94(9):858-871, 2018.

Ivaschuk, O.I., Jones, R.A., Ishida-Jones, T., Haggren, W., Adey, W.R., Phillips, J.L. Exposure of nerve growth factor-treated PC12 rat pheochromocytoma cells to a modulated radiofrequency field at 836.55 MHz: effects on c-jun and c-fos expression. Bioelectromagnetics. 18:223-229, 1997.

Jech, R., Sonka, K., Ruzicka, E., Nebuzelsky, A., Bohm, J., Juklickova, M., Nevsimalova, S. Electromagnetic field of mobile phones affects visual event related potential in patients with narcolepsy. Bioelectromagnetics. 22**:** 519-528, 2001.

Jha N, Sarsaiya P, Tomar AK, Pardhiya S, Nirala JP, Chaturvedi PK, Gupta S, Rajamani P. Effects of 700MHz radiofrequency radiation (5G lower band) on the reproductive parameters of female Wistar rats. Reprod Toxicol. 2025 Apr 10:108910.

[Jonwal](https://pubmed.ncbi.nlm.nih.gov/?sort=date&term=Jonwal+C&cauthor_id=30220680) [C](https://pubmed.ncbi.nlm.nih.gov/30220680/#affiliation-1),  [Sisodia](https://pubmed.ncbi.nlm.nih.gov/?sort=date&term=Sisodia+R&cauthor_id=30220680) R,  [K Saxena](https://pubmed.ncbi.nlm.nih.gov/?sort=date&term=Saxena+VK&cauthor_id=30220680) VK,  [Kesari](https://pubmed.ncbi.nlm.nih.gov/?sort=date&term=Kesari+KK&cauthor_id=30220680) KK. Effect of 2.45 GHz microwave radiation on the fertility pattern in male mice. Gen Physiol Biophys 37(4):453-460, 2018.

[Jooyan](https://pubmed.ncbi.nlm.nih.gov/?sort=date&term=Jooyan+N&cauthor_id=38092171) [N](https://pubmed.ncbi.nlm.nih.gov/38092171/#full-view-affiliation-1),  [Mortazavi](https://pubmed.ncbi.nlm.nih.gov/?sort=date&term=Mortazavi+SMJ&cauthor_id=38092171) [SMJ](https://pubmed.ncbi.nlm.nih.gov/38092171/#full-view-affiliation-2),  [Goliaei](https://pubmed.ncbi.nlm.nih.gov/?sort=date&term=Goliaei+B&cauthor_id=38092171) [B](https://pubmed.ncbi.nlm.nih.gov/38092171/#full-view-affiliation-3),  [Faraji-Dana](https://pubmed.ncbi.nlm.nih.gov/?sort=date&term=Faraji-Dana+R&cauthor_id=38092171) [R.](https://pubmed.ncbi.nlm.nih.gov/38092171/#full-view-affiliation-4) Indirect effects of interference of two emerging environmental contaminants on cell health: Radiofrequency radiation and gold nanoparticles. Chemosphere 11:140942, 2023.

[Karadayi](https://pubmed.ncbi.nlm.nih.gov/?sort=date&term=Karadayi+A&cauthor_id=38466013) [A](https://pubmed.ncbi.nlm.nih.gov/38466013/#full-view-affiliation-1), [Sarsmaz](https://pubmed.ncbi.nlm.nih.gov/?sort=date&term=Sarsmaz+H&cauthor_id=38466013) H,  [Çigel](https://pubmed.ncbi.nlm.nih.gov/?sort=date&term=%C3%87igel+A&cauthor_id=38466013) A,  [Engiz](https://pubmed.ncbi.nlm.nih.gov/?sort=date&term=Engiz+B&cauthor_id=38466013) B,  [Üna l](https://pubmed.ncbi.nlm.nih.gov/?sort=date&term=%C3%9Cnal+N&cauthor_id=38466013) N,  [Ürkmez](https://pubmed.ncbi.nlm.nih.gov/?sort=date&term=%C3%9Crkmez+S&cauthor_id=38466013) S,  [Gürgen](https://pubmed.ncbi.nlm.nih.gov/?sort=date&term=G%C3%BCrgen+S&cauthor_id=38466013) S.Does Microwave exposure at different doses in the pre/postnatal period affect growing rat bone development? Physiol Res 73(1):157-172, 2024.

[Karamazı](https://pubmed.ncbi.nlm.nih.gov/?sort=date&term=Karamaz%C4%B1+Y&cauthor_id=39692219) [Y](https://pubmed.ncbi.nlm.nih.gov/39692219/#full-view-affiliation-1),  [Emre](https://pubmed.ncbi.nlm.nih.gov/?sort=date&term=Emre+M&cauthor_id=39692219) [M](https://pubmed.ncbi.nlm.nih.gov/39692219/#full-view-affiliation-1),  [Uçar](https://pubmed.ncbi.nlm.nih.gov/?sort=date&term=U%C3%A7ar+S&cauthor_id=39692219) [S](https://pubmed.ncbi.nlm.nih.gov/39692219/#full-view-affiliation-2),  [Aksoy](https://pubmed.ncbi.nlm.nih.gov/?sort=date&term=Aksoy+G&cauthor_id=39692219) [G](https://pubmed.ncbi.nlm.nih.gov/39692219/#full-view-affiliation-3),  [Emre](https://pubmed.ncbi.nlm.nih.gov/?sort=date&term=Emre+T&cauthor_id=39692219) T,  [Tokuş](https://pubmed.ncbi.nlm.nih.gov/?sort=date&term=Toku%C5%9F+M&cauthor_id=39692219) [M.](https://pubmed.ncbi.nlm.nih.gov/39692219/#full-view-affiliation-1) Effect of 6 GHz radiofrequency electromagnetic field on the development of fetal bones. Electromagn Biol Med 44:17-25, 2025.

Katirci E, Kirimlioglu E, Oflamaz AO, Hidisoglu E, Cernomorcenco A, Yargıcoğlu P, Ozen S, Demir N. Expression levels of tam receptors and ligands in the testes of rats exposed to short and middle-term 2100 MHz radiofrequency radiation. Bioelectromagnetics. 45:235-248, 2024.

[Keleş, A.İ](https://www.ncbi.nlm.nih.gov/pubmed/?term=Kele%2525C5%25259F%252520A%2525C4%2525B0%25255BAuthor%25255D&cauthor=true&cauthor_uid=31465830)., [Nyengaard, J.R](https://www.ncbi.nlm.nih.gov/pubmed/?term=Nyengaard%252520JR%25255BAuthor%25255D&cauthor=true&cauthor_uid=31465830)., [Odacı, E](https://www.ncbi.nlm.nih.gov/pubmed/?term=Odac%2525C4%2525B1%252520E%25255BAuthor%25255D&cauthor=true&cauthor_uid=31465830). Changes in pyramidal and granular neuron numbers in the rat hippocampus 7 days after exposure to a continuous 900-MHz electromagnetic field during early and mid-adolescence. [J Chem Neuroanat.](https://www.ncbi.nlm.nih.gov/pubmed/?term=Nyengaard+and+900+MHz) 101:101681, 2019.

[Keleş](https://pubmed.ncbi.nlm.nih.gov/?sort=date&term=Kele%C5%9F+A%C4%B0&cauthor_id=39627484) [AI](https://pubmed.ncbi.nlm.nih.gov/39627484/#full-view-affiliation-1),  [Kaya](https://pubmed.ncbi.nlm.nih.gov/?sort=date&term=Kaya+H&cauthor_id=39627484) [H](https://pubmed.ncbi.nlm.nih.gov/39627484/#full-view-affiliation-2), [Keleş](https://pubmed.ncbi.nlm.nih.gov/?sort=date&term=Kele%C5%9F+G&cauthor_id=39627484) [G](https://pubmed.ncbi.nlm.nih.gov/39627484/#full-view-affiliation-3),  [Erol](https://pubmed.ncbi.nlm.nih.gov/?sort=date&term=Erol+HS&cauthor_id=39627484) [HS](https://pubmed.ncbi.nlm.nih.gov/39627484/#full-view-affiliation-4),  [Mercantepe](https://pubmed.ncbi.nlm.nih.gov/?sort=date&term=Mercantepe+T&cauthor_id=39627484) [T](https://pubmed.ncbi.nlm.nih.gov/39627484/#full-view-affiliation-5),  [Odaci](https://pubmed.ncbi.nlm.nih.gov/?sort=date&term=Odaci+E&cauthor_id=39627484) [E.](https://pubmed.ncbi.nlm.nih.gov/39627484/#full-view-affiliation-6) Exposure to a 0.9-GHz electromagnetic field on postnatal days 21-45 may trigger the renin-angiotensin system in male rat: a histological and biochemical study. J Mol Histol 56(1):22, 2024.

[Kerimoğlu G](https://www.ncbi.nlm.nih.gov/pubmed/?term=Kerimo%C4%9Flu%20G%5BAuthor%5D&cauthor=true&cauthor_uid=27650207), [Aslan A](https://www.ncbi.nlm.nih.gov/pubmed/?term=Aslan%20A%5BAuthor%5D&cauthor=true&cauthor_uid=27650207), [Baş O](https://www.ncbi.nlm.nih.gov/pubmed/?term=Ba%C5%9F%20O%5BAuthor%5D&cauthor=true&cauthor_uid=27650207), [Çolakoğlu S](https://www.ncbi.nlm.nih.gov/pubmed/?term=%C3%87olako%C4%9Flu%20S%5BAuthor%5D&cauthor=true&cauthor_uid=27650207), [Odacı E](https://www.ncbi.nlm.nih.gov/pubmed/?term=Odac%C4%B1%20E%5BAuthor%5D&cauthor=true&cauthor_uid=27650207). Adverse effects in lumbar spinal cord morphology and tissue biochemistry in Sprague Dawley male rats following exposure to a continuous 1-h a day 900-MHz electromagnetic field throughout adolescence. [J Chem Neuroanat.](https://www.ncbi.nlm.nih.gov/pubmed/27650207) 78:125-130, 2016a.

[Kerimoğlu G](https://www.ncbi.nlm.nih.gov/pubmed/?term=Kerimo%C4%9Flu%20G%5BAuthor%5D&cauthor=true&cauthor_uid=27430379), [Hancı H](https://www.ncbi.nlm.nih.gov/pubmed/?term=Hanc%C4%B1%20H%5BAuthor%5D&cauthor=true&cauthor_uid=27430379), [Baş O](https://www.ncbi.nlm.nih.gov/pubmed/?term=Ba%C5%9F%20O%5BAuthor%5D&cauthor=true&cauthor_uid=27430379), [Aslan A](https://www.ncbi.nlm.nih.gov/pubmed/?term=Aslan%20A%5BAuthor%5D&cauthor=true&cauthor_uid=27430379), [Erol HS](https://www.ncbi.nlm.nih.gov/pubmed/?term=Erol%20HS%5BAuthor%5D&cauthor=true&cauthor_uid=27430379), [Turgut A](https://www.ncbi.nlm.nih.gov/pubmed/?term=Turgut%20A%5BAuthor%5D&cauthor=true&cauthor_uid=27430379), [Kaya H](https://www.ncbi.nlm.nih.gov/pubmed/?term=Kaya%20H%5BAuthor%5D&cauthor=true&cauthor_uid=27430379), [Çankaya S](https://www.ncbi.nlm.nih.gov/pubmed/?term=%C3%87ankaya%20S%5BAuthor%5D&cauthor=true&cauthor_uid=27430379), [Sönmez OF](https://www.ncbi.nlm.nih.gov/pubmed/?term=S%C3%B6nmez%20OF%5BAuthor%5D&cauthor=true&cauthor_uid=27430379), [Odacı E](https://www.ncbi.nlm.nih.gov/pubmed/?term=Odac%C4%B1%20E%5BAuthor%5D&cauthor=true&cauthor_uid=27430379). Pernicious effects of long-term, continuous 900-MHz electromagnetic field throughout adolescence on hippocampus morphology, biochemistry and pyramidal neuron numbers in 60-day-old Sprague Dawley male rats. [J Chem Neuroanat.](https://www.ncbi.nlm.nih.gov/pubmed/27430379) 77:169-175, 2016b.

[Kerimoğlu G](https://www.ncbi.nlm.nih.gov/pubmed/?term=Kerimo%C4%9Flu%20G%5BAuthor%5D&cauthor=true&cauthor_uid=27715326), [Mercantepe T](https://www.ncbi.nlm.nih.gov/pubmed/?term=Mercantepe%20T%5BAuthor%5D&cauthor=true&cauthor_uid=27715326), [Erol HS](https://www.ncbi.nlm.nih.gov/pubmed/?term=Erol%20HS%5BAuthor%5D&cauthor=true&cauthor_uid=27715326), [Turgut A](https://www.ncbi.nlm.nih.gov/pubmed/?term=Turgut%20A%5BAuthor%5D&cauthor=true&cauthor_uid=27715326), [Kaya H](https://www.ncbi.nlm.nih.gov/pubmed/?term=Kaya%20H%5BAuthor%5D&cauthor=true&cauthor_uid=27715326), [Çolakoğlu S](https://www.ncbi.nlm.nih.gov/pubmed/?term=%C3%87olako%C4%9Flu%20S%5BAuthor%5D&cauthor=true&cauthor_uid=27715326), [Odacı E](https://www.ncbi.nlm.nih.gov/pubmed/?term=Odac%C4%B1%20E%5BAuthor%5D&cauthor=true&cauthor_uid=27715326). Effects of long-term exposure to 900 megahertz electromagnetic field on heart morphology and biochemistry of male adolescent rats. [Biotech Histochem.](https://www.ncbi.nlm.nih.gov/pubmed/27715326) 91(7):445-454, 2016c.

[Kerimoğlu G](https://www.ncbi.nlm.nih.gov/pubmed/?term=Kerimo%C4%9Flu%20G%5BAuthor%5D&cauthor=true&cauthor_uid=29331319), [Güney C](https://www.ncbi.nlm.nih.gov/pubmed/?term=G%C3%BCney%20C%5BAuthor%5D&cauthor=true&cauthor_uid=29331319), [Ersöz Ş](https://www.ncbi.nlm.nih.gov/pubmed/?term=Ers%C3%B6z%20%C5%9E%5BAuthor%5D&cauthor=true&cauthor_uid=29331319), [Odacı E](https://www.ncbi.nlm.nih.gov/pubmed/?term=Odac%C4%B1%20E%5BAuthor%5D&cauthor=true&cauthor_uid=29331319).A histopathological and biochemical evaluation of oxidative injury in the sciatic nerves of male rats exposed to a continuous 900-megahertz electromagnetic field throughout all periods of adolescence. [J Chem Neuroanat.](https://www.ncbi.nlm.nih.gov/pubmed/29331319) 91:1-7, 2018.

[Kesari, K.K](http://www.ncbi.nlm.nih.gov/sites/entrez?Db=pubmed&Cmd=Search&Term=%252522Kesari%252520KK%252522%25255BAuthor%25255D&itool=EntrezSystem2.PEntrez.Pubmed.Pubmed_ResultsPanel.Pubmed_DiscoveryPanel.Pubmed_RVAbstractPlus)., [Behari, J](http://www.ncbi.nlm.nih.gov/sites/entrez?Db=pubmed&Cmd=Search&Term=%252522Behari%252520J%252522%25255BAuthor%25255D&itool=EntrezSystem2.PEntrez.Pubmed.Pubmed_ResultsPanel.Pubmed_DiscoveryPanel.Pubmed_RVAbstractPlus). Fifty-gigahertz microwave exposure effect of radiations on rat brain. Appl Biochem Biotechnol. 158:126-139, 2009.

Kesari KK, Behari J. Microwave exposure affecting reproductive system in male rats. Appl Biochem Biotechnol 31:495-498, 2010a.

Kesari K.K., Behari J. Effects of microwave at 2.45 GHz radiations on reproductive system of male rats. Toxicology Environmental Chemistry 92(6):1135-1147, 2010b.

[Kesari, K.K](http://www.ncbi.nlm.nih.gov/pubmed?term=%252522Kesari%252520KK%252522%25255BAuthor%25255D)., [Behari, J](http://www.ncbi.nlm.nih.gov/pubmed?term=%252522Behari%252520J%252522%25255BAuthor%25255D)., [Kumar, S](http://www.ncbi.nlm.nih.gov/pubmed?term=%252522Kumar%252520S%252522%25255BAuthor%25255D). Mutagenic response of 2.45 GHz radiation exposure on rat brain. Int J Radiat Biol. 86:334-343, 2010.

[Kesari KK](http://www.ncbi.nlm.nih.gov/pubmed?term=Kesari%20KK%5BAuthor%5D&cauthor=true&cauthor_uid=23949848), [Meena R](http://www.ncbi.nlm.nih.gov/pubmed?term=Meena%20R%5BAuthor%5D&cauthor=true&cauthor_uid=23949848), [Nirala J](http://www.ncbi.nlm.nih.gov/pubmed?term=Nirala%20J%5BAuthor%5D&cauthor=true&cauthor_uid=23949848), [Kumar J](http://www.ncbi.nlm.nih.gov/pubmed?term=Kumar%20J%5BAuthor%5D&cauthor=true&cauthor_uid=23949848), [Verma HN](http://www.ncbi.nlm.nih.gov/pubmed?term=Verma%20HN%5BAuthor%5D&cauthor=true&cauthor_uid=23949848). Effect of 3G cell phone exposure with computer controlled 2-D stepper motor on non-thermal activation of the hsp27/p38MAPK stress pathway in rat brain. [Cell Biochem Biophys.](http://www.ncbi.nlm.nih.gov/pubmed/23949848) 68(2):347-358, 2014.

Kues HA, Monahan JC, D'Anna SA, McLeod DS, Lutty GA, Koslov S, Increased sensitivity of the non-human primate eye to microwave radiation following ophthalmic drug pretreatment. Bioelectromagnetics 13(5):379-393, 1992.

[Kulaber A](https://www.ncbi.nlm.nih.gov/pubmed/?term=Kulaber%20A%5BAuthor%5D&cauthor=true&cauthor_uid=28598680), [Kerimoğlu G](https://www.ncbi.nlm.nih.gov/pubmed/?term=Kerimo%C4%9Flu%20G%5BAuthor%5D&cauthor=true&cauthor_uid=28598680), [Ersöz Ş](https://www.ncbi.nlm.nih.gov/pubmed/?term=Ers%C3%B6z%20%C5%9E%5BAuthor%5D&cauthor=true&cauthor_uid=28598680), [Çolakoğlu S](https://www.ncbi.nlm.nih.gov/pubmed/?term=%C3%87olako%C4%9Flu%20S%5BAuthor%5D&cauthor=true&cauthor_uid=28598680), [Odacı E](https://www.ncbi.nlm.nih.gov/pubmed/?term=Odac%C4%B1%20E%5BAuthor%5D&cauthor=true&cauthor_uid=28598680). Alterations of thymic morphology and antioxidant biomarkers in 60-day-old male rats following exposure to a continuous 900 MHz electromagnetic field during adolescence. [Biotech Histochem.](https://www.ncbi.nlm.nih.gov/pubmed/28598680) 92(5):331-337, 2017

[Kumar, S](http://www.ncbi.nlm.nih.gov/pubmed?term=%252522Kumar%252520S%252522%25255BAuthor%25255D)., [Kesari, K.K](http://www.ncbi.nlm.nih.gov/pubmed?term=%252522Kesari%252520KK%252522%25255BAuthor%25255D)., [Behari, J](http://www.ncbi.nlm.nih.gov/pubmed?term=%252522Behari%252520J%252522%25255BAuthor%25255D). Influence of microwave exposure on fertility of male rats. Fertil Steril. 95:1500-1502, 2010a.

[Kumar, S](http://www.ncbi.nlm.nih.gov/pubmed?term=%252522Kumar%252520S%252522%25255BAuthor%25255D)., [Kesari, K.K](http://www.ncbi.nlm.nih.gov/pubmed?term=%252522Kesari%252520KK%252522%25255BAuthor%25255D)., [Behari, J](http://www.ncbi.nlm.nih.gov/pubmed?term=%252522Behari%252520J%252522%25255BAuthor%25255D).. Evaluation of genotoxic effects in male Wistar rats following microwave exposure. Indian J Exp Biol. 48:586-592, 2010b.

[Kumar S](http://www.ncbi.nlm.nih.gov/pubmed?term=Kumar%20S%5BAuthor%5D&cauthor=true&cauthor_uid=21876981), [Kesari KK](http://www.ncbi.nlm.nih.gov/pubmed?term=Kesari%20KK%5BAuthor%5D&cauthor=true&cauthor_uid=21876981), [Behari J](http://www.ncbi.nlm.nih.gov/pubmed?term=Behari%20J%5BAuthor%5D&cauthor=true&cauthor_uid=21876981). The therapeutic effect of a pulsed electromagnetic field on the reproductive patterns of male Wistar rats exposed to a 2.45-GHz microwave field. [Clinics (Sao Paulo).](http://www.ncbi.nlm.nih.gov/pubmed/21876981) 66(7):1237-1245, 2011a.

[Kumar S](http://www.ncbi.nlm.nih.gov/pubmed?term=%22Kumar%20S%22%5BAuthor%5D), [Kesari KK](http://www.ncbi.nlm.nih.gov/pubmed?term=%22Kesari%20KK%22%5BAuthor%5D), [Behari J](http://www.ncbi.nlm.nih.gov/pubmed?term=%22Behari%20J%22%5BAuthor%5D). Influence of microwave exposure on fertility of male rats. [Fertil Steril.](javascript:AL_get(this,%20'jour',%20'Fertil%20Steril.');) 95(4):1500-1502, 2011b.

[Kumar S](http://www.ncbi.nlm.nih.gov/pubmed?term=Kumar%20S%5BAuthor%5D&cauthor=true&cauthor_uid=22897403), [Behari J](http://www.ncbi.nlm.nih.gov/pubmed?term=Behari%20J%5BAuthor%5D&cauthor=true&cauthor_uid=22897403), [Sisodia R](http://www.ncbi.nlm.nih.gov/pubmed?term=Sisodia%20R%5BAuthor%5D&cauthor=true&cauthor_uid=22897403). Impact of microwave at X-band in the aetiology of male infertility. [Electromagn Biol Med.](http://www.ncbi.nlm.nih.gov/pubmed/22897403) 31(3):223-232, 2012.

Kumar, S., Behari, J., Sisodia, R. Influence of electromagnetic fields on reproductive system of male rats. Int J Radiat Biol. 89:147-154, 2013.

Kumar, A, Singh H.P., Batish, D.R., Kaur, S., Kohli, R.K. EMF radiations (1800 MHz)-inhibited early seedling growth of maize (Zea mays) involves alterations in starch and sucrose metabolism. Protoplasma. 253:1043-1049, 2016.

[Kumar, A](https://www.ncbi.nlm.nih.gov/pubmed/?term=Kumar%20A%5BAuthor%5D&cauthor=true&cauthor_uid=31698176)., [Kaur, S](https://www.ncbi.nlm.nih.gov/pubmed/?term=Kaur%20S%5BAuthor%5D&cauthor=true&cauthor_uid=31698176)., [Chandel, S](https://www.ncbi.nlm.nih.gov/pubmed/?term=Chandel%20S%5BAuthor%5D&cauthor=true&cauthor_uid=31698176)., [Singh, H.P](https://www.ncbi.nlm.nih.gov/pubmed/?term=Singh%20HP%5BAuthor%5D&cauthor=true&cauthor_uid=31698176)., [Batish, D.R](https://www.ncbi.nlm.nih.gov/pubmed/?term=Batish%20DR%5BAuthor%5D&cauthor=true&cauthor_uid=31698176)., [Kohli, R.K](https://www.ncbi.nlm.nih.gov/pubmed/?term=Kohli%20RK%5BAuthor%5D&cauthor=true&cauthor_uid=31698176). Comparative cyto- and genotoxicity of 900 MHz and 1800 MHz electromagnetic field radiations in root meristems of Allium cepa. [Ecotoxicol Environ Saf.](https://www.ncbi.nlm.nih.gov/pubmed/31698176) 188:109786, 2020.

[Kumar](https://pubmed.ncbi.nlm.nih.gov/?sort=pubdate&term=Kumar+R&cauthor_id=33035560), [R](https://pubmed.ncbi.nlm.nih.gov/33035560/#affiliation-1).,  [Deshmukh](https://pubmed.ncbi.nlm.nih.gov/?sort=pubdate&term=Deshmukh+PS&cauthor_id=33035560), [P](https://pubmed.ncbi.nlm.nih.gov/33035560/#affiliation-2).S., [Sharma](https://pubmed.ncbi.nlm.nih.gov/?sort=pubdate&term=Sharma+S&cauthor_id=33035560), [S](https://pubmed.ncbi.nlm.nih.gov/33035560/#affiliation-3).,  [Banerjee](https://pubmed.ncbi.nlm.nih.gov/?sort=pubdate&term=Banerjee+BD&cauthor_id=33035560), [B.D](https://pubmed.ncbi.nlm.nih.gov/33035560/#affiliation-4). Effect of mobile phone signal radiation on epigenetic modulation in the hippocampus of Wistar rat. Environ Res. 192:110297, 2021.

Kunjilwar KK, Behari J Effect of amplitude-modulated radio frequency radiation on cholinergic system of developing rats. Brain Res 601(1-2):321-324, 1993.

[Kuybulu AE](http://www.ncbi.nlm.nih.gov/pubmed/?term=Kuybulu%20AE%5BAuthor%5D&cauthor=true&cauthor_uid=26905323), [Öktem F](http://www.ncbi.nlm.nih.gov/pubmed/?term=%C3%96ktem%20F%5BAuthor%5D&cauthor=true&cauthor_uid=26905323), [Çiriş İM](http://www.ncbi.nlm.nih.gov/pubmed/?term=%C3%87iri%C5%9F%20%C4%B0M%5BAuthor%5D&cauthor=true&cauthor_uid=26905323), [Sutcu R](http://www.ncbi.nlm.nih.gov/pubmed/?term=Sutcu%20R%5BAuthor%5D&cauthor=true&cauthor_uid=26905323), [Örmeci AR](http://www.ncbi.nlm.nih.gov/pubmed/?term=%C3%96rmeci%20AR%5BAuthor%5D&cauthor=true&cauthor_uid=26905323), [Çömlekçi S](http://www.ncbi.nlm.nih.gov/pubmed/?term=%C3%87%C3%B6mlek%C3%A7i%20S%5BAuthor%5D&cauthor=true&cauthor_uid=26905323), [Uz E](http://www.ncbi.nlm.nih.gov/pubmed/?term=Uz%20E%5BAuthor%5D&cauthor=true&cauthor_uid=26905323). Effects of long-term pre- and post-natal exposure to 2.45 GHz wireless devices on developing male rat kidney. [Ren Fail.](http://www.ncbi.nlm.nih.gov/pubmed/26905323?dopt=Abstract) 38(4):571-580, 2016.

[Kuzay D](https://www.ncbi.nlm.nih.gov/pubmed/?term=Kuzay%20D%5BAuthor%5D&cauthor=true&cauthor_uid=28516790), [Ozer C](https://www.ncbi.nlm.nih.gov/pubmed/?term=Ozer%20C%5BAuthor%5D&cauthor=true&cauthor_uid=28516790), [Sirav B](https://www.ncbi.nlm.nih.gov/pubmed/?term=Sirav%20B%5BAuthor%5D&cauthor=true&cauthor_uid=28516790), [Canseven AG](https://www.ncbi.nlm.nih.gov/pubmed/?term=Canseven%20AG%5BAuthor%5D&cauthor=true&cauthor_uid=28516790), [Seyhan N](https://www.ncbi.nlm.nih.gov/pubmed/?term=Seyhan%20N%5BAuthor%5D&cauthor=true&cauthor_uid=28516790). Oxidative effects of extremely low frequency magnetic field and radio frequency radiation on testes tissues of diabetic and healthy rats. [Bratisl Lek Listy.](https://www.ncbi.nlm.nih.gov/pubmed/28516790) 118(5):278-282, 2017.

Kwee, S., Raskmark, P., Velizarov, P. Changes in cellular proteins due to environmental non-ionizing radiation. i. heat-shock proteins. Electro- and Magnetobiol. 20:141-152, 2001.

Lameth J, Royer J, Martin A, Marie C, Arnaud-Cormos D, Lévêque P, Poirier R, Edeline JM, Mallat M. Repeated Head Exposures to a 5G-3.5 GHz Signal Do Not Alter Behavior but Modify Intracortical Gene Expression in Adult Male Mice. Int J Mol Sci. 26(6):2459,2025.

Lerchl, A., Krüger, H., Niehaus, M., Streckert, J.R., Bitz, A.K., Hansen, V. Effects of mobile phone electromagnetic fields at nonthermal SAR values on melatonin and body weight of Djungarian hamsters (Phodopus sungorus). J Pineal Res. 44:267-272, 2008.

[Lian, H.Y](https://www.ncbi.nlm.nih.gov/pubmed/?term=Lian%20HY%5BAuthor%5D&cauthor=true&cauthor_uid=29214607)., [Lin, K.W](https://www.ncbi.nlm.nih.gov/pubmed/?term=Lin%20KW%5BAuthor%5D&cauthor=true&cauthor_uid=29214607)., [Yang, C](https://www.ncbi.nlm.nih.gov/pubmed/?term=Yang%20C%5BAuthor%5D&cauthor=true&cauthor_uid=29214607)., [Cai, P](https://www.ncbi.nlm.nih.gov/pubmed/?term=Cai%20P%5BAuthor%5D&cauthor=true&cauthor_uid=29214607). Generation and propagation of yeast prion [URE3] are elevated under electromagnetic field. [Cell Stress Chaperones.](https://www.ncbi.nlm.nih.gov/pubmed/29214607) 23:581-594, 2018.

[Lin KW](https://www.ncbi.nlm.nih.gov/pubmed/?term=Lin%20KW%5BAuthor%5D&cauthor=true&cauthor_uid=27630630), [Yang CJ](https://www.ncbi.nlm.nih.gov/pubmed/?term=Yang%20CJ%5BAuthor%5D&cauthor=true&cauthor_uid=27630630), [Lian HY](https://www.ncbi.nlm.nih.gov/pubmed/?term=Lian%20HY%5BAuthor%5D&cauthor=true&cauthor_uid=27630630), [Cai P](https://www.ncbi.nlm.nih.gov/pubmed/?term=Cai%20P%5BAuthor%5D&cauthor=true&cauthor_uid=27630630). Exposure of ELF-EMF and RF-EMF increase the rate of glucose transport and TCA cycle in budding yeast. [Front Microbiol.](https://www.ncbi.nlm.nih.gov/pubmed/?term=Lin+K-W+and+Yang+C-J+and+yeast) 7:1378, 2016.

[López-Furelos](https://pubmed.ncbi.nlm.nih.gov/?sort=date&term=L%C3%B3pez-Furelos+A&cauthor_id=27589837), [A](https://pubmed.ncbi.nlm.nih.gov/27589837/#affiliation-1).,  [Leiro-Vidal](https://pubmed.ncbi.nlm.nih.gov/?sort=date&term=Leiro-Vidal+JM&cauthor_id=27589837), [J.M](https://pubmed.ncbi.nlm.nih.gov/27589837/#affiliation-2).,  [Salas-Sánchez](https://pubmed.ncbi.nlm.nih.gov/?sort=date&term=Salas-S%C3%A1nchez+A%C3%81&cauthor_id=27589837), [A.Á](https://pubmed.ncbi.nlm.nih.gov/27589837/#affiliation-3).,  [Ares-Pena](https://pubmed.ncbi.nlm.nih.gov/?sort=date&term=Ares-Pena+FJ&cauthor_id=27589837), [F.J](https://pubmed.ncbi.nlm.nih.gov/27589837/#affiliation-3).,  [López-Martín](https://pubmed.ncbi.nlm.nih.gov/?sort=date&term=L%C3%B3pez-Mart%C3%ADn+ME&cauthor_id=27589837), [M.E.](https://pubmed.ncbi.nlm.nih.gov/27589837/#affiliation-1) Evidence of cellular stress and caspase-3 resulting from a combined two-frequency signal in the cerebrum and cerebellum of sprague-dawley rats. Oncotarget. 7:64674-64689, 2016.

[López-Furelos A](https://www.ncbi.nlm.nih.gov/pubmed/?term=L%C3%B3pez-Furelos%20A%5BAuthor%5D&cauthor=true&cauthor_uid=29659305), [Salas-Sánchez AA](https://www.ncbi.nlm.nih.gov/pubmed/?term=Salas-S%C3%A1nchez%20AA%5BAuthor%5D&cauthor=true&cauthor_uid=29659305), [Ares-Pena FJ](https://www.ncbi.nlm.nih.gov/pubmed/?term=Ares-Pena%20FJ%5BAuthor%5D&cauthor=true&cauthor_uid=29659305), [Leiro-Vidal JM](https://www.ncbi.nlm.nih.gov/pubmed/?term=Leiro-Vidal%20JM%5BAuthor%5D&cauthor=true&cauthor_uid=29659305), [López-Martín E](https://www.ncbi.nlm.nih.gov/pubmed/?term=L%C3%B3pez-Mart%C3%ADn%20E%5BAuthor%5D&cauthor=true&cauthor_uid=29659305). Exposure to radiation from single or combined radio frequencies provokes macrophage dysfunction in the RAW 264.7 cell line. I[nt J Radiat Biol.](https://www.ncbi.nlm.nih.gov/pubmed/29659305) (6):607-618, 2018.

[López-Martín, E](http://www.ncbi.nlm.nih.gov/pubmed?term=L%C3%B3pez-Mart%C3%ADn%20E%5BAuthor%5D&cauthor=true&cauthor_uid=19115403)., [Bregains, J](http://www.ncbi.nlm.nih.gov/pubmed?term=Bregains%20J%5BAuthor%5D&cauthor=true&cauthor_uid=19115403)., [Relova-Quinteiro, J.L](http://www.ncbi.nlm.nih.gov/pubmed?term=Relova-Quinteiro%20JL%5BAuthor%5D&cauthor=true&cauthor_uid=19115403)., [Cadarso-Suárez, C](http://www.ncbi.nlm.nih.gov/pubmed?term=Cadarso-Su%C3%A1rez%20C%5BAuthor%5D&cauthor=true&cauthor_uid=19115403)., [Jorge-Barreiro, F.J](http://www.ncbi.nlm.nih.gov/pubmed?term=Jorge-Barreiro%20FJ%5BAuthor%5D&cauthor=true&cauthor_uid=19115403)., [Ares-Pena, F.J](http://www.ncbi.nlm.nih.gov/pubmed?term=Ares-Pena%20FJ%5BAuthor%5D&cauthor=true&cauthor_uid=19115403). The action of pulse-modulated GSM radiation increases regional changes in brain activity and c-Fos expression in cortical and subcortical areas in a rat model of picrotoxin-induced seizure proneness. [J Neurosci Res.](http://www.ncbi.nlm.nih.gov/pubmed/19115403) 87:1484-1499, 2009.

[Manta AK](http://www.ncbi.nlm.nih.gov/pubmed?term=Manta%20AK%5BAuthor%5D&cauthor=true&cauthor_uid=23781995), [Stravopodis DJ](http://www.ncbi.nlm.nih.gov/pubmed?term=Stravopodis%20DJ%5BAuthor%5D&cauthor=true&cauthor_uid=23781995), [Papassideri IS](http://www.ncbi.nlm.nih.gov/pubmed?term=Papassideri%20IS%5BAuthor%5D&cauthor=true&cauthor_uid=23781995), [Margaritis LH](http://www.ncbi.nlm.nih.gov/pubmed?term=Margaritis%20LH%5BAuthor%5D&cauthor=true&cauthor_uid=23781995). Reactive oxygen species elevation and recovery in Drosophila bodies and ovaries following short-term and long-term exposure to DECT base EMF. [Electromagn Biol Med.](http://www.ncbi.nlm.nih.gov/pubmed/23781995) 33(2):118-131, 2014.

[Manta AK](https://www.ncbi.nlm.nih.gov/pubmed/?term=Manta%20AK%5BAuthor%5D&cauthor=true&cauthor_uid=27960592), [Papadopoulou D](https://www.ncbi.nlm.nih.gov/pubmed/?term=Papadopoulou%20D%5BAuthor%5D&cauthor=true&cauthor_uid=27960592), [Polyzos AP](https://www.ncbi.nlm.nih.gov/pubmed/?term=Polyzos%20AP%5BAuthor%5D&cauthor=true&cauthor_uid=27960592), [Fragopoulou AF](https://www.ncbi.nlm.nih.gov/pubmed/?term=Fragopoulou%20AF%5BAuthor%5D&cauthor=true&cauthor_uid=27960592), [Skouroliakou AS](https://www.ncbi.nlm.nih.gov/pubmed/?term=Skouroliakou%20AS%5BAuthor%5D&cauthor=true&cauthor_uid=27960592), [Thanos D](https://www.ncbi.nlm.nih.gov/pubmed/?term=Thanos%20D%5BAuthor%5D&cauthor=true&cauthor_uid=27960592), [Stravopodis DJ](https://www.ncbi.nlm.nih.gov/pubmed/?term=Stravopodis%20DJ%5BAuthor%5D&cauthor=true&cauthor_uid=27960592), [Margaritis LH](https://www.ncbi.nlm.nih.gov/pubmed/?term=Margaritis%20LH%5BAuthor%5D&cauthor=true&cauthor_uid=27960592). Mobile-phone radiation-induced perturbation of gene-expression profiling, redox equilibrium and sporadic-apoptosis control in the ovary of Drosophila melanogaster. [Fly (Austin).](https://www.ncbi.nlm.nih.gov/pubmed/27960592) 11(2):75-95, 2017.

Marinelli, F., La Sala, D., Cicciotti, G., Cattini, L., Trimarchi, C., Putti, S., Zamparelli, A., Giuliani, L., Tomassetti, G., Cinti ,C. Exposure to 900 MHz electromagnetic field induces an unbalance between pro-apoptotic and pro-survival signals in T-lymphoblastoid leukemia CCRF-CEM cells. J Cell Physiol. 198:324-332, 2004.

[Markovà, E](http://www.ncbi.nlm.nih.gov/pubmed?term=%252522Markov%2525C3%2525A0%252520E%252522%25255BAuthor%25255D&itool=EntrezSystem2.PEntrez.Pubmed.Pubmed_ResultsPanel.Pubmed_RVAbstract)., [Hillert, L](http://www.ncbi.nlm.nih.gov/pubmed?term=%252522Hillert%252520L%252522%25255BAuthor%25255D&itool=EntrezSystem2.PEntrez.Pubmed.Pubmed_ResultsPanel.Pubmed_RVAbstract)., [Malmgren, L](http://www.ncbi.nlm.nih.gov/pubmed?term=%252522Malmgren%252520L%252522%25255BAuthor%25255D&itool=EntrezSystem2.PEntrez.Pubmed.Pubmed_ResultsPanel.Pubmed_RVAbstract)., [Persson, B.R](http://www.ncbi.nlm.nih.gov/pubmed?term=%252522Persson%252520BR%252522%25255BAuthor%25255D&itool=EntrezSystem2.PEntrez.Pubmed.Pubmed_ResultsPanel.Pubmed_RVAbstract)., [Belyaev, I.Y](http://www.ncbi.nlm.nih.gov/pubmed?term=%252522Belyaev%252520IY%252522%25255BAuthor%25255D&itool=EntrezSystem2.PEntrez.Pubmed.Pubmed_ResultsPanel.Pubmed_RVAbstract). Microwaves from GSM mobile telephones affect 53BP1 and gamma-H2AX foci in human lymphocytes from hypersensitive and healthy persons. Environ Health Perspect. 113:1172-1177, 2005.

Markovà, E., Malmgren, L.O., Belyaev, I.Y. Microwaves from mobile phones inhibit 53 BP1

focus formation in human stem cells more strongly than in differentiated cells: possible

mechanistic link to cancer risk. Environ Health Perspect. 118:394-399, 2010.

Meena R, Kumari K, Kumar J, Rajamani P, Verma HN, Kesari KK. Therapeutic approaches of melatonin in microwave radiations-induced oxidative stress-mediated toxicity on male fertility pattern of Wistar rats. Electromagn Biol Med. 33(2):81-91, 2014.

[Megha, K](http://www.ncbi.nlm.nih.gov/pubmed?term=Megha%20K%5BAuthor%5D&cauthor=true&cauthor_uid=23986973)., [Deshmukh, P.S](http://www.ncbi.nlm.nih.gov/pubmed?term=Deshmukh%20PS%5BAuthor%5D&cauthor=true&cauthor_uid=23986973)., [Banerjee, B.D](http://www.ncbi.nlm.nih.gov/pubmed?term=Banerjee%20BD%5BAuthor%5D&cauthor=true&cauthor_uid=23986973)., [Tripathi, A.K](http://www.ncbi.nlm.nih.gov/pubmed?term=Tripathi%20AK%5BAuthor%5D&cauthor=true&cauthor_uid=23986973)., [Abegaonkar, M.P](http://www.ncbi.nlm.nih.gov/pubmed?term=Abegaonkar%20MP%5BAuthor%5D&cauthor=true&cauthor_uid=23986973). Microwave radiation induced oxidative stress, cognitive impairment and inflammation in brain of Fischer rats. [Indian J Exp Biol.](http://www.ncbi.nlm.nih.gov/pubmed/23986973) 50:889-896, 2012.

Megha, K., Deshmukh,P.S., Ravi, A.K., Tripathi, A.K., Abegaonkar, M.P., Banerjee, B.D. [Effect of low-intensity microwave radiation on monoamine neurotransmitters and their key regulating enzymes in rat brain.](https://pubmed.ncbi.nlm.nih.gov/25672490/) Cell Biochem Biophys. 73:93-100, 2015a.

Megha, K., Deshmukh, P.S., Banerjee, B.D., Tripathi, A.K., Ahmed, R., Abegaonkar, M.P. Low intensity microwave radiation induced oxidative stress, inflammatory response and DNA damage in rat brain. NeuroToxicol. 51:158-165, 2015b.

[Migdal](https://pubmed.ncbi.nlm.nih.gov/?sort=date&term=Migdal+P&cauthor_id=37172069) [P](https://pubmed.ncbi.nlm.nih.gov/37172069/#full-view-affiliation-1), [Bieńkowski](https://pubmed.ncbi.nlm.nih.gov/?sort=date&term=Bie%C5%84kowski+P&cauthor_id=37172069) [P](https://pubmed.ncbi.nlm.nih.gov/37172069/#full-view-affiliation-2),  [Cebrat](https://pubmed.ncbi.nlm.nih.gov/?sort=date&term=Cebrat+M&cauthor_id=37172069) [M](https://pubmed.ncbi.nlm.nih.gov/37172069/#full-view-affiliation-3),  [Berbeć](https://pubmed.ncbi.nlm.nih.gov/?sort=date&term=Berbe%C4%87+E&cauthor_id=37172069) [E](https://pubmed.ncbi.nlm.nih.gov/37172069/#full-view-affiliation-1),  [Plotnik](https://pubmed.ncbi.nlm.nih.gov/?sort=date&term=Plotnik+M&cauthor_id=37172069) [M](https://pubmed.ncbi.nlm.nih.gov/37172069/#full-view-affiliation-1), [Murawska](https://pubmed.ncbi.nlm.nih.gov/?sort=date&term=Murawska+A&cauthor_id=37172069) [A](https://pubmed.ncbi.nlm.nih.gov/37172069/#full-view-affiliation-1), [Sobkiewicz](https://pubmed.ncbi.nlm.nih.gov/?sort=date&term=Sobkiewicz+P&cauthor_id=37172069) [P](https://pubmed.ncbi.nlm.nih.gov/37172069/#full-view-affiliation-2), [Łaszkiewicz](https://pubmed.ncbi.nlm.nih.gov/?sort=date&term=%C5%81aszkiewicz+A&cauthor_id=37172069) [A](https://pubmed.ncbi.nlm.nih.gov/37172069/#full-view-affiliation-3),  [Latarowski](https://pubmed.ncbi.nlm.nih.gov/?sort=date&term=Latarowski+K&cauthor_id=37172069) [K.](https://pubmed.ncbi.nlm.nih.gov/37172069/#full-view-affiliation-4) Exposure to a 900 MHz electromagnetic field induces a response of the honey bee organism on the level of enzyme activity and the expression of stress-related genes. PLoS One 18(5):e0285522, 2023

[Misa Agustiño](https://pubmed.ncbi.nlm.nih.gov/?sort=date&term=Misa+Agusti%C3%B1o+MJ&cauthor_id=23213477), [M.J](https://pubmed.ncbi.nlm.nih.gov/23213477/#affiliation-1).,  [Leiro](https://pubmed.ncbi.nlm.nih.gov/?sort=date&term=Leiro+JM&cauthor_id=23213477), J.M.,  [Mora](https://pubmed.ncbi.nlm.nih.gov/?sort=date&term=Jorge+Mora+MT&cauthor_id=23213477), M.T.J.,  [Rodríguez-González](https://pubmed.ncbi.nlm.nih.gov/?sort=date&term=Rodr%C3%ADguez-Gonz%C3%A1lez+JA&cauthor_id=23213477), J.A.,  [Barreiro](https://pubmed.ncbi.nlm.nih.gov/?sort=date&term=Jorge+Barreiro+FJ&cauthor_id=23213477), F.J.J.,  [Ares-Pena](https://pubmed.ncbi.nlm.nih.gov/?sort=date&term=Ares-Pena+FJ&cauthor_id=23213477), F.J., [López-Martín](https://pubmed.ncbi.nlm.nih.gov/?sort=date&term=L%C3%B3pez-Mart%C3%ADn+E&cauthor_id=23213477), E. Electromagnetic fields at 2.45 GHz trigger changes in heat shock proteins 90 and 70 without altering apoptotic activity in rat thyroid gland. Biol Open. 1:831-838, 2012.

Mohammed, H.S., Fahmy, H.M., Radwah, N.M., Elsayed, A.A. Non-thermal continuous and modulated electromagnetic radiation fields effects on sleep EEG of rats. J Adv Res. 4:81-187, 2013.

Nakamura, H, Seto, T, Hatta, K, Matsuzaki, I, Nagase, H, Yoshida, M, Ogino, K, Natural killer cell activity reduced by microwave exposure during pregnancy is mediated by opioid systems. Environ Res 79(2):106-113, 1998.

Navakatikian, M.A., Tomashevskaya, L.A. Phasic behavioral and endocrine effects of microwaves of nonthermal intensity. In “Biological Effects of Electric and Magnetic Fields, Volume 1," D.O. Carpenter (ed) Academic Press, San Diego, CA, 1994, pp.333-342.

[Naziroğlu M](http://www.ncbi.nlm.nih.gov/pubmed?term=Naziro%C4%9Flu%20M%5BAuthor%5D&cauthor=true&cauthor_uid=19637079), [Gümral N](http://www.ncbi.nlm.nih.gov/pubmed?term=G%C3%BCmral%20N%5BAuthor%5D&cauthor=true&cauthor_uid=19637079). Modulator effects of L-carnitine and selenium on wireless devices (2.45 GHz)-induced oxidative stress and electroencephalography records in brain of rat. [Int J Radiat Biol.](http://www.ncbi.nlm.nih.gov/pubmed/19637079) 85(8):680-689, 2009.

[Nazıroğlu, M](http://www.ncbi.nlm.nih.gov/pubmed?term=Naz%C4%B1ro%C4%9Flu%20M%5BAuthor%5D&cauthor=true&cauthor_uid=22489926)., [Ciğ, B](http://www.ncbi.nlm.nih.gov/pubmed?term=Ci%C4%9F%20B%5BAuthor%5D&cauthor=true&cauthor_uid=22489926)., [Doğan, S](http://www.ncbi.nlm.nih.gov/pubmed?term=Do%C4%9Fan%20S%5BAuthor%5D&cauthor=true&cauthor_uid=22489926)., [Uğuz, A.C](http://www.ncbi.nlm.nih.gov/pubmed?term=U%C4%9Fuz%20AC%5BAuthor%5D&cauthor=true&cauthor_uid=22489926)., [Dilek, S](http://www.ncbi.nlm.nih.gov/pubmed?term=Dilek%20S%5BAuthor%5D&cauthor=true&cauthor_uid=22489926)., [Faouzi, D](http://www.ncbi.nlm.nih.gov/pubmed?term=Faouzi%20D%5BAuthor%5D&cauthor=true&cauthor_uid=22489926). 2.45-Gz wireless devices induce oxidative stress and proliferation through cytosolic Ca²⁺ influx in human leukemia cancer cells. [Int J Radiat Biol.](http://www.ncbi.nlm.nih.gov/pubmed/22489926) 88:449-456, 2012a.

[Nazıroğlu M](http://www.ncbi.nlm.nih.gov/pubmed?term=Naz%C4%B1ro%C4%9Flu%20M%5BAuthor%5D&cauthor=true&cauthor_uid=22019785), [Çelik Ö](http://www.ncbi.nlm.nih.gov/pubmed?term=%C3%87elik%20%C3%96%5BAuthor%5D&cauthor=true&cauthor_uid=22019785), [Özgül C](http://www.ncbi.nlm.nih.gov/pubmed?term=%C3%96zg%C3%BCl%20C%5BAuthor%5D&cauthor=true&cauthor_uid=22019785), [Çiğ B](http://www.ncbi.nlm.nih.gov/pubmed?term=%C3%87i%C4%9F%20B%5BAuthor%5D&cauthor=true&cauthor_uid=22019785), [Doğan S](http://www.ncbi.nlm.nih.gov/pubmed?term=Do%C4%9Fan%20S%5BAuthor%5D&cauthor=true&cauthor_uid=22019785), [Bal R](http://www.ncbi.nlm.nih.gov/pubmed?term=Bal%20R%5BAuthor%5D&cauthor=true&cauthor_uid=22019785), [Gümral N](http://www.ncbi.nlm.nih.gov/pubmed?term=G%C3%BCmral%20N%5BAuthor%5D&cauthor=true&cauthor_uid=22019785), [Rodríguez AB](http://www.ncbi.nlm.nih.gov/pubmed?term=Rodr%C3%ADguez%20AB%5BAuthor%5D&cauthor=true&cauthor_uid=22019785), [Pariente JA](http://www.ncbi.nlm.nih.gov/pubmed?term=Pariente%20JA%5BAuthor%5D&cauthor=true&cauthor_uid=22019785). Melatonin modulates wireless (2.45 GHz)-induced oxidative injury through TRPM2 and voltage gated Ca(2+) channels in brain and dorsal root ganglion in rat. [Physiol Behav.](http://www.ncbi.nlm.nih.gov/pubmed/22019785) 105(3):683-692, 2012b.

[Nittby](https://springerlink3.metapress.com/content/?Author=Henrietta+Nittby), H., [Widegren](https://springerlink3.metapress.com/content/?Author=Bengt+Widegren), B.,  [Krogh](https://springerlink3.metapress.com/content/?Author=Morten+Krogh), M., [Grafström](https://springerlink3.metapress.com/content/?Author=Gustav+Grafstr%2525c3%2525b6m), G., [Berlin](https://springerlink3.metapress.com/content/?Author=Henrik+Berlin), H.,  [Rehn](https://springerlink3.metapress.com/content/?Author=Gustav+Rehn), G.,  [Eberhardt](https://springerlink3.metapress.com/content/?Author=Jacob+L.+Eberhardt), J.L.,  [Malmgren](https://springerlink3.metapress.com/content/?Author=Lars+Malmgren), L.,  [Persson](https://springerlink3.metapress.com/content/?Author=Bertil+R.+R.+Persson), B.R.R., Salford, L. [Exposure to radiation from global system for mobile communications at 1,800 MHz significantly changes gene expression in rat hippocampus and cortex](https://springerlink3.metapress.com/content/91885487327u56w5/). [Environmentalist](https://springerlink3.metapress.com/content/0251-1088/). 28: 458-465, 2008a.

Nittby, H., Grafström, G., Tian, D.P., [Malmgren](https://pubmed.ncbi.nlm.nih.gov/?sort=pubdate&term=Malmgren+L&cauthor_id=18044737), L.,  [Brun](https://pubmed.ncbi.nlm.nih.gov/?sort=pubdate&term=Brun+A&cauthor_id=18044737), A.,  [Persson](https://pubmed.ncbi.nlm.nih.gov/?sort=pubdate&term=Persson+BR&cauthor_id=18044737), B.R.R.,  [Salford](https://pubmed.ncbi.nlm.nih.gov/?sort=pubdate&term=Salford+LG&cauthor_id=18044737), L.G.,  [Eberhardt](https://pubmed.ncbi.nlm.nih.gov/?sort=pubdate&term=Eberhardt+J&cauthor_id=18044737), J. [Cognitive impairment in rats after long-term exposure to GSM-900 mobile phone radiation.](https://pubmed.ncbi.nlm.nih.gov/18044737/) Bioelectromagnetics. 29:219-232, 2008b.

[Odacı E](http://www.ncbi.nlm.nih.gov/pubmed/?term=Odac%C4%B1%20E%5BAuthor%5D&cauthor=true&cauthor_uid=25786704), [Özyılmaz C](http://www.ncbi.nlm.nih.gov/pubmed/?term=%C3%96zy%C4%B1lmaz%20C%5BAuthor%5D&cauthor=true&cauthor_uid=25786704). Exposure to a 900 MHz electromagnetic field for one hour a day over 30 days does change the histopathology and biochemistry of the rat testis. [Int J Radiat Biol.](http://www.ncbi.nlm.nih.gov/pubmed/25786704?dopt=Abstract) 91(7):541-554, 2015.

Odacı E, Unal D, Mercantepe T, Topal Z, Hancı H, Türedi S, Erol H, Mungan S, Kaya H, Colakoğlu S. Pathological effects of prenatal exposure to a 900 MHz electromagnetic field on the 21-day-old male rat kidney. Biotech Histochem. 90(2):93-101, 2015.

[Odacı, E](https://www.ncbi.nlm.nih.gov/pubmed/?term=Odac%C4%B1%20E%5BAuthor%5D&cauthor=true&cauthor_uid=26472053)., [Hancı ,H](https://www.ncbi.nlm.nih.gov/pubmed/?term=Hanc%C4%B1%20H%5BAuthor%5D&cauthor=true&cauthor_uid=26472053)., [Yuluğ, E](https://www.ncbi.nlm.nih.gov/pubmed/?term=Yulu%C4%9F%20E%5BAuthor%5D&cauthor=true&cauthor_uid=26472053)., [Türedi, S](https://www.ncbi.nlm.nih.gov/pubmed/?term=T%C3%BCredi%20S%5BAuthor%5D&cauthor=true&cauthor_uid=26472053)., [Aliyazıcıoğlu, Y](https://www.ncbi.nlm.nih.gov/pubmed/?term=Aliyaz%C4%B1c%C4%B1o%C4%9Flu%20Y%5BAuthor%5D&cauthor=true&cauthor_uid=26472053)., [Kaya, H](https://www.ncbi.nlm.nih.gov/pubmed/?term=Kaya%20H%5BAuthor%5D&cauthor=true&cauthor_uid=26472053)., [Çolakoğlu S](https://www.ncbi.nlm.nih.gov/pubmed/?term=%C3%87olako%C4%9Flu%20S%5BAuthor%5D&cauthor=true&cauthor_uid=26472053). Effects of prenatal exposure to a 900 MHz electromagnetic field on 60-day-old rat testis and epididymal sperm quality. [Biotech Histochem.](https://www.ncbi.nlm.nih.gov/pubmed/26472053) 91:9-19, 2016.

[Okatan](https://pubmed.ncbi.nlm.nih.gov/?sort=date&term=Okatan+D%C3%96&cauthor_id=29268055) [DÖ](https://pubmed.ncbi.nlm.nih.gov/29268055/#affiliation-1),  [Kaya](https://pubmed.ncbi.nlm.nih.gov/?sort=date&term=Kaya+H&cauthor_id=29268055) [H](https://pubmed.ncbi.nlm.nih.gov/29268055/#affiliation-2),  [Aliyazıcıoğlu](https://pubmed.ncbi.nlm.nih.gov/?sort=date&term=Aliyaz%C4%B1c%C4%B1o%C4%9Flu+Y&cauthor_id=29268055) [Y](https://pubmed.ncbi.nlm.nih.gov/29268055/#affiliation-3), [Demir](https://pubmed.ncbi.nlm.nih.gov/?sort=date&term=Demir+S&cauthor_id=29268055) [S](https://pubmed.ncbi.nlm.nih.gov/29268055/#affiliation-4),  [Çolakoğlu](https://pubmed.ncbi.nlm.nih.gov/?sort=date&term=%C3%87olako%C4%9Flu+S&cauthor_id=29268055) [S](https://pubmed.ncbi.nlm.nih.gov/29268055/#affiliation-5),  [Odacı](https://pubmed.ncbi.nlm.nih.gov/?sort=date&term=Odac%C4%B1+E&cauthor_id=29268055) [E.](https://pubmed.ncbi.nlm.nih.gov/29268055/#affiliation-6) Continuous 900-megahertz electromagnetic field applied in middle and late-adolescence causes qualitative and quantitative changes in the ovarian morphology, tissue and blood biochemistry of the rat.  Int J Radiat Biol 94(2):186-198, 2018.

[Okatan DÖ](https://www.ncbi.nlm.nih.gov/pubmed/?term=Okatan%20D%C3%96%5BAuthor%5D&cauthor=true&cauthor_uid=31017002), [Kulaber A](https://www.ncbi.nlm.nih.gov/pubmed/?term=Kulaber%20A%5BAuthor%5D&cauthor=true&cauthor_uid=31017002), [Kerimoglu G](https://www.ncbi.nlm.nih.gov/pubmed/?term=Kerimoglu%20G%5BAuthor%5D&cauthor=true&cauthor_uid=31017002), [Odacı E](https://www.ncbi.nlm.nih.gov/pubmed/?term=Odac%C4%B1%20E%5BAuthor%5D&cauthor=true&cauthor_uid=31017002).Altered morphology and biochemistry of the female rat liver following 900 megahertz electromagnetic field exposure during mid to late adolescence. [Biotech Histochem.](https://www.ncbi.nlm.nih.gov/pubmed/31017002) 94(6):420-428, 2019.

[Oksay T](http://www.ncbi.nlm.nih.gov/pubmed?term=Oksay%20T%5BAuthor%5D&cauthor=true&cauthor_uid=23145464), [Naziroğlu M](http://www.ncbi.nlm.nih.gov/pubmed?term=Naziro%C4%9Flu%20M%5BAuthor%5D&cauthor=true&cauthor_uid=23145464), [Doğan S](http://www.ncbi.nlm.nih.gov/pubmed?term=Do%C4%9Fan%20S%5BAuthor%5D&cauthor=true&cauthor_uid=23145464), [Güzel A](http://www.ncbi.nlm.nih.gov/pubmed?term=G%C3%BCzel%20A%5BAuthor%5D&cauthor=true&cauthor_uid=23145464), [Gümral N](http://www.ncbi.nlm.nih.gov/pubmed?term=G%C3%BCmral%20N%5BAuthor%5D&cauthor=true&cauthor_uid=23145464), [Koşar PA](http://www.ncbi.nlm.nih.gov/pubmed?term=Ko%C5%9Far%20PA%5BAuthor%5D&cauthor=true&cauthor_uid=23145464). Protective effects of melatonin against oxidative injury in rat testis induced by wireless (2.45 GHz) devices. [Andrologia.](http://www.ncbi.nlm.nih.gov/pubmed/23145464) 46(1):65-72, 2014.

[Özdemir](https://pubmed.ncbi.nlm.nih.gov/?sort=pubdate&term=%C3%96zdemir+E&cauthor_id=33653184) [E](https://pubmed.ncbi.nlm.nih.gov/33653184/#affiliation-1),  [Çömelekoğlu](https://pubmed.ncbi.nlm.nih.gov/?sort=pubdate&term=%C3%87%C3%B6meleko%C4%9Flu+%C3%9C&cauthor_id=33653184) [U](https://pubmed.ncbi.nlm.nih.gov/33653184/#affiliation-1),  [Degirmenci](https://pubmed.ncbi.nlm.nih.gov/?sort=pubdate&term=Degirmenci+E&cauthor_id=33653184) E, [Bayrak](https://pubmed.ncbi.nlm.nih.gov/?sort=pubdate&term=Bayrak+G&cauthor_id=33653184) [G](https://pubmed.ncbi.nlm.nih.gov/33653184/#affiliation-3), [Yildirim](https://pubmed.ncbi.nlm.nih.gov/?sort=pubdate&term=Yildirim+M&cauthor_id=33653184) [M](https://pubmed.ncbi.nlm.nih.gov/33653184/#affiliation-4),  [Ergenoglu](https://pubmed.ncbi.nlm.nih.gov/?sort=pubdate&term=Ergenoglu+T&cauthor_id=33653184) [t](https://pubmed.ncbi.nlm.nih.gov/33653184/#affiliation-5) T,  Yilmaz [BC](https://pubmed.ncbi.nlm.nih.gov/33653184/#affiliation-3),   [Yalin](https://pubmed.ncbi.nlm.nih.gov/?sort=pubdate&term=Yalin+S&cauthor_id=33653184) [S](https://pubmed.ncbi.nlm.nih.gov/33653184/#affiliation-4),  [Koyuncu](https://pubmed.ncbi.nlm.nih.gov/?sort=pubdate&term=Koyuncu+DD&cauthor_id=33653184) [DD](https://pubmed.ncbi.nlm.nih.gov/33653184/#affiliation-5), [Ozbay](https://pubmed.ncbi.nlm.nih.gov/?sort=pubdate&term=Ozbay+E&cauthor_id=33653184) [E](https://pubmed.ncbi.nlm.nih.gov/33653184/#affiliation-7). The effect of 4.5 G (LTE Advanced-Pro network) mobile phone radiation on the optic nerve. Cutan Ocul Toxicol 40(3):198-206, 2021.

[Ozguner F](http://www.ncbi.nlm.nih.gov/entrez/query.fcgi?db=pubmed&cmd=Search&term=%22Ozguner+F%22%5BAuthor%5D), [Oktem F](http://www.ncbi.nlm.nih.gov/entrez/query.fcgi?db=pubmed&cmd=Search&term=%22Oktem+F%22%5BAuthor%5D), [Ayata A](http://www.ncbi.nlm.nih.gov/entrez/query.fcgi?db=pubmed&cmd=Search&term=%22Ayata+A%22%5BAuthor%5D), [Koyu A](http://www.ncbi.nlm.nih.gov/entrez/query.fcgi?db=pubmed&cmd=Search&term=%22Koyu+A%22%5BAuthor%5D), [Yilmaz HR](http://www.ncbi.nlm.nih.gov/entrez/query.fcgi?db=pubmed&cmd=Search&term=%22Yilmaz+HR%22%5BAuthor%5D). A novel antioxidant agent caffeic acid phenethyl ester prevents long-term mobile phone exposure-induced renal impairment in rat. Prognostic value of malondialdehyde, N-acetyl-beta-D-glucosaminidase and nitric oxide determination. Mol Cell Biochem. 277(1-2):73-80, 2005a.

[Ozguner F](http://www.ncbi.nlm.nih.gov/entrez/query.fcgi?db=pubmed&cmd=Search&term=%22Ozguner+F%22%5BAuthor%5D), [Altinbas A](http://www.ncbi.nlm.nih.gov/entrez/query.fcgi?db=pubmed&cmd=Search&term=%22Altinbas+A%22%5BAuthor%5D), [Ozaydin M](http://www.ncbi.nlm.nih.gov/entrez/query.fcgi?db=pubmed&cmd=Search&term=%22Ozaydin+M%22%5BAuthor%5D), [Dogan A](http://www.ncbi.nlm.nih.gov/entrez/query.fcgi?db=pubmed&cmd=Search&term=%22Dogan+A%22%5BAuthor%5D), [Vural H](http://www.ncbi.nlm.nih.gov/entrez/query.fcgi?db=pubmed&cmd=Search&term=%22Vural+H%22%5BAuthor%5D), [Kisioglu AN](http://www.ncbi.nlm.nih.gov/entrez/query.fcgi?db=pubmed&cmd=Search&term=%22Kisioglu+AN%22%5BAuthor%5D), [Cesur G](http://www.ncbi.nlm.nih.gov/entrez/query.fcgi?db=pubmed&cmd=Search&term=%22Cesur+G%22%5BAuthor%5D), [Yildirim NG](http://www.ncbi.nlm.nih.gov/entrez/query.fcgi?db=pubmed&cmd=Search&term=%22Yildirim+NG%22%5BAuthor%5D). Mobile phone-induced myocardial oxidative stress: protection by a novel antioxidant agent caffeic acid phenethyl ester. [Toxicol Ind Health.](javascript:AL_get(this,%20'jour',%20'Toxicol%20Ind%20Health.');) 21:223-230, 2005b.

[Ozgur, E](http://www.ncbi.nlm.nih.gov/pubmed?term=%22Ozgur%20E%22%5BAuthor%5D)., [Güler, G](http://www.ncbi.nlm.nih.gov/pubmed?term=%22G%C3%BCler%20G%22%5BAuthor%5D)., [Seyhan, N](http://www.ncbi.nlm.nih.gov/pubmed?term=%22Seyhan%20N%22%5BAuthor%5D). Mobile phone radiation-induced free radical damage in the liver is inhibited by the antioxidants n-acetyl cysteine and epigallocatechin-gallate. [Int J Radiat Biol.](javascript:AL_get(this,%20'jour',%20'Int%20J%20Radiat%20Biol.');) 86:935-945, 2010.

[Ozlem Nisbet H](http://www.ncbi.nlm.nih.gov/pubmed?term=Ozlem%20Nisbet%20H%5BAuthor%5D&cauthor=true&cauthor_uid=22130559), [Nisbet C](http://www.ncbi.nlm.nih.gov/pubmed?term=Nisbet%20C%5BAuthor%5D&cauthor=true&cauthor_uid=22130559), [Akar A](http://www.ncbi.nlm.nih.gov/pubmed?term=Akar%20A%5BAuthor%5D&cauthor=true&cauthor_uid=22130559), [Cevik M](http://www.ncbi.nlm.nih.gov/pubmed?term=Cevik%20M%5BAuthor%5D&cauthor=true&cauthor_uid=22130559), [Karayigit MO](http://www.ncbi.nlm.nih.gov/pubmed?term=Karayigit%20MO%5BAuthor%5D&cauthor=true&cauthor_uid=22130559). Effects of exposure to electromagnetic field (1.8/0.9 GHz) on testicular function and structure in growing rats. [Res Vet Sci.](http://www.ncbi.nlm.nih.gov/pubmed/22130559) 93(2):1001-1005, 2012.

[Ozorak A](http://www.ncbi.nlm.nih.gov/pubmed?term=Ozorak%20A%5BAuthor%5D&cauthor=true&cauthor_uid=24101576), [Nazıroğlu M](http://www.ncbi.nlm.nih.gov/pubmed?term=Naz%C4%B1ro%C4%9Flu%20M%5BAuthor%5D&cauthor=true&cauthor_uid=24101576), [Celik O](http://www.ncbi.nlm.nih.gov/pubmed?term=Celik%20O%5BAuthor%5D&cauthor=true&cauthor_uid=24101576), [Yüksel M](http://www.ncbi.nlm.nih.gov/pubmed?term=Y%C3%BCksel%20M%5BAuthor%5D&cauthor=true&cauthor_uid=24101576), [Ozçelik D](http://www.ncbi.nlm.nih.gov/pubmed?term=Oz%C3%A7elik%20D%5BAuthor%5D&cauthor=true&cauthor_uid=24101576), [Ozkaya MO](http://www.ncbi.nlm.nih.gov/pubmed?term=Ozkaya%20MO%5BAuthor%5D&cauthor=true&cauthor_uid=24101576), [Cetin H](http://www.ncbi.nlm.nih.gov/pubmed?term=Cetin%20H%5BAuthor%5D&cauthor=true&cauthor_uid=24101576), [Kahya MC](http://www.ncbi.nlm.nih.gov/pubmed?term=Kahya%20MC%5BAuthor%5D&cauthor=true&cauthor_uid=24101576), [Kose SA](http://www.ncbi.nlm.nih.gov/pubmed?term=Kose%20SA%5BAuthor%5D&cauthor=true&cauthor_uid=24101576). Wi-Fi (2.45 GHz)- and Mobile Phone (900 and 1800 MHz)-Induced Risks on Oxidative Stress and Elements in Kidney and Testis of Rats During Pregnancy and the Development of Offspring. [Biol Trace Elem Res.](http://www.ncbi.nlm.nih.gov/pubmed/24101576) 156(1-3):221-229, 2013.

Ozyilmaz C, Oktay MF, Dasdag S, Ulukaya E, Genel ME, Tansuker HD, Emre F, Yeğin K. Evaluation of the Thyroids of Offsprings Exposed to 2450 MHz Radiofrequency Radiation During Pregnancy: A Sixth Month Data. J Inter Dental Med Res. 17(2): 925-930, 2024.

Özyılmaz C, Daşdağ S, Oktay MF, Ulukaya E, Erkısa Genel M, Emre F, Yeğin K. One-year follow-up of thyroid status in rats exposed to 2.45 Ghz radiofrequency radiation during the prenatal period. Electromagn Biol Med. 2025 Oct 22:1-10. doi: 10.1080/15368378.2025.2577318.

[Pandey](https://pubmed.ncbi.nlm.nih.gov/?sort=date&term=Pandey+N&cauthor_id=29562845) [N](https://pubmed.ncbi.nlm.nih.gov/29562845/#affiliation-1), [Giri](https://pubmed.ncbi.nlm.nih.gov/?sort=date&term=Giri+S&cauthor_id=29562845) [S.](https://pubmed.ncbi.nlm.nih.gov/29562845/#affiliation-1) Melatonin attenuates radiofrequency radiation (900 MHz)-induced oxidative stress, DNA damage and cell cycle arrest in germ cells of male Swiss albino mice. Toxicol Ind Health 34(5):315-327, 2018.

[Pandey, N](https://www.ncbi.nlm.nih.gov/pubmed/?term=Pandey%20N%5BAuthor%5D&cauthor=true&cauthor_uid=27738269)., [Giri, S](https://www.ncbi.nlm.nih.gov/pubmed/?term=Giri%20S%5BAuthor%5D&cauthor=true&cauthor_uid=27738269)., [Das, S](https://www.ncbi.nlm.nih.gov/pubmed/?term=Das%20S%5BAuthor%5D&cauthor=true&cauthor_uid=27738269)., [Upadhaya, P](https://www.ncbi.nlm.nih.gov/pubmed/?term=Upadhaya%20P%5BAuthor%5D&cauthor=true&cauthor_uid=27738269). Radiofrequency radiation (900 MHz)-induced DNA damage and cell cycle arrest in testicular germ cells in swiss albino mice. [Toxicol Ind Health.](https://www.ncbi.nlm.nih.gov/pubmed/27738269) 33:33-384, 2017.

[Pavicić](https://pubmed.ncbi.nlm.nih.gov/?sort=date&term=Pavici%C4%87+I&cauthor_id=16832969), [I](https://pubmed.ncbi.nlm.nih.gov/16832969/#affiliation-1).,  [Trosić](https://pubmed.ncbi.nlm.nih.gov/?sort=date&term=Trosi%C4%87+I&cauthor_id=16832969), I.,  [Sarolić](https://pubmed.ncbi.nlm.nih.gov/?sort=date&term=Saroli%C4%87+A&cauthor_id=16832969), A. Comparison of 864 MHz and 935 MHz microwave radiation effects on cell culture. Arh Hig Rada Toksikol. 57:149-54, 2006.

P[elletier, A](http://www.ncbi.nlm.nih.gov/pubmed?term=Pelletier%20A%5BAuthor%5D&cauthor=true&cauthor_uid=23143821)., [Delanaud, S](http://www.ncbi.nlm.nih.gov/pubmed?term=Delanaud%20S%5BAuthor%5D&cauthor=true&cauthor_uid=23143821)., [Décima, P](http://www.ncbi.nlm.nih.gov/pubmed?term=D%C3%A9cima%20P%5BAuthor%5D&cauthor=true&cauthor_uid=23143821)., [Thuroczy, G](http://www.ncbi.nlm.nih.gov/pubmed?term=Thuroczy%20G%5BAuthor%5D&cauthor=true&cauthor_uid=23143821)., [de Seze, R](http://www.ncbi.nlm.nih.gov/pubmed?term=de%20Seze%20R%5BAuthor%5D&cauthor=true&cauthor_uid=23143821)., [Cerri, M](http://www.ncbi.nlm.nih.gov/pubmed?term=Cerri%20M%5BAuthor%5D&cauthor=true&cauthor_uid=23143821)., [Bach, V](http://www.ncbi.nlm.nih.gov/pubmed?term=Bach%20V%5BAuthor%5D&cauthor=true&cauthor_uid=23143821)., [Libert, J.P](http://www.ncbi.nlm.nih.gov/pubmed?term=Libert%20JP%5BAuthor%5D&cauthor=true&cauthor_uid=23143821)., [Loos, N](http://www.ncbi.nlm.nih.gov/pubmed?term=Loos%20N%5BAuthor%5D&cauthor=true&cauthor_uid=23143821). Effects of chronic exposure to radiofrequency electromagnetic fields on energy balance in developing rats. [Environ Sci Pollut Res Int.](http://www.ncbi.nlm.nih.gov/pubmed/23143821) 20:2735-2746, 2013.

[Pérez-Castejón, C](http://www.ncbi.nlm.nih.gov/pubmed?term=%22P%C3%A9rez-Castej%C3%B3n%20C%22%5BAuthor%5D&itool=EntrezSystem2.PEntrez.Pubmed.Pubmed_ResultsPanel.Pubmed_RVAbstract)., [Pérez-Bruzón, R.N](http://www.ncbi.nlm.nih.gov/pubmed?term=%22P%C3%A9rez-Bruz%C3%B3n%20RN%22%5BAuthor%5D&itool=EntrezSystem2.PEntrez.Pubmed.Pubmed_ResultsPanel.Pubmed_RVAbstract)., [Llorente, M](http://www.ncbi.nlm.nih.gov/pubmed?term=%22Llorente%20M%22%5BAuthor%5D&itool=EntrezSystem2.PEntrez.Pubmed.Pubmed_ResultsPanel.Pubmed_RVAbstract)., [Pes, N](http://www.ncbi.nlm.nih.gov/pubmed?term=%22Pes%20N%22%5BAuthor%5D&itool=EntrezSystem2.PEntrez.Pubmed.Pubmed_ResultsPanel.Pubmed_RVAbstract)., [Lacasa, C](http://www.ncbi.nlm.nih.gov/pubmed?term=%22Lacasa%20C%22%5BAuthor%5D&itool=EntrezSystem2.PEntrez.Pubmed.Pubmed_ResultsPanel.Pubmed_RVAbstract)., [Figols, T](http://www.ncbi.nlm.nih.gov/pubmed?term=%22Figols%20T%22%5BAuthor%5D&itool=EntrezSystem2.PEntrez.Pubmed.Pubmed_ResultsPanel.Pubmed_RVAbstract)., [Lahoz, M](http://www.ncbi.nlm.nih.gov/pubmed?term=%22Lahoz%20M%22%5BAuthor%5D&itool=EntrezSystem2.PEntrez.Pubmed.Pubmed_ResultsPanel.Pubmed_RVAbstract)., [Maestú, C](http://www.ncbi.nlm.nih.gov/pubmed?term=%22Maest%C3%BA%20C%22%5BAuthor%5D&itool=EntrezSystem2.PEntrez.Pubmed.Pubmed_ResultsPanel.Pubmed_RVAbstract)., [Vera-Gil, A](http://www.ncbi.nlm.nih.gov/pubmed?term=%22Vera-Gil%20A%22%5BAuthor%5D&itool=EntrezSystem2.PEntrez.Pubmed.Pubmed_ResultsPanel.Pubmed_RVAbstract)., [Del Moral, A](http://www.ncbi.nlm.nih.gov/pubmed?term=%22Del%20Moral%20A%22%5BAuthor%5D&itool=EntrezSystem2.PEntrez.Pubmed.Pubmed_ResultsPanel.Pubmed_RVAbstract)., [Azanza, M.J](http://www.ncbi.nlm.nih.gov/pubmed?term=%22Azanza%20MJ%22%5BAuthor%5D&itool=EntrezSystem2.PEntrez.Pubmed.Pubmed_ResultsPanel.Pubmed_RVAbstract). Exposure to ELF-pulse modulated X band microwaves increases in vitro human astrocytoma cell proliferation. [Histol Histopathol.](javascript:AL_get(this,%20'jour',%20'Histol%20Histopathol.');) 24: 1551-1561, 2009.

[Perov, S](https://www.ncbi.nlm.nih.gov/pubmed/?term=Perov%252520S%25255BAuthor%25255D&cauthor=true&cauthor_uid=31642089)., [Rubtsova, N](https://www.ncbi.nlm.nih.gov/pubmed/?term=Rubtsova%252520N%25255BAuthor%25255D&cauthor=true&cauthor_uid=31642089)., [Balzano, Q](https://www.ncbi.nlm.nih.gov/pubmed/?term=Balzano%252520Q%25255BAuthor%25255D&cauthor=true&cauthor_uid=31642089). Effects of 171 MHz low-intensity electromagnetic field on glucocorticoid and mineral corticoid activity of the adrenal glands of rats. [Bioelectromagnetics.](https://www.ncbi.nlm.nih.gov/pubmed/31642089) 40:578-587, 2019.

Persson, B.R.R., Salford, L.G., Brun, A, Blood-brain barrier permeability in rats exposed to electromagnetic fields used in wireless communication. Wireless Network. 3:455-461, 1997.

Phillips, J.L., Ivaschuk, O., Ishida-Jones, T., Jones, R.A., Campbell-Beachler, M., Haggren, W. DNA damage in Molt-4 T- lymphoblastoid cells exposed to cellular telephone radiofrequency fields *in vitro.* Bioelectrochem. Bioenerg. 45:103–110, 1998.

[Piccinetti, C.C](https://www.ncbi.nlm.nih.gov/pubmed/?term=Piccinetti%252520CC%25255BAuthor%25255D&cauthor=true&cauthor_uid=29477917)., [De Leo, A](https://www.ncbi.nlm.nih.gov/pubmed/?term=De%252520Leo%252520A%25255BAuthor%25255D&cauthor=true&cauthor_uid=29477917)., [Cosoli, G](https://www.ncbi.nlm.nih.gov/pubmed/?term=Cosoli%252520G%25255BAuthor%25255D&cauthor=true&cauthor_uid=29477917)., [Scalise, L](https://www.ncbi.nlm.nih.gov/pubmed/?term=Scalise%252520L%25255BAuthor%25255D&cauthor=true&cauthor_uid=29477917)., [Randazzo, B](https://www.ncbi.nlm.nih.gov/pubmed/?term=Randazzo%252520B%25255BAuthor%25255D&cauthor=true&cauthor_uid=29477917)., [Cerri, G](https://www.ncbi.nlm.nih.gov/pubmed/?term=Cerri%252520G%25255BAuthor%25255D&cauthor=true&cauthor_uid=29477917)., [Olivotto, I](https://www.ncbi.nlm.nih.gov/pubmed/?term=Olivotto%252520I%25255BAuthor%25255D&cauthor=true&cauthor_uid=29477917). Measurement of the 100 MHz EMF radiation in vivo effects on zebrafish D. rerio embryonic development: A multidisciplinary study. [Ecotoxicol Environ Saf.](https://www.ncbi.nlm.nih.gov/pubmed/29477917) 154:268-279, 2018.

[Porcher](https://pubmed.ncbi.nlm.nih.gov/?sort=date&term=Porcher+A&cauthor_id=37210775) [A](https://pubmed.ncbi.nlm.nih.gov/37210775/#full-view-affiliation-1),  [Girard](https://pubmed.ncbi.nlm.nih.gov/?sort=date&term=Girard+S&cauthor_id=37210775) [S](https://pubmed.ncbi.nlm.nih.gov/37210775/#full-view-affiliation-1),  [Bonnet](https://pubmed.ncbi.nlm.nih.gov/?sort=date&term=Bonnet+P&cauthor_id=37210775) [P](https://pubmed.ncbi.nlm.nih.gov/37210775/#full-view-affiliation-1),  [Rouveure](https://pubmed.ncbi.nlm.nih.gov/?sort=date&term=Rouveure+R&cauthor_id=37210775) [R](https://pubmed.ncbi.nlm.nih.gov/37210775/#full-view-affiliation-2),  [Guérin](https://pubmed.ncbi.nlm.nih.gov/?sort=date&term=Gu%C3%A9rin+V&cauthor_id=37210775) [V](https://pubmed.ncbi.nlm.nih.gov/37210775/#full-view-affiliation-3), [Paladian](https://pubmed.ncbi.nlm.nih.gov/?sort=date&term=Paladian+F&cauthor_id=37210775) [F](https://pubmed.ncbi.nlm.nih.gov/37210775/#full-view-affiliation-1),  [Vian](https://pubmed.ncbi.nlm.nih.gov/?sort=date&term=Vian+A&cauthor_id=37210775) [A.](https://pubmed.ncbi.nlm.nih.gov/37210775/#full-view-affiliation-4) Non thermal 2.45 GHz electromagnetic exposure causes rapid changes in Arabidopsis thaliana metabolism. J Plant Physiol 286:153999, 2023.

[Postaci, I](https://www.ncbi.nlm.nih.gov/pubmed/?term=Postaci%252520I%25255BAuthor%25255D&cauthor=true&cauthor_uid=30160155)., [Coskun, O](https://www.ncbi.nlm.nih.gov/pubmed/?term=Coskun%252520O%25255BAuthor%25255D&cauthor=true&cauthor_uid=30160155)., [Senol, N](https://www.ncbi.nlm.nih.gov/pubmed/?term=Senol%252520N%25255BAuthor%25255D&cauthor=true&cauthor_uid=30160155)., [Aslankoc, R](https://www.ncbi.nlm.nih.gov/pubmed/?term=Aslankoc%252520R%25255BAuthor%25255D&cauthor=true&cauthor_uid=30160155)., [Comlekci, S](https://www.ncbi.nlm.nih.gov/pubmed/?term=Comlekci%252520S%25255BAuthor%25255D&cauthor=true&cauthor_uid=30160155). The physiopathological effects of quercetin on oxidative stress in radiation of 4.5 g mobile phone exposed liver tissue of rat. [Bratisl Lek Listy.](https://www.ncbi.nlm.nih.gov/pubmed/?term=postaci+and+mobile+phone) 119:481-489, 2018.

Pyrpasopoulou, A., Kotoula, V., Cheva, A., Hytiroglou, P., Nikolakaki, E., Magras, I.N., Xenos, T.D., Tsiboukis, T.D., Karkavelas, G. Bone morphogenetic protein expression in newborn rat kidneys after prenatal exposure to radiofrequency radiation. Bioelectromagnetics. 25:216-227, 2004.

Qin F, Zhang J, Cao H, Guo W, Chen L, Shen O, Sun J, Yi C, Li J, Wang J, Tong J. Circadian alterations of reproductive functional markers in male rats exposed to 1800-MHz radiofrequency field. Chronobiol Int. 31(1):123-133, 2014.

Qin, F., Cao, H., Yuan, H., Guo, W., Pei, H., Cao, Y., Tong, J. [1800 MHz radiofrequency fields inhibits testosterone production via CaMKI /RORα pathway.](https://pubmed.ncbi.nlm.nih.gov/30125682/) Reprod Toxicol. 81:229-236, 2018.

[Qin F](https://www.ncbi.nlm.nih.gov/pubmed/?term=Qin%20F%5BAuthor%5D&cauthor=true&cauthor_uid=31296989), [Shen T](https://www.ncbi.nlm.nih.gov/pubmed/?term=Shen%20T%5BAuthor%5D&cauthor=true&cauthor_uid=31296989), [Cao H](https://www.ncbi.nlm.nih.gov/pubmed/?term=Cao%20H%5BAuthor%5D&cauthor=true&cauthor_uid=31296989), [Qian J](https://www.ncbi.nlm.nih.gov/pubmed/?term=Qian%20J%5BAuthor%5D&cauthor=true&cauthor_uid=31296989), [Zou D](https://www.ncbi.nlm.nih.gov/pubmed/?term=Zou%20D%5BAuthor%5D&cauthor=true&cauthor_uid=31296989), [Ye M](https://www.ncbi.nlm.nih.gov/pubmed/?term=Ye%20M%5BAuthor%5D&cauthor=true&cauthor_uid=31296989), [Pei H](https://www.ncbi.nlm.nih.gov/pubmed/?term=Pei%20H%5BAuthor%5D&cauthor=true&cauthor_uid=31296989). CeO_2_NPs relieve radiofrequency radiation, improve testosterone synthesis, and clock gene expression in Leydig cells by enhancing antioxidation. [Int J Nanomedicine.](https://www.ncbi.nlm.nih.gov/pubmed/31296989) 14:4601-4611, 2019.

[Rafati, A](https://www.ncbi.nlm.nih.gov/pubmed/?term=Rafati%252520A%25255BAuthor%25255D&cauthor=true&cauthor_uid=26396969)., [Rahimi, S](https://www.ncbi.nlm.nih.gov/pubmed/?term=Rahimi%252520S%25255BAuthor%25255D&cauthor=true&cauthor_uid=26396969)., [Talebi, A](https://www.ncbi.nlm.nih.gov/pubmed/?term=Talebi%252520A%25255BAuthor%25255D&cauthor=true&cauthor_uid=26396969)., [Soleimani, A](https://www.ncbi.nlm.nih.gov/pubmed/?term=Soleimani%252520A%25255BAuthor%25255D&cauthor=true&cauthor_uid=26396969)., [Haghani, M](https://www.ncbi.nlm.nih.gov/pubmed/?term=Haghani%252520M%25255BAuthor%25255D&cauthor=true&cauthor_uid=26396969)., [Mortazavi, S.M](https://www.ncbi.nlm.nih.gov/pubmed/?term=Mortazavi%252520SM%25255BAuthor%25255D&cauthor=true&cauthor_uid=26396969). Exposure to radiofrequency radiation emitted from common mobile phone jammers alters the pattern of muscle contractions: an animal model study. [J Biomed Phys Eng.](https://www.ncbi.nlm.nih.gov/pubmed/26396969) 5:133-142, 2015.

[Sagioglou NE](http://www.ncbi.nlm.nih.gov/pubmed/?term=Sagioglou%20NE%5BAuthor%5D&cauthor=true&cauthor_uid=25333897), [Manta AK](http://www.ncbi.nlm.nih.gov/pubmed/?term=Manta%20AK%5BAuthor%5D&cauthor=true&cauthor_uid=25333897), [Giannarakis IK](http://www.ncbi.nlm.nih.gov/pubmed/?term=Giannarakis%20IK%5BAuthor%5D&cauthor=true&cauthor_uid=25333897), [Skouroliakou AS](http://www.ncbi.nlm.nih.gov/pubmed/?term=Skouroliakou%20AS%5BAuthor%5D&cauthor=true&cauthor_uid=25333897), [Margaritis LH](http://www.ncbi.nlm.nih.gov/pubmed/?term=Margaritis%20LH%5BAuthor%5D&cauthor=true&cauthor_uid=25333897). Apoptotic cell death during Drosophila oogenesis is differentially increased by electromagnetic radiation depending on modulation, intensity and duration of exposure. [Electromagn Biol Med.](http://www.ncbi.nlm.nih.gov/pubmed/25333897) 35:40-53, 2016.

Salford, L.G., Brun, A.R., Eberhardt, J.L., Malmgren, L., Persson, B.R.R. Nerve cell damage in mammalian brain after exposure to microwaves from GSM mobile phones. Environ Health Persp. 111:881-883, 2003.

[Sangun O](http://www.ncbi.nlm.nih.gov/pubmed?term=Sangun%20O%5BAuthor%5D&cauthor=true&cauthor_uid=24460416), [Dundar B](http://www.ncbi.nlm.nih.gov/pubmed?term=Dundar%20B%5BAuthor%5D&cauthor=true&cauthor_uid=24460416), [Darici H](http://www.ncbi.nlm.nih.gov/pubmed?term=Darici%20H%5BAuthor%5D&cauthor=true&cauthor_uid=24460416), [Comlekci S](http://www.ncbi.nlm.nih.gov/pubmed?term=Comlekci%20S%5BAuthor%5D&cauthor=true&cauthor_uid=24460416), [Doguc DK](http://www.ncbi.nlm.nih.gov/pubmed?term=Doguc%20DK%5BAuthor%5D&cauthor=true&cauthor_uid=24460416), [Celik S](http://www.ncbi.nlm.nih.gov/pubmed?term=Celik%20S%5BAuthor%5D&cauthor=true&cauthor_uid=24460416). The effects of long-term exposure to a 2450 MHz electromagnetic field on growth and pubertal development in female Wistar rats. [Electromagn Biol Med.](http://www.ncbi.nlm.nih.gov/pubmed/24460416) 34(1):63-71, 2015.

Sannino A, Zeni O, Romeo S, Massa R, Gialanella G, Grossi G,  [Manti](https://pubmed.ncbi.nlm.nih.gov/?sort=date&term=Manti+L&cauthor_id=23979077) L, [Vijayalaxmi](https://pubmed.ncbi.nlm.nih.gov/?sort=date&term=Vijayalaxmi&cauthor_id=23979077),  [Scarfì](https://pubmed.ncbi.nlm.nih.gov/?sort=date&term=Scarf%C3%AC+MR&cauthor_id=23979077) MR. Adaptive response in human blood lymphocytes exposed to non-ionizing radiofrequency fields: resistance to ionizing radiation-induced damage. J Radiat Res. 55: 210-217, 2014.

Sarimov, R., [Malmgren, L.O.G.](http://ieeexplore.ieee.org/search/quicksrchresult.jsp?queryText=(%252520malmgren%252520%252520l.%252520o.%252520g.%25253CIN%25253Eau)&valnm=+Malmgren%25252C+L.O.G.&ResultCount=15&SortField=pyr&SortOrder=desc&reqloc=au), [Markova, E.](http://ieeexplore.ieee.org/search/quicksrchresult.jsp?queryText=(%252520markova%252520%252520e.%25253CIN%25253Eau)&valnm=+Markova%25252C+E.&ResultCount=15&SortField=pyr&SortOrder=desc&reqloc=au),  [Persson, B.R.R.](http://ieeexplore.ieee.org/search/quicksrchresult.jsp?queryText=(%252520persson%252520%252520b.%252520r.%252520r.%25253CIN%25253Eau)&valnm=+Persson%25252C+B.R.R.&ResultCount=15&SortField=pyr&SortOrder=desc&reqloc=au),  [Belyaev, I.Y.](http://ieeexplore.ieee.org/search/quicksrchresult.jsp?queryText=(%252520belyaev%252520%252520i.%252520y.%25253CIN%25253Eau)&valnm=+Belyaev%25252C+I.Y.&ResultCount=15&SortField=pyr&SortOrder=desc&reqloc=au)  Nonthermal GSM microwaves affect chromatin conformation in human lymphocytes similar to heat shock. IEEE Trans Plasma Sci. 32:1600-1608, 2004.

[Schwarz, C](http://www.ncbi.nlm.nih.gov/sites/entrez?Db=pubmed&Cmd=Search&Term=%252522Schwarz%252520C%252522%25255BAuthor%25255D&itool=EntrezSystem2.PEntrez.Pubmed.Pubmed_ResultsPanel.Pubmed_DiscoveryPanel.Pubmed_RVAbstractPlus)., [Kratochvil, E](http://www.ncbi.nlm.nih.gov/sites/entrez?Db=pubmed&Cmd=Search&Term=%252522Kratochvil%252520E%252522%25255BAuthor%25255D&itool=EntrezSystem2.PEntrez.Pubmed.Pubmed_ResultsPanel.Pubmed_DiscoveryPanel.Pubmed_RVAbstractPlus)., [Pilger, A](http://www.ncbi.nlm.nih.gov/sites/entrez?Db=pubmed&Cmd=Search&Term=%252522Pilger%252520A%252522%25255BAuthor%25255D&itool=EntrezSystem2.PEntrez.Pubmed.Pubmed_ResultsPanel.Pubmed_DiscoveryPanel.Pubmed_RVAbstractPlus)., [Kuster, N](http://www.ncbi.nlm.nih.gov/sites/entrez?Db=pubmed&Cmd=Search&Term=%252522Kuster%252520N%252522%25255BAuthor%25255D&itool=EntrezSystem2.PEntrez.Pubmed.Pubmed_ResultsPanel.Pubmed_DiscoveryPanel.Pubmed_RVAbstractPlus)., [Adlkofer, F](http://www.ncbi.nlm.nih.gov/sites/entrez?Db=pubmed&Cmd=Search&Term=%252522Adlkofer%252520F%252522%25255BAuthor%25255D&itool=EntrezSystem2.PEntrez.Pubmed.Pubmed_ResultsPanel.Pubmed_DiscoveryPanel.Pubmed_RVAbstractPlus)., [Rüdiger, H.W](http://www.ncbi.nlm.nih.gov/sites/entrez?Db=pubmed&Cmd=Search&Term=%252522R%2525C3%2525BCdiger%252520HW%252522%25255BAuthor%25255D&itool=EntrezSystem2.PEntrez.Pubmed.Pubmed_ResultsPanel.Pubmed_DiscoveryPanel.Pubmed_RVAbstractPlus). Radiofrequency electromagnetic fields (UMTS, 1,950 MHz) induce genotoxic effects in vitro in human fibroblasts but not in lymphocytes. Int Arch Occup Environ Health. 81:755-767, 2008.

Schwartz, J.L., House, D.E., Mealing, G.A, Exposure of frog hearts to CW or amplitude-modulated VHF fields: selective efflux of calcium ions at 16 Hz. Bioelectromagnetics. 11:349-358, 1990.

Seewooruttun C, Bouguila B, Corona A, Delanaud S, Bodin R, Bach V, Desailloud R, Pelletier A. 5G Radiofrequency Exposure Reduces *PRDM16* and *C/EBP β* mRNA Expression, Two Key Biomarkers for Brown Adipogenesis. Int J Mol Sci. 26(6):2792, 2025.

[Sefidbakht, Y](http://www.ncbi.nlm.nih.gov/pubmed?term=Sefidbakht%20Y%5BAuthor%5D&cauthor=true&cauthor_uid=24886806)., [Moosavi-Movahedi, A.A](http://www.ncbi.nlm.nih.gov/pubmed?term=Moosavi-Movahedi%20AA%5BAuthor%5D&cauthor=true&cauthor_uid=24886806)., [Hosseinkhani, S](http://www.ncbi.nlm.nih.gov/pubmed?term=Hosseinkhani%20S%5BAuthor%5D&cauthor=true&cauthor_uid=24886806)., [Khodagholi, F](http://www.ncbi.nlm.nih.gov/pubmed?term=Khodagholi%20F%5BAuthor%5D&cauthor=true&cauthor_uid=24886806)., [Torkzadeh-Mahani, M](http://www.ncbi.nlm.nih.gov/pubmed?term=Torkzadeh-Mahani%20M%5BAuthor%5D&cauthor=true&cauthor_uid=24886806)., [Foolad, F](http://www.ncbi.nlm.nih.gov/pubmed?term=Foolad%20F%5BAuthor%5D&cauthor=true&cauthor_uid=24886806)., [Faraji-Dana, R](http://www.ncbi.nlm.nih.gov/pubmed?term=Faraji-Dana%20R%5BAuthor%5D&cauthor=true&cauthor_uid=24886806). Effects of 940 MHz EMF on bioluminescence and oxidative response of stable luciferase producing HEK cells. [Photochem Photobiol Sci.](http://www.ncbi.nlm.nih.gov/pubmed/24886806) 13:1082-1092, 2014.

Sepehrimanesh M, Saeb M, Nazifi S, Kazemipour N, Jelodar G, Saeb S. Impact of 900 MHz electromagnetic field exposure on main male reproductive hormone levels: a Rattus norvegicus model. Int J Biometeorol. 58(7):1657-1663, 2014a.

[Sepehrimanesh M](http://www.ncbi.nlm.nih.gov/pubmed?term=Sepehrimanesh%20M%5BAuthor%5D&cauthor=true&cauthor_uid=25146694), [Kazemipour N](http://www.ncbi.nlm.nih.gov/pubmed?term=Kazemipour%20N%5BAuthor%5D&cauthor=true&cauthor_uid=25146694), [Saeb M](http://www.ncbi.nlm.nih.gov/pubmed?term=Saeb%20M%5BAuthor%5D&cauthor=true&cauthor_uid=25146694), [Nazifi S](http://www.ncbi.nlm.nih.gov/pubmed?term=Nazifi%20S%5BAuthor%5D&cauthor=true&cauthor_uid=25146694). Analysis of rat testicular proteome following 30-days exposure to 900 MHz electromagnetic field radiation. [Electrophoresis.](http://www.ncbi.nlm.nih.gov/pubmed/25146694)35(23):3331-3338, 2014b.

[Sepehrimanesh M](https://www.ncbi.nlm.nih.gov/pubmed/?term=Sepehrimanesh%20M%5BAuthor%5D&cauthor=true&cauthor_uid=28397118), [Kazemipour N](https://www.ncbi.nlm.nih.gov/pubmed/?term=Kazemipour%20N%5BAuthor%5D&cauthor=true&cauthor_uid=28397118), [Saeb M](https://www.ncbi.nlm.nih.gov/pubmed/?term=Saeb%20M%5BAuthor%5D&cauthor=true&cauthor_uid=28397118), [Nazifi S](https://www.ncbi.nlm.nih.gov/pubmed/?term=Nazifi%20S%5BAuthor%5D&cauthor=true&cauthor_uid=28397118), [Davis DL](https://www.ncbi.nlm.nih.gov/pubmed/?term=Davis%20DL%5BAuthor%5D&cauthor=true&cauthor_uid=28397118). Proteomic analysis of continuous 900-MHz radiofrequency electromagnetic field exposure in testicular tissue: a rat model of human cell phone exposure. [Environ Sci Pollut Res Int.](https://www.ncbi.nlm.nih.gov/pubmed/28397118) 24(15):13666-13673, 2017.

[Shahin, S](http://www.ncbi.nlm.nih.gov/pubmed/?term=Shahin%252520S%25255BAuthor%25255D&cauthor=true&cauthor_uid=23334843)., [Singh, V.P](http://www.ncbi.nlm.nih.gov/pubmed/?term=Singh%252520VP%25255BAuthor%25255D&cauthor=true&cauthor_uid=23334843)., [Shukla, R.K](http://www.ncbi.nlm.nih.gov/pubmed/?term=Shukla%252520RK%25255BAuthor%25255D&cauthor=true&cauthor_uid=23334843)., [Dhawan, A](http://www.ncbi.nlm.nih.gov/pubmed/?term=Dhawan%252520A%25255BAuthor%25255D&cauthor=true&cauthor_uid=23334843)., [Gangwar, R.K](http://www.ncbi.nlm.nih.gov/pubmed/?term=Gangwar%252520RK%25255BAuthor%25255D&cauthor=true&cauthor_uid=23334843)., [Singh, S.P](http://www.ncbi.nlm.nih.gov/pubmed/?term=Singh%252520SP%25255BAuthor%25255D&cauthor=true&cauthor_uid=23334843)., [Chaturvedi, C.M](http://www.ncbi.nlm.nih.gov/pubmed/?term=Chaturvedi%252520CM%25255BAuthor%25255D&cauthor=true&cauthor_uid=23334843).. 2.45 GHz microwave irradiation-induced oxidative stress affects implantation or pregnancy in mice, Mus musculus. [Appl Biochem Biotechnol.](http://www.ncbi.nlm.nih.gov/pubmed/23334843) 169:1727-1751, 2013.

[Shahin S](http://www.ncbi.nlm.nih.gov/pubmed/?term=Shahin%20S%5BAuthor%5D&cauthor=true&cauthor_uid=24490664), [Mishra V](http://www.ncbi.nlm.nih.gov/pubmed/?term=Mishra%20V%5BAuthor%5D&cauthor=true&cauthor_uid=24490664), [Singh SP](http://www.ncbi.nlm.nih.gov/pubmed/?term=Singh%20SP%5BAuthor%5D&cauthor=true&cauthor_uid=24490664), [Chaturvedi CM](http://www.ncbi.nlm.nih.gov/pubmed/?term=Chaturvedi%20CM%5BAuthor%5D&cauthor=true&cauthor_uid=24490664). 2.45-GHz microwave irradiation adversely affects reproductive function in male mouse, Mus musculus by inducing oxidative and nitrosative stress. [Free Radic Res.](http://www.ncbi.nlm.nih.gov/pubmed/24490664) 48(5):511-525, 2014.

[Shahin](https://pubmed.ncbi.nlm.nih.gov/?sort=date&term=Shahin+S&cauthor_id=29968967) [S](https://pubmed.ncbi.nlm.nih.gov/29968967/#affiliation-1),  [Singh](https://pubmed.ncbi.nlm.nih.gov/?sort=date&term=Singh+SP&cauthor_id=29968967) [SP](https://pubmed.ncbi.nlm.nih.gov/29968967/#affiliation-2),  [Chaturvedi](https://pubmed.ncbi.nlm.nih.gov/?sort=date&term=Chaturvedi+CM&cauthor_id=29968967) [CM](https://pubmed.ncbi.nlm.nih.gov/29968967/#affiliation-1). 2.45 GHz microwave radiation induced oxidative and nitrosative stress mediated testicular apoptosis: Involvement of a p53 dependent bax-caspase-3 mediated pathway. Environ Toxicol 33(9):931-945, 2018.

[Sharma A](https://www.ncbi.nlm.nih.gov/pubmed/?term=Sharma%20A%5BAuthor%5D&cauthor=true&cauthor_uid=28470342), [Kesari KK](https://www.ncbi.nlm.nih.gov/pubmed/?term=Kesari%20KK%5BAuthor%5D&cauthor=true&cauthor_uid=28470342), [Saxena VK](https://www.ncbi.nlm.nih.gov/pubmed/?term=Saxena%20VK%5BAuthor%5D&cauthor=true&cauthor_uid=28470342), [Sisodia R](https://www.ncbi.nlm.nih.gov/pubmed/?term=Sisodia%20R%5BAuthor%5D&cauthor=true&cauthor_uid=28470342). Ten gigahertz microwave radiation impairs spatial memory, enzymes activity, and histopathology of developing mice brain. [Mol Cell Biochem.](https://www.ncbi.nlm.nih.gov/pubmed/28470342) 435(1-2):1-13, 2017.

[Sharma S](https://www.ncbi.nlm.nih.gov/pubmed/?term=Sharma%20S%5BAuthor%5D&cauthor=true&cauthor_uid=32205214), [Shukla S](https://www.ncbi.nlm.nih.gov/pubmed/?term=Shukla%20S%5BAuthor%5D&cauthor=true&cauthor_uid=32205214). Effect of electromagnetic radiation on redox status, acetylcholine esterase activity and cellular damage contributing to the diminution of the brain working memory in rats. [J Chem Neuroanat.](https://www.ncbi.nlm.nih.gov/pubmed/32205214) 106:101784, 2020.

[Sharma](https://pubmed.ncbi.nlm.nih.gov/?sort=date&term=Sharma+S&cauthor_id=39612822) [S](https://pubmed.ncbi.nlm.nih.gov/39612822/#full-view-affiliation-1),  [Sharma](https://pubmed.ncbi.nlm.nih.gov/?sort=date&term=Sharma+P&cauthor_id=39612822) [P](https://pubmed.ncbi.nlm.nih.gov/39612822/#full-view-affiliation-1),  [Singh](https://pubmed.ncbi.nlm.nih.gov/?sort=date&term=Singh+J&cauthor_id=39612822) [J](https://pubmed.ncbi.nlm.nih.gov/39612822/#full-view-affiliation-1),  [Bahel](https://pubmed.ncbi.nlm.nih.gov/?sort=date&term=Bahel+S&cauthor_id=39612822) [S](https://pubmed.ncbi.nlm.nih.gov/39612822/#full-view-affiliation-2),  [Dutta](https://pubmed.ncbi.nlm.nih.gov/?sort=date&term=Dutta+R&cauthor_id=39612822) [R](https://pubmed.ncbi.nlm.nih.gov/39612822/#full-view-affiliation-1),  [Vig](https://pubmed.ncbi.nlm.nih.gov/?sort=date&term=Vig+AP&cauthor_id=39612822) AP[1](https://pubmed.ncbi.nlm.nih.gov/39612822/#full-view-affiliation-1),  [Katnoria](https://pubmed.ncbi.nlm.nih.gov/?sort=date&term=Katnoria+JK&cauthor_id=39612822) [JK.](https://pubmed.ncbi.nlm.nih.gov/39612822/#full-view-affiliation-3) Assessing cell viability and genotoxicity in Trigonella foenum-graecum L. exposed to 2100 MHz and 2300 MHz electromagnetic field radiations. Plant Physiol Biochem 219:109311, 2024.

[Singh](https://pubmed.ncbi.nlm.nih.gov/?sort=date&term=Singh+KV&cauthor_id=34984797), [K.V](https://pubmed.ncbi.nlm.nih.gov/34984797/#affiliation-1)., [Arya](https://pubmed.ncbi.nlm.nih.gov/?sort=date&term=Arya+R&cauthor_id=34984797), R.,  [Nirala](https://pubmed.ncbi.nlm.nih.gov/?sort=date&term=Nirala+JP&cauthor_id=34984797), [J..P](https://pubmed.ncbi.nlm.nih.gov/34984797/#affiliation-1),  [Sahu](https://pubmed.ncbi.nlm.nih.gov/?sort=date&term=Sahu+D&cauthor_id=34984797), [D](https://pubmed.ncbi.nlm.nih.gov/34984797/#affiliation-4).,  [Nanda](https://pubmed.ncbi.nlm.nih.gov/?sort=date&term=Nanda+RK&cauthor_id=34984797), [R](https://pubmed.ncbi.nlm.nih.gov/34984797/#affiliation-2).K., [Rajamani](https://pubmed.ncbi.nlm.nih.gov/?sort=date&term=Rajamani+P&cauthor_id=34984797), [P.](https://pubmed.ncbi.nlm.nih.gov/34984797/#affiliation-1) Effects of mobile phone electromagnetic radiation on rat hippocampus proteome. Environ. Toxicol. 37(4):836-841, 2022.

[Sirav, B](http://www.ncbi.nlm.nih.gov/pubmed?term=Sirav%20B%5BAuthor%5D&cauthor=true&cauthor_uid=22047463)., [Seyhan, N](http://www.ncbi.nlm.nih.gov/pubmed?term=Seyhan%20N%5BAuthor%5D&cauthor=true&cauthor_uid=22047463). Effects of radiofrequency radiation exposure on blood-brain barrier permeability in male and female rats. [Electromagn Biol Med.](http://www.ncbi.nlm.nih.gov/pubmed/22047463) 30:253-260, 2011.

[Sırav, B](https://www.ncbi.nlm.nih.gov/pubmed/?term=S%C4%B1rav%20B%5BAuthor%5D&cauthor=true&cauthor_uid=26723545)., [Seyhan, N](https://www.ncbi.nlm.nih.gov/pubmed/?term=Seyhan%20N%5BAuthor%5D&cauthor=true&cauthor_uid=26723545). Effects of GSM modulated radio-frequency electromagnetic radiation on permeability of blood-brain barrier in male & female rats. [J Chem Neuroanat.](https://www.ncbi.nlm.nih.gov/pubmed/26723545) 75(Pt B):123-127, 2016.

[Sokolovic D](http://www.ncbi.nlm.nih.gov/sites/entrez?Db=pubmed&Cmd=Search&Term=%22Sokolovic%20D%22%5BAuthor%5D&itool=EntrezSystem2.PEntrez.Pubmed.Pubmed_ResultsPanel.Pubmed_DiscoveryPanel.Pubmed_RVAbstractPlus), [Djindjic B](http://www.ncbi.nlm.nih.gov/sites/entrez?Db=pubmed&Cmd=Search&Term=%22Djindjic%20B%22%5BAuthor%5D&itool=EntrezSystem2.PEntrez.Pubmed.Pubmed_ResultsPanel.Pubmed_DiscoveryPanel.Pubmed_RVAbstractPlus), [Nikolic J](http://www.ncbi.nlm.nih.gov/sites/entrez?Db=pubmed&Cmd=Search&Term=%22Nikolic%20J%22%5BAuthor%5D&itool=EntrezSystem2.PEntrez.Pubmed.Pubmed_ResultsPanel.Pubmed_DiscoveryPanel.Pubmed_RVAbstractPlus), [Bjelakovic G](http://www.ncbi.nlm.nih.gov/sites/entrez?Db=pubmed&Cmd=Search&Term=%22Bjelakovic%20G%22%5BAuthor%5D&itool=EntrezSystem2.PEntrez.Pubmed.Pubmed_ResultsPanel.Pubmed_DiscoveryPanel.Pubmed_RVAbstractPlus), [Pavlovic D](http://www.ncbi.nlm.nih.gov/sites/entrez?Db=pubmed&Cmd=Search&Term=%22Pavlovic%20D%22%5BAuthor%5D&itool=EntrezSystem2.PEntrez.Pubmed.Pubmed_ResultsPanel.Pubmed_DiscoveryPanel.Pubmed_RVAbstractPlus), [Kocic G](http://www.ncbi.nlm.nih.gov/sites/entrez?Db=pubmed&Cmd=Search&Term=%22Kocic%20G%22%5BAuthor%5D&itool=EntrezSystem2.PEntrez.Pubmed.Pubmed_ResultsPanel.Pubmed_DiscoveryPanel.Pubmed_RVAbstractPlus), [Krstic D](http://www.ncbi.nlm.nih.gov/sites/entrez?Db=pubmed&Cmd=Search&Term=%22Krstic%20D%22%5BAuthor%5D&itool=EntrezSystem2.PEntrez.Pubmed.Pubmed_ResultsPanel.Pubmed_DiscoveryPanel.Pubmed_RVAbstractPlus), [Cvetkovic T](http://www.ncbi.nlm.nih.gov/sites/entrez?Db=pubmed&Cmd=Search&Term=%22Cvetkovic%20T%22%5BAuthor%5D&itool=EntrezSystem2.PEntrez.Pubmed.Pubmed_ResultsPanel.Pubmed_DiscoveryPanel.Pubmed_RVAbstractPlus), [Pavlovic V](http://www.ncbi.nlm.nih.gov/sites/entrez?Db=pubmed&Cmd=Search&Term=%22Pavlovic%20V%22%5BAuthor%5D&itool=EntrezSystem2.PEntrez.Pubmed.Pubmed_ResultsPanel.Pubmed_DiscoveryPanel.Pubmed_RVAbstractPlus). Melatonin reduces oxidative stress induced by chronic exposure of microwave radiation from mobile phones in rat brain. [J Radiat Res (Tokyo).](javascript:AL_get(this,%20'jour',%20'J%20Radiat%20Res%20(Tokyo).');) 49(6):579-586, 2008.

Sokolovic D, Djordjevic B, Kocic G, Veljkovic A, Marinkovic M, Basic J, Jevtovic-Stoimenov T, Stanojkovic Z, Sokolovic DM, Pavlovic V, Djindjic B, Krstic D. Melatonin protects rat thymus against oxidative stress caused by exposure to microwaves and modulates proliferation/apoptosis of thymocytes. Gen Physiol Biophys 32(1):79-90, 2013.

Sokolovic D, Djordjevic B, Kocic G, Stoimenov TJ, Stanojkovic Z, Sokolovic DM, et al. The Effects of Melatonin on Oxidative Stress Parameters and DNA Fragmentation in Testicular Tissue of Rats Exposed to Microwave Radiation. Adv Clin Exp Med. 24(3):429-436, 2015.

Somosy, Z., Thuroczy, G., Kubasova, T., Kovacs, J., Szabo, L.D. Effects of modulated and continuous microwave irradiation on the morphology and cell surface negative charge of 3T3 fibroblasts. Scanning Microsc. 5:1145-1155, 1991.

Spandole-Dinu S, Catrina A-M, Voinea OC, Andone A, Radu S , Haidoiu C, Călborean O , Popescu DM , Suhăianu V, Baltag O , Tută L, Rosu G. Pilot study of the long-term effects of radiofrequency electromagnetic radiation exposure on the mouse brain. Int. J. Environ. Res. Public Health 20(4):3025, 2023.

Stagg, R.B., Thomas, W.J., Jones, R.A., Adey, W.R. DNA synthesis and cell proliferation in C6 glioma and primary glial cells exposed to a 836.55 MHz modulated radiofrequency field. Bioelectromagnetics. 18:230-236, 1997.

Stankiewicz, W., Dąbrowski, M.P., Kubacki, R., Sobiczewska, E., Szmigielski, S. Immunotropic lnfluence of 900 MHz microwave GSM signal on human blood immune cells activated in vitro. Electromagn Biol Med. 25: 45-51, 2006.

[Stasinopoulou M](https://www.ncbi.nlm.nih.gov/pubmed/?term=Stasinopoulou%20M%5BAuthor%5D&cauthor=true&cauthor_uid=27544572), [Fragopoulou AF](https://www.ncbi.nlm.nih.gov/pubmed/?term=Fragopoulou%20AF%5BAuthor%5D&cauthor=true&cauthor_uid=27544572), [Stamatakis A](https://www.ncbi.nlm.nih.gov/pubmed/?term=Stamatakis%20A%5BAuthor%5D&cauthor=true&cauthor_uid=27544572), [Mantziaras G](https://www.ncbi.nlm.nih.gov/pubmed/?term=Mantziaras%20G%5BAuthor%5D&cauthor=true&cauthor_uid=27544572), [Skouroliakou K](https://www.ncbi.nlm.nih.gov/pubmed/?term=Skouroliakou%20K%5BAuthor%5D&cauthor=true&cauthor_uid=27544572), [Papassideri IS](https://www.ncbi.nlm.nih.gov/pubmed/?term=Papassideri%20IS%5BAuthor%5D&cauthor=true&cauthor_uid=27544572), [Stylianopoulou F](https://www.ncbi.nlm.nih.gov/pubmed/?term=Stylianopoulou%20F%5BAuthor%5D&cauthor=true&cauthor_uid=27544572), [Lai H](https://www.ncbi.nlm.nih.gov/pubmed/?term=Lai%20H%5BAuthor%5D&cauthor=true&cauthor_uid=27544572), [Kostomitsopoulos N](https://www.ncbi.nlm.nih.gov/pubmed/?term=Kostomitsopoulos%20N%5BAuthor%5D&cauthor=true&cauthor_uid=27544572), [Margaritis LH](https://www.ncbi.nlm.nih.gov/pubmed/?term=Margaritis%20LH%5BAuthor%5D&cauthor=true&cauthor_uid=27544572). Effects of pre- and postnatal exposure to 1880-1900MHz DECT base radiation on development in the rat. [Reprod Toxicol.](https://www.ncbi.nlm.nih.gov/pubmed/27544572) 65:248-262, 2016.

[Suhhova](https://pubmed.ncbi.nlm.nih.gov/?sort=pubdate&term=Suhhova+A&cauthor_id=23280729), [A](https://pubmed.ncbi.nlm.nih.gov/23280729/#affiliation-1).,  [Bachmann](https://pubmed.ncbi.nlm.nih.gov/?sort=pubdate&term=Bachmann+M&cauthor_id=23280729), M., [Karai](https://pubmed.ncbi.nlm.nih.gov/?sort=pubdate&term=Karai+D&cauthor_id=23280729), D., [Lass](https://pubmed.ncbi.nlm.nih.gov/?sort=pubdate&term=Lass+J&cauthor_id=23280729), J., [Hinrikus](https://pubmed.ncbi.nlm.nih.gov/?sort=pubdate&term=Hinrikus+H&cauthor_id=23280729), H. Effect of microwave radiation on human EEG at two different levels of exposure. Bioelectromagnetics. 34:264-74, 2013.

Sun, Y., Zong, L., Gao, Z., Zhu, S., Tong, J., Cao, Y. Mitochondrial DNA damage and oxidative damage in HL-60 cells exposed to 900 MHz radiofrequency fields. Mutat Res. 797:7-14, 2017.

[Szymański, Ł](https://www.ncbi.nlm.nih.gov/pubmed/?term=Szyma%2525C5%252584ski%252520%2525C5%252581%25255BAuthor%25255D&cauthor=true&cauthor_uid=31832654)., [Sobiczewska, E](https://www.ncbi.nlm.nih.gov/pubmed/?term=Sobiczewska%252520E%25255BAuthor%25255D&cauthor=true&cauthor_uid=31832654)., [Cios, A](https://www.ncbi.nlm.nih.gov/pubmed/?term=Cios%252520A%25255BAuthor%25255D&cauthor=true&cauthor_uid=31832654)., [Szymanski, P](https://www.ncbi.nlm.nih.gov/pubmed/?term=Szymanski%252520P%25255BAuthor%25255D&cauthor=true&cauthor_uid=31832654)., [Ciepielak, M](https://www.ncbi.nlm.nih.gov/pubmed/?term=Ciepielak%252520M%25255BAuthor%25255D&cauthor=true&cauthor_uid=31832654)., [Stankiewicz, W](https://www.ncbi.nlm.nih.gov/pubmed/?term=Stankiewicz%252520W%25255BAuthor%25255D&cauthor=true&cauthor_uid=31832654). Immunotropic effects in cultured human blood mononuclear cells exposed to a 900 MHz pulse-modulated microwave field. [J Radiat Res.](https://www.ncbi.nlm.nih.gov/pubmed/31832654) 61:27-33, 2020.

[Tahir](https://pubmed.ncbi.nlm.nih.gov/?sort=date&term=Tahir+E&cauthor_id=38454287) [E](https://pubmed.ncbi.nlm.nih.gov/38454287/#full-view-affiliation-1),  [Karadayı](https://pubmed.ncbi.nlm.nih.gov/?sort=date&term=Akar+Karaday%C4%B1+A&cauthor_id=38454287) [AA](https://pubmed.ncbi.nlm.nih.gov/38454287/#full-view-affiliation-2),  [Gürgen](https://pubmed.ncbi.nlm.nih.gov/?sort=date&term=G%C3%BCl%C5%9Fen+G%C3%BCrgen+S&cauthor_id=38454287) [SG](https://pubmed.ncbi.nlm.nih.gov/38454287/#full-view-affiliation-3),  [Engiz](https://pubmed.ncbi.nlm.nih.gov/?sort=date&term=Korunur+Engiz+B&cauthor_id=38454287) [BK](https://pubmed.ncbi.nlm.nih.gov/38454287/#full-view-affiliation-3), [Turgut](https://pubmed.ncbi.nlm.nih.gov/?sort=date&term=Turgut+A&cauthor_id=38454287) [A.](https://pubmed.ncbi.nlm.nih.gov/38454287/#full-view-affiliation-4) Effect of 2.45 GHz microwave radiation on the inner ear: a histopathological study on 2.45 GHz microwave radiation and cochlea. J Int Adv Otol 20(1):35-43, 2024.

[Tan](https://pubmed.ncbi.nlm.nih.gov/?sort=date&term=Cant%C3%BCrk+Tan+F&cauthor_id=35635232) [FC](https://pubmed.ncbi.nlm.nih.gov/35635232/#full-view-affiliation-1),  [Yalçin](https://pubmed.ncbi.nlm.nih.gov/?sort=date&term=Yal%C3%A7in+B&cauthor_id=35635232) [B](https://pubmed.ncbi.nlm.nih.gov/35635232/#full-view-affiliation-2),  [Yay](https://pubmed.ncbi.nlm.nih.gov/?sort=date&term=Yay+AH&cauthor_id=35635232) [AH](https://pubmed.ncbi.nlm.nih.gov/35635232/#full-view-affiliation-2),  [Tan](https://pubmed.ncbi.nlm.nih.gov/?sort=date&term=Tan+B&cauthor_id=35635232) [B](https://pubmed.ncbi.nlm.nih.gov/35635232/#full-view-affiliation-3),  [Yeğin](https://pubmed.ncbi.nlm.nih.gov/?sort=date&term=Ye%C4%9Fin+K&cauthor_id=35635232) [K](https://pubmed.ncbi.nlm.nih.gov/35635232/#full-view-affiliation-4),  [Daşdağ](https://pubmed.ncbi.nlm.nih.gov/?sort=date&term=Da%C5%9Fda%C4%9F+S&cauthor_id=35635232) [S.](https://pubmed.ncbi.nlm.nih.gov/35635232/#full-view-affiliation-5) Effects of pre and postnatal 2450 MHz continuous wave (CW) radiofrequency radiation on thymus: Four generation exposure. Electromagn Biol Med 41(3):315-324, 2022.

[Tang, J](http://www.ncbi.nlm.nih.gov/pubmed/?term=Tang%20J%5BAuthor%5D&cauthor=true&cauthor_uid=25598203)., [Zhang, Y](http://www.ncbi.nlm.nih.gov/pubmed/?term=Zhang%20Y%5BAuthor%5D&cauthor=true&cauthor_uid=25598203)., [Yang, L](http://www.ncbi.nlm.nih.gov/pubmed/?term=Yang%20L%5BAuthor%5D&cauthor=true&cauthor_uid=25598203)., [Chen, Q](http://www.ncbi.nlm.nih.gov/pubmed/?term=Chen%20Q%5BAuthor%5D&cauthor=true&cauthor_uid=25598203)., [Tan, L](http://www.ncbi.nlm.nih.gov/pubmed/?term=Tan%20L%5BAuthor%5D&cauthor=true&cauthor_uid=25598203)., [Zuo, S](http://www.ncbi.nlm.nih.gov/pubmed/?term=Zuo%20S%5BAuthor%5D&cauthor=true&cauthor_uid=25598203)., [Feng, H](http://www.ncbi.nlm.nih.gov/pubmed/?term=Feng%20H%5BAuthor%5D&cauthor=true&cauthor_uid=25598203)., [Chen, Z](http://www.ncbi.nlm.nih.gov/pubmed/?term=Chen%20Z%5BAuthor%5D&cauthor=true&cauthor_uid=25598203)., [Zhu, G](http://www.ncbi.nlm.nih.gov/pubmed/?term=Zhu%20G%5BAuthor%5D&cauthor=true&cauthor_uid=25598203). Exposure to 900 MHz electromagnetic fields activates the mkp-1/ERK pathway and causes blood-brain barrier damage and cognitive impairment in rats. [Brain Res.](http://www.ncbi.nlm.nih.gov/pubmed/25598203) 1601:92-101, 2015.

[Tas M](http://www.ncbi.nlm.nih.gov/pubmed?term=Tas%20M%5BAuthor%5D&cauthor=true&cauthor_uid=23781998), [Dasdag S](http://www.ncbi.nlm.nih.gov/pubmed?term=Dasdag%20S%5BAuthor%5D&cauthor=true&cauthor_uid=23781998), [Akdag MZ](http://www.ncbi.nlm.nih.gov/pubmed?term=Akdag%20MZ%5BAuthor%5D&cauthor=true&cauthor_uid=23781998), [Cirit U](http://www.ncbi.nlm.nih.gov/pubmed?term=Cirit%20U%5BAuthor%5D&cauthor=true&cauthor_uid=23781998), [Yegin K](http://www.ncbi.nlm.nih.gov/pubmed?term=Yegin%20K%5BAuthor%5D&cauthor=true&cauthor_uid=23781998), [Seker U](http://www.ncbi.nlm.nih.gov/pubmed?term=Seker%20U%5BAuthor%5D&cauthor=true&cauthor_uid=23781998), [Ozmen MF](http://www.ncbi.nlm.nih.gov/pubmed?term=Ozmen%20MF%5BAuthor%5D&cauthor=true&cauthor_uid=23781998), [Eren LB](http://www.ncbi.nlm.nih.gov/pubmed?term=Eren%20LB%5BAuthor%5D&cauthor=true&cauthor_uid=23781998). Long-term effects of 900 MHz radiofrequency radiation emitted from mobile phone on testicular tissue and epididymal semen quality. [Electromagn Biol Med.](http://www.ncbi.nlm.nih.gov/pubmed/23781998) 33(3):216-222, 2014.

Tattersall, J.E., Scott, I.R., Wood, S.J., Nettell. J.J., Bevir, M.K., Wang,. Z, Somasiri, N.P., Chen, X. Effects of low intensity radiofrequency electromagnetic fields on electrical activity in rat hippocampal slices. Brain Res. 904: 43-53, 2001.

[Tkalec, M](http://www.ncbi.nlm.nih.gov/pubmed?term=Tkalec%20M%5BAuthor%5D&cauthor=true&cauthor_uid=23352129)., [Stambuk, A](http://www.ncbi.nlm.nih.gov/pubmed?term=Stambuk%20A%5BAuthor%5D&cauthor=true&cauthor_uid=23352129)., [Srut, M](http://www.ncbi.nlm.nih.gov/pubmed?term=Srut%20M%5BAuthor%5D&cauthor=true&cauthor_uid=23352129)., [Malarić, K](http://www.ncbi.nlm.nih.gov/pubmed?term=Malari%C4%87%20K%5BAuthor%5D&cauthor=true&cauthor_uid=23352129)., [Klobučar, G.I](http://www.ncbi.nlm.nih.gov/pubmed?term=Klobu%C4%8Dar%20GI%5BAuthor%5D&cauthor=true&cauthor_uid=23352129). Oxidative and genotoxic effects of 900 MHz electromagnetic fields in the earthworm Eisenia fetida. [Ecotoxicol Environ Saf.](http://www.ncbi.nlm.nih.gov/pubmed/23352129) 90:7-12, 2013.

[Tomruk](https://pubmed.ncbi.nlm.nih.gov/?sort=date&term=Tomruk+A&cauthor_id=35904122) A,  [Ozgur-Buyukatalay](https://pubmed.ncbi.nlm.nih.gov/?sort=date&term=Ozgur-Buyukatalay+E&cauthor_id=35904122) [E](https://pubmed.ncbi.nlm.nih.gov/35904122/#affiliation-1),  [Ozturk](https://pubmed.ncbi.nlm.nih.gov/?sort=date&term=Ozturk+GG&cauthor_id=35904122) [GG](https://pubmed.ncbi.nlm.nih.gov/35904122/#affiliation-1),  [Ulusu](https://pubmed.ncbi.nlm.nih.gov/?sort=date&term=Ulusu+NN&cauthor_id=35904122) [NN.](https://pubmed.ncbi.nlm.nih.gov/35904122/#affiliation-2) Short-term exposure to radiofrequency radiation and metabolic enzymes' activities during pregnancy and prenatal development. Electromagn Biol Med 41(4):370-378, 2022.

[Topal Z](http://www.ncbi.nlm.nih.gov/pubmed/?term=Topal%20Z%5BAuthor%5D&cauthor=true&cauthor_uid=26084117), [Hanci H](http://www.ncbi.nlm.nih.gov/pubmed/?term=Hanci%20H%5BAuthor%5D&cauthor=true&cauthor_uid=26084117), [Mercantepe T](http://www.ncbi.nlm.nih.gov/pubmed/?term=Mercantepe%20T%5BAuthor%5D&cauthor=true&cauthor_uid=26084117), [Erol HS](http://www.ncbi.nlm.nih.gov/pubmed/?term=Erol%20HS%5BAuthor%5D&cauthor=true&cauthor_uid=26084117), [Keleş ON](http://www.ncbi.nlm.nih.gov/pubmed/?term=Kele%C5%9F%20ON%5BAuthor%5D&cauthor=true&cauthor_uid=26084117), [Kaya H](http://www.ncbi.nlm.nih.gov/pubmed/?term=Kaya%20H%5BAuthor%5D&cauthor=true&cauthor_uid=26084117), [Mungan S](http://www.ncbi.nlm.nih.gov/pubmed/?term=Mungan%20S%5BAuthor%5D&cauthor=true&cauthor_uid=26084117), [Odaci E](http://www.ncbi.nlm.nih.gov/pubmed/?term=Odaci%20E%5BAuthor%5D&cauthor=true&cauthor_uid=26084117). The effects of prenatal long-duration exposure to 900-MHz electromagnetic field on the 21-day-old newborn male rat liver. [Turk J Med Sci.](http://www.ncbi.nlm.nih.gov/pubmed/26084117) 45(2):291-297, 2015.

[Tripathi](https://pubmed.ncbi.nlm.nih.gov/?sort=date&term=Tripathi+R&cauthor_id=38098324) [R](https://pubmed.ncbi.nlm.nih.gov/38098324/#full-view-affiliation-1),  [Banerjee](https://pubmed.ncbi.nlm.nih.gov/?sort=date&term=Banerjee+SK&cauthor_id=38098324) [SK](https://pubmed.ncbi.nlm.nih.gov/38098324/#full-view-affiliation-2),  [Nirala](https://pubmed.ncbi.nlm.nih.gov/?sort=date&term=Nirala+JP&cauthor_id=38098324) [JP](https://pubmed.ncbi.nlm.nih.gov/38098324/#full-view-affiliation-3),  [Mathur](https://pubmed.ncbi.nlm.nih.gov/?sort=date&term=Mathur+R&cauthor_id=38098324) R. Exposure to electromagnetic fields from mobile phones and fructose consumption coalesce to perturb metabolic regulators AMPK/SIRT1-UCP2/FOXO1 in growing rats. Biomed Environ Sci 36(11):1045-1058, 2023.

[Trosić](https://pubmed.ncbi.nlm.nih.gov/?sort=date&term=Trosi%C4%87+I&cauthor_id=19329382), I. , [Pavicić](https://pubmed.ncbi.nlm.nih.gov/?sort=date&term=Pavici%C4%87+I&cauthor_id=19329382), I. Disturbance of cell proliferation in response to mobile phone frequency radiation. Arh Hig Rada Toksikol. 60:109-115, 2009.

[Tsoy, A](https://www.ncbi.nlm.nih.gov/pubmed/?term=Tsoy%20A%5BAuthor%5D&cauthor=true&cauthor_uid=30953670)., [Saliev, T](https://www.ncbi.nlm.nih.gov/pubmed/?term=Saliev%20T%5BAuthor%5D&cauthor=true&cauthor_uid=30953670)., [Abzhanova, E](https://www.ncbi.nlm.nih.gov/pubmed/?term=Abzhanova%20E%5BAuthor%5D&cauthor=true&cauthor_uid=30953670)., [Turgambayeva, A](https://www.ncbi.nlm.nih.gov/pubmed/?term=Turgambayeva%20A%5BAuthor%5D&cauthor=true&cauthor_uid=30953670)., [Kaiyrlykyzy, A](https://www.ncbi.nlm.nih.gov/pubmed/?term=Kaiyrlykyzy%20A%5BAuthor%5D&cauthor=true&cauthor_uid=30953670)., [Akishev, M](https://www.ncbi.nlm.nih.gov/pubmed/?term=Akishev%20M%5BAuthor%5D&cauthor=true&cauthor_uid=30953670)., [Saparbayev, S](https://www.ncbi.nlm.nih.gov/pubmed/?term=Saparbayev%20S%5BAuthor%5D&cauthor=true&cauthor_uid=30953670)., [Umbayev, B](https://www.ncbi.nlm.nih.gov/pubmed/?term=Umbayev%20B%5BAuthor%5D&cauthor=true&cauthor_uid=30953670)., [Askarova, S](https://www.ncbi.nlm.nih.gov/pubmed/?term=Askarova%20S%5BAuthor%5D&cauthor=true&cauthor_uid=30953670). The effects of mobile phone radiofrequency electromagnetic fields on β-amyloid-Induced oxidative stress in human and rat primary astrocytes. [Neurosci.](https://www.ncbi.nlm.nih.gov/pubmed/30953670)408:46-57, 2019.

[Tsybulin, O](https://www.ncbi.nlm.nih.gov/pubmed/?term=Tsybulin%252520O%25255BAuthor%25255D&cauthor=true&cauthor_uid=23578013)., [Sidorik, E](https://www.ncbi.nlm.nih.gov/pubmed/?term=Sidorik%252520E%25255BAuthor%25255D&cauthor=true&cauthor_uid=23578013)., [Brieieva, O](https://www.ncbi.nlm.nih.gov/pubmed/?term=Brieieva%252520O%25255BAuthor%25255D&cauthor=true&cauthor_uid=23578013)., [Buchynska, L](https://www.ncbi.nlm.nih.gov/pubmed/?term=Buchynska%252520L%25255BAuthor%25255D&cauthor=true&cauthor_uid=23578013)., [Kyrylenko, S](https://www.ncbi.nlm.nih.gov/pubmed/?term=Kyrylenko%252520S%25255BAuthor%25255D&cauthor=true&cauthor_uid=23578013)., [Henshel, D](https://www.ncbi.nlm.nih.gov/pubmed/?term=Henshel%252520D%25255BAuthor%25255D&cauthor=true&cauthor_uid=23578013)., [Yakymenko, I](https://www.ncbi.nlm.nih.gov/pubmed/?term=Yakymenko%252520I%25255BAuthor%25255D&cauthor=true&cauthor_uid=23578013). GSM 900 MHz cellular phone radiation can either stimulate or depress early embryogenesis in Japanese quails depending on the duration of exposure. [Int J Radiat Biol.](https://www.ncbi.nlm.nih.gov/pubmed/23578013) 89:756-763, 2013.

[Türedi](https://pubmed.ncbi.nlm.nih.gov/?sort=date&term=T%C3%BCredi+S&cauthor_id=25166431) [S](https://pubmed.ncbi.nlm.nih.gov/25166431/#affiliation-1),  [Hancı](https://pubmed.ncbi.nlm.nih.gov/?sort=date&term=Hanc%C4%B1+H&cauthor_id=25166431) [H](https://pubmed.ncbi.nlm.nih.gov/25166431/#affiliation-1),  [Topal](https://pubmed.ncbi.nlm.nih.gov/?sort=date&term=Topal+Z&cauthor_id=25166431) [Z](https://pubmed.ncbi.nlm.nih.gov/25166431/#affiliation-1),  [Ünal](https://pubmed.ncbi.nlm.nih.gov/?sort=date&term=%C3%9Cnal+D&cauthor_id=25166431) [D](https://pubmed.ncbi.nlm.nih.gov/25166431/#affiliation-2),  [Mercantepe](https://pubmed.ncbi.nlm.nih.gov/?sort=date&term=Mercantepe+T&cauthor_id=25166431) [T](https://pubmed.ncbi.nlm.nih.gov/25166431/#affiliation-3),  [Bozkurt](https://pubmed.ncbi.nlm.nih.gov/?sort=date&term=Bozkurt+%C4%B0&cauthor_id=25166431) [I](https://pubmed.ncbi.nlm.nih.gov/25166431/#affiliation-4),  [Kaya](https://pubmed.ncbi.nlm.nih.gov/?sort=date&term=Kaya+H&cauthor_id=25166431) [H](https://pubmed.ncbi.nlm.nih.gov/25166431/#affiliation-5),  [Odacı](https://pubmed.ncbi.nlm.nih.gov/?sort=date&term=Odac%C4%B1+E&cauthor_id=25166431) [E.](https://pubmed.ncbi.nlm.nih.gov/25166431/#affiliation-1) The effects of prenatal exposure to a 900-MHz electromagnetic field on the 21-day-old male rat heart. Electromagn Biol Med 34(4):390-397, 2015.

Türedi S, Hancı H, Çolakoğlu S, Kaya H, Odacı E. Disruption of the ovarian follicle reservoir of prepubertal rats following prenatal exposure to a continuous 900-MHz electromagnetic field. Int J Radiat Biol. 92(6):329-337, 2016.

[Türedi S](https://www.ncbi.nlm.nih.gov/pubmed/?term=T%C3%BCredi%20S%5BAuthor%5D&cauthor=true&cauthor_uid=28747141), [Kerimoğlu G](https://www.ncbi.nlm.nih.gov/pubmed/?term=Kerimo%C4%9Flu%20G%5BAuthor%5D&cauthor=true&cauthor_uid=28747141), [Mercantepe T](https://www.ncbi.nlm.nih.gov/pubmed/?term=Mercantepe%20T%5BAuthor%5D&cauthor=true&cauthor_uid=28747141), [Odacı E](https://www.ncbi.nlm.nih.gov/pubmed/?term=Odac%C4%B1%20E%5BAuthor%5D&cauthor=true&cauthor_uid=28747141). Biochemical and pathological changes in the male rat kidney and bladder following exposure to continuous 900-MHz electromagnetic field on postnatal days 22-59. [Int J Radiat Biol.](https://www.ncbi.nlm.nih.gov/pubmed/28747141) 93(9):990-999, 2017.

Upadhyaya, C., Upadhyaya, T., Patel, I. Attributes of non-ionizing radiation of 1800 MHz frequency on plant health and antioxidant content of tomato (Solanum lycopersicum) plants. J Rad Res Appl Sci. 15:54-68, 2022.

Vafaei  [H](https://pubmed.ncbi.nlm.nih.gov/32695301/#affiliation-1),  [Kavari](https://pubmed.ncbi.nlm.nih.gov/?term=Kavari+G&cauthor_id=32695301)  G, [Izadi](https://pubmed.ncbi.nlm.nih.gov/?term=Izadi+HR&cauthor_id=32695301) [HR](https://pubmed.ncbi.nlm.nih.gov/32695301/#affiliation-2),  [Dorahi](https://pubmed.ncbi.nlm.nih.gov/?term=Zare+Dorahi+Z&cauthor_id=32695301)  ZZ,  [Dianatpour](https://pubmed.ncbi.nlm.nih.gov/?term=Dianatpour+M&cauthor_id=32695301)  M, [Daneshparvar](https://pubmed.ncbi.nlm.nih.gov/?term=Daneshparvar+A&cauthor_id=32695301)  A, [Jamhiri](https://pubmed.ncbi.nlm.nih.gov/?term=Jamhiri+I&cauthor_id=32695301)  I. Wi-Fi (2.4 GHz) affects anti-oxidant capacity, DNA repair genes expression and, apoptosis in pregnant mouse placenta. Iran J Basic Med Sci 23(6):833-840, 2020.

Velizarov, S., Raskmark, P., Kwee, S. The effects of radiofrequency fields on cell proliferation are non-thermal. Bioelectrochem Bioenerg. 48:177-180, 1999.

Veyret, B., Bouthet, C., Deschaux, P., de Seze, R., Geffard, M., Joussot-Dubien, J, Le Diraison, M., Moreau, J.M., Caristan A. Antibody responses of mice exposed to low-power microwaves under combined, pulse-and-amplitude modulation. Bioelectromagnetics. 12:47-56, 1991.

Wang J, Dong J, Xu Q, Yan S, Wang H, Lei H, Ma X, Yang T, Wang K, Li Z, Wang X. Melatonin ameliorates RF-EMR-induced reproductive damage by inhibiting ferroptosis through Nrf2 pathway activation. Pathol Res Pract. 270:156003, 2025.

[Wang XW](http://www.ncbi.nlm.nih.gov/pubmed?term=%22Wang%20XW%22%5BAuthor%5D), [Ding GR](http://www.ncbi.nlm.nih.gov/pubmed?term=%22Ding%20GR%22%5BAuthor%5D), [Shi CH](http://www.ncbi.nlm.nih.gov/pubmed?term=%22Shi%20CH%22%5BAuthor%5D), [Zeng LH](http://www.ncbi.nlm.nih.gov/pubmed?term=%22Zeng%20LH%22%5BAuthor%5D), [Liu JY](http://www.ncbi.nlm.nih.gov/pubmed?term=%22Liu%20JY%22%5BAuthor%5D), [Li J](http://www.ncbi.nlm.nih.gov/pubmed?term=%22Li%20J%22%5BAuthor%5D), [Zhao T](http://www.ncbi.nlm.nih.gov/pubmed?term=%22Zhao%20T%22%5BAuthor%5D), [Chen YB](http://www.ncbi.nlm.nih.gov/pubmed?term=%22Chen%20YB%22%5BAuthor%5D), [Guo GZ](http://www.ncbi.nlm.nih.gov/pubmed?term=%22Guo%20GZ%22%5BAuthor%5D).

Mechanisms involved in the blood-testis barrier increased permeability induced by EMP. [Toxicology.](http://www.ncbi.nlm.nih.gov/pubmed/20633596##) 276(1):58-63, 2010.

[Wang](https://pubmed.ncbi.nlm.nih.gov/?sort=date&term=Wang+Y&cauthor_id=34896224), [Y](https://pubmed.ncbi.nlm.nih.gov/34896224/#affiliation-1).,  [Jiang](https://pubmed.ncbi.nlm.nih.gov/?sort=date&term=Jiang+Z&cauthor_id=34896224), [Z](https://pubmed.ncbi.nlm.nih.gov/34896224/#affiliation-1)., [Zhang](https://pubmed.ncbi.nlm.nih.gov/?sort=date&term=Zhang+L&cauthor_id=34896224), [L](https://pubmed.ncbi.nlm.nih.gov/34896224/#affiliation-1).,  [Zhang](https://pubmed.ncbi.nlm.nih.gov/?sort=date&term=Zhang+Z&cauthor_id=34896224), [Z](https://pubmed.ncbi.nlm.nih.gov/34896224/#affiliation-2).,  [Liao](https://pubmed.ncbi.nlm.nih.gov/?sort=date&term=Liao+Y&cauthor_id=34896224), [Y](https://pubmed.ncbi.nlm.nih.gov/34896224/#affiliation-1)., [Cai](https://pubmed.ncbi.nlm.nih.gov/?sort=date&term=Cai+P&cauthor_id=34896224), [P](https://pubmed.ncbi.nlm.nih.gov/34896224/#affiliation-3). 3.5-GHz radiofrequency electromagnetic radiation promotes the development of Drosophila melanogaster. Environ Pollut. 294:118646, 2022.

Wang Y, Zhang Z, Zhang L, Liao Y, Cai P. 3.5GHz radiofrequency electromagnetic fields (RF-EMF) on metabolic disorders in Drosophila melanogaster. Ecotoxicol Environ Saf. 304:119132, 2025.

Wen X, Rui X, Caiyun F, Chunyu Y, Haiyan C, Yi C. 900 MHz Radiofrequency Field Induces Mitochondrial Unfolded Protein Response in Mouse Bone Marrow Stem Cells. Frontiers in Public Health. 9:1265, 2021.

Wolke, S., Neibig, U., Elsner, R., Gollnick, F., Meyer, R. Calcium homeostasis of isolated heart muscle cells exposed to pulsed high-frequency electromagnetic fields. Bioelectromagnetics. 17:144-153, 1996.

[Xie](https://pubmed.ncbi.nlm.nih.gov/?sort=date&term=Xie+W&cauthor_id=34513791), W.,  [Xu](https://pubmed.ncbi.nlm.nih.gov/?sort=date&term=Xu+R&cauthor_id=34513791), R,  [Fan](https://pubmed.ncbi.nlm.nih.gov/?sort=date&term=Fan+C&cauthor_id=34513791),  [C., Yang](https://pubmed.ncbi.nlm.nih.gov/?sort=date&term=Yang+C&cauthor_id=34513791), C.,  [Chen](https://pubmed.ncbi.nlm.nih.gov/?sort=date&term=Chen+H&cauthor_id=34513791), H., [Cao](https://pubmed.ncbi.nlm.nih.gov/?sort=date&term=Cao+Y&cauthor_id=34513791), Y. 900 MHz radiofrequency field induces mitochondrial unfolded protein response in mouse bone marrow stem cells. Front. Public Health. 9:724239, 2021.

[Xu](https://pubmed.ncbi.nlm.nih.gov/?sort=date&term=Xu+Y&cauthor_id=33070827) [Y](https://pubmed.ncbi.nlm.nih.gov/33070827/#affiliation-1),  [Zheng](https://pubmed.ncbi.nlm.nih.gov/?sort=date&term=Zheng+ZA&cauthor_id=33070827) [ZA](https://pubmed.ncbi.nlm.nih.gov/33070827/#affiliation-1),  [Zhu](https://pubmed.ncbi.nlm.nih.gov/?sort=date&term=Zhu+T&cauthor_id=33070827) [T](https://pubmed.ncbi.nlm.nih.gov/33070827/#affiliation-1), [Zhu](https://pubmed.ncbi.nlm.nih.gov/?sort=date&term=Zhu+B&cauthor_id=33070827) [B](https://pubmed.ncbi.nlm.nih.gov/33070827/#affiliation-1),  [Feng](https://pubmed.ncbi.nlm.nih.gov/?sort=date&term=Feng+C&cauthor_id=33070827) [C](https://pubmed.ncbi.nlm.nih.gov/33070827/#affiliation-1),  [Chen](https://pubmed.ncbi.nlm.nih.gov/?sort=date&term=Chen+Y&cauthor_id=33070827) [Y](https://pubmed.ncbi.nlm.nih.gov/33070827/#affiliation-1),  [Qin](https://pubmed.ncbi.nlm.nih.gov/?sort=date&term=Qin+F&cauthor_id=33070827) [F.](https://pubmed.ncbi.nlm.nih.gov/33070827/#affiliation-1) [Joint effects of nano-selenium and nano-cerium on the male reproductive function of mice exposed to microwave radiation] Wei Sheng Yan Jiu 49(5):795-801, 2020. [Article in Chinese]

Yavas MC, Kilitci A, Çelik E, Yegin K, Sirav B, Varol S. Rat brain and testicular tissue effects of radiofrequency radiation exposure: Histopathological, DNA damage of brain and qRT-PCR analysis. INTERNATIONAL JOURNAL OF RADIATION RESEARCH. JUL 2024. 22(3):529-536. doi: 10.61186/ijrr.22.3.529

[Yilmaz](https://pubmed.ncbi.nlm.nih.gov/?sort=date&term=Yilmaz+A&cauthor_id=27427155) [A](https://pubmed.ncbi.nlm.nih.gov/27427155/#affiliation-1), [Tumkaya](https://pubmed.ncbi.nlm.nih.gov/?sort=date&term=Tumkaya+L&cauthor_id=27427155) [L](https://pubmed.ncbi.nlm.nih.gov/27427155/#affiliation-2), [Akyildiz](https://pubmed.ncbi.nlm.nih.gov/?sort=date&term=Akyildiz+K&cauthor_id=27427155) [K](https://pubmed.ncbi.nlm.nih.gov/27427155/#affiliation-2),  [Kalkan](https://pubmed.ncbi.nlm.nih.gov/?sort=date&term=Kalkan+Y&cauthor_id=27427155) [Y](https://pubmed.ncbi.nlm.nih.gov/27427155/#affiliation-2),  [Bodur](https://pubmed.ncbi.nlm.nih.gov/?sort=date&term=Bodur+AF&cauthor_id=27427155) [AF](https://pubmed.ncbi.nlm.nih.gov/27427155/#affiliation-3),  [Sargin](https://pubmed.ncbi.nlm.nih.gov/?sort=date&term=Sargin+F&cauthor_id=27427155) [F](https://pubmed.ncbi.nlm.nih.gov/27427155/#affiliation-4),  [Efe](https://pubmed.ncbi.nlm.nih.gov/?sort=date&term=Efe+H&cauthor_id=27427155) [F](https://pubmed.ncbi.nlm.nih.gov/27427155/#affiliation-1),  [Uydu](https://pubmed.ncbi.nlm.nih.gov/?sort=date&term=Uydu+HA&cauthor_id=27427155) [HA](https://pubmed.ncbi.nlm.nih.gov/27427155/#affiliation-1),  [Yazici](https://pubmed.ncbi.nlm.nih.gov/?sort=date&term=Yazici+ZA&cauthor_id=27427155) [ZA.](https://pubmed.ncbi.nlm.nih.gov/27427155/#affiliation-5) Lasting hepatotoxic effects of prenatal mobile phone exposure. J Matern Fetal Neonatal Med 30(11):1355-1359, 2017.

Yilmaz H, Tümkaya L, Mercantepe T, Yılmaz A, Gül F, Suzan ZT. Effects of 5G mobile phone network electromagnetic field exposure on testicular endoplasmic reticulum stress and the protective role of coenzyme Q10. Arch Med Res. 56(4):103157, 2025.

[Yüksel M](http://www.ncbi.nlm.nih.gov/pubmed/?term=Y%C3%BCksel%20M%5BAuthor%5D&cauthor=true&cauthor_uid=26578367), [Nazıroğlu M](http://www.ncbi.nlm.nih.gov/pubmed/?term=Naz%C4%B1ro%C4%9Flu%20M%5BAuthor%5D&cauthor=true&cauthor_uid=26578367), [Özkaya MO](http://www.ncbi.nlm.nih.gov/pubmed/?term=%C3%96zkaya%20MO%5BAuthor%5D&cauthor=true&cauthor_uid=26578367). Long-term exposure to electromagnetic radiation from mobile phones and Wi-Fi devices decreases plasma prolactin, progesterone, and estrogen levels but increases uterine oxidative stress in pregnant rats and their offspring. [Endocrine.](http://www.ncbi.nlm.nih.gov/pubmed/26578367) 52(2):352-362, 2016.

[Yurekli, A.I](http://www.ncbi.nlm.nih.gov/entrez/query.fcgi?db=pubmed&cmd=Search&itool=pubmed_AbstractPlus&term=%252522Yurekli+AI%252522%25255BAuthor%25255D)., [Ozkan, M](http://www.ncbi.nlm.nih.gov/entrez/query.fcgi?db=pubmed&cmd=Search&itool=pubmed_AbstractPlus&term=%252522Ozkan+M%252522%25255BAuthor%25255D)., [Kalkan, T](http://www.ncbi.nlm.nih.gov/entrez/query.fcgi?db=pubmed&cmd=Search&itool=pubmed_AbstractPlus&term=%252522Kalkan+T%252522%25255BAuthor%25255D)., [Saybasili, H](http://www.ncbi.nlm.nih.gov/entrez/query.fcgi?db=pubmed&cmd=Search&itool=pubmed_AbstractPlus&term=%252522Saybasili+H%252522%25255BAuthor%25255D)., [Tuncel, H](http://www.ncbi.nlm.nih.gov/entrez/query.fcgi?db=pubmed&cmd=Search&itool=pubmed_AbstractPlus&term=%252522Tuncel+H%252522%25255BAuthor%25255D)., [Atukeren, P](http://www.ncbi.nlm.nih.gov/entrez/query.fcgi?db=pubmed&cmd=Search&itool=pubmed_AbstractPlus&term=%252522Atukeren+P%252522%25255BAuthor%25255D)., [Gumustas, K](http://www.ncbi.nlm.nih.gov/entrez/query.fcgi?db=pubmed&cmd=Search&itool=pubmed_AbstractPlus&term=%252522Gumustas+K%252522%25255BAuthor%25255D)., [Seker, S](http://www.ncbi.nlm.nih.gov/entrez/query.fcgi?db=pubmed&cmd=Search&itool=pubmed_AbstractPlus&term=%252522Seker+S%252522%25255BAuthor%25255D). GSM base station electromagnetic radiation and oxidative stress in rats. Electromagn Biol Med. 25:177-188, 2006.

Zeni O, Sannino A, Romeo S, Massaa R, Sarti M, Reddy AB, et al. Induction of an adaptive response in human blood lymphocytes exposed to radiofrequency fields: Influence of the universal mobile telecommunication system (UMTS) signal and the specific absorption rate. Mutat Res. 747: 29-35, 2012.

Zong, C., Ji, Y., He, Q., Zhu, S., Qin, F., Tong, J., Cao, Y. Adaptive response in mice exposed to 900 MHz radiofrequency fields: Bleomycin-induced DNA and oxidative damage/repair. Int J Radiat Biol. 91: 270-276, 2015.

[Zosangzuali](https://pubmed.ncbi.nlm.nih.gov/?sort=pubdate&term=Zosangzuali+M&cauthor_id=33687298) [M](https://pubmed.ncbi.nlm.nih.gov/33687298/#affiliation-1),  [Lalremruati](https://pubmed.ncbi.nlm.nih.gov/?sort=pubdate&term=Lalremruati+M&cauthor_id=33687298) [M](https://pubmed.ncbi.nlm.nih.gov/33687298/#affiliation-1), [Lalmuansangi](https://pubmed.ncbi.nlm.nih.gov/?sort=pubdate&term=Lalmuansangi+C&cauthor_id=33687298) [C](https://pubmed.ncbi.nlm.nih.gov/33687298/#affiliation-1), [Nghakliana](https://pubmed.ncbi.nlm.nih.gov/?sort=pubdate&term=Nghakliana+F&cauthor_id=33687298) [F](https://pubmed.ncbi.nlm.nih.gov/33687298/#affiliation-1),  [Pachuau](https://pubmed.ncbi.nlm.nih.gov/?sort=pubdate&term=Pachuau+L&cauthor_id=33687298) [L](https://pubmed.ncbi.nlm.nih.gov/33687298/#affiliation-2),  [P](https://pubmed.ncbi.nlm.nih.gov/33687298/#affiliation-3), [Siama](https://pubmed.ncbi.nlm.nih.gov/?sort=pubdate&term=Zothan+Siama&cauthor_id=33687298) [Z^.^](https://pubmed.ncbi.nlm.nih.gov/33687298/#affiliation-1) Effects of radiofrequency electromagnetic radiation emitted from a mobile phone base station on the redox homeostasis in different organs of Swiss albino mice. Electromagn Biol Med 40:393-407, 2021.

**There are 16 ‘low intensity’ studies that reported no significant effects:**

[Bornhausen](https://pubmed.ncbi.nlm.nih.gov/?sort=date&term=Bornhausen+M&cauthor_id=11102946) [M](https://pubmed.ncbi.nlm.nih.gov/11102946/#affiliation-1), [Scheingraber](https://pubmed.ncbi.nlm.nih.gov/?sort=date&term=Scheingraber+H&cauthor_id=11102946) H. Prenatal exposure to 900 MHz, cell-phone electromagnetic fields had no effect on operant-behavior performances of adult rats. Bioelectromagnetics 21(8):566-574, 2000. (0.0175-0.075 W/kg) (900 MHz. 217 Hz pulse-modulation during pregnancy)

[Borzoueisileh](https://pubmed.ncbi.nlm.nih.gov/?sort=pubdate&term=Borzoueisileh+S&cauthor_id=32874440) S, [Shabestani Monfared](https://pubmed.ncbi.nlm.nih.gov/?sort=pubdate&term=Shabestani+Monfared+A&cauthor_id=32874440) [A](https://pubmed.ncbi.nlm.nih.gov/32874440/#affiliation-3),  [Ghorbani](https://pubmed.ncbi.nlm.nih.gov/?sort=pubdate&term=Ghorbani+H&cauthor_id=32874440) [H](https://pubmed.ncbi.nlm.nih.gov/32874440/#affiliation-4),  [Mortazavi](https://pubmed.ncbi.nlm.nih.gov/?sort=pubdate&term=Mortazavi+SMJ&cauthor_id=32874440) [SMJ](https://pubmed.ncbi.nlm.nih.gov/32874440/#affiliation-5), [Zabihi](https://pubmed.ncbi.nlm.nih.gov/?sort=pubdate&term=Zabihi+E&cauthor_id=32874440) [E](https://pubmed.ncbi.nlm.nih.gov/32874440/#affiliation-1),  [Pouramir](https://pubmed.ncbi.nlm.nih.gov/?sort=pubdate&term=Pouramir+M&cauthor_id=32874440) [M](https://pubmed.ncbi.nlm.nih.gov/32874440/#affiliation-1),  [Doustimotlagh](https://pubmed.ncbi.nlm.nih.gov/?sort=pubdate&term=Doustimotlagh+AH&cauthor_id=32874440) [AH](https://pubmed.ncbi.nlm.nih.gov/32874440/#affiliation-6),  [Shafiee](https://pubmed.ncbi.nlm.nih.gov/?sort=pubdate&term=Shafiee+M&cauthor_id=32874440) [M](https://pubmed.ncbi.nlm.nih.gov/32874440/#affiliation-7), [Niksirat](https://pubmed.ncbi.nlm.nih.gov/?sort=pubdate&term=Niksirat+F&cauthor_id=32874440) F. Assessment of function, histopathological changes, and oxidative stress in liver tissue due to ionizing and non-ionizing radiations. Caspian J Intern Med 11(3):315-323, 2020. (0.00557-0.092 W/kg) (radiation from a GSM 900/1800 MHz mobile phone 12 h/day for 14 days)

[Dasdag S](http://www.ncbi.nlm.nih.gov/sites/entrez?Db=pubmed&Cmd=Search&Term=%22Dasdag%20S%22%5BAuthor%5D&itool=EntrezSystem2.PEntrez.Pubmed.Pubmed_ResultsPanel.Pubmed_RVAbstractPlus), [Akdag MZ](http://www.ncbi.nlm.nih.gov/sites/entrez?Db=pubmed&Cmd=Search&Term=%22Akdag%20MZ%22%5BAuthor%5D&itool=EntrezSystem2.PEntrez.Pubmed.Pubmed_ResultsPanel.Pubmed_RVAbstractPlus), [Ulukaya E](http://www.ncbi.nlm.nih.gov/sites/entrez?Db=pubmed&Cmd=Search&Term=%22Ulukaya%20E%22%5BAuthor%5D&itool=EntrezSystem2.PEntrez.Pubmed.Pubmed_ResultsPanel.Pubmed_RVAbstractPlus), [Uzunlar AK](http://www.ncbi.nlm.nih.gov/sites/entrez?Db=pubmed&Cmd=Search&Term=%22Uzunlar%20AK%22%5BAuthor%5D&itool=EntrezSystem2.PEntrez.Pubmed.Pubmed_ResultsPanel.Pubmed_RVAbstractPlus), [Yegin D](http://www.ncbi.nlm.nih.gov/sites/entrez?Db=pubmed&Cmd=Search&Term=%22Yegin%20D%22%5BAuthor%5D&itool=EntrezSystem2.PEntrez.Pubmed.Pubmed_ResultsPanel.Pubmed_RVAbstractPlus). Mobile phone exposure does not induce apoptosis on spermatogenesis in rats. [Arch Med Res.](javascript:AL_get(this,%20'jour',%20'Arch%20Med%20Res.');) 39(1):40-44, 2008. (SAR 0.07-0.57 W/kg) (900 MHz; 2 h/day, 7 days/week for 10 months)

[Gurisik](https://pubmed.ncbi.nlm.nih.gov/?term=Gurisik+E&cauthor_id=16877012) [E](https://pubmed.ncbi.nlm.nih.gov/16877012/#affiliation-1), [Warton](https://pubmed.ncbi.nlm.nih.gov/?term=Warton+K&cauthor_id=16877012) K,  [Martin](https://pubmed.ncbi.nlm.nih.gov/?term=Martin+DK&cauthor_id=16877012) DK,  [Valenzuela](https://pubmed.ncbi.nlm.nih.gov/?term=Valenzuela+SM&cauthor_id=16877012) SM. An in vitro study of the effects of exposure to a GSM signal in two human cell lines: monocytic U937 and neuroblastoma SK-N-SH. Cell Biol Int 30(10):793-759, 2006. (0.2 W/kg) (900 MHz, 217-Hz pulses for 2 h)

Haidar J, Nabos P, Orlacchio R, Hurtier A, de Gannes FP, Rambert J, Cario-André M, Moisan F, Rezvani HR, Lagroye I, Leveque P, Arnaud-Cormos D, Percherancier Y. Impact of in vitro exposure to 5G-modulated 3.5 GHz fields on oxidative stress and DNA repair in skin cells. Sci Rep. 15(1):31214, 2025. (0.08W/kg) (5G-modulated 3.5 GHz RFR for 24 h.)

Ilgaz NS, Karamazı Y, Emre M, Toyran T, Karaoğlan Ö, Emre T, Dönmez Kutlu M, Öksüz Üçkayabaşı H, Aydın Ç, Yılmaz MB. Genotoxic and histopathological effects of 6 GHz radiofrequency electromagnetic radiation on rat liver tissue. Electromagn Biol Med. 2025 Jul 22:1-12. doi: 10.1080/15368378.2025.2534381. Epub ahead of print. (0.065 W/kg) (6 GHz, 4 h/day for 42 days)

[Imai N](http://www.ncbi.nlm.nih.gov/pubmed?term=%22Imai%20N%22%5BAuthor%5D), [Kawabe M](http://www.ncbi.nlm.nih.gov/pubmed?term=%22Kawabe%20M%22%5BAuthor%5D), [Hikage T](http://www.ncbi.nlm.nih.gov/pubmed?term=%22Hikage%20T%22%5BAuthor%5D), [Nojima T](http://www.ncbi.nlm.nih.gov/pubmed?term=%22Nojima%20T%22%5BAuthor%5D), [Takahashi S](http://www.ncbi.nlm.nih.gov/pubmed?term=%22Takahashi%20S%22%5BAuthor%5D), [Shirai T](http://www.ncbi.nlm.nih.gov/pubmed?term=%22Shirai%20T%22%5BAuthor%5D). Effects on rat testis of 1.95-GHz W-CDMA for IMT-2000 cellular phones. [Syst Biol Reprod Med.](javascript:AL_get(this,%20'jour',%20'Syst%20Biol%20Reprod%20Med.');) 57(4):204-209, 2011. (0.08 W/kg) (1950 MHz W-CDMA signal; 5 h/day, 7 days/week for 5 weeks)

[Krivova](https://pubmed.ncbi.nlm.nih.gov/?sort=date&term=Krivova+NA&cauthor_id=38704416) [NA](https://pubmed.ncbi.nlm.nih.gov/38704416/#full-view-affiliation-1),  [Kudabaeva](https://pubmed.ncbi.nlm.nih.gov/?sort=date&term=Kudabaeva+MS&cauthor_id=38704416) [MS](https://pubmed.ncbi.nlm.nih.gov/38704416/#full-view-affiliation-2),  [Zaeva](https://pubmed.ncbi.nlm.nih.gov/?sort=date&term=Zaeva+OB&cauthor_id=38704416) [OB](https://pubmed.ncbi.nlm.nih.gov/38704416/#full-view-affiliation-3),  [Borodina](https://pubmed.ncbi.nlm.nih.gov/?sort=date&term=Borodina+SV&cauthor_id=38704416) [SV](https://pubmed.ncbi.nlm.nih.gov/38704416/#full-view-affiliation-4),  [Lepekhina](https://pubmed.ncbi.nlm.nih.gov/?sort=date&term=Lepekhina+TB&cauthor_id=38704416) [TB](https://pubmed.ncbi.nlm.nih.gov/38704416/#full-view-affiliation-4),  [Pavlenko](https://pubmed.ncbi.nlm.nih.gov/?sort=date&term=Pavlenko+OA&cauthor_id=38704416) [OA](https://pubmed.ncbi.nlm.nih.gov/38704416/#full-view-affiliation-5),  [Makhmanazarov](https://pubmed.ncbi.nlm.nih.gov/?sort=date&term=Makhmanazarov+RM&cauthor_id=38704416) [RM](https://pubmed.ncbi.nlm.nih.gov/38704416/#full-view-affiliation-6),  [Kokin](https://pubmed.ncbi.nlm.nih.gov/?sort=date&term=Kokin+DS&cauthor_id=38704416) [DS](https://pubmed.ncbi.nlm.nih.gov/38704416/#full-view-affiliation-6),  [Shipilov](https://pubmed.ncbi.nlm.nih.gov/?sort=date&term=Shipilov+SE&cauthor_id=38704416) SE. The effect of exposure to RF-EMF from the laboratory simulator of 5G NR base station on physiological parameters and cognitive abilities of male wistar rats of different ages. Sci Rep 14(1):10283, 2024. (0.0322-0.0017 W/kg) (2400 MHz, 4 weeks)

[Nazıroğlu M](http://www.ncbi.nlm.nih.gov/pubmed/?term=Naz%C4%B1ro%C4%9Flu%20M%5BAuthor%5D&cauthor=true&cauthor_uid=25381485), [Özkan FF](http://www.ncbi.nlm.nih.gov/pubmed/?term=%C3%96zkan%20FF%5BAuthor%5D&cauthor=true&cauthor_uid=25381485), [Hapil SR](http://www.ncbi.nlm.nih.gov/pubmed/?term=Hapil%20SR%5BAuthor%5D&cauthor=true&cauthor_uid=25381485), [Ghazizadeh V](http://www.ncbi.nlm.nih.gov/pubmed/?term=Ghazizadeh%20V%5BAuthor%5D&cauthor=true&cauthor_uid=25381485), [Çiğ B](http://www.ncbi.nlm.nih.gov/pubmed/?term=%C3%87i%C4%9F%20B%5BAuthor%5D&cauthor=true&cauthor_uid=25381485). Epilepsy but not mobile phone frequency (900 MHz) induces apoptosis and calcium entry in hippocampus of epileptic rat: involvement of TRPV1 channels. [J Membr Biol.](http://www.ncbi.nlm.nih.gov/pubmed/25381485) 248(1):83-91, 2015. (0.23 x 10^-3^ W/kg) (900 MHz, 217 Hz pulses, 1 h)

[Okatan](https://pubmed.ncbi.nlm.nih.gov/?sort=date&term=Okatan+D%C3%96&cauthor_id=30009691) [D](https://pubmed.ncbi.nlm.nih.gov/30009691/#affiliation-1)Ö,  [Okatan](https://pubmed.ncbi.nlm.nih.gov/?sort=date&term=Okatan+AE&cauthor_id=30009691) [AE](https://pubmed.ncbi.nlm.nih.gov/30009691/#affiliation-2),  [Hancı](https://pubmed.ncbi.nlm.nih.gov/?sort=date&term=Hanc%C4%B1+H&cauthor_id=30009691) [H](https://pubmed.ncbi.nlm.nih.gov/30009691/#affiliation-3),  [Demir](https://pubmed.ncbi.nlm.nih.gov/?sort=date&term=Demir+S&cauthor_id=30009691) [S](https://pubmed.ncbi.nlm.nih.gov/30009691/#affiliation-4),  [Yaman](https://pubmed.ncbi.nlm.nih.gov/?sort=date&term=Yaman+S%C3%96&cauthor_id=30009691) [S](https://pubmed.ncbi.nlm.nih.gov/30009691/#affiliation-1)Ö,  [Çolakoğlu](https://pubmed.ncbi.nlm.nih.gov/?sort=date&term=%C3%87olako%C4%9Flu+S&cauthor_id=30009691) [S](https://pubmed.ncbi.nlm.nih.gov/30009691/#affiliation-6),  [Odacı](https://pubmed.ncbi.nlm.nih.gov/?sort=date&term=Odac%C4%B1+E&cauthor_id=30009691) [E.](https://pubmed.ncbi.nlm.nih.gov/30009691/#affiliation-3) Effects of 900-MHz electromagnetic fields exposure throughout middle/late adolescence on the kidney morphology and biochemistry of the female rat. Toxicol Ind Health 34(10):693-702, 2018. (0.012 W/kg) (900 MHz, 1 h/day from postnatal days 35-59)

[Porcher](https://pubmed.ncbi.nlm.nih.gov/?sort=date&term=Porcher+A&cauthor_id=37408527) [A](https://pubmed.ncbi.nlm.nih.gov/37408527/#full-view-affiliation-1), [Wilmot](https://pubmed.ncbi.nlm.nih.gov/?sort=date&term=Wilmot+N&cauthor_id=37408527) [N](https://pubmed.ncbi.nlm.nih.gov/37408527/#full-view-affiliation-2),  [Bonnet](https://pubmed.ncbi.nlm.nih.gov/?sort=date&term=Bonnet+P&cauthor_id=37408527) [P](https://pubmed.ncbi.nlm.nih.gov/37408527/#full-view-affiliation-1),  [Procaccio](https://pubmed.ncbi.nlm.nih.gov/?sort=date&term=Procaccio+V&cauthor_id=37408527) [V](https://pubmed.ncbi.nlm.nih.gov/37408527/#full-view-affiliation-2),  [Vian](https://pubmed.ncbi.nlm.nih.gov/?sort=date&term=Vian+A&cauthor_id=37408527) [A.](https://pubmed.ncbi.nlm.nih.gov/37408527/#full-view-affiliation-3) Changes in gene expression after exposing Arabidopsis thaliana plants to nanosecond high amplitude electromagnetic field pulses. Bioelectromagnetics 45(1):4-15, 2024. (10^-13^ to 10^-16^ W/kg) (30,000 EMP over 10 min (180 KHz), pulse duration 500 ps)

[Shirai T](https://www.ncbi.nlm.nih.gov/pubmed/?term=Shirai%20T%5BAuthor%5D&cauthor=true&cauthor_uid=27694283), [Wang J](https://www.ncbi.nlm.nih.gov/pubmed/?term=Wang%20J%5BAuthor%5D&cauthor=true&cauthor_uid=27694283), [Kawabe M](https://www.ncbi.nlm.nih.gov/pubmed/?term=Kawabe%20M%5BAuthor%5D&cauthor=true&cauthor_uid=27694283), [Wake K](https://www.ncbi.nlm.nih.gov/pubmed/?term=Wake%20K%5BAuthor%5D&cauthor=true&cauthor_uid=27694283), [Watanabe SI](https://www.ncbi.nlm.nih.gov/pubmed/?term=Watanabe%20SI%5BAuthor%5D&cauthor=true&cauthor_uid=27694283), [Takahashi S](https://www.ncbi.nlm.nih.gov/pubmed/?term=Takahashi%20S%5BAuthor%5D&cauthor=true&cauthor_uid=27694283), [Fujiwara O](https://www.ncbi.nlm.nih.gov/pubmed/?term=Fujiwara%20O%5BAuthor%5D&cauthor=true&cauthor_uid=27694283). No adverse effects detected for simultaneous whole-body exposure to multiple-frequency radiofrequency electromagnetic fields for rats in the intrauterine and pre- and post-weaning periods. [J Radiat Res.](https://www.ncbi.nlm.nih.gov/pubmed/27694283) 58(1):48-58, 2017. (SAR 0.384-0.433 W/kg) (eight different communication signal EMFs (two of 800 MHz band, two of 2 GHz band, one of 2.4 GHz band, two of 2.5 GHz band and one of 5.2 GHz band; 20 h per day from gestational Day 7 to weaning, and F_1_ offspring rats (46-48 F1 pups per group) were then exposed up to 6 weeks of age also for 20 h per day.)

[Silva V](http://www.ncbi.nlm.nih.gov/pubmed/?term=Silva%20V%5BAuthor%5D&cauthor=true&cauthor_uid=26689947), [Hilly O](http://www.ncbi.nlm.nih.gov/pubmed/?term=Hilly%20O%5BAuthor%5D&cauthor=true&cauthor_uid=26689947), [Strenov Y](http://www.ncbi.nlm.nih.gov/pubmed/?term=Strenov%20Y%5BAuthor%5D&cauthor=true&cauthor_uid=26689947), [Tzabari C](http://www.ncbi.nlm.nih.gov/pubmed/?term=Tzabari%20C%5BAuthor%5D&cauthor=true&cauthor_uid=26689947), [Hauptman Y](http://www.ncbi.nlm.nih.gov/pubmed/?term=Hauptman%20Y%5BAuthor%5D&cauthor=true&cauthor_uid=26689947), [Feinmesser R](http://www.ncbi.nlm.nih.gov/pubmed/?term=Feinmesser%20R%5BAuthor%5D&cauthor=true&cauthor_uid=26689947). Effect of cell phone-like electromagnetic radiation on primary human thyroid cells. [Int J Radiat Biol.](http://www.ncbi.nlm.nih.gov/pubmed/26689947) 92(2):107-115, 2016. (900 MHz for 3 or 16 h at 0.082 W/kg; 895 MHz for 65 h at 0.17 W/kg)

[Takahashi S](http://www.ncbi.nlm.nih.gov/pubmed?term=Takahashi%20S%5BAuthor%5D&cauthor=true&cauthor_uid=20199221), [Imai N](http://www.ncbi.nlm.nih.gov/pubmed?term=Imai%20N%5BAuthor%5D&cauthor=true&cauthor_uid=20199221), [Nabae K](http://www.ncbi.nlm.nih.gov/pubmed?term=Nabae%20K%5BAuthor%5D&cauthor=true&cauthor_uid=20199221), [Wake K](http://www.ncbi.nlm.nih.gov/pubmed?term=Wake%20K%5BAuthor%5D&cauthor=true&cauthor_uid=20199221), [Kawai H](http://www.ncbi.nlm.nih.gov/pubmed?term=Kawai%20H%5BAuthor%5D&cauthor=true&cauthor_uid=20199221), [Wang J](http://www.ncbi.nlm.nih.gov/pubmed?term=Wang%20J%5BAuthor%5D&cauthor=true&cauthor_uid=20199221), [Watanabe S](http://www.ncbi.nlm.nih.gov/pubmed?term=Watanabe%20S%5BAuthor%5D&cauthor=true&cauthor_uid=20199221), [Kawabe M](http://www.ncbi.nlm.nih.gov/pubmed?term=Kawabe%20M%5BAuthor%5D&cauthor=true&cauthor_uid=20199221), [Fujiwara O](http://www.ncbi.nlm.nih.gov/pubmed?term=Fujiwara%20O%5BAuthor%5D&cauthor=true&cauthor_uid=20199221), [Ogawa K](http://www.ncbi.nlm.nih.gov/pubmed?term=Ogawa%20K%5BAuthor%5D&cauthor=true&cauthor_uid=20199221), [Tamano S](http://www.ncbi.nlm.nih.gov/pubmed?term=Tamano%20S%5BAuthor%5D&cauthor=true&cauthor_uid=20199221), [Shirai T](http://www.ncbi.nlm.nih.gov/pubmed?term=Shirai%20T%5BAuthor%5D&cauthor=true&cauthor_uid=20199221). Lack of adverse effects of whole-body exposure to a mobile telecommunication electromagnetic field on the rat fetus. [Radiat Res.](http://www.ncbi.nlm.nih.gov/pubmed/20199221) 173(3):362-372, 2010.( (SAR for the dams was 0.066-0.093 W/kg. The SAR for the fetuses and the F(1) progeny was 0.068-0.146 W/kg) (2140 MHz signals were applied for 20 h per day during the gestation and lactation periods.)

[Tumkaya L](http://www.ncbi.nlm.nih.gov/pubmed?term=Tumkaya%20L%5BAuthor%5D&cauthor=true&cauthor_uid=24097363), [Kalkan Y](http://www.ncbi.nlm.nih.gov/pubmed?term=Kalkan%20Y%5BAuthor%5D&cauthor=true&cauthor_uid=24097363), [Bas O](http://www.ncbi.nlm.nih.gov/pubmed?term=Bas%20O%5BAuthor%5D&cauthor=true&cauthor_uid=24097363), [Yilmaz A](http://www.ncbi.nlm.nih.gov/pubmed?term=Yilmaz%20A%5BAuthor%5D&cauthor=true&cauthor_uid=24097363). Mobile phone radiation during pubertal development has no effect on testicular histology in rats. [Toxicol Ind Health.](http://www.ncbi.nlm.nih.gov/pubmed/24097363) 32(2):328-336, 2016. (SAR 0.48 W/kg) (radiation from a mobile phone 895-915 MHz, 217 Hz modulation, 1 h/day for 45 days).

Zeni, O., Di Pietro, R., d'Ambrosio, G., Massa, R., Capri, M., Naarala, J., Juutilainen, J. and Scarfi, M. R. Formation of reactive oxygen species in L929 cells after exposure to 900 MHz RF radiation with and without co-exposure to 3-chloro-4-(dichloromethyl)-5-hydroxy-2(5H)-furanone. Radiat. Res. 167: 306-311, 2007. (0.3 W/*k*g) (GSM signal or 10 or 30 min)
