# Supplementary material for: Flora and fauna: how nonhuman species interact with natural and man-made EMF at ecosystem levels and public policy recommendations
Source: Front Public Health. 2025 Nov 19;13:1693873. doi: 10.3389/fpubh.2025.1693873 (PMC12675998; doi:10.3389/fpubh.2025.1693873)
Supplement: Supplementary file 2 [file Supplementary_file_2.docx]

**November 3, 2025**

**Supplement Table 2.**

**Small Sampling of Studies**

**(For more information, see Supplements in Levitt, Lai, and Manville b 2021)**

**Birds:**

Balmori, A. 2005. Possible Effects of Electromagnetic Fields from Phone Masts on a Population of White Stork (Ciconia ciconia). Electromagn Biol Med 24:109-119.

Balmori, A., Hallberg, O. 2007. The urban decline of the House Sparrow (*Passer domestics*): a possible link with electromagnetic radiation. Electromagn Biol Med 26:141-151.

Beason RC, Semm P. Responses of neurons to an amplitude modulated microwave stimulus. *Neuroscience Letters* (2002) 333:175–178. doi: 10.1016/S0304-3940(02)00903-5

Everaert, J., Bauwens, D. 2007. A possible effect of electromagnetic radiation from mobile phone base stations on the number of breeding House Sparrows (Passer domesticus). [Electromagn Biol Med.](https://www.ncbi.nlm.nih.gov/pubmed/17454083) 26:63-72.

Fernie, K.J., Bird, D.M., Petitclerc. D. 1999. Effects of electromagnetic fields on photophasic circulating melatonin levels in American kestrels. Environ Health Perspect.107: 901–904.

Fernie, K.J, Bird, D.M., Dawson, R.D., Lague, P.C. 2000. Effects of electromagnetic fields on the reproductive success of American kestrels. Physiol. Biochem. Zool. 73 60–65.

Fernie, K.J., Leonard, N.J., Bird. D.M. 2000. Behavior of free-ranging and captive American kestrels under electromagnetic fields. J. Toxicol. Environ. Health, Part A 59. 597–603.

Fernie, K.J., Bird, D.M. 2001. Evidence of oxidative stress in American kestrels exposed to electromagnetic fields. Environ. Res. 86:198-207.

[Fernie, K.J](http://www.ncbi.nlm.nih.gov/pubmed?term=%22Fernie%20KJ%22%5bAuthor%5d).[,](http://www.ncbi.nlm.nih.gov/pubmed?term=%22Fernie%20KJ%22%5bAuthor%5d) [Reynolds, S.J](http://www.ncbi.nlm.nih.gov/pubmed?term=%22Reynolds%20SJ%22%5bAuthor%5d). 2005. The effects of electromagnetic fields from power lines on avian reproductive biology and physiology: a review. [Toxicol Environ Health B Crit Rev.](http://www.ncbi.nlm.nih.gov/pubmed/15804752) 8:127-140.

Ritz, T., Thalau, P., Phillips, J. B., Wiltschko, R., Wiltschko, W. 2004. Resonance effects indicate a radical pair mechanism for avian magnetic compass. Nature 429:177–180.

Ritz, T., Wiltschko, R., Hore, P. J., Rodgers, C. T., Stapput, K., Thalau, P., Timmel, C. R., Wiltschko, W. 2009. Magnetic compass of birds is based on a molecule with optimal directional sensitivity. Biophys. J. 96:3451–3457.

Tanner, J.A. 1966. Effect of microwave radiation on birds. Nature 210:636.

Tanner, J.A., Romero-Sierra, C., Davie, S.J. 1967. Non-thermal effects of microwave radiation on birds. Nature 216:1139.

Wiltschko, W., Freire, R., Munro, U., Ritz, T., Rogers, L., Thalau, P., Wiltschko, R. 2007. The magnetic compass of domestic chickens, Gallus gallus. J. Exp. Biol. 210:2300–2310.

Wiltschko R., Wiltschko W. 2014. Sensing magnetic directions in birds: radical pair processes involving cryptochrome. Biosensors 4:221–243.

Wiltschko, R., Thalau, P., Gehring, D., Nießner, C., Ritz, T., Wiltschko, W. 2015. Magnetoreception in birds: the effect of radio-frequency fields. J. R. Soc. Interface 12:20141103.

**Insects:**

As N, Karan Y, Dizman S, Sayi BC, Kuvanci A, Cinbirtoğlu Ş, Öztürk SH, Şahin ME. 2025. An experimental study on the effect of non-ionizing electromagnetic fields on honey bees, Electromagn Biol Med. 44:65-78.

Cammaerts, M.C., De Doncker, P., Patris, X., Bellens, F., Rachidi, Z., Cammaerts, D. 2012. GSM900 MHz radiation inhibits ants’ association between food sites and encountered cues. Electromagn Biol Med, 31:151-165.

[Cammaerts, M.C](https://www.ncbi.nlm.nih.gov/pubmed/?term=Cammaerts%20MC%5bAuthor%5d&cauthor=true&cauthor_uid=23320633)., [Rachidi, Z](https://www.ncbi.nlm.nih.gov/pubmed/?term=Rachidi%20Z%5bAuthor%5d&cauthor=true&cauthor_uid=23320633)., [Bellens, F](https://www.ncbi.nlm.nih.gov/pubmed/?term=Bellens%20F%5bAuthor%5d&cauthor=true&cauthor_uid=23320633)., [De Doncker, P](https://www.ncbi.nlm.nih.gov/pubmed/?term=De%20Doncker%20P%5bAuthor%5d&cauthor=true&cauthor_uid=23320633). 2013. Food collection and response to pheromones in an ant species exposed to electromagnetic radiation. [Electromagn Biol Med.](https://www.ncbi.nlm.nih.gov/pubmed/23320633) 32:315-332.

Cammaerts, M.C., Vandenbosch, G.A.E., Volski, V. 2014. Effect of short-term GSM radiation at representative levels in society on a biological model: the ant Myrmica sabuleti. J Insect Behav 27:514-526.

Greggers, U., Koch, G., Schmidt, V., Du ̈rr, A., Floriou-Servou, A., Piepenbrock, D., Go ̈pfert, M.C., Menzel, R. 2013. Reception and learning of electric fields in bees. Proc R Soc B 280:20130528.

Guerra, P., [Gegear](http://www.nature.com/ncomms/2014/140624/ncomms5164/full/ncomms5164.html), R.J., [Reppert](http://www.nature.com/ncomms/2014/140624/ncomms5164/full/ncomms5164.html), S.M. 2014. A magnetic compass aids monarch butterfly migration. Nature Commun, 5:4164.

Kirschvink J.L., Padmanabha, S., Boyce, C.K., Oglesby, J. 1997. Measurement of the threshold sensitivity of honeybees to weak, extremely low-frequency magnetic fields. J Exp Biol 200:1363–1368.

Kumar, N. R., Sangwan, S., Badotra, P. 2011. Exposure to cell phone radiations produces biochemical changes in worker honey bees. Toxicol Int. 18:70–72.

Lazaro, A., Chroni, A., Tscheulin, T., Devalez, J., Matsoukas, C., Petanidou, T. 2016. Electromagnetic radiation of mobile telecommunication antennas affects the abundance and composition of wild pollinators. J Insect Conserv 20:315–324.

Mallinson VJ, Woodburn FA, O'Reilly LJ. Weak anthropogenic electric fields affect honeybee foraging. iScience. 2025 May 19;28(6):112550. doi: 10.1016/j.isci.2025.112550. PMID: 40612901; PMCID: PMC12225925.

Migdał P, Plotnik M, Bieńkowski P, Berbeć E, Latarowski K, Białecka N, Murawska A. The influence of an electromagnetic field at a radiofrequency of 900 MHz on the behavior of a honey bee. Agriculture. 15:1266.

Nik Abdull Halim, N.M.H., Mohd Jamili, A.F., Che Dom, N., Abd Rahman, N.H., Jamal Kareem, Z., Dapari, R. 2024. The impact of radiofrequency exposure on Aedes aegypti (Diptera: Culicidae) development. PLoS One. 2024 Feb 27;19(2):e0298738. doi: 10.1371/journal.pone.0298738. PMID: 38412167; PMCID: PMC10898727.

Odemer, R., Odemer, F. 2019. Effects of radiofrequency electromagnetic radiation (RF-EMF) on honey bee queen development and mating success. Sci Total Enviro. 661:553-562.

Shepherd S, Lima MAP, Oliveira EE, Sharkh SM, Jackson CW, Newland PL. Extremely Low Frequency Electromagnetic Fields impair the Cognitive and Motor Abilities of Honey Bees. Sci Rep. 2018 May 21;8(1):7932. doi: 10.1038/s41598-018-26185-y. PMID: 29785039; PMCID: PMC5962564.

Sutton, G.P., Clarke D., Morley E. L., Robert D. 2016. Mechanosensory hairs in bumble bees (Bombus terrestris) detect weak electric fields. Proc Nat Acad Sci. 113:1261-1265.

Treder M, Müller M, Fellner L, Traynor K, Rosenkranz P. 2023. Defined exposure of honey bee colonies to simulated radiofrequency electromagnetic fields (RF-EMF): Negative effects on the homing ability, but not on brood development or longevity. Sci Total Environ. 896:165211.

Treder, M., Glück, M., England, S.J., Traynor, K.S. 2025. Radiofrequency electromagnetic fields reduce bumble bee visitation to flowers, Environ Pollut. 14:126836.

[Vácha M](https://www.ncbi.nlm.nih.gov/pubmed/?term=V%C3%A1cha%20M%5bAuthor%5d&cauthor=true&cauthor_uid=19837889) , [Puzová T](https://www.ncbi.nlm.nih.gov/pubmed/?term=Puzov%C3%A1%20T%5bAuthor%5d&cauthor=true&cauthor_uid=19837889), [Kvícalová M](https://www.ncbi.nlm.nih.gov/pubmed/?term=Kv%C3%ADcalov%C3%A1%20M%5bAuthor%5d&cauthor=true&cauthor_uid=19837889). 2009. Radio frequency magnetic fields disrupt magnetoreception in American cockroach. [J Exp Biol.](https://www.ncbi.nlm.nih.gov/pubmed/19837889?dopt=Abstract) 212(Pt 21):3473-3477.

Vargová, B., Kurimský, J., Cimbala, R., Kosterec, M., Majláth, I., Pipová, N., Tryjanowski, P., Jankowiak, L., Majláthová , V. 2017. Ticks and radio-frequency signals: behavioural response of ticks (*Dermacentor reticulatus*) in a 900 MHz electromagnetic field. Sys Appl Acarol 22: 683–693.

[Vargová, B](https://www.ncbi.nlm.nih.gov/pubmed/?term=Vargov%C3%A1%20B%5bAuthor%5d&cauthor=true&cauthor_uid=29605834)., [Majláth, I](https://www.ncbi.nlm.nih.gov/pubmed/?term=Majl%C3%A1th%20I%5bAuthor%5d&cauthor=true&cauthor_uid=29605834)., [Kurimský, J](https://www.ncbi.nlm.nih.gov/pubmed/?term=Kurimsk%C3%BD%20J%5bAuthor%5d&cauthor=true&cauthor_uid=29605834)., [Cimbala, R](https://www.ncbi.nlm.nih.gov/pubmed/?term=Cimbala%20R%5bAuthor%5d&cauthor=true&cauthor_uid=29605834)., [Kosterec, M](https://www.ncbi.nlm.nih.gov/pubmed/?term=Kosterec%20M%5bAuthor%5d&cauthor=true&cauthor_uid=29605834)., [Tryjanowski, P](https://www.ncbi.nlm.nih.gov/pubmed/?term=Tryjanowski%20P%5bAuthor%5d&cauthor=true&cauthor_uid=29605834)., [Jankowiak, Ł](https://www.ncbi.nlm.nih.gov/pubmed/?term=Jankowiak%20%C5%81%5bAuthor%5d&cauthor=true&cauthor_uid=29605834)., [Raši, T](https://www.ncbi.nlm.nih.gov/pubmed/?term=Ra%C5%A1i%20T%5bAuthor%5d&cauthor=true&cauthor_uid=29605834)., [Majláthová, V](https://www.ncbi.nlm.nih.gov/pubmed/?term=Majl%C3%A1thov%C3%A1%20V%5bAuthor%5d&cauthor=true&cauthor_uid=29605834). 2018. Electromagnetic radiation and behavioural response of ticks: an experimental test. [Exp Appl Acarol.](https://www.ncbi.nlm.nih.gov/pubmed/29605834) 75:85-95.

Vili´c, M.; Žura Žaja, I.; Tkalec, M.; Tucak, P.; Malari´c, K.; Popara, N.; Žura, N.; Paši´c, S.; Gajger, I.T. 2024. Oxidative stress response of honey bee colonies (Apis mellifera L.) during long-term exposure at a frequency of 900 MHz under field conditions. Insects 15:372.

Wang Y, Zhang Z, Zhang L, Liao Y, Cai P. 2025. 3.5GHz radiofrequency electromagnetic fields (RF-EMF) on metabolic disorders in Drosophila melanogaster. Ecotoxicol Environ Saf. 2025 Oct 6;304:119132. doi: 10.1016/j.ecoenv.2025.119132. Epub ahead of print. PMID: 41056677.

**Cows, bats:**

**Rodent studies, too numerous to mention here, are cited in Appendix 1 and in Levitt et al. (2021b)**

Löscher, W., Käs, G. 1998. Behavioral abnormalities in a dairy cow herd near a TV and radio transmitting antenna. Prakt Tierarzt. 79:437-444. (Article in German)

Löscher, W. 2003. Survey of effects of radiofrequency electromagnetic fields on production, health and behavior of farm animals. *Prakt Tierarzt.* 84:11 (Article in German)

[Nicholls, B](https://www.ncbi.nlm.nih.gov/pubmed/?term=Nicholls%20B%5bAuthor%5d&cauthor=true&cauthor_uid=17372629)., [Racey, P.A](https://www.ncbi.nlm.nih.gov/pubmed/?term=Racey%20PA%5bAuthor%5d&cauthor=true&cauthor_uid=17372629). 2007. Bats avoid radar installations: could electromagnetic fields deter bats from colliding with wind turbines? [PLoS One.](https://www.ncbi.nlm.nih.gov/pubmed/17372629) 2:e297, 2007.

Nicholls, B., Racey, P.A. 2009. The aversive effect of electromagnetic radiation on foraging bats: a possible means of discouraging bats from approaching wind turbines. PLoS One. 4:e6246.

Rodriguez, M., Petitclerc, D., Burchard, J.F., Nguyen, D.H., Block, E., Downey, B.R. 2003. Responses of the estrous cycle in dairy cows exposed to electric and magnetic fields (60 Hz) during 8-h photoperiods. Anim Reprod. Sci.15: 11-20. [.](https://www.sciencedirect.com/science/article/pii/S0378432002002737)

**Bacteria and Protozoa: (Implications for antibiotic resistance)**

Cammaerts, M.C., Debeir, O., Cammaerts, R. 2011. Changes in Paramecium caudatum (Protozoa) near a switched-on GSM telephone. Electromagn Biol Med. 30:57-66.

Cellini, L., Grande, R., Di Campli, E., Di Bartolomeo, S., Di Giulio, M., Robuffo, I., Trubiani, O., Mariggio, M. A. 2008. Bacterial response to the exposure of 50 Hz electromagnetic fields. Bioelectromagnetics 29:302-311.

Movahedi, M. M., Nouri, F., Tavakoli Golpaygani, A., Ataee, L., Amani, S., Taheri, M. 2019. Antibacterial susceptibility pattern of the Pseudomonas aeruginosa and Staphylococcus aureus after exposure to electromagnetic waves emitted from mobile phone simulator. J Biomed Phys Eng. 9:637-646.

Potenza, L., Ubaldi, L., De Sanctis, R., De Bellis, R., Cucchiarini, L., Dachà, M. 2004. Effects of a static magnetic field on cell growth and gene expression in *Escherichia coli.* Mutat Res 561:53–62.

Rodriguez-de la Fuente, A.O., Gomez-Flores, R., Heredia-Rojas, J.A., Garcia-Munoz, E.M., Vargas-Villarreal, J., Hernandez-Garcia, M.E., Gonzalez-Salazar, F., Garza-Gonzalez, N.B., Beltcheva, M., Heredia-Rodriguez, O. 2019. Trichomonas vaginalis and Giardia lamblia growth alterations by low-frequency electromagnetic fields. Iran J Parasitol. 14:652-656.

Said-Salman, I., Mortazavi, S. M. J., Khatib, S. E., Mortazavi, S. A., Sihver, L. (2025). 'Bacterial Adaptation to Radiofrequency Electromagnetic Fields Based on Experiences from Ionizing Radiation', Journal of Biomedical Physics and Engineering, (), pp. -. doi: 10.31661/jbpe.v0i0.2405-1770

Said-Salman, I. H., Jebaii F. A., Yusef, H. H., Moustafa, M. E. 2019. Evaluation of Wi-Fi radiation effects on antibiotic susceptibility, metabolic activity and biofilm formation by Escherichia coli 0157H7, Staphylococcus aureus and Staphylococcus Epidermis. J Biomed Phys Eng. 9:579-586.

Salmen, S.H., Alharbi, S.A., Faden, A.A., Wainwright, M. 2018. Evaluation of effect of high frequency electromagnetic field on growth and antibiotic sensitivity of bacteria. Saudi J Biol Sci 25:105–110.

**Amphibians:**

Balmori, A. 2010. Mobile phone mast effects on common frog (Rana temporaria) tadpoles: the city turned into a laboratory. Electromagn Biol Med. 29:31-5.

Balmori, A. 2006. The incidence of electromagnetic pollution on the amphibian decline: Is this an important piece of the puzzle? Toxicol Environ Chem 88: 287–299.

Komazaki, S., Takano, K. 2007. Induction of increase in intracellular calcium concentration of embryonic cells and acceleration of morphogenetic cell movements during amphibian gastrulation , by a 50-Hz magnetic field. J. Exp. Zool. 307A:156–162.

Phillips, J.B., Deutschlander, M.E., Freake, M.J., Borland, S.C. 2001. The role of extraocular photoreceptors in newt magnetic compass orientation: evidence for parallels between light–dependent magnetoreception and polarized light detection in vertebrates. J Exp Biol 204:2543–2552

Phillips, J.B., Jorge, P.E., Muheim, R. 2010. Light-dependent magnetic compass orientation in amphibians and insects: candidate receptors and candidate molecular mechanisms. J R Soc Interface 7(Suppl 2):S241–S256.

Shakhparonov, V.V., Ogurtsov, S.V. 2017. Marsh frogs, *Pelophylax ridibundus*, determine migratory direction by magnetic field. J Comp Physiol A 203:35–43.

**Fish and Turtles:**

Hermans A, Maris T, Hubert J, Rochas C, Scott K, Murk AJ, Winter HV. From subsea power cable to small-spotted catshark Scyliorhinus canicula: Behavioural effects of electromagnetic fields in tank experiments. Mar Environ Res. 208:107127.

James, E., Ford, A. et al. 2025. Female Crabs Are More Sensitive to Environmentally Relevant Electromagnetic Fields from Submarine Power Cables. Environmental Science & Technology Letters. DOI: 10.1021/acs.estlett.5c00862

Landler, L., Painter, M.S., Youmans, P.W., Hopkins, W.A., Phillips, J.B. 2015. Spontaneous magnetic alignment by yearling snapping turtles: rapid association of radio frequency dependent pattern of magnetic input with novel surroundings. PLOS one 10:e0124728.

Lohmann, K. J., Lohmann, C. M. F. 1996. Detection of magnetic field intensity by sea turtles. Nature 380:59-61.

Lohmann, K. J., Lohmann, C. M. F. 1996. Orientation and open-sea navigation in sea turtles*.* J Exp Biol*.* 199:73-81.

Lohmann, K. J., Witherington, B. E., Lohmann, C. M.F., Salmon, M. 1997. Orientation, navigation, and natal beach homing in sea turtles. In: The Biology of Sea Turtles, *edited by* P. Lutz and J. Musick, Boca Raton: CRC Press, pp. 107-135.

Lohmann, K. J., Lohmann, C. M. F. 1998. Migratory guidance mechanisms in marine turtles. J. Avian Biol. 29:585-596.

Luschi, P., Benhamou, S., Girard, C., Ciccione, S., Roos, D., Sudre, J., Benvenuti, S. 2007. Marine turtles use geomagnetic cues during open-sea homing. Curr. Biol. 17:126–133.

Merrill, M.W., Salmon, M. 2010. Magnetic orientation by hatchling loggerhead sea turtles (Caretta caretta) from the Gulf of Mexico. Mar.Biol. 158:101–112.

[Naisbett-Jones, L.C](https://www.ncbi.nlm.nih.gov/pubmed/?term=Naisbett-Jones%20LC%5bAuthor%5d&cauthor=true&cauthor_uid=28416118)., [Putman, N.F](https://www.ncbi.nlm.nih.gov/pubmed/?term=Putman%20NF%5bAuthor%5d&cauthor=true&cauthor_uid=28416118)., [Stephenson, J.F](https://www.ncbi.nlm.nih.gov/pubmed/?term=Stephenson%20JF%5bAuthor%5d&cauthor=true&cauthor_uid=28416118)., [Ladak, S](https://www.ncbi.nlm.nih.gov/pubmed/?term=Ladak%20S%5bAuthor%5d&cauthor=true&cauthor_uid=28416118)., [Young, K.A](https://www.ncbi.nlm.nih.gov/pubmed/?term=Young%20KA%5bAuthor%5d&cauthor=true&cauthor_uid=28416118). 2017. A magnetic map leads juvenile European eels to the gulf stream. [Curr Biol.](https://www.ncbi.nlm.nih.gov/pubmed/28416118) 27:1236-1240.

Naisbett-Jones, L.C., Putman, N.F., Scanlan, M.M., Noakes, D.L. Lohmann, K.J., 2020. Magnetoreception in fishes: the effect of magnetic pulses on orientation of juvenile Pacific salmon. J Exp Biol. 223(pt 1):jeb222091.

[Putman](https://www.cell.com/current-biology/fulltext/S0960-9822(14)00018-9), N.F., [Scanlan](https://www.cell.com/current-biology/fulltext/S0960-9822(14)00018-9), M.M., [Billman](https://www.cell.com/current-biology/fulltext/S0960-9822(14)00018-9), E.J.,  [O’Neil](https://www.cell.com/current-biology/fulltext/S0960-9822(14)00018-9), J.P., [Couture](https://www.cell.com/current-biology/fulltext/S0960-9822(14)00018-9), R.B., Quinn., T.P., Lohmann, K.J., Noakes, D.L.G. 2014. An inherited magnetic map guides ocean navigation in juvenile Pacific salmon. Curr. Biol. 24:446-450.

P[utman, N.F](https://www.ncbi.nlm.nih.gov/pubmed/?term=Putman%20NF%5bAuthor%5d&cauthor=true&cauthor_uid=25056214)., [Jenkins, E.S](https://www.ncbi.nlm.nih.gov/pubmed/?term=Jenkins%20ES%5bAuthor%5d&cauthor=true&cauthor_uid=25056214)., [Michielsens, C.G](https://www.ncbi.nlm.nih.gov/pubmed/?term=Michielsens%20CG%5bAuthor%5d&cauthor=true&cauthor_uid=25056214)., and [Noakes, D.L](https://www.ncbi.nlm.nih.gov/pubmed/?term=Noakes%20DL%5bAuthor%5d&cauthor=true&cauthor_uid=25056214). 2014. Geomagnetic imprinting predicts spatio-temporal variation in homing migration of pink and sockeye salmon. [J R Soc Interface.](https://www.ncbi.nlm.nih.gov/pubmed/25056214) 11:20140542.

[Putman, N.F](https://www.ncbi.nlm.nih.gov/pubmed/?term=Putman%20NF%5bAuthor%5d&cauthor=true&cauthor_uid=24899681)., [Meinke, A.M](https://www.ncbi.nlm.nih.gov/pubmed/?term=Meinke%20AM%5bAuthor%5d&cauthor=true&cauthor_uid=24899681)., and [Noakes, D.L](https://www.ncbi.nlm.nih.gov/pubmed/?term=Noakes%20DL%5bAuthor%5d&cauthor=true&cauthor_uid=24899681). 2014*.* Rearing in a distorted magnetic field disrupts the 'map sense' of juvenile steelhead trout. [Biol Lett.](https://www.ncbi.nlm.nih.gov/pubmed/24899681) 10: pii: 20140169.

Putman, N.F., Williams, C.R., Gallagher, E.P., Dittman, A.H. 2020. A sense of place: pink salmon use a magnetic map for orientation. J Exp Biol 223: jeb218735.

Quinn, T.P., Merrill, R.T., Brannon, E.L. 2005. Magnetic field detection in Sockeye salmon. J Exp Zool 217:137-142.

Xu P, Wang B, Wang Z, Jin R, Ahmad M, Shang Y, Hu M, Chen F, Khalil MF, Huang W, Wang Y. Effects of electromagnetic radiation from offshore wind power on the physiology and behavior of two marine fishes. Mar Pollut Bull. 213:117633.

**Flora: (There are over 200 studies on plants alone)**

Belyavskaya, N.A. 2001. Ultrastructure and calcium balance in meristem cells of pea roots exposed to extremely low magnetic fields. Adv. Space Res. 28: 645-450.

Czerwińskia M, Januszkiewicz L, Vian A, Lázaro A. 2020. The influence of bioactive mobile telephony radiation at the level of a plant community – possible mechanisms and indicators of the effects. Ecol Indicat 108:105683.

Halgamuge MN. 2017. Weak radiofrequency radiation exposure from mobile phone radiation on plants. Electromagn Biol Med 36:213–35.

Halgamuge, M.N., Davis, D. 2019. Lessons learned from the application of machine learning to studies on plant response to radio-frequency. Environ Res. 178:108634

Panda DK, Das DP, Behera SK, Dhal NK. 2024. Review on the impact of cell phone radiation effects on green plants. Environ Monit Assess. 196:565.

[Vian, A](http://www.ncbi.nlm.nih.gov/pubmed?term=%22Vian%20A%22%5bAuthor%5d&itool=EntrezSystem2.PEntrez.Pubmed.Pubmed_ResultsPanel.Pubmed_RVAbstract)., [Roux, D](http://www.ncbi.nlm.nih.gov/pubmed?term=%22Roux%20D%22%5bAuthor%5d&itool=EntrezSystem2.PEntrez.Pubmed.Pubmed_ResultsPanel.Pubmed_RVAbstract)., [Girard, S](http://www.ncbi.nlm.nih.gov/pubmed?term=%22Girard%20S%22%5bAuthor%5d&itool=EntrezSystem2.PEntrez.Pubmed.Pubmed_ResultsPanel.Pubmed_RVAbstract)., [Bonnet, P](http://www.ncbi.nlm.nih.gov/pubmed?term=%22Bonnet%20P%22%5bAuthor%5d&itool=EntrezSystem2.PEntrez.Pubmed.Pubmed_ResultsPanel.Pubmed_RVAbstract)., [Paladian, F](http://www.ncbi.nlm.nih.gov/pubmed?term=%22Paladian%20F%22%5bAuthor%5d&itool=EntrezSystem2.PEntrez.Pubmed.Pubmed_ResultsPanel.Pubmed_RVAbstract)., [Davies, E](http://www.ncbi.nlm.nih.gov/pubmed?term=%22Davies%20E%22%5bAuthor%5d&itool=EntrezSystem2.PEntrez.Pubmed.Pubmed_ResultsPanel.Pubmed_RVAbstract)., [Ledoigt, G](http://www.ncbi.nlm.nih.gov/pubmed?term=%22Ledoigt%20G%22%5bAuthor%5d&itool=EntrezSystem2.PEntrez.Pubmed.Pubmed_ResultsPanel.Pubmed_RVAbstract). 2006. Microwave irradiation affects gene expression in plants. Plant Signal Behav. 1:67-70.

[Vian, A](https://www.ncbi.nlm.nih.gov/pubmed/?term=Vian%20A%5bAuthor%5d&cauthor=true&cauthor_uid=26981524)., [Davies, E](https://www.ncbi.nlm.nih.gov/pubmed/?term=Davies%20E%5bAuthor%5d&cauthor=true&cauthor_uid=26981524)., [Gendraud, M](https://www.ncbi.nlm.nih.gov/pubmed/?term=Gendraud%20M%5bAuthor%5d&cauthor=true&cauthor_uid=26981524)., [Bonnet, P](https://www.ncbi.nlm.nih.gov/pubmed/?term=Bonnet%20P%5bAuthor%5d&cauthor=true&cauthor_uid=26981524). 2016. Plant responses to high frequency electromagnetic fields. [Biomed Res Int.](https://www.ncbi.nlm.nih.gov/pubmed/26981524) 2016:1830262.

Xie Y, Guo J, Su T, Tan B, Huang L, Lai J. 2025. Growth and defense mechanism of Phaeocystis globosa exposed to extremely low frequency electromagnetic fields. Ecotoxicol Environ Saf. 301:118507.
