# Supplementary material for: Flora and fauna: how nonhuman species interact with natural and man-made EMF at ecosystem levels and public policy recommendations
Source: Front Public Health. 2025 Nov 19;13:1693873. doi: 10.3389/fpubh.2025.1693873 (PMC12675998; doi:10.3389/fpubh.2025.1693873)
Supplement: Supplementary file 3 [file Supplementary_file_3.docx]

**November 3, 2025**

**Supplement Table 3**

**Recommendations for U.S. Agency Reform and Co-operation**

| **Environmental Protection Agency** | **U.S. Fish and Wildlife Service** | **Federal Communications Commission** |
| --- | --- | --- |
| - Define airspace as habitat: EPA & relevant agencies should recognize EMF as biologically active pollution to air, ground and water; regulate like other pollutants.    - “Airspace as habitat” provides a legal foundation to assess cumulative EMF impacts and mitigate exposures as many wildlife species depend on airspace for migration, mating, foraging, and territorial defense.    - Launch dedicated research program to evaluate biological and ecological impacts of RF; treat it as environmental pollutant akin to lead and pesticides.   - Determine what ICRP flora/fauna protection for ionizing radiation can/does apply to nonionizing protection; institute regulations according to ethical and biological constraints.   - Program should include hazard identification, long-term exposure studies, cumulative risk assessments, synergies with other environmental stressors with focus on human health and wildlife.    - Ensure rigorous independent science separate from industry influence guides development of science-based, federally developed safety standards.   - Maintain at least one bioelectromagnetics scientist to guide programs. | - Re-initiate inter-agency USFWS/NTIA EIS collaboration that provides framework for preliminary research design for bird radiation study (already developed).    - Acknowledge/ address EMF effects to migratory birds, federally listed fish and wildlife, and other protected plants and animals.    - Create wildlife exposure standards to avoid or minimize “take” of birds/listed species to include chronic, low-level EMF exposures.    - Amend USFWS Dec. 2021 updated “take” rule that allowed “incidental take” to include known/suspected impacts from EMF radiation on migratory birds. | - FCC should respond to Court mandate in *EHT et al v. FCC*.  - Request relevant expert agencies evaluate current scientific evidence on flora and fauna.  - Request relevant agencies address data gaps.  - Update regulations to include wildlife protection for chronic, low-level exposures.   - Include thorough assessment of RF effects in tower permitting; incorporate rulemaking changes regarding “effects of communication towers on migratory birds.”     - Work with USFWS to help private landowners develop ESA Section 10 Habitat    - Conservation Plans in “critical habitat” where listed species recovery goals are designated and cell tower/other radiation impacts are at issue.  - Establish transparent nationwide measuring & monitoring program for environmental oversight  - Include centralized, publicly accessible registry of all existing/proposed cell towers, 4G, 5G, small cell base stations.  - Prioritize ecologically sensitive areas: national & state parks, forests, wildlife refuges, wetlands, coastal ecosystems, migratory corridors, designated wilderness areas, and human populations in other areas.  - Monitoring program should collaborate with environmental agencies; include enviro surveillance; track effects to flora and fauna including behavior, reproduction, population trends in high-exposure areas to ID risks; inform responsible infrastructure deployment, just as public health systems track human populations.  - Align environmental review with NEPA; restore public accountability; develop NEPA-compliant procedures to assess cumulative impacts on wildlife, insects, flora.  - Require documentation of categorically excluded (CE) facilities like small cells; conduct environmental assessments; include RF compliance reports; make publicly accessible.  - Conduct regular, independent spot checks for RF levels pre- and post-construction mandated for all base stations.  - No longer allow industry to determine extent/need for environmental review.  - Public notice/comment periods must be meaningful, transparent, genuinely considered in decision-making.  - Implement NEPA reviews for all satellite systems, present and proposed.  - Request congressional funding for at least one staff wildlife biologist. |
